# Clues to Evolution of the SERA Multigene Family in 18 *Plasmodium* Species

**DOI:** 10.1371/journal.pone.0017775

**Published:** 2011-03-15

**Authors:** Nobuko Arisue, Satoru Kawai, Makoto Hirai, Nirianne M. Q. Palacpac, Mozhi Jia, Akira Kaneko, Kazuyuki Tanabe, Toshihiro Horii

**Affiliations:** 1 Department of Molecular Protozoology, Research Institute for Microbial Diseases, Osaka University, Suita, Osaka, Japan; 2 Laboratory of Tropical Medicine and Parasitology, Dokkyo University School of Medicine, Mibu, Shimotsuga, Tochigi, Japan; 3 Department of Parasitology, Graduate School of Medicine, Gunma University, Maebashi, Gunma, Japan; 4 Department of Parasitology, Osaka City University Graduate School of Medicine, Osaka, Japan; 5 Island Malaria Group, Department of Microbiology, Tumor and Cell Biology, Karolinska Institutet, Stockholm, Sweden; 6 Institute of Tropical Medicine, Nagasaki University, Nagasaki, Japan; 7 Laboratory of Malariology, Research Institute for Microbial Diseases, Osaka University, Suita, Osaka, Japan; Institut national de la santé et de la recherche médicale - Institut Cochin, France

## Abstract

SERA gene sequences were newly determined from 11 primate *Plasmodium* species including two human parasites, *P. ovale* and *P. malariae*, and the evolutionary history of SERA genes was analyzed together with 7 known species. All have one each of Group I to III cysteine-type SERA genes and varying number of Group IV serine-type SERA genes in tandem cluster. Notably, Group IV SERA genes were ascertained in all mammalian parasite lineages; and in two primate parasite lineages gene events such as duplication, truncation, fragmentation and gene loss occurred at high frequency in a manner that mimics the birth-and-death evolution model. Transcription profile of individual SERA genes varied greatly among rodent and monkey parasites. Results support the lineage-specific evolution of the *Plasmodium* SERA gene family. These findings provide further impetus for studies that could clarify/provide proof-of-concept that duplications of SERA genes were associated with the parasites' expansion of host range and the evolutionary conundrums of multigene families in *Plasmodium*.

## Introduction

Malaria, caused by the genus *Plasmodium*, is one of the most serious infectious diseases prevalent in the tropics. There were an estimated 243 million cases and 863,000 malaria deaths in 2008 (WHO, 2009). The emergence of drug-resistant parasites has made its control more difficult than before and, thus, a better understanding of the biology of malaria parasites is required to gain insights into new effective control measures including malaria vaccines and new antimalarial drugs. The genome of *Plasmodium* presents basic information for this understanding. One of the prominent features of *Plasmodium* genomes is the presence of various unique multigene families, such as *the Plasmodium* interspersed repeats, *pir* [1]. The *pir* families are highly species-specific, suggesting evolution of lineage-specific immune evasion mechanisms. By far the best documented of multigene families is the *var* gene family of *P. falciparum*, the most virulent human malaria parasite. Products of *var* genes appear on the surface of infected erythrocytes and are involved in antigenic variation to evade host immunity. Other species-specific gene families encode proteins involved in host cell invasion, *e.g.* rhoptry proteins and parasite surface antigens, merozoite surface protein-3 and -7. In sharp contrast to a very large number (several hundreds) of rRNA gene family in other eukaryotes [2], [3], *Plasmodium* has a very limited array (n = 4–7 units). Thus, *Plasmodium* possesses unique multigene family members in its genome with distinctive evolutionary conundrums.

*Plasmodium* also has the serine repeat antigen (SERA) multigene family that encodes proteins with a putative papain-like cysteine protease motif. In *P. falciparum*, SERA5 (Pfa-SERA5), one of nine SERAs, is a vaccine candidate now on phase Ib clinical trial in Uganda [4]. Serum antibodies against the N-terminal domain of Pfa*-*SERA5 in individuals living in malaria endemic areas protect infants from clinical malaria and inhibit *in vitro* parasite growth [4]–[7]. These studies and previous vaccine trials using laboratory animals [4], [8]–[10] suggest the N-terminal domain of Pfa-SERA5 as a promising malaria vaccine candidate. Pfa-SERA5 (120 kDa) is abundantly produced at the late trophozoite to schizont stages of parasite development [7], [11]–[12], is secreted together with other SERAs into the parasitophorous vacuole in an infected erythrocyte [7], and is processed into three fragments: the N-terminal domain (47 kDa), the central domain containing putative papain-like cysteine protease motif (56 kDa) and the C-terminal domain (18 kDa). The N-terminal 47 kDa fragment is further processed into two 25 kDa fragments, linked with the C-terminal 18 kDa fragment via disulfide bonding, and attach to the merozoite surface. The central fragment, before being shed to the medium, is further processed to 50 kDa and 6 kDa fragments [12]–[14]. Several protease inhibitors have been identified to block proteolytic processing of Pfa-SERA5 resulting to a developmental arrest at schizont rupture/merozoite release [13]. Pfa-SERA5 processing is mediated by a subtilisin-like serine protease called PfSUB1 and the inhibition of this processing, likewise, results in blockade of merozoite release [15], [16]. The precise molecular mechanism(s) of parasite egress from an infected erythrocyte, however, remains to be determined.

Previous evolutionary studies of SERA genes from eight *Plasmodium* species have shown that these can be categorized into Groups I to IV, according to gene structure and phylogenetic relatedness [17]. Groups I to III and Group IV SERA genes encode proteins with protease motif that either have cysteine or serine residue, respectively, in the catalytic site. The SERA multigene family of *P. falciparum* (Pfa-SERA1 to Pfa-SERA8) is clustered head-to-tail on chromosome 2 between a conserved hypothetical protein (HP) gene and a putative iron-sulfur assembly protein gene (hesB) [3]. Another SERA gene, Pfa-SERA9, is located on chromosome 9. The gene synteny of the clustered SERA multigene family is conserved among *Plasmodium* species examined, except for SERA3 of *P. gallinaceum*, an avian parasite [17]. The number of SERA genes in the clustered region varies among parasite species, from two in *P. gallinaceum* to 12 in *P. vivax*, the benign human malaria parasite. Outside the genus *Plasmodium*, no apicomplexan parasite has a SERA ortholog, except *Theileria*, a closely related protozoan parasite of cattle, which has one SERA ortholog [17]. The evolutionary process of the *Plasmodium* SERA multigene family, however, remains largely unknown. Also, no study has been done for SERA multigene families of human malaria parasites, *P. malariae* and *P. ovale*; and *P. vivax*-related monkey malaria parasites. Since *P. vivax* became a human parasite by host switch from a monkey parasite, it is worth to see whether *P. vivax* SERA gene family underwent unique evolution, distinctive from closely related monkey parasites.

In this study, in our attempt to unravel the evolutionary history of the SERA gene family of *Plasmodium*, we newly determined SERA genes from 11 primate *Plasmodium* species: nine *P. vivax*-related monkey parasites; and *P. malariae* and *P. ovale.* Together with previously reported SERA sequences, we performed evolutionary analyses of the gene family. Results obtained here show that the number of SERA genes remarkably differs among parasite lineages and the variation in mammalian parasites was found only in serine-type SERA gene but not in cysteine-type SERA gene. We noted that the gene number variation occurred lineage-specifically, which was particularly evident in human, ape and monkey parasite groups. In addition, we found that transcription of individual SERA genes varied greatly among rodent and monkey parasites, supporting lineage-specific evolution of the *Plasmodium* SERA gene family.

## Results

### Arrangement of SERA multigene family in 18 *Plasmodium* species

**Figure 1:**
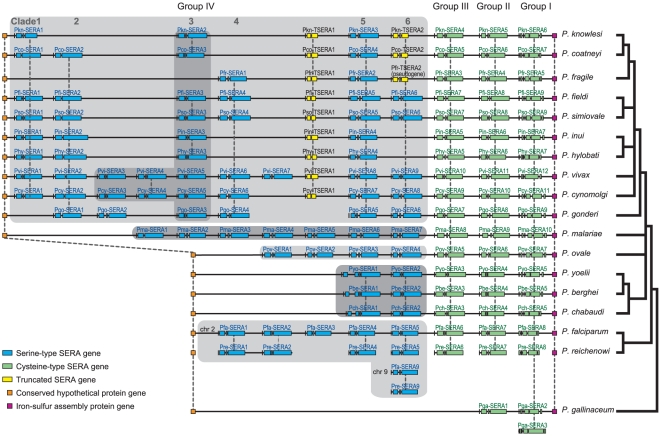
The organization of the SERA gene family in 18 *Plasmodium* species. SERA genes are arrayed onto a solid horizontal line for each parasite species. Gene arrangement follows the genomic organization in each species. Individual SERA genes are clustered between a conserved hypothetical gene and the iron-sulfur assembly protein gene. Pfa-SERA9, Pre-SERA9 and PgaSERA3 have aberrant locations. SERA genes were categorized to Groups I to IV and Clades 1 to 6 reflecting orthologous gene groups as inferred from phylogenetic analyses shown in Figure 2 and Figure S3. SERA genes of Groups I to III (cysteine-type SERA gene) and those of Group IV (serine-type SERA gene) are shown in green and blue, respectively. TSERA denotes truncated SERA genes shown in yellow. SERA genes are drawn to scale, but other genes and intergenic regions are not. Dashed lines and/or dark gray boxes denote orthologous relationships. A generally accepted consensus phylogenetic tree of *Plasmodium* species is shown in right. The abbreviations for species names are: *P. falciparum* (Pfa), *P. vivax* (Pvi), *P. malariae* (Pma), *P. ovale* (Pov), *P. reichenowi* (Pre), *P. gonderi* (Pgo), *P. fragile* (Pfr), *P. coatneyi* (Pco), *P. knowlesi* (Pkn), *P. inui* (Pin), *P. fieldi* (Pfi), *P. simiovale* (Pso), *P. cynomolgi* (Pcy), *P. hylobati* (Phy), *P. yoelii* (Pyo), *P. berghei* (Pbe), *P. chabaudi* (Pch), and *P. gallinaceum* (Pga). The SERA gene family has common exon/intron structure: four exons and three introns, with some exceptions. Group I SERA genes have six exons and five introns structure, except for Pfa-SERA8 and Pvi-SERA12, which lack one intron. SERA genes of Group IV Clade 2 and Pma-SERA1 have no third intron and consist of three exons and two introns. Group I SERA genes of three rodent parasites have an extra intron near the 5′-end. Pkn-SERA1 gene in Clade 1 contains three stop codons, causing truncation of the cysteine-rich conserved domain; but since this gene was expressed, we consider this is a SERA gene. TSERA1 genes have truncations of the protease domain, variable domain 2 and cysteine-rich conserved domain. Pco-TSERA2, Pkn-TSERA2 and Pfr-TSERA2 lack a long amino acid region (498 residues) including the enzyme domain (Figure S4), of which Pfr-TSERA2 seems to be a putative pseudogene because its 2nd exon contains two stop codons.

The 18 *Plasmodium* species analyzed in this study and their natural hosts are: *P. falciparum*, *P. vivax*, *P. malariae* and *P. ovale* (humans); *P. reichenowi* (chimpanzees); *P. gonderi* (African Old World monkeys); *P. fragile*, *P. coatneyi*, *P. knowlesi*, *P. inui*, *P. fieldi*, *P. simiovale*, and *P. cynomolgi* (Asian Old World monkeys), *P. hylobati* (gibbons), *P. yoelii*, *P. berghei* and *P. chabaudi* (rodents); and *P. gallinaceum* (birds) [Underline denotes newly determined SERA sequences in this study]. *P. vivax* is closely related to Asian Old World monkey parasites [18]. Additionally, it should be mentioned that primate malaria parasites are phylogenetically classified into two distinct groups: group 1 for *P. falciparum* and *P. reichenowi*; and group 2 for *P. malariae*, *P. ovale*, *P. vivax* and the nine Old World monkey parasite species [19], [20]. A total of 116 new SERA gene sequences, of which 11 and 18 are truncated genes and pseudogenes, respectively (Table 1 and Figure S1) were analyzed together with 47 SERA genes previously published from seven *Plasmodium* species (Table 1 and Figure S1). Details of SERA genes, truncated genes and pseudogenes are described in Figure S1. A SERA gene map that follows the genomic organization of the genes is shown in Figure 1.

**Table 1:** Summary of the *Plasmodium* SERA genes determined and analyzed in this study.

Species (strain^a^)	SERA gene	Truncated SERA gene	Fragmented or Pseude SERA gene	Accession number (bp)
*P. malariae* (Kisii67)	10	0	3	AB576870 (66,408)
*P. ovale* (Nigeria II)	7	0	1	AB576871 (45,833)
*P. cynomolgi* (Mulligan)	11	1	2	AB576872 (67,118)
*P. fieldi* (N-3)	9	1	2	AB576873 (56,691)
*P. simiovale*	9	1	2	AB576874 (56,056)
*P. inui* (Celebes)	7	1	4	AB576875 (53,234)
*P. hylobati* (WAK)	7	1	0	AB576876 (41,987)
*P. coatneyi* (CDC)	7	2	1	AB576877 (44,850)
*P. knowlesi* (H)	6	2	0	AB576878 (40,735)
*P. fragile* (Hackeri)	5	2^b^	2	AB576879 (34,519)
*P. gonderi*	9	0	1	AB576880+AB576881 (22,367+27,149)
*P. vivax* (SalI)	12	1	1	See Table S2
*P. berghei* (ANKA)	5	0	0	See Table S2
*P. yoelii* (17NXL)	5	0	0	See Table S2
*P. chabaudi* (AS)	5	0	0	See Table S2
*P. falciparum* (3D7)	9	0	0	See Table S2
*P. reichenowi* (Oscar)	8^c^	0	0	Ref. 17
*P. gallinaceum*	3	0	0	Ref. 17

a ATCC numbers: P. cynomolgy = 30155, P. fieldi = 30163, P. simiovale = 30104, P. hylobati = 30154, P. knowlesi = 30158, P. gonderi = 30045.

b One of two truncated SERA genes is also a putative pseudogene.

c SERA gene sequences of P. reichenowi is not complete.

**Figure 2:**
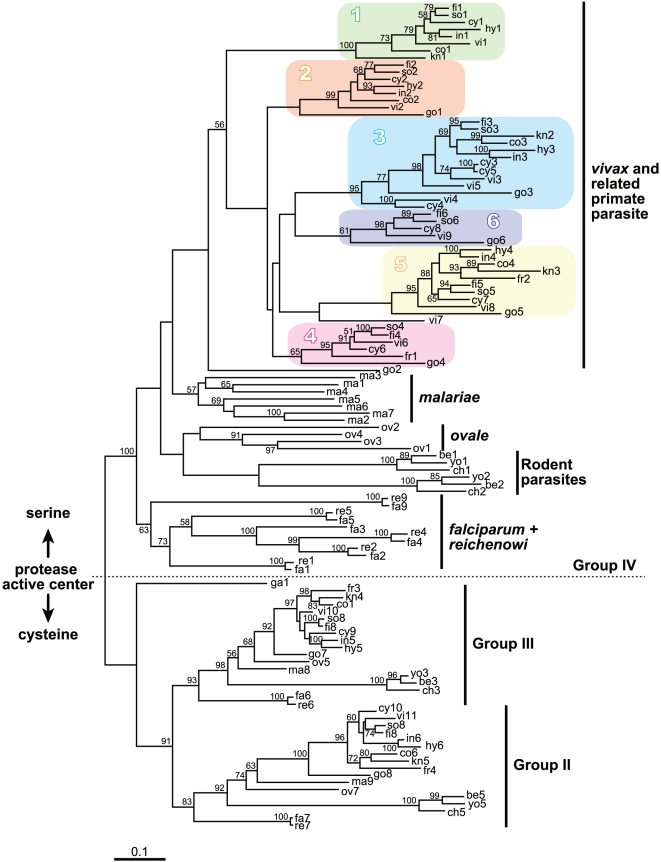
The maximum likelihood (ML) phylogenetic tree of *Plasmodium* SERA genes. This unrooted tree was constructed from 115 SERA genes (encompassing Groups II to IV, see Figure 1) using 570 amino acid positions under the JTT + Γ (eight categories) model (α = 1.15) with 500 heuristic replicates. Bootstrap proportions >50% are shown along nodes. Groups II - III and Group IV are cysteine-type and serine-type SERA genes, respectively. Note that Pga-SERA1 (ga1) is an offshoot of Groups II and III SERA genes, suggesting the occurrence of a common ancestor, leading to Pga-SERA1 (ga1) and a common ancestor of Group II and Group III. In *P. vivax* and *P. vivax*-related monkey malaria parasite species, the six clades are color-boxed. Pgo-SERA1 (go1) and Pgo-SERA5 (go5) were grouped, despite low bootstrap values, into Clade 2 and Clade 5 respectively, because these genes showed common features to each clade in exon/intron structure and/or gene array.

Almost all SERA genes were clustered between two conserved genes; a conserved hypothetical protein (HP) gene and the iron-sulfur assembly protein gene (hesB) (Figure 1 and Figure S1). Except for one SERA gene from *P. falciparum*, *P. reichenowi* and *P. gallinaceum* that are located outside the clustered region, all SERA genes lie disposed head-to-tail on the chromosomes where they were found. The number of SERA genes in the cluster varied from 2 (*P. gallinaceum*) to 12 (*P. vivax*) among 18 *Plasmodium* species. All parasite species have one SERA gene from Group I, Group II and Group III (cysteine-type SERA gene), with the exception of *P. gallinaceum*, which has only two SERA genes, one from Group I and the other from a common ancestor of Groups II and III (Figure 2). Thus, the difference in the number of SERA genes among the 17 *Plasmodium* species lies in Group IV (serine-type SERA gene). Likewise, while orthologous relationship of SERA genes in Groups I to III were readily identified from sequence similarity and phylogenetic analysis (Figure 1 and Figure 2), the relationship was identified only for some (but not all) SERA genes in Group IV (Figure 1, orthologous gene groups supported by phylogenetic analyses (below) are indicated by vertical dashed- lines). The high divergence of SERA genes including variations in gene number was notable only in Group IV SERA genes in mammalian malaria parasites. In particular, the number of Group IV SERA genes remarkably increased in two primate parasite lineages.

### Primary structure of SERA genes

The SERA gene family has a common exon/intron structure: four exons and three introns, with few exceptions. SERA genes of Group I have six exons and five introns structure, except for Pfa-SERA8 and Pvi-SERA12, which lack one intron. Other exceptions are detailed in Figure 1. Pcy-SERA3 was closely related to Pcy-SERA5 but not to Pvi-SERA3 (Figure 2), and Pco-SERA1 showed a fusion of exon 3 and 4, which is similar to Pco-SERA2 (Figure 1). These genes were generated probably by gene conversion between Pcy-SERA3 and Pcy-SERA5, and Pco-SERA1 and Pco-SERA2, respectively. SERA was originally named after tandem repeats of serine residues in Pfa-SERA5 (and Pre-SERA5, an ortholog of Pfa-SERA5) [21]. Here, we found 47 tandem repeats of serine in Pma-SERA8. The position of serine repeats was, however, markedly different between Pma-SERA8 (in variable domain 2) and Pfa-SERA5/Pre-SERA5 (in variable domain 1) (Figure S2).

**Figure 3:**
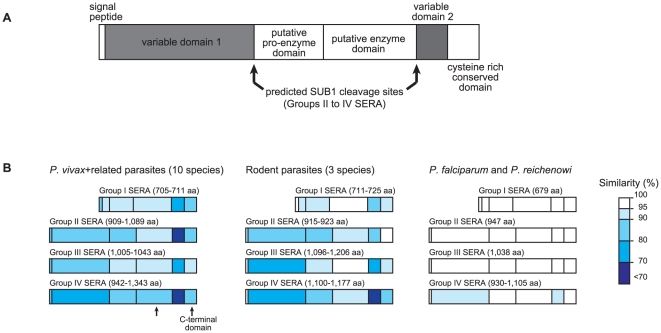
Primary structure and inter-species sequence variation of the *Plasmodium* SERA gene. The putative domain structure of the gene family is shown in (A). In (B) are sequence variations in Groups I to IV SERA domains using amino acid sequence similarity for three parasite lineages: (i) *P. vivax* and *P. vivax*-related monkey malaria parasite species, (ii) three rodent parasite species, and (iii) *P. falciparum* and *P. reichenowi*. Percent (%) similarity is color coded.

Amino acid sequence alignments reveal the consensus primary structure of SERA genes (Figure 3A, % similarity is color coded). Downstream of the signal peptide sequence at the N-terminus, is a sequence region (variable domain 1), in which extensive sequence variations are found among parasite species. At the central domain of Pfa-SERA5, functional genetic and structural analyses identified the pro-enzyme and enzyme domain [22], [23], flanked by the reported PfSUB1 cleavage sites [15]. Pfa-SERA4 (Group IV) and Pfa-SERA6 (Group II) were likewise cleaved by recombinant PfSUB1 [15].

The consensus sequence of the cleavage site is (Val/Leu/Ile)-Xaa-(Gly/Ala)-Paa, in which Xaa is any amino acid residue and Paa is a non-polar residue except for Leu [15]. The consensus sequence is well conserved with slight modifications in all Group II to IV *Plasmodium* SERA genes analyzed here (Figure S2). In the C-terminal region, there occur interspecies variable sequence region (variable domain 2) and interspecies conserved sequence region, in which 7 cysteine residues are perfectly conserved in all SERA genes (Figure S2). Degenerating oligopeptide tandem repeats were found in both variable domains 1 and 2. Group I SERA genes lack most of the N-terminal variable domain 1 and SUB1 cleavage sites.

Amino acid sequence variations of Groups I to IV SERA genes varied greatly among parasite lineages (Figure 3B), with the highest variation in *P. vivax* and related monkey malaria parasite lineage. In *P. falciparum* and *P. reichenowi*, sequences are highly conserved in all domains of Groups I to IV, except for Group IV variable domains 1 and 2. In rodent parasite lineage, sequences are somewhat variable in the two variable domains of Groups I to IV, but relatively conserved in both the putative pro-enzyme and enzyme domains. In *P. vivax* and related monkey parasites, putative pro-enzyme and putative enzyme domains remains relatively conserved compared to two variable domains whose sequence variations are very high. Thus, overall, although putative pro-enzyme and putative enzyme domains seem fairly conserved, the extent of variation in variable domains 1 and 2 differs greatly among parasite lineages.

### Phylogenetic analyses of *Plasmodium* SERA genes

The maximum likelihood (ML) phylogenetic tree was constructed from 134 SERA genes representing 18 *Plasmodium* species, using 392 unambiguously aligned amino acid sites (Figure S3). The best tree shows that genes were categorized into four major groups, Groups I to Group IV. The monophyletic grouping of Group I SERA genes is supported with 100% bootstrap value. The long internal branch separating Group I from Groups II to IV suggests that the root of the tree is located on the branch leading to the common ancestor of the Group I SERA genes. It is thus likely that Group I genes have appeared early in the evolution of SERA gene family, being consistent with our previous analysis [17].

To enhance/increase the resolution of the ML tree, the long branched Group I genes were excluded from analysis and the ML phylogenetic tree was constructed from 115 Group II to Group IV SERA genes using 570 amino acid sites. The monophyletic grouping of Group II, III and IV SERA genes is supported by 82%, 95% and 100% bootstrap values, respectively (Figure 2). Group IV SERA genes, which diverged after the substitution of cysteine to serine in the catalytic site of the cysteine protease motif, were further categorized to five monophyletic sub-Groups: (i) *P. falciparum* and *P. reichenowi*, (ii) rodent *Plasmodium* species, (iii) *P. ovale*, (iv) *P. malariae* and (v) *P. vivax* and *P. vivax*-related monkey parasite species (Figure 2). Closer examination of these sub-groups revealed several notable features. First, in lineages which contain multiple parasite species, internal branches form sub-lineages, from which orthologous relationship of SERA genes are evident. For example, in three rodent parasites, SERA1 genes and SERA2 genes are separable with 100% bootstrap value. Similar separations can be seen in lineages of *P. falciparum*/*P reichenowi*, and *P. vivax* and related monkey parasites. These indicate that Group IV SERA genes were duplicated independently in each of the three sub-group lineage: *P. falciparum*/*P. reichenowi*, rodent parasites, and *P. vivax* and related monkey parasites.

**Figure 4:**
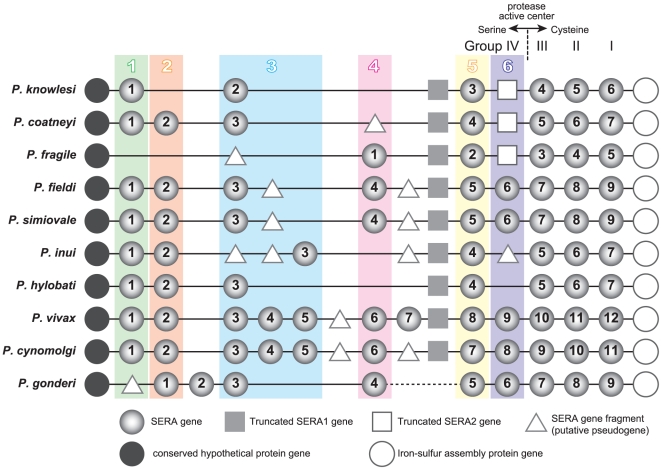
The SERA gene organization of *P. vivax* and *P. vivax*-related monkey malaria parasites. Six monophylic clades of Group IV SERA genes, Clade 1 to Clade 6 are designated in colored boxes. SERA genes, truncated SERA genes and SERA gene fragments are shown by light gray circle, square, and triangle, respectively. The conserved hypothetical protein gene at 5′-end and the iron-sulfur assembly protein gene at the 3′-end are shown by dark gray circle and open circle, respectively.

Group IV SERA genes of *P. vivax* and *P. vivax*-related monkey parasites (10 species) were further categorized into six orthologous gene groups (Clade 1 to Clade 6), except for Pgo-SERA2 and Pvi-SERA7 (Figure 2). The number of SERA genes varies from 5 (*P. fragile*) to 12 (*P. vivax*). Orthologous relationships of the SERA genes and their locations are shown in Figure 4. Each clade has 5 (Clade 6) to 10 (Clade 5) parasite species. This does suggest that a common ancestor of *P. vivax* and related monkey malaria parasites had at least 6 SERA genes of Group IV, followed by gene duplications and gene deletions in each lineage. Pgo-SERA2 and Pvi-SERA7 have no orthologous genes. Pgo-SERA2 was located at the earliest branching position in Group IV SERA genes (Figure 2). An ortholog of Pgo-SERA2 was possibly lost in a common ancestor of Asian Old World monkey parasites, after divergence from a common ancestor of African and Asian Old World monkey parasites. Although Pvi-SERA7 has no ortholog in other species, several parasite species have a SERA gene fragment just upstream of TSERA1 (Figure 4), which is similar to Pvi-SERA7. Since we failed to obtain *P. gonderi* sequences corresponding to Pvi-SERA7 and TSERA1 orthologs, we cannot infer further on the origin of these genes. In Group IV, there are notably many SERA gene fragments and pseudogenes containing multiple stop codons. Taken together, extensive gene duplications, gene deletions as well as pseudogenization/truncation are evident in the serine type SERA gene (Group IV) of *P. vivax* and related monkey malaria parasites.

### Transcription analysis of SERA gene

**Figure 5:**
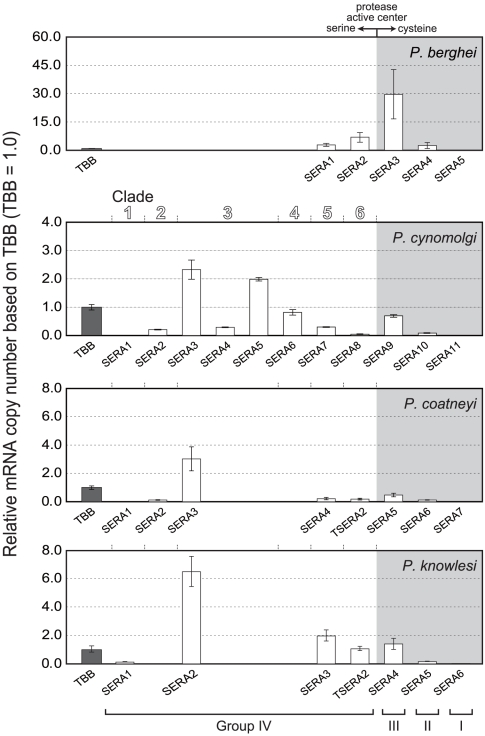
Transcription of the SERA gene family of rodent parasite *P. berghei*, and three monkey parasites, *P. cynomolgi*, *P. coatneyi* and *P. knowlesi*. Parasites at the late trophozoite to schizont stages were used for analyses. Relative amounts of transcribed SERA genes were standardized relative to β-tubulin (TBB), set at 1.0. Cysteine-type SERA genes (Groups I, II and III) and serine-type SERA genes (Group IV) are arrayed in gray shaded areas and unshaded areas, respectively. SERA genes of three monkey parasites are separated into six clades, as designated on top of the *P. cynomolgi* panel.

Transcription of SERA genes was analyzed for the late trophozoite to schizont stages of the rodent parasite, *P. berghei*, and monkey parasites *P. knowlesi*, *P. cynomolgi* and *P. coatneyi*. The amount of each SERA gene transcript is presented relative to that of β-tubulin in Figure 5. In *P. berghei*, Pbe-SERA3 (Group III SERA gene) was predominantly expressed followed to a lesser extent by other SERA genes except for SERA 5 that did not show detectable expression. In three monkey parasites, the abundantly expressed genes are members of Group IV Clade 3: Pcy-SERA3 and Pcy-SERA5, Pco-SERA3 and Pkn-SERA2. Other SERA genes of Group IV, except Pcy-SERA1 and Pco-SERA1, were also expressed to varying degrees. All Group III SERA gene expression was evident; whereas, no expression was observed for all Group I SERA genes. From above, SERA genes were differently expressed between rodent and primate parasites, with the exception of Group I SERA genes, which were not expressed at the blood stage parasites of both *P. berghei* and three monkey parasites.

## Discussion

**Figure 6:**
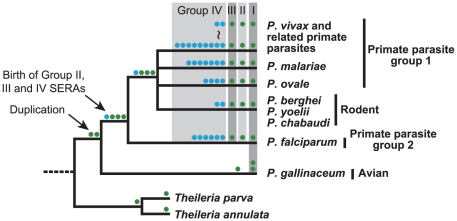
Inferred gene duplication events in the evolution of the *Plasmodium* SERA gene family. Each colored circle represents cysteine-type SERA gene (Groups I to III, green circle) and serine-type SERA genes (Group IV, blue circle), and is placed alongside parasite lineages of a generally accepted phylogenetic tree of *Plasmodium*, with *Theileria* used as an outgroup.

This study presents an overview of the evolution of the *Plasmodium* SERA gene family (Figure 6). Since the genus *Theileria*, closely related to *Plasmodium*, has only one SERA gene of the cysteine-type, it is inferred that cysteine-type SERA gene initially duplicated in a common ancestor of all *Plasmodium* species. One of the duplicated genes became a common ancestor of a SERA gene of Groups II and III. Another duplication took place in a lineage leading to *P. gallinaceum* to form two SERA genes of Group I. In a common mammalian malaria parasite ancestor, the common ancestral SERA gene was duplicated which subsequently generated two cysteine-type SERA genes (Group II and Group III). Following divergence of mammalian malaria parasite species, a lineage leading to *P. falciparum* and *P. reichenowi* (primate parasite group1) had 5– 6 SERA genes of Group IV by multiple gene duplications. In the lineage leading to three rodent parasites, a Group IV SERA gene was duplicated. In lineages of primate parasite group2 (*P. malariae*, *P. ovale*, and *P. vivax* and related monkey parasites), gene duplications of Group IV SERA gene took place in each parasite lineage resulting to 4 SERA genes in *P. ovale*, 7 SERA genes in *P. malariae* and 2–9 SERA genes in *P. vivax* and related monkey parasites. Simultaneously, gene deletions as well as pseudogenization and truncation also took place in Group IV SERA genes of *P. vivax* and related monkey parasites. It is notable that gene duplication events occurred only in Group IV SERA genes in mammalian parasites and this duplication was particularly frequent in parasite species that infect humans, apes and monkeys.

In general, duplicated genes undergo either (i) concerted evolution or (ii) birth-and-death evolution [2]. Homogenization of duplicated genes by gene conversion drives concerted evolution as evident in the rRNA multigene families of vertebrates; while duplicated genes show divergence by independent diversification processes to result in birth-and-death evolution, e.g., the major histocompatibility complex (MHC) gene families of mammals. The birth-and-death evolution model applies to most multigene families. The model assumes that new genes are created by repeated gene duplications; and that duplicated genes can be maintained in the genome for a long time, whereas others become deleted or nonfunctional through deleterious mutations [2]. The observed gene duplication and gene deletion found in the *Plasmodium* SERA genes is in concordance with the birth-and-death model, though traits of gene conversion are detected in a few Group IV SERA genes of limited parasites. The birth-and-death model also has been recently proposed for gene duplication/gene deletion of msp-7, an immune target parasite surface antigen gene [24]. It may thus be argued that diversification of *Plasmodium* SERA multigene family was, likewise, driven by the birth-and-death evolution.

It is worth noting that gene duplication/gene deletion of serine-type Group IV SERA genes occurred primarily in *P. vivax* and related monkey malaria parasites. The reason for this lineage-specific evolutionary event is presently unknown. We consider that the gene duplication/gene deletion may be associated with an expansion of host range during the radiation of macaques that took place about 4–5 million years ago in Asia [25]. *P. vivax* related monkey parasites generally infect multiple hosts. For example, *P. knowlesi* and *P. cynomolgi* have the ability to infect a wide variety of macaques and humans [26]. The exception is *P. vivax*, which became a human parasite by host switch [18], [27]. It is likely that Asian macaque malaria parasites radiated along with radiation events of host monkey species [28]. Duplicated SERA genes might have gained a new pathway in the process of merozoite release or parasite egress from infected erythrocytes, and ancestral parasites that diversified duplicated SERA genes might have succeeded in expanding its host range during the radiation period. Alternatively, it can be assumed that duplicated SERA genes played an important role in immune evasion.

In *P. falciparum* and *P. berghei*, many SERA genes could be disrupted without any obvious phenotypic change and appear to be non-essential. If non-functional, the birth-and-death evolution model assumes that these genes could have been gradually deleted or become pseudogenes. But many *Plasmodium* SERA genes have sequence similarity with each other even in different species and, moreover, transcription and/or translation can be detected in the different family members. These observations suggest that some SERA gene family member may play a role in the parasite life cycle. Titers of anti-Pfa-SERA5 IgG antibodies show a strong negative correlation with malaria symptom [4]. Despite being an abundantly expressed antigen, however, epidemiological studies provide evidence for low sero-conversion in individuals residing in malaria endemic areas compared to another abundant malaria blood stage antigen, merozoite surface protein 1 [4]. This may reflect the parasites' use of other strategies to evade immune responses, and it is tempting to speculate that SERA genes may play a role in this. It should be mentioned that SERA genes have inter-species variable sequence regions, variable domains 1 and 2, in which amino acid sequence variations are extensive including various tandem repeats. Sequence divergence would have been favorable for parasites to evade host immunity. Some ancestral parasites that gained diversified multiple SERA genes may have succeeded in adapting to newly appeared macaque species, thus leaving a signature for ancestral gene duplications. Gene duplication of Group IV SERA genes was also observed in *P. malariae*, *P. ovale* and *P. falciparum/P. reichenowi*. Recent findings of infections of these parasites as well as new parasite species in Great Apes (chimpanzees, bonobos and gorillas) indicate that these parasites show a wider host range than previously thought [29]–[35]. Investigations of SERA genes in newly identified Great Ape malaria parasites may reveal variation in the number of Group IV SERA genes. It is thus assumed that Group IV SERA genes of these parasites have also undergone the -and-death evolution. Together, birth-and-death evolution of Group IV SERA genes is likely to be common in primate parasites which have multiple hosts.

The present evolutionary and experimental (expression) analyses, when coupled with previous gene disruption studies using *P. falciparum* and *P. berghei*, shed some light on the importance of SERA proteins to malaria parasites. The presence of Group I SERA gene in all 18 *Plasmodium* species examined suggests that the gene is maintained by *Plasmodium* for its function. Disruption of *P. berghei* Group I SERA (Pbe-SERA5) blocked parasite development at the mosquito stage by inhibiting egress of sporozoites from an oocyst [36]. It is thus likely that Group I SERA gene encodes a protein involved in sporozoite egress for other parasite species; and therefore, not surprising to see no expression of Group I SERA genes at the blood stage of *P. berghei* and three *P. vivax*-related monkey parasites. Group II SERA gene is present in all *Plasmodium* species, except *P. gallinaceum*. This group is also expressed, as evident from the four parasites used for expression analysis and, likewise, from studies on Group II SERA genes of *P. falciparum* [7] and *P. vivax* [37]. These suggest the importance of this gene at the blood stage, although disruption of *P. falciparum* Pfa-SERA7 and *P. berghei* Pbe-SERA4 (Group II SERA genes) were nonlethal [38, Arisue et al. unpublished]. All 17 mammalian *Plasmodium* species have Group III SERA gene; and disruption of Group III SERA genes (Pfa-SERA6 and Pbe-SERA3) has been unsuccessful in both *P. falciparum* and *P. berghei* [38], [39]. Pfa-SERA6 was suggested to be involved in schizont rupture and merozoite release from an infected erythrocyte [15]. Considerable expression of Pbe-SERA3 and Group III SERA genes in *P. vivax*-related monkey parasites suggests that Group III SERA gene is essential to all mammalian *Plasmodium* at the blood stage.

In contrast to Groups I to III, Group IV includes multiple SERA genes in all mammalian parasites. Contrary to clear orthologous relationship of Group I to III SERA genes, orthologous relationship in Group IV was not clearly seen across all mammalian parasites, although relationship was identified in mammalian parasite sub-groups (Figure 1). This suggests that the relative importance of individual SERA genes varies among parasite lineages. In the lineage of *P. falciparum* and *P. reichenowi*, most SERA genes of Group IV are orthologous. Pfa-SERA5 has been shown to be essential for parasite survival [40]. The importance of other Group IV SERA genes in this lineage cannot be ruled out because of substantial expression of these genes at the blood stage [7]. In the three rodent parasites, two Group IV SERA genes are orthologous. In *P. berghei*, simultaneous disruption of the two SERA genes does not affect parasite growth [39]; however, substantial expression of these two genes, as observed in this study, cannot rule out their importance in rodent parasites. In the lineage of *P. vivax* and related monkey parasites, Group IV SERA genes are further categorized into six clades. In Clade 5, all ten species have one SERA gene and in the *P. vivax* ortholog, Pvi-SERA8, expression has been detected [37]. All species but *P. fragile* have one to three SERA genes in Clade 3. Two *P. vivax* orthologs, Pvi-SERA4 and Pvi-SERA5 have been shown to be abundantly expressed [37]. Here we also observed high levels of transcription of Clade 3 SERA genes from three *P. vivax*-related monkey parasites (Pcy-SERA3 and 5, Pco-SERA3, and Pkn-SERA2). These suggest that SERA genes of Clade 3 and 5 likely play an important role at the blood stage. The substantial expression of SERA genes observed for other clades in the three monkey parasites as well as in *P. vivax* [37] suggests some role in each parasite species.

In conclusion, this study presents an overview of the evolution of the *Plasmodium* SERA gene family. The gene family was incipiently born in a common ancestor of the genus *Plasmodium.* Gene duplications during the parasite evolution generated two types of SERA genes, the cysteine-type SERA genes (Groups I to III) and the serine-type SERA genes (Group IV). Of note is that in mammalian malaria parasites, gene duplication occurred only in Group IV SERA genes, particularly frequent in primate parasites. Diversification of duplicated SERA genes supports the birth-and-death evolution in this gene family. It is intriguing to assume that duplications of SERA genes were associated with the parasite's expansion of host range. This study thus points to unique features of the *Plasmodium* SERA gene family and reinforces the importance of investigating other uncharacterized gene families of *Plasmodium* to further understand the evolutionary history and biology of this parasite.

## Materials and Methods

### DNA sequences

DNA sequences of the *Plasmodium* SERA gene family were determined for the following eleven parasite species: *P. malariae*, *P. ovale*, *P. gonderi*, *P. fragile*, *P. coatneyi*, *P. knowlesi*, *P. inui*, *P. fieldi*, *P. simiovale*, *P. cynomolgi*, and *P. hylobati* (Table 1). For PCR amplification, we initially targeted conserved protease domains of SERA genes, and conserved regions from the conserved hypothetical protein (HP) gene and the putative iron-sulfur assembly protein gene (hesB) (Figure S5). Sets of degenerate primers were designed for each of the conserved protease regions (Table S1). For *P. knowlesi*, primers were designed using the parasite genome sequences [41]. The PCR products were cloned into a plasmid vector and sequenced (see below). Additional specific primers were designed based from sequenced regions for further amplification and confirmation of HP, SERA genes and hesB (Figure S5). More than five clones for each fragment were sequenced on both strands by primer walking. Finally, obtained sequences were verified by direct sequencing, using sequencing primers designed to cover target regions in both directions. Using this sequence strategy, we successfully obtained 116 SERA gene sequences located between the HP gene and hesB from all but one parasite species, *P. gonderi*. Despite extensive trials of amplifications, including several long-PCR protocols, for some unknown reasons, we failed to amplify a SERA family gene member between the region of Pgo-SERA4 and Pgo-SERA5 (Figure S5). This unamplified sequence may be a very long intergenic region lacking an SERA gene. The 3′ terminus of Pgo-SERA4 and the 5′ terminus of Pgo-SERA5 were, however, determined by amplification using the uneven PCR method [42]. Phusion DNA polymerase (Finnzymes) was used for PCR using degenerate primers. Pfu (Promega), and KOD-FX (TOYOBO) or LA-Taq (Takara) were used for amplification of fragments shorter than 2 kb or those longer than 2 kb, respectively. The PCR condition was 2 min at 94°C, followed by 40 cycles at 94°C for 15 sec, x°C for 30 sec and 68°C for y min, with final elongation of 5 min at 68°C. Annealing temperature, x, was set 2-3°C below the *Tm* of primers, which was calculated using Genetyx ver. 9 (GENETYX Co.). The extension time, y (min), was set at 1 min per 1 kb. Amplified fragments were cloned into pCR Blunt II TOPO vector or pCR XL TOPO vector (Invitrogen). Fragments amplified using KOD-FX were dA-tailed with A-attachment mix (TOYOBO) before TA-cloning. DNA sequencing was conducted on a 3130 Genetyx Analyzer (Applied Biosystems, Foster City, CA). SERA gene sequences reported in this study were deposited in DDBJ, with accession numbers AB576870-AB576881 (Table 1).

### Sequence alignment

Analyses was done for 116 SERA genes obtained, together with 49 SERA sequences retrieved in public database from seven *Plasmodium* species: *P. falciparum*, *P. vivax*, *P. reichenowi*, *P. yoelii*, *P. berghei*, *P. chabaudi* and *P. gallinaceum* (Table S2). Open reading frame of each SERA gene was predicted by comparison to *P. falciparum*-SERA genes. A total of 134 predicted SERA amino acid sequences from 18 *Plasmodium* species were aligned using CLUSTAL W program under default options [43] with manual corrections. The sequence alignment obtained here and amino acid sites used in the present analyses are shown in Figure S2. Amino acid sequence similarity among orthologous SERA genes was calculated by the Nei and Gojobori method implemented in MEGA version 4 [44] using pairwise deletion option and overall average of p-distance is presented.

### Phylogenetic analyses

Maximum likelihood (ML) trees were constructed using PROML programs in PHYLIP version 3.69 [45]. Jones-Taylor-Thornton (JTT) amino acid substitution model [46] was used. To take the evolutionary rate heterogeneity across sites into consideration, the R (Hidden Markov Model rates) option was set for discrete Γ distribution with 8 categories approximating the site-rate distribution. CODEML programs in PAML 4.4 [47], [48] were used for estimating the Γ shape parameter, α values. For bootstrap analyses, SEQBOOT program in PHYLIP was applied to generate resampled datasets. Five hundred replicates were used for analyses. Bootstrap proportion values were calculated for internal branches of each tree using the CONSENSE program in PHYLIP.

### Transcription analyses

Parasitized erythrocytes were obtained from mice infected with *P. berghei* (ANKA) and from Japanese macaques, *Macaca fuscata*, infected with *P. coatneyi* (CDC), *P. knowlesi* (H) or *P. cynomolgi* (B). Blood was taken several times with 4 to 12 hour intervals and parasites at the late trophozoite stage were selected for transcription analyses.

Studies using mice were approved by Animal Care and Use Committee of Gunma University and conducted in compliance with guidelines (Permit ID: 10-007). The experimental monkeys were second-generation offspring bred in captivity. The investigators adhered to the Guidelines for the Use of Experimental Animals authorized by the Japanese Association for Laboratory Animal Science. The protocol was approved by the Committee on Ethics of Animal Experiments at Dokkyo University School of Medicine (Permit Number: 0536). All procedures were performed under anesthesia by a combination of ketamine hydrochloride (10 mg/kg, i.m.) and xylaxine (0.5 mg/kg, i.m.), and all efforts were made to minimize suffering. The details of animal welfare/care and steps taken to ameliorate suffering were in accordance with the recommendations of the Weatherall report, “The use of non-human primates in research”.

Total RNA was isolated by RNeasy Mini kit (QIAGEN) according to the manufacture's protocol. First strand cDNA was synthesized with Superscript III First-strand System for RT-PCR (Invitrogen) using 20 µg of total RNA. To confirm SERA gene transcriptions, PCR amplifications were performed using synthesized cDNA and specific primers for each SERA gene. Since no PCR product can be obtained for *P. cynomolgi*-SERA1 gene and *P. coatneyi*-SERA1 gene, these were excluded from further analysis.

Real time quantitative PCR was performed using ABI PRISM 7900 (Applied Biosystems), and results were analyzed with the SDS software version 2.2 (Applied Biosystems). A 15 ul mixture was formulated with 7.5 ul of TaqMan Gene Expression Master Mix (Applied Biosystems), an appropriate volume of first strand cDNA, 3 pmol of forward and reverse primers, and 0.5 pmol TaqMan probe. Sequences of primers and probes are shown in Table S3. The PCR condition was 2 min at 50°C and 10 min at 95°C, followed by 40 cycles at 95°C for 15 sec and 60°C for 1 min. Relative mRNA copy number of SERA genes within each species was compared using the internal control β-tubulin. Standard curves were generated using serially diluted cDNA template for each β-tubulin and SERA gene. After confirming the reproducible linearity of the curve where R° value is >0.98, threshold cycles (Ct) of each gene was applied to the curve and the relative expression amount of each SERA gene was calculated against β-tubulin in each run. Experiments were conducted three times with triplicate samples.

## Supporting Information

Figure S1 **The SERA gene family drawn to scale in17** ***Plasmodium*** **species.** The 5′-end of each gene map is set at the start codon position of the conserved hypothetical protein gene, whose transcriptional direction is opposite to that of SERA genes. SERA genes of Groups I to III (cysteine-type) and those of Group IV (serine-type) are shown in green and blue, respectively. TSERA denotes truncated SERA genes shown in yellow. SERA gene fragments (putative pseudogenes) are in gray. The region that was not successfully sequenced in *P. gonderi* is indicated by a double slash [*P. reichenowi* SERA genes in this region are not shown due to the lack of complete sequences].
(EPS)

Figure S2 **Amino acid sequence alignments of 134** ***Plasmodium*** **SERA genes.** Amino acid sites used for constructing phylogenetic trees for Figure 2 (570 amino acid sites) and Figure S3 (392 amino acid sites) are marked (#). The catalytic serine and cysteine residues are shaded in green and pink, respectively. Other active site residues are shaded in yellow. Tandem repeats of serine residues are highlighted in red. Putative PfSUB1 recognition motives are shaded in blue.
(DOC)

Figure S3 **The maximun likelihood phylogenetic tree of the** ***Plasmodium*** **SERA gene family.** This unrooted tree was constructed from 134 SERA genes using 392 amino acid sites under the JTT and Γ (8 catogories) model (α = 0.96) with 500 heuristic replicates. Bootstrap values >50% are shown along nodes. Groups I-III and Group IV are cysteine-type and serine-type protease SERA genes, respectively.
(EPS)

Figure S4 **Amino acid sequence alignments of Group IV Clade 6 SERA genes of** ***P. vivax*** **and** ***P. vivax*** **-related monkey malaria parasite species.** The catalytic serine residue and other active site residues are shaded in green and yellow, respectively. Asterisks denote stop codons. Putative PfSUB1 recognition motifs are shaded in blue. The long deletion in truncated SERA genes of *P. coatneyi*, *P. knowlesi* and *P. fragile* are highlighted in black.
(DOC)

Figure S5 **Sequencing strategy for the SERA gene family in 11** ***Plasmodium*** **species.** Thick bars indicate conserved protease domain of SERA genes targeted for PCR amplification and sequencing. Primer names are given in either ends of bars, and their sequences are listed in Table S1. The region that was not successfully sequenced in *P. gonderi* is shown by a double slash. SERA genes of Group I to III (cysteine-type SERA gene) and those of Group IV (serine-type SERA gene) are shown in green and blue, respectively. TSERA denotes truncated SERA gene shown in yellow. SERA gene fragments are shown in gray. In this study, the H strain of *P. knowlesi* (ATCC 30158) is different from the H strain used for the genome sequencing [41] for unknown reasons. The number of SERA genes and the organization of the SERA gene family, however, are identical between the two, and the sequence identity of SERA gene family region is 95.5%.
(EPS)

Table S1 **PCR Primers used.**
(PDF)

Table S2 **SERA gene accession numbers in PlasmoDB database.**
(PDF)

Table S3 **Primers and probes used for real time PCR.**
(PDF)
