# Supplementary material for: Clues to Evolution of the SERA Multigene Family in 18 Plasmodium Species
Source: PLoS One. 2011 Mar 15;6(3):e17775. doi: 10.1371/journal.pone.0017775 (PMC3058004; doi:10.1371/journal.pone.0017775)
Supplement: Figure S2 — Amino acid sequence alignments of 134 Plasmodium SERA genes. Amino acid sites used for constructing phylogenetic trees for Figure 2 (570 amino acid sites) and Figure S3 (392 amino acid sites) are marked (#). The catalytic serine and cysteine residues are shaded in green and pink, respectively. Other active site residues are shaded in yellow. Tandem repeats of serine residues are highlighted in red. Putative PfSUB1 recognition motives are shaded in blue. (DOC) [file pone.0017775.s002.doc]

Figure S2. Amino acid sequence alignments of 134 Plasmodium SERA genes.

Amino acid sites used for constructing phylogenetic trees for Figure 2 (570 amino acid sites) and Figure S3 (392 amino acid sites) are marked (#). The catalytic serine and cysteine residues are shaded in green and pink, respectively. Other active site residues are shaded in yellow. Tandem repeats of serine residues are highlighted in red. Putative PfSUB1 recognition motives are shaded in blue.

392 :--------10--------20--------30--------40--------50--------60--------70--------80--------90-------100

392 :----------------------------------------------------------------------------------------------------

570 :----------------------------------------------------------------------------------------------------

vi1 :MNPRVCFAWAMCALIGAEVGAKVAEEGLRTGLAPPQNAGSNHTGAKRLPLDRGSDRC-VSPQFSSHIEADAP-PRSADDAAGR--------GQPPQQVEH

cy1 :MNHRVCFTWAICALLGTDVGAKLVVDGLWTGSVSLSNASSKRTDAKRLPLYRGSDRG-VSSEHSSDIETGSAVPHPAPNVAPRNADDAAGGGQPPQEVDH

fi1 :MNPRVCFTWAICALLGTDVGAKLAEEGLWTGSASPPNASSNYTDAKRLPLDRGNDRD-VPPEYSSDIEADGAVPHPAPDVPTRNADDAVGRCQLPHQVEY

so1 :MNPRVCFTWAICALLGTDVGAKLAEEGLWTGSASPPNERSNYTDAKKSPLDRGSDRG-VSPEYSSDIEADGAVPHPTPDVPPRNEDDIAGRGQPPQQVVH

in1 :MNHRVCFTWAICALLGSDVGAKLAAEGPWTGSASPSNVSSNYTDAKKLPSDRGSDRG-ISPEYSSDVEADGAVQHHTPDVSPRNADDAAGRGQPPQQVEH

hy1 :MINRVYFTWAICALLGTDVGAKLAAEGPWTGSGSPPNVSSNDRDAKKLPLNRGSDRG-VSHEYSSGIEADGEVPHPASDVPPGNTDDAAGRGHPPEQVDH

co1 :MNSRVCFTWAICTLLGTNVGAKLAPGGLWTGTSSSQNASSNHADAKKLPLGGQSDRS-VSPEYSSNIEADG--------------------EEPPQQVEH

kn1 :MNPRISFTWAICALFGTYVAAQLASGGLWNGSSFPTSAYSNHTDENKLSLDQENGRGDASPEYSSDREVDGTIAHTPLSVPPPNADNPAGQGQSPQQVDQ

vi2 :MKLALPFLFILSVALLDNAIKCDEE--VTIPDPPQSPDENPGG----KDDPPGDSDPLPGEGAGAVEPAGGETDSGVEEGPAEQAVDQPLTQSTDQPADQ

cy2 :MKLTLPFLFILSVTLLDNVIKCDEE--VTIPDPPQSQDENPES----KDNPPGNSDPPPHKGAGAVEQAVDKSADKPVDKPADKPADKPADKPADKPADK

fi2 :MKLALPFFFTLSVALLDNVIKCDEE--VTIPDPPKLQDENHES----KDNPPGNSDSPPHKGAGAVEPTQEENESGGEHGAVDQPADQTTNQTTNQTTNQ

so2 :MKLALPFFFILSVALLDNVIKCDEE--VTITDPPQSQDEKPES----KDNPPGNSDPPPHKGVGAVEPTVDKNESGGELGAVDQPTNQTTNLTTNLTTDQ

in2 :MQLSLPFLFIASAALLDNVIKCDEE--VTIPDPPQPPDENPES----KDDPPGNWDPSPHKGDGAVEPSVDENESGGEHGAVDQQTDQPTDQRTDQPTDQ

hy2 :MKLALSFLFIASAALLDNVIKCDEE--VTIPNPPQSPDEYNES----KDDPPGNGDPPPDKGDDAVEPPVDENDSGGEQGAVDQAVDQPKDQPTEQPTEQ

co2 :MKLALPFLFILSAALLDDVIKCDED--VNIPDPPESQNENPGR----KDNPPSNSNPSPHEDSGTVEPTVDGNESGGEHKTVGEAIDKPADQPADQPAEQ

go1 :MKLVISLLFILGIKTFENVIKCEEDKNIIFNTPKEHEENNLNIDNPLGDSGSPPSDDGTHKSGDHVIDGVELESEDRKTPDTCHTVNEVSGGKEEKPVIE

go2 :MKSHLLFLFILGVVFSKYEILGEGTEQRVSSGETASSPSADAGGGGSGGGQAGNSSGAPGSVQGGVQNGASSATQGDRVTTSMTTSPASSQTVVSPPSGN

vi3 :MKSRICALLLIGLVFVNEGARCAPPAGEGTAVTVQAGQSPGSAGGGDGGGGGGGSSAGSSHSNSGGGQGSDAAAQRPAAPQPPPTPPAEGSSSPPAGNQN

vi4 :MRLPIFHLLALYWVCTEGVVKCEQEGGGSGTSDSSGGDPQTSPKGGEAASSAHGSQGSKESVTPGGSHAQQEAAAPEKTLVPQESPAKEGGAPPSVPENQ

vi5 :MKSRLCALLILYMLLNGHSVKCTAAVGQGQGTGVSTDQGVSSQHTANSAGQGIGSSTGSTGVPQSRPQSGGGDTGTQGGQQDRASGTSATHVPVVPQNGH

cy3 :MKSRICALLLIGLALANGGARCAQTGNGGPSASSNPGGPSPDNAAGQSPSTGSGGDVSSTGSSSSNSGAGGGSDPAAEASRADGSPSPQENNLNIPSPVP

cy4 :MRLPIFFLLTLYWLCTEGVVKCESQEGSTSHTGDSTDGDSQKNSEGGDAPSPPNSGGEEGESQDRSPAQEEPQDPQELPPQPESPPQPESPPQQDGVTHS

cy5 :MKSRWCALLILYMLLNRHSVKCTPGEISESSSGLGSGPDGVQSSGPTSGVSDDSSKSPQQNAEQTIPGNGSSTGTTVSSPSGQPSGGGGTDLQAGSGSAG

fi3 :MKFRISALLIIGLTFANGGARCTPGGSEGHSAGTPGGSSQDAGSREVSSASLSQGTSSGPVNQANPSQGTGIVGQVSSADSSSGSSGGVGSGNTTLQAPE

so3 :MKFRICALLIIGLTFVNGGARCTASGGEGPSGSTTGVSSTGTGNGGVSQAGSTPVTANGGQANSVDSSSGSSGGGGGGDTTVQTHASGGVGSPSPQADNQ

in3 :MKSRLCALLILYMLLNRHSVECATTPSSGTGSGVSNTPSGSSQQNPSEGRSADGSSTGTNNTSLHGPTNVRGETGTQTGSPGTEETGTSREGGNQGNVVP

hy3 :MRTRLCGLLILYMLLNRHSVKCATNSGSGEGSEVSNVPSGSLDQDPSKSSAEDGTSSGSATTSPSQPPSGGGDTPTQVGTSHSTGQTNPAPTHSSLDQTH

co3 :MKARICALLLIGLLFANGGARCADAGSGGTPGDNPAGPPQSTDNGGMNSASSSGGHSSAGGSSAPAAQSPEAPNAGGSPPPQATSANIPAVSQAPSVSNT

kn2 :MKLRICALLLIGLAFASGGARCAPGESEGQSPNDSSGPSGGSSGGQSEGDSTGPSQNAPASPSHDGAGSPPGGNPTSSPQGPGNGEVSSVNPSSSTPGTE

go3 :MKLRTYVLLITGLVFTNGVVRCNATTGTPDGRSVQASDQKGAEGQDSGGESTPPSTPTREGSEQPSPQQSAEHQASEQVSNAVSPSQETPESSSQGSANS

vi6 :MRSRFSVLLLTYAVLNGGILIKAEQSNDSPPPAGPPPKDDTSLPSAPPPNPVTPGVEVQQGPSHSPTLPGGTHDTAVQGPQQDPQGPTHQVPSEESSDQG

cy6 :MRSCMSVLLLTYAVLNGGILLKAEENDNKETSPVSPSQEDTSSPTVTSPNPENPSAQETQDLSSNLTVQNGSLDAAAPGPPSAPQGATDPVVSVESASQG

fi4 :MRSCMSVLLLTYAVLNGGILLKAEGGDDNALSLVPPSQDGPLPPNGTPPNHVTSSPDESQDPSSNPKVQNGSQDSPVPVPPRGPQEVTPPDASVERSDQQ

so4 :MRSCMSVLLLTYAMLNGGILLKAETGEDDTLSLGSQSQDFTLPSGDTPPNTVSTSNEEKQDPSLSPTVGDESHNADLPVPPPVPQEVTPPVTSVERSDED

fr1 :MRSCVFILLLTYAALNGRMLLNAETSDQNTLSPESPSHGVTSPTPVTPNSETPQNPSPSASGQNGSHNTAVPGTPQGPQGATHTVTTIPSAHQGAHAGPQ

go4 :MKSRLYALILICVIFNETVIIKTETTDNPASSDSSTPGPASPPGPPSPSGPPSHSGSPPSSDPSTQTESLPQSDIPNSAENQVGVSHVSSNDLKTPSGNA

vi7 :MRSRISFFLVTCAIFGRFAVDCVEGTDHPPDPKQTGPSPTDPNQADQNPAGPSPAGPDEAVPNPPVPNQPVRVPPPPTPPSEHNVSPAGQTPGGTPQVET

vi8 :MKSSVLLLLALGATYGNNVAMCTATPPSGGPDASLPNPAQPNSLPAVETLSQQGGGAPAASSNALTAGGNVPPGSQVNSGEQGGAT--------------

cy7 :MKSSFLLLLALGAAYGSNVAMCTTVPESSSGGSGGSQSTSSASGTVTENGGSSGAGLQSASSGNEQGVTQPTNPSPPVSQAPNSAESNPSQPVQTPDQHA

fi5 :MKSSFLLLLALGAAYGNNVAMCTDVPTPPSGGSGDSSSSSSGSVAGAGNPGSSGSGSQTSSHGSGQVGDESLNPQQPVSGAPISAGPDVSLSSSGGPGTE

so5 :MKSSFLLLLALGTAYGNNVVICTTGQTPSSGESGVASSSSSGPGTGSDNQQESGQGPQPESAGNGQNVEESTNHQQPVPGAPNSAGSNLSQGGATIKSNG

in4 :MKSSLFLLLALGAVYGNNEAMCTTGQTLSSEVSGHSSSSSSVSASGGGNPGSSESGTQNSPGSDPPQNGQTSNQHGDGTHNTHPTVPTSQESVSGSSSGN

hy4 :MRSSLFLLLALGAVYGNNVAMCTESPSLPTEGSGGSSSDSSVPGTGTENQGTSGSGAQNVPGPNPSQTVQTESQNGDGSPSTNITAPTPKESVSDSSSGS

co4 :MKYSFLLLLALCAAYGNHVAICTTTETSSDGGSNVSQPGPSGSGTGAENPGSPQAGSHSVSSESEQGVGESTNLPSAVSEAQSSAEPNSPQEGQAANQDG

kn3 :MKSSFLLLLALCATYGNNLAICTTEGTSQSEVSSDSQHSLSSSETETGSHGAAGAEAQSVSPADGQGVVHSKNQSTESDAENPTGPNPPQEDETSNEDGN

fr2 :MKYSFLVFLALCAAYGNNVAVFTTTQTSSGDSPVTQSTSTDQVTETGHVTSSGEGSHSGSSESGQKGSQADNLQSHGSNAQSYEPT---------NPQGG

go5 :MKYSILFLIALCATYSNTLVSSTTTTTVTTGTTGATHGATTSQAATAEAQTPNTSTVTASPGTSSSPSPTEQAPSAGSVQA-------------------

vi9 :MKARLSLILILCVVCRDCAVRCTGTTEAQGAVEGAKGPKPGAEEAGANVGEAGTGGPGGPGADGGTEAGARAEEGEGAGTEAEPEAEPEAEAEPARGPEP

cy8 :MKARLSLILILCVVCRDCAVRCTGTNDNPGAETGKQAEERTEAGGGPGGVEGTGAGAQGVVGAAGGGVAGTGAVPGQGGAVPGPGSGEAVSLSQDAGDTP

fi6 :MKARLSLILILCVVCRDCAVRCTGNNGNPVAGTEPAPGPVTAPGGSGGQGSEQVAGGQARAEAGQGTEVGQRTGAQVQAGTGAGTGAGPGAG--------

so6 :MKARLSLILILCVLCRDCAVRCTGSNESPEVKPGPGGVAGEGTGEQAQEQEQAQVQVQAQAQAQAQVRSGTEAGGVPGVGGVPGVGGVPGVSGVGEAGAG

go6 :MKTRISLFLILCAVFSNSAIKCEGGTAGAGTTTTGQTSGISTENIQSQGGQAPQGTGEDTSQVQSPGIPSGATAPSPPINSDTPRAQDQIVTSPSP----

ma1 :MKCSISFFLIIYIILSRDIFRCKGEDKAVEPTQGQSGAQGSSSDTEGRANSPSVDGSHNGPLSVNQQTDPKVTAPTVPSTSNTQTGESNFRQEVASASAA

ma2 :MKSCISLLFIICVVYSNSIMPCHGGTTMEQPPNVTSPSNSIAQDVSQNNGSSSVQGSSTNTNPNGAGDPSVVPGAGVGGSGPTGANGVVGENHNGSSDGT

ma3 :MMYPISFLLIIYILISNNIIKCYCSTKNAPVTSISVFLPKVDGTRISSSEGLADQSQATATAVSHVNTKEGKGKKILKRGESLQTGQQNSSIVGAPNGPA

ma4 :MKTRITLFLVLFNVCFYILYFYDKFYADIVLGKIITECTEKPPEAPVLPKDGNTASSGTGSTSSQTPLPSQPPPTGSLPSASPGVPLTPEPAKQEASDRE

ma5 :MKSLIPLLLITYTVVSKNVVICANDTVTSVTPPKEVLNTSNSLKTTLPAAQPDAKNGNEDTGRVDTNVTTIMTNPENS----------------------

ma6 :MKYGILYIFMICISFGSNTIKCTTVSVSDNRGNEASEQPLQPAQPGPQTHEPSNSQVQNSSNPNISDLTVTTPPVAQNLSHETPKNGSSQSPPQNGPLLS

ma7 :MRSCISLLLTISPGSTGNSSTTISSSNSERSASPSESPSSGGQATSNGGSNLVGSSTAVPSNPQPSQDVSTSIANK------------------------

ov1 :MKSHVSIIVTLYGIFSIHVTQCTGGGAHWPPHNMHTSNPGNGNKTGDSAIPPSSGNLSTSASGSTG----------------------------------

ov2 :MKSLIPFFLIIYTILSRGAVKCASASGESGGGGSGGGGSAVNTGSSGTGSTGSQGTGNNQPQGVVAGVTGTQGNATVTTGHA------------------

ov3 :MKCCIPLFLVLCASFGKDTIKLASGENVPKEVQDVSVPADPGNHKSEDTGSSVPTSISSKPVVDAQPVSQVTEAASAGSGTHKISTEGKSPITGSTESAS

ov4 :MKTHISFFLIIYTVLSKHIVKCENNNINQSNGGLGSASLSQPVQSAQSQGTGHNGGGEESNTVSNQNSQGGGGGQGGLSPGLVSGTTPKV----------

yo1 :MRR-LPILFILYALLIRNSKEGGLGVNGEGDALTSLPTSGGTEGDTSGKKADGSLSPGPGPTEPGGPAPVGPLPGSPQVEEPKLSTPSVEGSQLESSSLT

be1 :MRR-LSILFILYALLIRNYGLGVNGEGDTSSDVPTSDGTGGDTSGKENVELPSPVPGPEPGTAGPAGPQVAEPKVDTQSVEGSQLATSPPTGNGTKSVEN

ch1 :MRR-LSILFILYALLIRNSIETDNGVPSAGETPPSTPPSGGTGDDSTGKEKAESPSPVPGPASDDSLSKESVNTGGSELKTPSSSDGGPKATEEDRKEAG

yo2 :MKTYIPLIFLLYAMLGNDIINCRPVPQHFPYPSTLTGGNNGTTPGGAQKAEKGTTDKNASELESEKDKNSQKSTDPSAPSSSNIATLPGSPDSAKQGSGS

be2 :MKTYIPLIFLLYAMLGNDIINCRPVPEPFGSEVTTSGTTGGSQKETTSTTGKNTSELESKKDNPSEKSQDPSTPSSNTATLPGSPGTSGTPNPSDSSDSS

ch2 :MKKCIPLIFLLYAMLGNDIINCRPVPEPMFSTHEYDKHINGAGKGTGGSNGVTPETGKGTTNEKISQPESEKDKAPESSPDSSTLPSPGSGPSPSSPESS

fa1 :MKFFIPYVYIICVIFIINVIRTRGEEDDDNNNISGKSILGTSHNNISNIDLSSIPNLDSNIHASFSSDTKEWSPNNLTSKKKKKKKEIRPKDIMSNSDSS

re1 :MKFFIPYVYIICVIFIINVIRTSGGEDDDNNNISGKSILGTRHNNISNIDLSSIPNLDSNIYVSLSXXXXXXXXXXXXXXXXXXXXXXXXXXXXXXXXXX

fa2 :MKFHISFFLILYIVFFKNTIKSETTTDESATGSLSSDGSRVTTQARIEKPKQQPTLPTLAQETQPQQQQQQKEVGSGIGAEQKVESARPGAEVSQSDVER

re2 :MKFHISFFLILYIVFFKNTIKSETTTDESATGSLSSDGSSVTTQERIEKPKQQPALPTSAQETQSQQQQQQKEVGSGIGAEQKVESARPGVEESQSDVER

fa3 :MKFSISLFLILCVLFCKNDIKCTTVDESTKEGSQNPKNSSSTTPASGSQKGSSSESPGSSVEKQSQESNKESTNGGNVVSQGTPANTFGQNSNNPSDSPQ

fa4 :MKIHIFLIATIYVLFSEKLIKWTTASTTQGGDTDTHPGTPPGEGSDVSQGAGQDASQGAGQDANPD--PTLPKPPSPPADDTKDTGSQGDADSSSSKIEI

re4 :MKIHIFLIATIYVLFSEKLIKCTTASTNQGGDKVTQPSTLPGTNQDTQSSTLPGPTPTPGASSGPGTGPTSPKSPSPPADDTKNSGSADNADSSSSKIEI

fa5 :MKSYISLFFILCVIFNKNVIKCTGESQTGNTGGGQAGNTGGDQAGSTGGSPQGSTGASPQGSTGASPQGSTGASQPGSSEPSNPVSSGHSVSTVSVSQTS

re5 :MKSYISLFFILCVIFNKNVIKCTGGSTSTSPEQSNPGSTRGSTPSSSVESNSVSTGGSTSSSSVQSNSVSTGENSPSSSVESNSGSTGESSPPSSVESNS

fa9 :MKVSFTLFFIIYIILNCDVLKCEVESSDSTSFLNLISGQSSSNSDDTVSSSDSRGVVASVSGTVDSDVSRTDSTGNVNSKNSELSETRQNSVEQNNNSNS

re9 :MKVSFTLFFIIYIILNCDVLKCEVESSDSTSFLNSISGQSLSNSDSTVSSSDSRGVGASVSGTVDSDVSRIDSTGNVNVKNPELSETRQNSLEQNNNSNS

ga1 :MMSPFFFFLIIYVIFSKNIIECTNDEVPQTNDLGEKEAAKAELQNEPTPANGQTLEQEVQSATQREVTAEPEGKSENGVTTSAVASPAAPQDAPLDSREA

vi10 :MRIRPAALILILALLTSTNVTICEAHKIDDVTQGGNKGTSGGGSNSAPPSKNKPPNDGSNNDEISFPTKTISFSMPPTLEGGGSKNDSAGSADQVGGTTG

cy9 :MIKRPVALILIIAILTSTNLTICETNKVDNVTQEGNKGTSGGSSNLSPPSNNNPPSDGSNNDETAFPTKTISFSMPPNLEGGGSSQNESAGSTDQVGTTT

fi7 :MFKRPVALILIIAFLTSTNVTICEANKVDNITPGGNNGTSGGSSNSSPPSNNSPPSDGSNNHETAFPTKTISFSMPPNLEGGGSKNESVSSTDQVGATTG

so7 :MIKRPLALIFIIAALTSTNVTICETNKVDNVTQGGNNGTSEGGSNSSPPSKNNPPSDGSNNNETAFPTKTISFSMPPNLEGGGSKNESVSSTDQVGETTG

in5 :MIKLPVALLLIIALLTSTNVTICETNKVYNVTQEGNIGTSGGRNNSSPPSEDKPPSDGSNNDETAFPTKTISFSIPPNLEGKGSKNESAGSTDQVGATTA

hy5 :MKKLPVALLIIIAALTSTNVTICETNKVDNVTQDGNMGTSGRSNDSSPPSEDNPPTDGSNNDETAFPTKTISFSMPPNLEGEGSKNESAGSTDQVEATTA

co5 :MIKRPVALILLIAFLTSTNVTICETNKVDNVTQGGNKGTSGGKSNGSPPSKNSPPSDGSKKNETPFPTKTISFSMPPNLDGGESKNENASSTEKKGAPTD

kn4 :MIKRPVALILILAFLTSANVTICETNKVDNVTQGGNGGISGGDSNSSPPSKNNPPNDGSKNNETAFPTKTISFNMPSNLEGAGSKNDSAKSTDEAGARTD

fr3 :MIKRSLALILIIAFLTNINVTICGTNNVDNVTQGSDKGTSGGYSDSSPPSKNNSPSDGSKNDVTDFPTTTISFYMPLNLEGGGSKNESASSSHKVEEATD

go7 :MIIRPVWIFFVLVFLIIKNVVICETNKKNDASEDSNKGANETNNNSSSSQNNNPSDKSNNEDDD-FPTKTISFNMPTNLENGGSTDDNLENKDKSKTTRN

ma8 :MIRLVSLFLIIWVIFSKNIIKCGKDKLNEPPQDSTGVGEVIKPVSSQNKENIPNDTSKKRDTPTKTISFTIPGNLENDNSNDENKNGYGVVGTSSSHAIV

ov5 :MGRPVSFIFVIMGVVFSKHVIIRAEDKPNEVPFYKPGEGNVDPPPASSENKKNTPSDTSSNENFPTKTISFTMPDTPGSEHPKEEPKEESKKEPKEESKK

yo3 :MARLSSIVFIICLLLCNNAISNEVIESPPSEGTLAGGGGGGGSGSGADGDTGGGTDSQDGKGDGKGEGKGEGKGETVGDKGKNNEGSQTDQELQTNPGSD

be3 :MARLSSIVFIICLLLCNNAISDEVIESPSSGGTLSGGGSGTDTVTGTQDGKGKSEGKGNEGGQTDQKGKENPENGQNSDSTGDSSLGSTGSNGSQPAPTT

ch3 :MARLSSIVFIICLLLCNNVISDDVVDLPSSGGDVSDGGVTVGDGGSGGGGSDGETLPGGSGDIKTGDGQTGKGEQGSSNTDQVPGSTDSQDSDQPPKESA

fa6 :MICPIFFLYIINVLFTQYFIKCEGNKVTVISHNNGHNDNLDVNKNGVISQENVFDTSESLNLPSNKKVGSDDLNTTTISFTVPDNLENEVKVVSSSESGK

re6 :MICPIFFLFIINVLFTQYFISCEGNKVTVISQNNGHNDNLDVDKNGVISQENVFDSSESLNLPSNKKVGSDDLNTTTISFTVPDNLENEVKVVSSSESGN

vi11 :MVSRLCFLLTICVALGTHVTRCAGDGPPETPVGEKPHTASPPQGGGPDAGAGGPSLPVAGPASQDADPPSSSSANGIASPPADGETALPPRSAETTDAAD

cy10 :MVSRLCFLLTICVALGTHVTHCAGDGPPESNVGDQLHTGSSPQGGVPDAGAGNPSLSVPGQTSQDTEPSSSSSENGIGSSSSNEETASPPTSADAKSNGI

fi8 :MVSRLCFLLTICVALGTHVTRCAGDGPPEPSVGEQLHTVSPPQGGDPDAGVGSPGAGVGGPGAGVGGPGAGVGGPDAVVGGPDAGVGGPDAGVGGPDA--

so8 :MVSRLCFLLAICVALGMHITRCEGDVPPEPRVGEQ-HTVSLSQGGGPDAGVGGQKADAGDLDAGSGDSEAGAGSPEAGVAGPEAGARVPNADVGGPDAAS

in6 :MVSRLCFLLTIYVALVTHITRCAGDRSPETGVGQQLHRRSPPQGGGPETSAGGPSMSVPGKVSQDANPSSTSSANGIGSSSSVGGTALPPRSTDVESNDI

hy6 :MVSRLCFLLTICVALVTHVTRCAGDGPPDTVVGQELHRGSPPQRVGPDAGAADPSVSVPGHASQDADPSSTSSSNGIGSFSSVGGTALPPRSEDVESNGI

co6 :MVSRLCFLLIICVALGTHVTRCTGDGSAVPGGGDKPHTVNPSQGGSPD-------------------AKSNDIESESEG------AASMEVADASKED--

kn5 :MVSRFSVLLIICVTLGTYVTRCGGDGPAVTDGGEKFHTVSPSQGGSPDSGAGDLSLSVPGQASQDANSKSNAMETASEG------AVSMQVADPSEED--

fr4 :MVSRICFLLTLCVALGTHVTRCAGDGPAKPGVGEKLHIVNPSQDADSPSSSSANGISSSPPVGETPLSHTSADAKSNAIESESAHAASMEAVDTSKQD--

go8 :MIRRLCFLLIICVIFGKYVARCEGDTPVESGNGDSGVATPGAGVSQGGASGSSDKDTDKPQSPESSSSSSSSSSSSPSSEDVAKEQDPPAAVPPPQAESP

ma9 :MIFRSCFLLIICVIYSKNITNCVAETTVNSDTGGPHGSGIGEQGLSSSSSENVTGESHSPPGTSVGTSSDNDQSSSSSVTAGNTGDVGQTSEASSKVPEV

ov6 :MVYLFFLLLLICFVRSKKVTDSLSDTPISFDGIRVKGIEGTKHPNDDLFHNHSEETLKNIVTSSSGGKGQQDLSKILSLGEQLNISSSVNSYNIRNDTIL

yo4 :MNPRRYLLLVSXAIFTINVHEIKTQSSNPPNDIINDAYKN-------------GTYKNELNESSGLSTNHIDETDVKKESDNNTKNDSLNNEISDTE---

be4 :MKPRIYLLLVSCAIFTINIHEINTQTSNPPNDIIDDVYTNVQNDNDKSNQDLDDEFQNGLNTSSGLSTN-IGQTNVEKESDNNEKNESLNNETSYMK---

ch4 :MNSRLYLLLVSCAIFTINVHEIKTQPPNTPNDIINKGDPNVQNNNDISNKNSHDGLENGISTSSDLSEN---------QSNNNTNSESSNSETPDVK---

fa7 :MVYRLFIILVLYVICCTNVIVGQEKPPPDSTVGANPGDERESSGRVNNPASGEQGTTNSPTEQPDQTRDRSSSVPQGSPREPVSPENPNPVTQIPGNGGA

re7 :MIYRLFIILVLYVICCTNVIVGQEKPAPDSTAGVNPGDERESSGTVNSSASGEHGTTNLPTAQPDQTKDRSSSVPQGSPREPVSPENPNSVAQTPGNGGA

vi12 :----------------------------------------------------------------------------------------------------

cy11 :----------------------------------------------------------------------------------------------------

fi9 :----------------------------------------------------------------------------------------------------

so9 :----------------------------------------------------------------------------------------------------

in7 :----------------------------------------------------------------------------------------------------

hy7 :----------------------------------------------------------------------------------------------------

co8 :----------------------------------------------------------------------------------------------------

kn6 :----------------------------------------------------------------------------------------------------

fr5 :----------------------------------------------------------------------------------------------------

go9 :----------------------------------------------------------------------------------------------------

ma10 :----------------------------------------------------------------------------------------------------

ov7 :----------------------------------------------------------------------------------------------------

yo5 :----------------------------------------------------------------------------------------------------

be5 :----------------------------------------------------------------------------------------------------

ch5 :----------------------------------------------------------------------------------------------------

fa8 :----------------------------------------------------------------------------------------------------

re8 :----------------------------------------------------------------------------------------------------

ga2 :----------------------------------------------------------------------------------------------------

ga3 :----------------------------------------------------------------------------------------------------

392 :-------110-------120-------130-------140-------150-------160-------170-------180-------190-------200

392 :----------------------------------------------------------------------------------------------------

570 :----------------------------------------------------------------------------------------------------

vi1 :PPNRVTNVMAPPMSKQSA----------------------------------------------------------------------------------

cy1 :PLNRVTSVMAAPVSKRSE----------------------------------------------------------------------------------

fi1 :PLNRVTSVAMAPASKRSE----------------------------------------------------------------------------------

so1 :PLNRITSVATPPMSKRSE----------------------------------------------------------------------------------

in1 :TLNPVTSVAPGLASKLSE----------------------------------------------------------------------------------

hy1 :TLNRVTNVAPSPTSNLSE----------------------------------------------------------------------------------

co1 :PLNRVTDVAAAPANKRTE----------------------------------------------------------------------------------

kn1 :ATSNTTGIADAPASKRIG----------------------------------------------------------------------------------

vi2 :PAEQPADQALTQPTDQPANQPVDQPTDQPIDQPTDQPVDQTTDQTTEQPAGEPLTQSTDEPVDQPLTQSTDQPAGEPLTQSTDEPVDQPLTQSTDQPAGE

cy2 :PADKPADKPADKPADKPADEPADKPADKPADEPADQPTDEPTDEPADKPADKPADEPADKPADKPADQPTDQPTDKPADKPADQPTDQPTDQALTQPADQ

fi2 :TTNQTTDQPTDQPADQTTDQPADQTTDQP-----------------------------------------------------------------------

so2 :TTGQTTGQTTGQTAGQPTDQP-------------------------------------------------------------------------------

in2 :PTDQPTDQPTDQPTDQPTDQPTDQPTDQPTDQPTDQPTDQPTNQPTNQPTNQPTNQPTNQPTDQPTDQP-------------------------------

hy2 :PTDQPTEQPTDQPTDQPIEQPIEQPIEQPIEQPTDQPIEQPTEHPTDQPIEQPIEQPTDQPIEQSTEQPIEQSTEQPIEQPTDLPADHPEDKPADQPTDE

co2 :SAKQSTDKPTDGP---------------------------------------------------------------------------------------

go1 :YPRKEIIT--------------------------------------------------------------------------------------------

go2 :GENPSQRHGSSESVPETNENA-------------------------------------------------------------------------------

vi3 :PSTSGSQPQNVSSVAAPVPPVEATPPPSVTNP--------------------------------------------------------------------

vi4 :AHSPQTPGGPPPTASNIIGTGVSNNGSPPHAAATPGGDNPKQEEATTA-----LKP--------------------------------------------

vi5 :VQSNPPPSTSSGGPNGGGGASITQNVQQANLQAGSDTQVAATPSESFTNP--------------------------------------------------

cy3 :QPSNIPSAASAPSAPNAASDASASSAASADPPEAAPAPAITTNP--------------------------------------------------------

cy4 :VSQDQAHNPGTSEVPPSTESNLIDTGISTNGSPPLAASAPGGDNPIQDEAQASLKP--------------------------------------------

cy5 :ETHNATQVPGTQIPAIPENAQDQSIEETITSNGGQNGDNVVSTVQNPEQANPQADSATQVAAPPPAITTNP-----------------------------

fi3 :ASNAVGPQSPQPNNQDTPAVSQEPSAPRAASPPTVPTPPSPPNADSPVVDPAPTSFTNP-----------------------------------------

so3 :GTPPAVSQQPSDPSPSSAAVVPSADSPVAATAPTSFTNP-------------------------------------------------------------

in3 :TTQDPQPTTTQPGSTAQPGSTTQGDSRTEGVPRTPTPSATQTPPATQTPPATQTPPATQTPPATQTPPATQTPPATQTPPATQTPPATQTPPATQTPPAT

hy3 :SQIANPDESQNQGSVGSTTQNSQATTTPSSSTIEGVSRTATASDTQTPPGTATPPAPQGPAAAQPPAAAQTPSASQTSDAAQGPPAAQPPSATQLATPTP

co3 :ASADAQVAAAPSASFTYP----------------------------------------------------------------------------------

kn2 :GDNAATVQSPEVSNTEVPPQPEANSPDTPPVSQPSSVPTSSSADSPVPATSSASFTNP------------------------------------------

go3 :NLNGAGTLSTEPTSDAPPPVTENAQPAVTNP---------------------------------------------------------------------

vi6 :PRVDQQQQARVADVSGVPSSATETISETSTVVSEQTVVPTPPEAS-------------------------------------------LSSAAPPEASPP

cy6 :ANADPQQPAPVADVSGVPPNPTETNSQASIAEAEKTAAENPPAAT-------------------------------------------LTSSGAPETSSS

fi4 :QQDRVADGSHNAAVQGPPQVPQEGAHSVDSVESSEQGVHADPQQQTPVAAGSGVPSTSTETNPETS------IAEAEQTVAETLPAATLTSAGTPETSSS

so4 :AHEDPPSSAIVQNGPHEAPVPLPPQVPQGVTHTVDSVETSDQGDHTGPQQQDQVADGSVGPSHSTQTNSEGSITEAEQTVAETPPAATLTS-GAPETSSS

fr1 :QQAPALGGSGPPREGTHETTH--------------------------------------------------------------------TSVGAPESSSS

go4 :APNSPADSDTTLETADQGPSQGKHEGPAKPLDEAVETVSESSTLSQPGSS------------------------------------GNETE-G-SE-STS

vi7 :EADQINLPLSNDVPGSEMSVISLPDDDNEEEAKGPSGTHEPTQGADKPLE--------------------------------------------------

vi8 :--------------------------------------------------------------------------------------QLQATPKK------

cy7 :DVPPSQSSTTPTAQGIASASSSGNTNGQGGAT------------------------------------------------------QLQTSTKK------

fi5 :MGSQQLPGAQLQPASPGNGQGGGQSQNQPLPESEVPNPAESNSLQTGQTSNQSGDGTHSESSTTLTPGGSVSPSSPGSTSGPGGATQLQVNSQK------

so5 :DGNPSAISPAPNAEGSVSPSSSGSTRGPGGAT------------------------------------------------------QLQASHKK------

in4 :SSGQDAAA------------------------------------------------------------------------------QLQAIPQK------

hy4 :STGQEVAT------------------------------------------------------------------------------QPQASPQK------

co4 :NGTDSEDSSASTAQGGVSPAPPGSPSGQAGAT------------------------------------------------------QLQAIPKK------

kn3 :GGHSVSSAPSVPGGSASPSSVENSNEQAAGT-------------------------------------------------------QLQVAPQK------

fr2 :GSHSGGSYAAAASGSGSPSSPASPSAQAAAS-------------------------------------------------------QVQPITKK------

go5 :--------------------------------------------------------------------------------------EPAASPKK------

vi9 :EPEAGGEGINRDAAGNQREGQLEAPSDSARPGAIPQVAPRDTVETSSDAADSSSPDQNPLPGADNTKVGNAATPPEGAKEE-------------------

cy8 :REGQPVASQDGALPAENSEDAQGRTDETSSNSVDSPSPDSNPNPVVDNAEAVTVTA-----------------PSEGPKEE-------------------

fi6 :---------VVEGAGVPGVGAGG-VPGVGAGVPEGVPGGVPGGVPG-VPGVPGEVAEAEQAIA----------PSEGTKEE-------------------

so6 :GEPGVGVGGVPGAGPVTGVPGAAPVTGEPAQAVEGGVPGVGAGVQGAVPEVPGAGEEVEQAIA----------PSEGAKEE-------------------

go6 :-------------------------------------------------------------------------PSEGTKKE-------------------

ma1 :AGDRVDASGSGASSIAVQSATPSSQNN-------------------------------------------------------------------------

ma2 :SGSPPNGAEPSEVSGNGQGKSDRSIAGEQRNNDSSEPVTTSNNSD-------------------------------------------------------

ma3 :QQPIMVRLSNEHGNSGSNDVPAERVSSDISNV--------------------------------------------------------------------

ma4 :TETLSQGKDLPNEVATNGGNTNGGTPPHNAPETANTPNI-------------------------------------------------------------

ma5 :----------------------------------------------------------------------------------------------------

ma6 :SPSAVNNGQPNVSSGGAVSPNLSSAGNSNGATQLSAESQNGAVSPKVPNYHNM-----------------------------------------------

ma7 :----------------------------------------------------------------------------------------------------

ov1 :----------------------------------------------------------------------------------------------------

ov2 :----------------------------------------------------------------------------------------------------

ov3 :PSKVGALGETVNGSIGPRLPQKVQGK--------------------------------------------------------------------------

ov4 :----------------------------------------------------------------------------------------------------

yo1 :KDGPKAVGEDGTLISGTEQEGSETDRTKQEKSGSDNNKQGGSPPGKPPGETITQDPSQQKIVDQNGPVKEEPGPKGPVLPPSSPDQDAVNTLESLQSDSP

be1 :PTEISGTGHDGGSDQTKQAETSLGKSNEETITQNPPRQETADQNNPAQEPGSKGPIPSPPSTVSTDQNSGSTLELPQSDSPSEEQLPDSIRIIPIQN---

ch1 :ETEKKVSESDKPKEGEPASDKTQQDSTKQDASPSSVDGQNPQAKGETGSPGPVQPPPESDSTPDATPAVFLEN---------------------------

yo2 :DSSDSSDSSDSSDSSDSSDSSDSSDSSDSSDSSDSSDVTRTKEVTPGTPVGTNTSDQQQSQDALQKAAGGPSSDPASISSSEVIVMDISKIKRRSEHR--

be2 :DSSDSSDSSDSSDSSDSSDSSDSSDSSDSSGTTSETPGTPTGTERSLSPQPQNSQPQNSQTQDSQTQDSQTQQQSQRSEATTGSDSSKNATEEPQSDDAS

ch2 :DSSNSSDSSDPSASSDKSKNPSGTGTDSSSSSQGQDAKNQTQPQPQPQPQPQPQPQTDDNPSSKTNTDNVDASQTLSVTEPAANSTQVVAPKNVQT----

fa1 :NTSSINKQNN------------------------------------------------------------------------------------------

re1 :XXXXXXXXXXXXXXXXXXXXXXXXXXXXXXXXX-------------------------------------------------------------------

fa2 :AGRSSGTGGSVGTKISPGSQGQGKVAGPQLPRLPQLPQSFEQSRNQQSSPVTPKRNGISPTNAKSPESVLPPAQSFTNLNKSTIPHT-------------

re2 :AGRSSGTGGSVGTKISPGSQGQGKVAGPQLP------QSFEQSRNQQSSSVTSKRNDISPINAKSPESVLPADQSYTNLNKSTIPPT-------------

fa3 :GTSTLPSPPKS-----------------------------------------------------------------------------------------

fa4 :PPLVKPENH-------------------------------------------------------------------------------------------

re4 :PPLGKPENH-------------------------------------------------------------------------------------------

fa5 :TSSEKQDT--------------------------------------------------------------------------------------------

re5 :GSTGQTASTPLVTQSSTSSANQST----------------------------------------------------------------------------

fa9 :SDNS------------------------------------------------------------------------------------------------

re9 :SDNS------------------------------------------------------------------------------------------------

ga1 :NPNQADHQNGGADLPEEALNVTVRGANGDAGTSGDSAAALKNATSVN-----------------------------------------------------

vi10 :GSPPSSNNATGGSSNSSG-KKAGNTHIPSRGLQKTKSSARGVTQKGHQTAVDIFGGSTTKSNAPHEGP--------------------------------

cy9 :GDSPASSNNAPGGSNSSSDKKAEHTLIPSQGLQQTKSSSPGVTPQDHQTAVDFFGGNTAKSNDPLEGP--------------------------------

fi7 :GSTPSSNNATAESSSSSSDKKEGLTLIPSQGLHQTKPSSPGVTPQGHQTAVDISGGNTTKSNGPHEDP--------------------------------

so7 :DSPPSSNNSTGGSTSSS-DKKEENTLIPSEGLQQTKPSAHGVTPQGHPTAEDIYGGNTTKSNDPHEGP--------------------------------

in5 :GSPPSSNNTTGGSSTSSG-KKEEHTVIPLGGDSQTKPSAPGVTPKGHQRAVDMFRGNTTKSIGPLKGP--------------------------------

hy5 :SSPPSSDNATGGSSTSSG-KKEEHTVIPPGGDSQTKPSAPEVTSQGHQKAVGLFRGNTTKWNGSLKGP--------------------------------

co5 :GSPPSSTNATGGSSTSSG-KEAENKVIPPQKSEQTKPFATEVTGQGHQTALEIFGGNTTNSNGPLDGP--------------------------------

kn4 :GSLPSSTNATEKSNTSSG-KEAEHTAISSGEREKTEPSTNEVAGEGHQTAVAMFEGNKTKSNGSLDGS--------------------------------

fr3 :ASPPPSSNNDTEEDSSSRDKKEEHTDITSPELQPTTPPASEVIAQSNQTAVQIFEGNTTQSNDLINGP--------------------------------

go7 :NSSQSSNNVTEDGMSNST-SKLGPPFISSRGLQQTKPYARSVPMQGKKEGENNNSQNRSKSDGPPEGP--------------------------------

ma8 :VTSLPQPEGTTSTVTPPGPVSTGVSESSDNSGRTSLVTSESGDASANTASAVVLTETGQLGQLQQPQQPRSPVTPSQGLQQPGSTPVKDNLEPGSPDKP-

ov5 :EPKVVPPLAQTVEGTPAQGGSKESGMEPTSEAAKEVVSISKGELPPVSEIAPPQTQQPPSNVVMPLTKGGSAGLGSNTSKSGLSEKS-------------

yo3 :QDSGSSGNKVPGAPSDDVNNPTGDLSTGLSGGKVTGASSDHVPGPTGEQGSDSKGGQKKEQPPPKEPAPTTPKEPAPTTPKEPEPTTPKEPEPTTPKEKA

be3 :PKEPEPTTPKEPESATPKASEPVTPQKTAETASGKQVSPTPSENPPSKDTPKPESSSEKKVNSALATPPAPEVSKAQEGAGLATQKEQTPSKRAKRSPPP

ch3 :PTTPKEPAPTTPKEPAPTTPKEPEPTTPKEPEPTTPKESEPTTPKEPAPTTQKDSGSQSVEKSGITTNDGPAVITGPDSSTGDASLPSAGDQKGEKKENQ

fa6 :GATVSHTKVTSEGLSDTQPNVTQSVSSSTHTPGSLDSTMSTEQHSSVSQSSLPTE-------SSSETLNKATVPEIP-----------------------

re6 :GATVSHTKVPSEGSSDTQPNITPSVSSSTHTPGSLDSTVSTEQHSSVSQSSLPTEHPSNTTESSSETLNKATVAEMP-----------------------

vi11 :VTKSPSSATEP---------------------------------------ESAGAASTQAEDDAEQG---------------------------------

cy10 :ES------------------------------------------------ESSDAASVQVEDTSEQN---------------------------------

fi8 :GVGGPGEGSGGPGADAGDPSLSVPGQASQDADLSSSSSANGIGSSSSSSSVGETALPGISAEAKSNAMESESAGAATMQAEEASEQD-------------

so8 :GV----------------PSLSVPGQASQDANPSSSLSTNGIGSSSSS-PVGETVLPTRNEDAESNAIESEPAGSASMQDADASEQN-------------

in6 :ESKSAGAASIQTADPSEQD---------------------------------------------------------------------------------

hy6 :QSTSAGGVSIQAADPSEKD---------------------------------------------------------------------------------

co6 :----------------------------------------------------------------------------------------------------

kn5 :----------------------------------------------------------------------------------------------------

fr4 :----------------------------------------------------------------------------------------------------

go8 :VGTEHQQPTAPPQDPSADGTQNQPPTSPPTPSASETRSPDENPISQMTVAEPEGDTSAETSDDSEQG---------------------------------

ma9 :PSKPSPHLNTNPQQPNREAPQAVQSDGQNVEQG-------------------------------------------------------------------

ov6 :EEKVYVKNLDKG----------------------------------------------------------------------------------------

yo4 :----------------------------------------------------------------------------------------------------

be4 :----------------------------------------------------------------------------------------------------

ch4 :----------------------------------------------------------------------------------------------------

fa7 :LVTPIPLPKLTLEDSESSKSVID-----------------------------------------------------------------------------

re7 :VVTPIPLPKLALEDSESSKSVTG-----------------------------------------------------------------------------

vi12 :----------------------------------------------------------------------------------------------------

cy11 :----------------------------------------------------------------------------------------------------

fi9 :----------------------------------------------------------------------------------------------------

so9 :----------------------------------------------------------------------------------------------------

in7 :----------------------------------------------------------------------------------------------------

hy7 :----------------------------------------------------------------------------------------------------

co8 :----------------------------------------------------------------------------------------------------

kn6 :----------------------------------------------------------------------------------------------------

fr5 :----------------------------------------------------------------------------------------------------

go9 :----------------------------------------------------------------------------------------------------

ma10 :----------------------------------------------------------------------------------------------------

ov7 :----------------------------------------------------------------------------------------------------

yo5 :----------------------------------------------------------------------------------------------------

be5 :----------------------------------------------------------------------------------------------------

ch5 :----------------------------------------------------------------------------------------------------

fa8 :----------------------------------------------------------------------------------------------------

re8 :----------------------------------------------------------------------------------------------------

ga2 :----------------------------------------------------------------------------------------------------

ga3 :----------------------------------------------------------------------------------------------------

392 :-------210-------220-------230-------240-------250-------260-------270-------280-------290-------300

392 :----------------------------------------------------------------------------------------------------

570 :----------------------------------------------------------------------------------------------------

vi1 :----------------------------------------------------------------------------------------------------

cy1 :----------------------------------------------------------------------------------------------------

fi1 :----------------------------------------------------------------------------------------------------

so1 :----------------------------------------------------------------------------------------------------

in1 :----------------------------------------------------------------------------------------------------

hy1 :----------------------------------------------------------------------------------------------------

co1 :----------------------------------------------------------------------------------------------------

kn1 :----------------------------------------------------------------------------------------------------

vi2 :PLTQSTDEPVDQPLTQSTDQPAGEPLTQSTDQPAGEPLTQSTDQPADQPADQSADQPVDQTTDQVTEQPTDQPTEQPTDQTTDQPTEQPTDEPLTQPTDE

cy2 :PADQPTEQPTEQPTEQPADQPTDQPTEQPAEQPTEQPADQPTDQPTEQP---------------------------------------------------

fi2 :-LTQPNVEASDRAAAAAVKNPNE-----------------------------------------------------------------------------

so2 :-LTQPNVEASDRAAAAAVKNPNE-----------------------------------------------------------------------------

in2 :-LTQPPVEVSDKAAAAAVRNPNE-----------------------------------------------------------------------------

hy2 :TFTQPTVEVSNKAAAAAVRNPNE-----------------------------------------------------------------------------

co2 :-LTQPTVEVSSKAAAAAVRNPNE-----------------------------------------------------------------------------

go1 :-KSVILPKVKSSAEVAALKNPNA-----------------------------------------------------------------------------

go2 :----------------------------------------------------------------------------------------------------

vi3 :----------------------------------------------------------------------------------------------------

vi4 :----------------------------------------------------------------------------------------------------

vi5 :----------------------------------------------------------------------------------------------------

cy3 :----------------------------------------------------------------------------------------------------

cy4 :----------------------------------------------------------------------------------------------------

cy5 :----------------------------------------------------------------------------------------------------

fi3 :----------------------------------------------------------------------------------------------------

so3 :----------------------------------------------------------------------------------------------------

in3 :QTPPATQTPPATQVAAPLPPSSPNP---------------------------------------------------------------------------

hy3 :ASSPNP----------------------------------------------------------------------------------------------

co3 :----------------------------------------------------------------------------------------------------

kn2 :----------------------------------------------------------------------------------------------------

go3 :----------------------------------------------------------------------------------------------------

vi6 :SGNS------------------------------------------------------------------------------------------------

cy6 :NDST------------------------------------------------------------------------------------------------

fi4 :NGGG------------------------------------------------------------------------------------------------

so4 :NESA------------------------------------------------------------------------------------------------

fr1 :NDGG------------------------------------------------------------------------------------------------

go4 :TSSVTTPNK-------------------------------------------------------------------------------------------

vi7 :----------------------------------------------------------------------------------------------------

vi8 :----------------------------------------------------------------------------------------------------

cy7 :----------------------------------------------------------------------------------------------------

fi5 :----------------------------------------------------------------------------------------------------

so5 :----------------------------------------------------------------------------------------------------

in4 :----------------------------------------------------------------------------------------------------

hy4 :----------------------------------------------------------------------------------------------------

co4 :----------------------------------------------------------------------------------------------------

kn3 :----------------------------------------------------------------------------------------------------

fr2 :----------------------------------------------------------------------------------------------------

go5 :----------------------------------------------------------------------------------------------------

vi9 :----------------------------------------------------------------------------------------------------

cy8 :----------------------------------------------------------------------------------------------------

fi6 :----------------------------------------------------------------------------------------------------

so6 :----------------------------------------------------------------------------------------------------

go6 :----------------------------------------------------------------------------------------------------

ma1 :----------------------------------------------------------------------------------------------------

ma2 :----------------------------------------------------------------------------------------------------

ma3 :----------------------------------------------------------------------------------------------------

ma4 :----------------------------------------------------------------------------------------------------

ma5 :----------------------------------------------------------------------------------------------------

ma6 :----------------------------------------------------------------------------------------------------

ma7 :----------------------------------------------------------------------------------------------------

ov1 :----------------------------------------------------------------------------------------------------

ov2 :----------------------------------------------------------------------------------------------------

ov3 :----------------------------------------------------------------------------------------------------

ov4 :----------------------------------------------------------------------------------------------------

yo1 :SAESVPSIIESIPFQD------------------------------------------------------------------------------------

be1 :----------------------------------------------------------------------------------------------------

ch1 :----------------------------------------------------------------------------------------------------

yo2 :----------------------------------------------------------------------------------------------------

be2 :ISSNETNVVNNSVIKPRIVHK-------------------------------------------------------------------------------

ch2 :----------------------------------------------------------------------------------------------------

fa1 :----------------------------------------------------------------------------------------------------

re1 :----------------------------------------------------------------------------------------------------

fa2 :----------------------------------------------------------------------------------------------------

re2 :----------------------------------------------------------------------------------------------------

fa3 :----------------------------------------------------------------------------------------------------

fa4 :----------------------------------------------------------------------------------------------------

re4 :----------------------------------------------------------------------------------------------------

fa5 :----------------------------------------------------------------------------------------------------

re5 :----------------------------------------------------------------------------------------------------

fa9 :----------------------------------------------------------------------------------------------------

re9 :----------------------------------------------------------------------------------------------------

ga1 :----------------------------------------------------------------------------------------------------

vi10 :----------------------------------------------------------------------------------------------------

cy9 :----------------------------------------------------------------------------------------------------

fi7 :----------------------------------------------------------------------------------------------------

so7 :----------------------------------------------------------------------------------------------------

in5 :----------------------------------------------------------------------------------------------------

hy5 :----------------------------------------------------------------------------------------------------

co5 :----------------------------------------------------------------------------------------------------

kn4 :----------------------------------------------------------------------------------------------------

fr3 :----------------------------------------------------------------------------------------------------

go7 :----------------------------------------------------------------------------------------------------

ma8 :----------------------------------------------------------------------------------------------------

ov5 :----------------------------------------------------------------------------------------------------

yo3 :GDDPEKQVSPEGSASKTVEKKELSSSGDSLPSSSEDPGSPQKKPAGEEDPPSPPKKEESLPAKKPEAPPAKPAAPESLKVSQAGLDPNAQKQGQGKRPKR

be3 :QVNNI-----------------------------------------------------------------------------------------------

ch3 :DPASLPSEDTTDEQTKKETEQESLPTSKGEPESLSEKPSGDPAPKPAEDPASPPAKEPESETPKVSQDATDLNTQKQGEPNKRSRRSPQPQVPETKKVND

fa6 :----------------------------------------------------------------------------------------------------

re6 :----------------------------------------------------------------------------------------------------

vi11 :----------------------------------------------------------------------------------------------------

cy10 :----------------------------------------------------------------------------------------------------

fi8 :----------------------------------------------------------------------------------------------------

so8 :----------------------------------------------------------------------------------------------------

in6 :----------------------------------------------------------------------------------------------------

hy6 :----------------------------------------------------------------------------------------------------

co6 :----------------------------------------------------------------------------------------------------

kn5 :----------------------------------------------------------------------------------------------------

fr4 :----------------------------------------------------------------------------------------------------

go8 :----------------------------------------------------------------------------------------------------

ma9 :----------------------------------------------------------------------------------------------------

ov6 :----------------------------------------------------------------------------------------------------

yo4 :----------------------------------------------------------------------------------------------------

be4 :----------------------------------------------------------------------------------------------------

ch4 :----------------------------------------------------------------------------------------------------

fa7 :----------------------------------------------------------------------------------------------------

re7 :----------------------------------------------------------------------------------------------------

vi12 :----------------------------------------------------------------------------------------------------

cy11 :----------------------------------------------------------------------------------------------------

fi9 :----------------------------------------------------------------------------------------------------

so9 :----------------------------------------------------------------------------------------------------

in7 :----------------------------------------------------------------------------------------------------

hy7 :----------------------------------------------------------------------------------------------------

co8 :----------------------------------------------------------------------------------------------------

kn6 :----------------------------------------------------------------------------------------------------

fr5 :----------------------------------------------------------------------------------------------------

go9 :----------------------------------------------------------------------------------------------------

ma10 :----------------------------------------------------------------------------------------------------

ov7 :----------------------------------------------------------------------------------------------------

yo5 :----------------------------------------------------------------------------------------------------

be5 :----------------------------------------------------------------------------------------------------

ch5 :----------------------------------------------------------------------------------------------------

fa8 :----------------------------------------------------------------------------------------------------

re8 :----------------------------------------------------------------------------------------------------

ga2 :----------------------------------------------------------------------------------------------------

ga3 :----------------------------------------------------------------------------------------------------

392 :-------310-------320-------330-------340-------350-------360-------370-------380-------390-------400

392 :----------------------------------------------------------------------------------------------------

570 :-------------------------##################----######################-------------------------------

vi1 :-----------------------VEVRSALLKNHDGVKITGTC-NAKVQLFLVPHISISVEAESNTIQLGRKLEDVTITKEFYRGVGGKSSP-LLQFEE-

cy1 :-----------------------VEVRSALLKNHDGVKITGTC-NAKFQLFLVPHISISVEAESNTIQLGRKLEVVTITKKQHKVVGSKSSP-LLQFEE-

fi1 :-----------------------VEVRSALLKDHNGVKITGTC-NAKFQLFLVPHISISVEAESNTIQLGRKLEDVMITKEFYK-GDRNSSQ-RLQFEE-

so1 :-----------------------VEVRSALLKDHDGVKITGTC-NAKFQLFLVPHISINVEAESNTIQLGRKLEDVTITKEFYK-GDRNSSQ-RLQFEE-

in1 :-----------------------VEVRSALLKDHDGVKVTGTC-NAKFQLFLVPHISISVEAESNTIQIGRKLEDVTMTKEFYK-GDSDSSE-RLQFEE-

hy1 :-----------------------VEVRSALLKGHDGVKVTGTC-NAKFQVFLVPHISISVEAESNTIQIGRNLKDVTMTKEFYK-GDRNPSE-RLQFEE-

co1 :-----------------------VEVRSALLKNHDGVKITGTC-NAKFQLFLVPHVSISVEAESNTIQLGRKLENVTITKKQHKVISSESSP-LLQFEE-

kn1 :-----------------------VEVRSALLKDHDGVKITGTC-NAKFQVILVPHISISVEAESNTIQISRMIENVTTTKEWDK-GDGNSSQ-PLQFEE-

vi2 :PLPQPIVEASDRAAAAAVKNPNEIEAKCAQLKDQDGVKITGPC-GAKFQVFLIPHVTINVETETNAIHLGKKLDDVVITKKMHKGVGGKSPP-LLQFEE-

cy2 :-LTQPNVEASDRATAAALKNPNEIEAQCAQLKDQDGVKITGLC-RAKFQVFLVPHVTINVETETNTIYIGKKLDDVVITKKQNKVVSSKSSP-LLQFEE-

fi2 :-----------------------IEATCAQLKDQDGVKITGPC-RAKFQVFLVPHLTINVETETNTIYIGKKLDDVAITKKQHKMVSSKSPP-LLQFGE-

so2 :-----------------------IEAKCAQLKDQDGVKITGPC-RAKFQVFLVPHVTINVETETNTIYIGKKLDDVAITKKQNKVVSSKSSP-LLQFEE-

in2 :-----------------------IEAKCSQLKDQEGVKITGPC-GAKFQVFLVPHVTINVETETNTIYIGKKLDDVIITKKQHKVVSGKSSP-LLQFEE-

hy2 :-----------------------IEAKCSQLKDQDGVKITGPC-GAKFQVFLVPHVTINVETETNTIYIGKKLDDVIITKKQQKVVSSKSSP-LLQFEE-

co2 :-----------------------IEAKCEQLKDQDGVKITGPC-GAKFQVFLVPHVTINVETETNTIYIGKKLNDVIITKKQHKVISSESSP-LLQFEE-

go1 :-----------------------IEVSSALLKDQDGIKITGLC-NARFQLFLVPHILINVETENNKIYLGKKFQDVIITKELHKGI-GDPNE-RLQFEE-

go2 :-----------------------IKINSSFLKNHTGVKITGPC-SSEFLVVLVPHIYIEVDAIMDIIRLGPKLNDAEG---------------RVEFP--

vi3 :-----------------------FKVKSSLLKDQKGLKITGPC-ESYFQVYLVPYLYMNVNATSSEIEMEPMFMKVDD---------------KIKFEK-

vi4 :-----------------------IQVKSALLKDHKGVKVTGPC-DATFQVYLVPYLYIDVNAKNTEIEMDPMFTKVDN---------------KIKFEE-

vi5 :-----------------------IQVKASLLRDQKGLKITGPCKS-YFQVYLVPYLYLNVNAKESEIEMDPMFMKVDD---------------KIKFEK-

cy3 :-----------------------IEVKSSLLKDQKGLKITGPC-KSFFQVYLVPYLYMNVNAERSEIEMEPLFMKVDN---------------RIKFEK-

cy4 :-----------------------IQVKSALLKDQKGIKITGPC-DATFQVFLVPYLYIDVNAKNTDIEMDPMFTKVDD---------------KIKFEQ-

cy5 :-----------------------IEVKSSLLKDQKGLKITGPCKS-FFQVYLVPYLYMNVNAERSEIEMEPLFMKVDN---------------RIKFEK-

fi3 :-----------------------IKVQASLLKDQKGLKITGPC-ESYFQVYLVPYLYMNVNAASSEIEMEPLFMKVDE---------------KIKFEK-

so3 :-----------------------IKVQASLLKDQKGLKITGPC-NSYFQVYLVPYLYMNVNAAISEIEMEPLFMKVDD---------------KIKFEK-

in3 :-----------------------IQVKSFLLRDHKGLKIIGPCKSSYFQVYLVPYLYMNVNAANSEIEMDPMFMKVDD---------------KIKFER-

hy3 :-----------------------IQVKSFLLRDHKGLRITGPCKSSFFQVYLVPYLYINVNAANTEIEMDPMFMKVDE---------------KIKFEK-

co3 :-----------------------IHVKASLLKNYKGVKVTGPC-GSYFQVYLVPYLYVNVNSGGSEIEMEPLFMKVDN---------------KIKFEK-

kn2 :-----------------------IQVKASLLKNYKGVKVTGPC-GSYFQVYLVPYLYMNVNSSNSEIEMEPLFMKVDN---------------KIKFEK-

go3 :-----------------------FAVKSALLKDHNGLKITGPC-DSYFQVYLVPYLYINVKPLESEIEMEPTFMKVDE---------------KIKFEK-

vi6 :-----------------------IQVITALLKDANGVKVTGAC-GAHFELSLVPHISISAETKTNDIKLRPILHKLLDAK--EADVGTTKLDNAIQFEP-

cy6 :-----------------------IQVKTALLKDANGVKVTGPC-GSHFELYLVPHISISAETKTNSIKLRPKLHKLIDSK--EATAGKVKFDNAVQFEE-

fi4 :-----------------------IQVKAALLKDANGVKVTGPC-GAHFELSLVPHISISAETKTNDIKLRPKLHKLIDLK--DTTAGTIKFYNAIQFEE-

so4 :-----------------------IQVKTALLKDANGVKVTGPC-GAHFELSLVPHISISAETKTNDIKLRPKLHKVIDSK--DVTTGTVQFDKAIQFEE-

fr1 :-----------------------IQVKTALLKDANGVKVTGPC-GANFEISLVPHMSISAETSTNDIKLRPKLHKLSDTN--EAKDEIMKLYNAIQFGK-

go4 :-----------------------IQIKSALLKDFNGVKVTGLC-GAIFDLTLVPHISITVDTDENIIKLRPKFQKLIDEE-TSTTAGIAKLYNAVHFEE-

vi7 :-----------------------ISITSALLKDHNGVKITGPC-SADFQLFLVPHIIIHVESAKDKIELRSKWEDVDNKQSKGAKVPVEELKSGILFKQ-

vi8 :-----------------------AELQSSLLKNFTGVKVTGPC-DTEVGLYLIPYIYISVNAKTDTIELSTRFPNSDNV--------------LVQFKK-

cy7 :-----------------------AELQSALLKNFTGVKVTGPC-DTEVGLFLIPHMYISVKAAKDIIELSTKFPNSDNT--------------IIQFTK-

fi5 :-----------------------AELQSALLKNFTGVKVTGPC-DTEVGLFLIPHMYISVKVGTDKIELSTKFPNSDNM--------------LVEFTK-

so5 :-----------------------AELQSALLKNFTGVKVTGPC-DTEVGLFLIPHMYISVKVATDKIELSTKFPNSDNT--------------LVEFTK-

in4 :-----------------------AQLQSALLKNFKGVKVTGPC-DTEVGLFLIPHIYIFVNAAADTIELSTKFPNSDNF--------------LVEFKK-

hy4 :-----------------------APVQSALLKNLKGVRVTGPCNTEVVGLFLIPHIYISVNAASDTIELTSKFPNSDNL--------------LLEFKK-

co4 :-----------------------AEVKSALLKNFTGVKVTGPC-DTEVGLYLIPYIYISVKVAADTIELSTKFPNSDNI--------------IVAFTD-

kn3 :-----------------------AQVKSALLKNFTGVKVTGPC-DTEVGLFLIPYIYISVKVAEDNIELSTNFPNSENI--------------IVKFSN-

fr2 :-----------------------AEIKSALLKNFTGVKVTGPC-DTEVGLFLIPYIYISVKVADNNIELSTKFPNADNI--------------MVQFSD-

go5 :-----------------------DEIKSALLKNYAGVKVTGPC-DSDVGLYLIPYIYISVMPKDDIIQLSTSFPDSDNT--------------IVEFKN-

vi9 :-----------------------TQVKSSLLKGHKGVKVTGPC-GASFLVFFAPYLFIDVDTDSSNVYLGTDLSDLEVTEKMG-----------------

cy8 :-----------------------IPVKSSLLKGYKGVKVTGPC-SASFLVFFAPYLFIDVDAESSNIYLGTDLSDLEVTEKMG-----------------

fi6 :-----------------------IQVKSSLLKGHKGVKVTGTC-NASFLVFFAPYLFIDVDAESSNIYLGTDLSDLEVTEKMG-----------------

so6 :-----------------------IQVKSSLLKGYKGVKVTGTC-NASFLVFFAPYLFIDVDAESSNIYLGTDLSDLEVTEKMG-----------------

go6 :-----------------------IQIKSSLLKGRKGVVVTGSC-NASFLVFFVPYLFIDVDTESSNVYMGTDVNYLDVTEYMGNKD--------------

ma1 :-----------------------INVKSALLKDHKGVKVTGTC-NAKVQLFLVPHISITLETKESKIRLGPKYEDSDITKEFQTDNEELDIKKIFEI---

ma2 :-----------------------IQINSALLKDHKGVKITGLC-NVDFLVFLVPYIYIHVKTDVDLILLRTKSNNQTINVDFS-----------------

ma3 :-----------------------VQVKSALLKEHKGIKITGPC-TAKFYVFLIPFLEIYVDAENNEIEMNPIFIKITEKILFEK----------------

ma4 :-----------------------ILVKSALLKDYNGVKITGPC-KAKFHLFLVPHITIEVETENNKIILKSKLDELKKKKKGQSIDQDLNKNLEFEK---

ma5 :-----------------------DKVKSAFLKNETGVKLNGVC-GAEVGIFLVPHIYIYLQTKDTNIQLQSELIPIDKTIIL------------------

ma6 :-----------------------AKIESALLKNHTGVRITGPC-NEEVGVFLIPYIYITVKSKTDNIELSSKFPQSDNEVLEFKK---------------

ma7 :-----------------------AKIQSALLTDSNGVMVTGPC-NEIFQVFFVPNIFINVQTDKNTVEMGNKFKSLSNSITLK-----------------

ov1 :-----------------------SILPECTGESKGEDVKGGAC-SNSAPIEDLTLDRIEVVSIDKDGEDVEEGQNNEEKTENYIQKIIHSRTHSTVSHIR

ov2 :-----------------------PKVKSFFLKDEKGVKLEGSC-GTEIGLFLVPHVYIYAQTKTTNVQIISELIPTTKKIEL------------------

ov3 :-----------------------DMVESSLLKNYTGIKVSGPC-GEEIGIFLIPYIYITVQTEIDSIKVATKFPQSDNEILEFKN---------------

ov4 :-----------------------THVESALLKDHKGIMVTGPC-KQNFLVFLLPHIFIEVDTENDTVQMGNDLKFLSDAINLS-----------------

yo1 :-----------------------FDIKSAMLKNYKGLKITGQC-NSSFFMFFVPYVYIDVDTKINQISIFSTKKKVNGTTNDVPTQLIEFKS--------

be1 :-----------------------FDIKSAMLRNYKGLKITGQC-NSNFVIFFVPYIYIEVDTKLNQISIISTKKKVNGTTEGVPTQSIDFKS--------

ch1 :-----------------------FNVKSAMLKNYKGLKITGEC-NADLSIFFVPYILIDVNTKTNQIFIMSTKKKVMGTTKGVATPSIDFKS--------

yo2 :-----------------------GTVESALLKNYKGVKVTGSC-GDEVGIFLEPYIYISVDSKNNVMDLSATFPNVLGQHITIFS---------------

be2 :-----------------------GTVESALLKNYKGVKVTGLC-GDEVGIFLEPHIYINVESKINNIELSAKFPSIHGQYITIFS---------------

ch2 :-----------------------GSVESALLKNFNGVKVTGAC-GDEAAIFLEPYIYINIDSKNTNINLSANFSNLFGQHITVFA---------------

fa1 :-----------------------NQIKSVLLKENKGVKITGPC-NVNLSIFLVPHIYIDVETKYNNIELKYELDEFSDSIKFKDTTTELRTSDDTLMNTN

re1 :-----------------------------------------------LXIFLVPHIYIDVXXKSNNIELKYELDEFSDSIKFKDTTTELSTSDDTLMNTN

fa2 :-----------------------IPIKSSFLKYYKGVKITGSC-GVQFQLVIVPHLFIYVETKENNIQLEPRFMKLNERIDFEK----------------

re2 :-----------------------IPIKSSFLKYYKGVKITGSC-GVQFQLVIVPHVFIIVETNENNIQLEPRFMKLNERIDFEK----------------

fa3 :-----------------------IDVKSAFLKHYKGVKVTGSC-NANFQLFLVPHIFINVETKENNIQLDVKFLKLTKRIDFAK----------------

fa4 :-----------------------KTIVSAMLKNYKGVKVTGTC-GADFGLFLVPHIYVHVKSEDTEIELSSELAPPEMQTKF------------------

re4 :-----------------------KTIDSAMLKNYKGVKVTGTC-GADFGLFLVPHIYVHVKSKDTEIELSSELAPPEMQTKF------------------

fa5 :-----------------------IQVKSALLKDYMGLKVTGPC-NENFIMFLVPHIYIDVDTEDTNIELRTTLKKTNNAISFESNSGSLEKKKYVKLPSN

re5 :-----------------------IQVKSALLKDYMGLKVTGPC-NENFIMFLVPHIYIDVDTEDTYIELRTTLKETSNALSFEANSGSLEKKKYVKLQSN

fa9 :-----------------------IKIKSAFLKENVGVKITGPC-DEGFGLVMVPHIIINVDTRNTDIQLGKKSSEINYGIKLRKKGKDEYEEKV------

re9 :-----------------------IKIKSAFLKENVGVKITGPC-DEGFGLVMVPYIIINVDTRNTDIQLGKKSAEINYGIKLRKKGKDEYEEKV------

ga1 :-----------------------MPIKSALLKKNTGVKVTGKC-GNYFQLSFVPHMTLNFETDDGKIELEQKFAASEII---------------------

vi10 :-----------------------HHVESALLKNYKGVKVTGSC-GSYFRVYLVPHILIYALTKNSIIQIESLFDDNTRVDFE------------------

cy9 :-----------------------QQVESALLKNYNGVKVTGSC-GSYFRVYLVPHILIYALTKSSIIQIESLFDDNTRIDFE------------------

fi7 :-----------------------LQVESALLKNYKGVKVTGSC-GSYFRVYLVPHILIYVLTKNSTIQIESLFDDNTRIDFE------------------

so7 :-----------------------HQVESALLKNYKGVKVTGSC-GSYFRVYLAPHILIYVLTKNSTIQIESLFDDNTRVDFE------------------

in5 :-----------------------LQVESALLKNHKGVKVTGLC-GSYFRVYLVPHILIYALTKNSTIQIESLFDDNTRVDFE------------------

hy5 :-----------------------LQVESALLKKHKGVKITGLC-GSYFRVYLVPHILIYALTKNSTIQIESLFDDNTRVDFE------------------

co5 :-----------------------MQVESALLKNYKGVKVTGPC-GSYFRVYLVPHILIYALTKNSIIQIESLFDDNTRIDFE------------------

kn4 :-----------------------MQVESALLKNYKGIKVTGPC-GSYFRVYLVPHILIYALTKNSIIQIESLFDDNTRIDFE------------------

fr3 :-----------------------MQVESALLKNYNGIKVTGPC-GSYFRVYLVPHILIYALTKNTIIQVESLFDDNTRVDFE------------------

go7 :-----------------------VKVESALLKNYNGIKVTGSC-GSYFRVYLAPHILIYVLTKNSLIQIESLFDDNTRIDFE------------------

ma8 :-----------------------IQVESSLLKNFDGVKIVGSC-GAYFRVHLVPHILIYALTKYSIIQIESLFNENTKIDFE------------------

ov5 :-----------------------TEVESFLLKNYKGVKVSGLC-GAYFRVYFVPHILIYALTKHSILQIESLFDDNTRIDFE------------------

yo3 :ALPPQVQVPQVNDI---------TDMESYLLKNYDGVKVIGLC-GVYFRVQFSPHLLLYGLTKFSIIQIEPFFER-VRIDFE------------------

be3 :-----------------------TDMESYLMKNYDGVKVIGLC-GVYFRVQFSPHLLLYGLTTFSIIQIEPFFEG-VRIDFE------------------

ch3 :I----------------------TDMESYLMKNYDGVKVIGLC-GVYFRVQFSPHLLLYGLTKFSIIQIEPFFER-VRIDFE------------------

fa6 :-----------------------IQINSGLLKNYNGVKVTGSC-GSYFRVYLVPHILIYALTKYSVIQLESLFNDNARIDVE------------------

re6 :-----------------------IQINSGLLKNYKGVKVTGPC-GSYFRVYLVPHILIYALTKYSVIQLESLFNDNARIDVE------------------

vi11 :-----------------------IQITSALLKHFNGIKITGSC-KEPFRVFLSPHLWIYAVPPENKIQLRPNFGPITSI---------------------

cy10 :-----------------------IQITSALLKHSNGIKITGSC-KAHFRVFLSPHLWIYAVAAENKIQLRPNFGPITSI---------------------

fi8 :-----------------------IQITSALLKNSNGIKITGSC-KAHFRVFLSPHLWIYAVAPENKIQLRPNFGPITSI---------------------

so8 :-----------------------IQITSALLKHSNGIKITGSC-KAHFRVFLSPHLWIYAVAAENKIQLRPNFGPIISI---------------------

in6 :-----------------------TQITSALLKHSNGIKITGSC-KAHFRVFLSPHLWIYVVAAENKIQLRPNFGPITSV---------------------

hy6 :-----------------------TQLTSALLKHSNGIKITGSC-KAHFRVFLSPHLWIYVVAAENRIQLRPNFGPITSV---------------------

co6 :-----------------------VQITSALLKNYNGIKITGSC-KAHFRVFLLPHLWIYAAAPENKIQLRPNFGPITSI---------------------

kn5 :-----------------------IQVTSALLKNYNGIKITGSC-KAHFRVFLAPHLWIYAAAPENKIQLRPNFGPITSI---------------------

fr4 :-----------------------VQITSAILKKYNGIKITGSC-KAHFRVFLSPHLWLYVEAAENKIQLKPNFGQISSI---------------------

go8 :-----------------------IQIRSALLKQLNGVKITGSC-KAHFRVYLFPHLWIYAVTGENKIQLSLNFGSIPSI---------------------

ma9 :-----------------------IQVTSTFLKNFDGIKVTGSC-KAYFRVFFVPHIWIYVDPTKDSIELKQLFEPVRTV---------------------

ov6 :-----------------------IHVTSTLLKNFEGIKVTGLC-KAHFRVFFVPYIWIYVLTTEDKIHLKPSFELNKSV---------------------

yo4 :-----------------------TLIKSSLSKNGKGIKAIGVC-NEDFRIYFGPYIWIYVTLSENIIKIEPENGVRTSI---------------------

be4 :-----------------------TLIKSSLSKNGKGIKVIGIC-DEDFRIYFAPYIWIYVTLSENIIKIEPENGVSTSI---------------------

ch4 :-----------------------TSIKSSLSKDGKGIKVTGTC-NEDFRIYFSPYVWIYVTFSEGIIKIESENGVTSSI---------------------

fa7 :-----------------------IEVKSALLKNYDGVKITGPC-RSYFRVMLVPHITVYVYATYDRIQLEPKFGPSDLI---------------------

re7 :-----------------------IEVKSALLKNYDGVKITGPC-RSYFRVMLVPYITVYVYATYDRIQLEPKFGPSDLI---------------------

vi12 :----------------------------------------------------------------------------------------------------

cy11 :----------------------------------------------------------------------------------------------------

fi9 :----------------------------------------------------------------------------------------------------

so9 :----------------------------------------------------------------------------------------------------

in7 :----------------------------------------------------------------------------------------------------

hy7 :----------------------------------------------------------------------------------------------------

co8 :----------------------------------------------------------------------------------------------------

kn6 :----------------------------------------------------------------------------------------------------

fr5 :----------------------------------------------------------------------------------------------------

go9 :----------------------------------------------------------------------------------------------------

ma10 :----------------------------------------------------------------------------------------------------

ov7 :----------------------------------------------------------------------------------------------------

yo5 :----------------------------------------------------------------------------------------------------

be5 :----------------------------------------------------------------------------------------------------

ch5 :----------------------------------------------------------------------------------------------------

fa8 :----------------------------------------------------------------------------------------------------

re8 :----------------------------------------------------------------------------------------------------

ga2 :----------------------------------------------------------------------------------------------------

ga3 :----------------------------------------------------------------------------------------------------

392 :-------410-------420-------430-------440-------450-------460-------470-------480-------490-------500

392 :----------------------------------------------------------------------------------------------------

570 :---------------------------------------------------------------------------------------------###----

vi1 :---------------------------------------------------------------------------------------DADSLLNQCAEG-

cy1 :---------------------------------------------------------------------------------------DEKFLLNQCAEG-

fi1 :---------------------------------------------------------------------------------------NADFLLNQCAEG-

so1 :---------------------------------------------------------------------------------------NMDFLLNQCAEG-

in1 :---------------------------------------------------------------------------------------NADLLVNQCAEG-

hy1 :---------------------------------------------------------------------------------------NANLLLNQCSDG-

co1 :---------------------------------------------------------------------------------------NVHSLVNQCTGR-

kn1 :---------------------------------------------------------------------------------------NIDSLLNQCTEG-

vi2 :---------------------------------------------------------------------------------------DADSLLNQCTEG-

cy2 :---------------------------------------------------------------------------------------NADSLLNQCAEG-

fi2 :---------------------------------------------------------------------------------------NTDFLLNQCAEG-

so2 :---------------------------------------------------------------------------------------NADLLLNQCAEG-

in2 :---------------------------------------------------------------------------------------NSNLLLNQCVDG-

hy2 :---------------------------------------------------------------------------------------NANLLLNQCSDG-

co2 :---------------------------------------------------------------------------------------NVHSLVNQCTGR-

go1 :---------------------------------------------------------------------------------------NPNSLVNKCAKG-

go2 :---------------------------------------------------------------------------------------VNTNLKNQCGTG-

vi3 :---------------------------------------------------------------------------------------EKHLLHNICAAD-

vi4 :---------------------------------------------------------------------------------------GKHRLMNICEKG-

vi5 :---------------------------------------------------------------------------------------EKHLLNNICEDN-

cy3 :---------------------------------------------------------------------------------------EKHLLQNICADN-

cy4 :---------------------------------------------------------------------------------------DKYRLLNICGEG-

cy5 :---------------------------------------------------------------------------------------EKHLLQNICAEN-

fi3 :---------------------------------------------------------------------------------------EKHLLQNICANN-

so3 :---------------------------------------------------------------------------------------EKHLLQNICANN-

in3 :---------------------------------------------------------------------------------------EKHLLQNICADN-

hy3 :---------------------------------------------------------------------------------------EKHLLQNICADN-

co3 :---------------------------------------------------------------------------------------EKHLLQNICAEN-

kn2 :---------------------------------------------------------------------------------------EKHLLHNICANN-

go3 :---------------------------------------------------------------------------------------DKKNLHNTCEKG-

vi6 :---------------------------------------------------------------------------------------NQEKLKNKCQPGK

cy6 :---------------------------------------------------------------------------------------DQKKLKNKCQPGK

fi4 :---------------------------------------------------------------------------------------NQEKLKNKCQTGK

so4 :---------------------------------------------------------------------------------------DQEKLKNKCEPGK

fr1 :---------------------------------------------------------------------------------------NPEKLKNKCQPGK

go4 :---------------------------------------------------------------------------------------NPENLINKCDKEN

vi7 :---------------------------------------------------------------------------------------PNETLQNNCAEG-

vi8 :---------------------------------------------------------------------------------------EGETLTNVCEGSS

cy7 :---------------------------------------------------------------------------------------GGETLINKCEGNP

fi5 :---------------------------------------------------------------------------------------EGQTLINKCGGSS

so5 :---------------------------------------------------------------------------------------EGQKLLNKCEGSS

in4 :---------------------------------------------------------------------------------------EDQELINKCEKSS

hy4 :---------------------------------------------------------------------------------------EKEELLNKCENSS

co4 :---------------------------------------------------------------------------------------GGKTMVNKCEGDF

kn3 :---------------------------------------------------------------------------------------EGQEMNNKCGNDP

fr2 :---------------------------------------------------------------------------------------DGKTMTNKCDNDP

go5 :---------------------------------------------------------------------------------------GNDSSTNKCQGST

vi9 :---------------------------------------------------------------------------------------IQDNGKNKCEDK-

cy8 :---------------------------------------------------------------------------------------VGENNTNKCDDG-

fi6 :---------------------------------------------------------------------------------------KEPNEKNICDVG-

so6 :---------------------------------------------------------------------------------------NEQDEKNKCKEG-

go6 :---------------------------------------------------------------------------------------NKNDIKNVCENN-

ma1 :---------------------------------------------------------------------------------------KIDKLQNRCADGK

ma2 :---------------------------------------------------------------------------------------TEEQSKNKCHDKY

ma3 :---------------------------------------------------------------------------------------EKSNLHNKCAHNK

ma4 :---------------------------------------------------------------------------------------NKDNLKNTCTNGK

ma5 :---------------------------------------------------------------------------------------DKGTLKNKCSEDK

ma6 :---------------------------------------------------------------------------------------EKEELQNKCGQDS

ma7 :---------------------------------------------------------------------------------------TFEYSKNECAGGK

ov1 :ERTTDEPNIIEIKSALLRDYNGVKVTGPCKAVFQMFLVPHITVNVETNKNSITLGPKLVQAHKKERVTTDGVDAYTEHIEKGLMFEKEEKKLLNKCADGK

ov2 :---------------------------------------------------------------------------------------NKDKFQNECDADG

ov3 :---------------------------------------------------------------------------------------ENDKLSNQCAINE

ov4 :---------------------------------------------------------------------------------------SGKKSKNKCTSVN

yo1 :---------------------------------------------------------------------------------------EDDTLQNQCADNK

be1 :---------------------------------------------------------------------------------------EGDTLQNQCGDNK

ch1 :---------------------------------------------------------------------------------------EGDTLQNVCGENK

yo2 :---------------------------------------------------------------------------------------KESPMKNLCYDDD

be2 :---------------------------------------------------------------------------------------EESPLKNKCHEND

ch2 :---------------------------------------------------------------------------------------GQTPMGNVCHEED

fa1 :FNVGVSRDTL-----------------------------------------------------------------------------DKDRLYNICAENK

re1 :FNVGVSRDTL-----------------------------------------------------------------------------DKDRLYNICTENK

fa2 :---------------------------------------------------------------------------------------DKSNLKNKCDVNK

re2 :---------------------------------------------------------------------------------------DKSKLKNKCDVNK

fa3 :---------------------------------------------------------------------------------------DKSMLKNKCESGK

fa4 :---------------------------------------------------------------------------------------DKTQLKKFCVKDD

re4 :---------------------------------------------------------------------------------------DKTQLKNFCLEND

fa5 :GTTGEQGSSTGTVRGDTEPISDSSSSSSSSSSSSSSSSSSSSSSSSSSS------------ESLPANGPDSPTVK------------PPRNLQNICETGK

re5 :GTTSEQNSGTGTVQGNEESSSDSSSSSSSSSEPSSDSSSSSSSSSSSSSSSSSSSSSSSGSVNTPANGASSNPDA------------KKRNLNNICAPGK

fa9 :---------------------------------------------------------------------------------------GIDLLKNVCETGK

re9 :---------------------------------------------------------------------------------------GIDLLKNVCETGK

ga1 :---------------------------------------------------------------------------------------NIN-FTKKCQNEQ

vi10 :---------------------------------------------------------------------------------------YADDIVNKCEEGK

cy9 :---------------------------------------------------------------------------------------YSDDMVNKCEEGK

fi7 :---------------------------------------------------------------------------------------YADDIVNKCEEGR

so7 :---------------------------------------------------------------------------------------YADDIVNKCEEGK

in5 :---------------------------------------------------------------------------------------YADDVVNKCEEGK

hy5 :---------------------------------------------------------------------------------------YADDVVNKCEEGK

co5 :---------------------------------------------------------------------------------------YADDIVNKCEEGK

kn4 :---------------------------------------------------------------------------------------YADNMVNKCEEGK

fr3 :---------------------------------------------------------------------------------------YADDIVNKCAEGK

go7 :---------------------------------------------------------------------------------------YAEDIINKCESGK

ma8 :---------------------------------------------------------------------------------------HADDIKNKCKTGH

ov5 :---------------------------------------------------------------------------------------YAEDIVHKCAEGK

yo3 :---------------------------------------------------------------------------------------HQHPIKNKCAPGK

be3 :---------------------------------------------------------------------------------------HQHPIRNKCAPGK

ch3 :---------------------------------------------------------------------------------------HQHPIRNKCVPGK

fa6 :---------------------------------------------------------------------------------------HKGELQNKCSEGY

re6 :---------------------------------------------------------------------------------------HTGALQNKCSEGY

vi11 :---------------------------------------------------------------------------------------ELGNLSNDCKKGA

cy10 :---------------------------------------------------------------------------------------DLGNLINDCKKGA

fi8 :---------------------------------------------------------------------------------------DLGNLINDCKKGA

so8 :---------------------------------------------------------------------------------------DLGNLINDCKKGA

in6 :---------------------------------------------------------------------------------------DLGNLMNNCKKGA

hy6 :---------------------------------------------------------------------------------------DIGNLMNDCQKGA

co6 :---------------------------------------------------------------------------------------DLGNLINDCKKGT

kn5 :---------------------------------------------------------------------------------------DLGNLINDCKKGA

fr4 :---------------------------------------------------------------------------------------DVGNLMNDCKNGS

go8 :---------------------------------------------------------------------------------------DLDNLTNDCKKGT

ma9 :---------------------------------------------------------------------------------------NITQLRNKCINKS

ov6 :---------------------------------------------------------------------------------------NVSALNNKCSNEG

yo4 :---------------------------------------------------------------------------------------NLNNLNNKCNDKK

be4 :---------------------------------------------------------------------------------------NLNNLNNKCNDKK

ch4 :---------------------------------------------------------------------------------------NLNSLNNKCDNKK

fa7 :---------------------------------------------------------------------------------------DINDLTNKCNKDS

re7 :---------------------------------------------------------------------------------------DINDLTNKCNKDS

vi12 :----------------------------------------------------------------------------------------------------

cy11 :----------------------------------------------------------------------------------------------------

fi9 :----------------------------------------------------------------------------------------------------

so9 :----------------------------------------------------------------------------------------------------

in7 :----------------------------------------------------------------------------------------------------

hy7 :----------------------------------------------------------------------------------------------------

co8 :----------------------------------------------------------------------------------------------------

kn6 :----------------------------------------------------------------------------------------------------

fr5 :----------------------------------------------------------------------------------------------------

go9 :----------------------------------------------------------------------------------------------------

ma10 :----------------------------------------------------------------------------------------------------

ov7 :----------------------------------------------------------------------------------------------------

yo5 :----------------------------------------------------------------------------------------------------

be5 :----------------------------------------------------------------------------------------------------

ch5 :----------------------------------------------------------------------------------------------------

fa8 :----------------------------------------------------------------------------------------------------

re8 :----------------------------------------------------------------------------------------------------

ga2 :----------------------------------------------------------------------------------------------------

ga3 :----------------------------------------------------------------------------------------------------

392 :-------510-------520-------530-------540-------550-------560-------570-------580-------590-------600

392 :----------------------------------------------------------------------------------------------------

570 :------------##########-#########----------------------------------####################---------#####

vi1 :----------KTFKFVVVIRGE-ELILKWKVYEKVPSPSD----NNKV------------------DVRKYLLKNLGRPITAIQVHSGKEESDVFLLESK

cy1 :----------KAFKFVVIVKGE-ELILKWKVYEREPSATD----NNKV------------------DVRKYVLRNLGSPITSIQVHSGKEDNDVFLLESK

fi1 :----------KTFKFVVIVKGE-ELILKWKVYEKVPSAKE----NNKI------------------DVRKYVLKNLGSPITAIQVHSGKEDNDVFLLESK

so1 :----------KTFKFVVIVKGE-ELILKWKVYEKVPSATD----NNKI------------------DVRKYVLKNLGSPISAIQVHSGKEDNDVFLLESK

in1 :----------KTFKFVVIVKGE-ELILKWKVYEKVPSATD----NNKT------------------DVRKYMLKNLGSPITAIQVHSGKENNDVFLLESK

hy1 :----------KTFKFVVIVKGE-ELILKWKVYEKVPSATD----NNKI------------------DVRKYMLKNLTGPITAIQVHSGKENNDVFMLESK

co1 :----------RTFKFVVIIKGK-ELILKWKVYEKMPSTTE----NNKV------------------NVRTFLLKNTDQPITAIQVHTAKGNEDSFLLESK

kn1 :----------RTFKFVVIIKGK-ELILKWKVYEEIPSATE----NTKV------------------DVRKYALKNLGLPITAIQVHSGKEDSNLFLLKSK

vi2 :----------KTFKFVVVVKGE-ELILKWKVYEKVPSPSD----NNKV------------------DVRTFLLKNTDRPITAIQVHTAKGNEDSFLLESK

cy2 :----------KTFKFVVIVKGE-ELILKWKVYEKEPSATD----NNKV------------------DVRTFLLKNTDRPITAIQVHTAKGNEESFLLESK

fi2 :----------KTFKFVVIVKGE-ELIIKWKVYEKVPSATD----NNKV------------------DVRTFLLKNTDRPITSIQVHTAKGNEDSFLLESK

so2 :----------KTFKFVVIVKGE-ELILKWKVYEKVPSAKD----DNKV------------------DVRTFLLKNTDRPITAIQVHTAKGNEDSFLLESK

in2 :----------KTFKFVVIVKGE-EIILKWKVYEKGPSETD----NDKV------------------DVRTFVLKNTDRPITAIQVHTAKGNEDSFLLESK

hy2 :----------KTFKFVVIVKGE-EIILKWKVYEKGPPETD----NDKI------------------DVRTFVLKNTDRPITAIQVHTAKGNEDSFLLESK

co2 :----------RTFKFVVIIKGK-ELILKWKVYEKMPSTTE----NNKV------------------DVRTFLLKNTDKPITAIQVHTAKGNEDSFLLESK

go1 :----------KTFKFVVSVNGM-ELVLKWKVYEKDTSETS----NDKV------------------DVRTYLIKNLGHPITAIQLHSAKGNNDSFLLESK

go2 :----------KTFKLVLYIYDD-KLTLKWKVYDASPGTTTT---DNKI------------------DMQTFILRNMDHPITSIQIHNVIEKGNSHVLESK

vi3 :----------KTFKLVLYMYDG-VLTIKWKVYPLNGDKTA----DRTL------------------DIRKYKMKDIGQPITSMQVVVMTEQNKTVYVESK

vi4 :----------KTFKLVVYMYED-VLTIKWKVYAPKGVPA-----DKTL------------------DVRKYKMRDIGRPITSIQVLATSKNDETVLVESK

vi5 :----------KTFKLVVYMYEG-ELTIKWKVYPPKGETSS----DKTL------------------DIRKYKMKDIGQPITSMQVVVMSELNKTIYMESK

cy3 :----------KTFKLVLYIYEG-ELTIKWKVYPPKGESTN----DKMV------------------DIRKYKMKDIGQPITSMQVMVMTEKHKTIYVESK

cy4 :----------KTFKLVVFMHED-VLTIKWKVYPPKGVTAS----DKTL------------------DVRKYKMRDIGRPITSIQVLVTSKNDKTLLVESK

cy5 :----------KSFKLVLYIYEG-ELTIKWKVYPPKGESTN----DKTV------------------DIRKYKMKDIGQPITSMQVMVMTEKHKTIYVESK

fi3 :----------KTFKLVLYIHEG-VLTIKWKVYPPEGETIS----DNTV------------------DIRKYKMKNIGQPITSLQVMMMTEKDKTLYVESK

so3 :----------KTFKLVLYVHEG-VLTIKWKVYPPEGETAS----DNTV------------------DIRKYKMKKIDQPITSMQVMMLTEKDKTLYVESK

in3 :----------KTFKLVLYLDDA-ELIIKWKVYPAKGRSIY----DRTV------------------DIRKYKMKDIGQPITSMQVMVMTEQDKTIFVESK

hy3 :----------KTFKLVLYLHED-ELIIKWKVYPAKGRSVY----DRTV------------------DIRKYRMKDIGQPITSIQVVVATQEDQTLYLESK

co3 :----------KTFKLVLYVDKD-VLTIKWKVYPAEGEAIPPAGSDNTV------------------DIRKYKMKDIGQPITSMQVMMVAEQDGTMYVESK

kn2 :----------KTFKLVLYVYEE-VLTIKWKVYPAEGGEGS----DKKV------------------DIRKYKMKDIGQPITSIQVMMVTERDGTIYVESK

go3 :----------KTFKLVIFIYDG-VLTIKWKVYPPQNEQDT----DKTL------------------DIRKYKMRDSGMPITSLQVQVVTKQNETLYVESK

vi6 :ENE-------DTFKFLVFIHEG-ELTLKWKVYNKSAPADA----SKEV------------------DVRKYTLKNLDQPITTVQVRSSKVDEETLLVESK

cy6 :ENE-------QTFKFLVLIHEGGELTLKWKVYDKSAPADA----SNEV------------------DVRKYILKNLDQPITTIQVHSSKVNDERLLVESK

fi4 :ENE-------QTFKFLVFIHEG-ELTLKWKVYDKSAPPNA----SEEV------------------DVRKYILKNLDQPITTIQVRSSKVNDETLLVESK

so4 :ENE-------QTFKFLVFIHEG-ELTLKWKVYDKSAPENA----SEEV------------------DVRKYILKNLDQPITTIQVRSSKVNDETLLVESK

fr1 :GNE-------QTFKFLVLIHEG-ELTLKWKVYDKSAPPRR----SKDV------------------DVRTYILNNMDQPITTIQVHSSKVNDDTLLVETK

go4 :KKD-------QTFKFVVFYDEE-ELTLKWKVYDNFATGNT----NKEV------------------DVRKFLIKNLDRPFTTIQVHAAKVNEDTILVESK

vi7 :----------KNFKFVLYVHED-QLIVKWKVYNKTPAATD----NKKV------------------DVRKYLVKNLKRPITTIHVHSSSVNQTTFLLESK

vi8 :E---------KTFKFIMHLEDS-ILTLKWIVNPQSTTAN-----KNKA------------------DVKKYKVPELERPITSIQVHSVLIQEDTVIYKSK

cy7 :E---------KTFMFIVYLEDN-ILTLKWIVYPQSEGEN-----KNKA------------------DVRKYRLPNLERPITSIQVHSVMVQEDTVIYTSK

fi5 :E---------KTYKFIVYLEDN-ILTLKWLVNPSSDEA------NNQA------------------DVRKYRLPNLERPITSIQVHSVMVQEDTVIYTSK

so5 :E---------KTYKFIVYLEDN-ILTLKWIVYPTSGNA------NNQA------------------EVRKYRLPNLERPITSIQVHSAIVQGGTVIYTSK

in4 :GDSSINSPQ-KTFKFIVHLEDN-ILTLKWIVYPTSDGDN-----KNKA------------------DVRKYRLPKLETPVTSIQVHEGVVHEGTAVYTSK

hy4 :E---------KTFKFIVHLEDN-ILTLKWIVYPPTNEEN-----KNKA------------------DVRKYRLPKLERPVTSIQVHEGIVEGDTIVYTSK

co4 :Q---------KTFKFIAYLEDN-ILTLKWIVYPQGTNEN-----NKVA------------------DVRKYKLPNLERPITSIQVHSLMLQGDSVIYTSK

kn3 :K---------KTYKFIAYLDDN-ILTLKWVVYPPNDDGE-----KKVP------------------DVKKYKLPNLERPITSIQVHSLMLQGDNIIYTSK

fr2 :E---------KTFKFIVYLEDN-ILTLKWIVYPLSESN------NKKA------------------DMKKYKLPNLETPITSIQVHSVMLQDDTVIYTSK

go5 :T---------KTFKLILHLEDN-ILTLKWVVYDQGNGTSSST--SKQA------------------DVKKYRLPKLERPITSIQVHSVIVQGDKVFYKSK

vi9 :----------KTFKFVALIGED-HLTIKWKVYDPAVKTPTP---NEKV------------------EMRKYVMKNLSGEFTAVQVHTVIQQNGSNVFESK

cy8 :----------KTFKFVALIGDD-HLTIKWKVYDPKVQTPPQ---DDTA------------------EMRKYVMKSLNGEFTSVQVHTVIQQNGSNVFESK

fi6 :----------KTFKFVALIGDD-HLTIKWKVYDSAIQIPSP---NDEV------------------EVRKYVMKNLNGEFTSVQVHTVIQQNGSNVFESK

so6 :----------STFKFVAFIGDD-HLTIKWKVYDSAVQTPSS---DDQV------------------EVKKYVMKNLSGEFTSVQVHTVIQQNGSNVFESK

go6 :----------KKFKFVTLIDDD-NLILKWKVYGPNDKIPNP---AEKV------------------EVRKYKIKNLSGVFTAVQVHTALQQNGSTVFESK

ma1 :T-----------FKFIAYLQGE-ELTLKWKVYDNTTPPNK----GSKT------------------DIMNYLIKNLDRPITAIQVHTTKNIQSSFLIESK

ma2 :T-----------FKLIVNIHDN-ILTLKWKVYDKVNNPPNT---DNKV------------------DIRKFMIRNIDQPITSIQIFNVLEKKDVHILESK

ma3 :T-----------FKVVVYIEEN-ILTLKWKVYPSTTETGK----NKMV------------------DVRKYRMKDIGRPITSIQVITSSNPEKQILIESK

ma4 :T-----------FKFVVYTEGN-TLTLKWMVYDTEIAAST----KKNV------------------DMRKYLMKNLDRPITAIQVYSSEANAKTFLVESK

ma5 :TKT---------FKFVAYIKDN-ILTLKWKVYPPKPAPSE----DNIV------------------DVRKFRIPTLDVPISSIQVHTWIGSNRSYILESK

ma6 :KKT---------FKFIIYIQQD-VLTLKWKVYPSEPSSDAS---NIKA------------------DVRKYKIPKLETPITSIQVHTAAVEEGKAFLKSK

ma7 :T-----------FKFVALIQEN-KLTLKWKVYDAPNQNKTT---DDMA------------------DIKRYIIRNVLSPFTAIQVHTVLKHNDEVIMESK

ov1 :S-----------FKFVLFIEGN-KLTVKWKVYDATEAENS----NKQV------------------DVRTYLMKSMDRPFTSIQIHSASISTSTFLLESK

ov2 :NKK---------FKLVVFINND-ILTLRWIVYEGQQLPREPT--GAKL------------------DIKKFRIPTVNVPITSIQVHTWVGSNRSYILESK

ov3 :SSK---------FTFIVYIYKG-LLTLKWKVHTTISSQNTD---KTKA------------------DTRTYVMPPMEMPITSIQVHTFLVEGETVFLKSK

ov4 :STNDSSGSNNKTFKFVAYIDDD-SLTLIWKVYDASSANTT----EDKA------------------DVKKFFIRNVQGPITAIQVHNVVSHDETDYFESK

yo1 :T-----------FKIVIYLDNG-VLHVKWKVYDPSSETEPNTNV----------------------DVRKYRLKNIETPITSIQVHNVKRTLKNITIESK

be1 :T-----------FKIVVYLDSG-VLYVKWKVYDSSTETEPNTNV----------------------DVRKYQLKNLETPITSIQVHTVKRTLKNVTLESK

ch1 :T-----------FKIVVYFDGD-VLYVKWKVYDPSVQTEPNTNV----------------------DVRKYRLKNFETPITSIQVHSIKGGSKTVTFETK

yo2 :-HLY--------YKMLVYLDQD-VLTIKWTVFPIDPGMGKCELCANHNSEN-NV------------DVKKFRIPKLETPFTTIQVHSLLTKENQVVIKSK

be2 :DHLY--------YKILVYLEGD-ILTIKWTTFSINPETNKCELCTNRNNENTNV------------DVKKFRIPKLETPFTTIQIHSLLTKENKIVVKTK

ch2 :DSLR--------YKMLVYLQDD-ILTIKWSVFPSNPESNSCKYCTGHTNESEHV------------DVKKFRLPRLPTPLTTIQVHSLLNKEDKVIIRSK

fa1 :T-----------FKFVVYIKDN-ILTLKWKVYETGVTNNKV-------------------------DIRQYKMKELTRPITTIQIHSVSENKDTHLLESK

re1 :T-----------FKFVVYFKDN-VLTLKWKVYETGVTNNKV-------------------------DIRQYKMKELTRPITTIQIHSVSENKDTHLLESK

fa2 :KQS---------FKFILYLQHD-LITIKWKVYEEKPDTTTRIAPGQVDLNV---------------DVKRYKLPKLDQPVISIQIHSLAQDGETYLMESK

re2 :KQS---------FKFILYLQHD-LITIKWKVYEEKPDTTTTID--QVDLNV---------------DVKRYKLPKLDQPVISIQIHTLAQDGETYLMESK

fa3 :NQT---------FKFVLYFKDD-ILTIKWKVYEEKSATPQKSEENTV-------------------DIKLYKLPKLDQTITSIQVHTLSIEGTSYLMESK

fa4 :TKK---------FDFIAYIYKD-ILVFKWKVYEEGLSKEQDV------------------------DEMKYLLPNLKQPITSIQVHSWTGTKESYILESK

re4 :TKK---------FNFIAYIYKD-ILVFKWKVYTEGPSTTQEI------------------------DEMKYLLPNLKQPITSIQVHSWTGTKESYILESK

fa5 :N-----------FKLVVYIKEN-TLILKWKVYGETKDTTENNKV----------------------DVRKYLINEKETPFTNILIHAYKEHNGTNLIESK

re5 :T-----------SNFVVYIKEN-TLILKWKVYEDPNDTSENNKV----------------------DVRKYLINEKETPFTSILVHAYKEHNGTNLIESK

fa9 :N-----------FKFVAYIKGD-ELIIKWKVIEEGNIPI---------------------------DIKKFKIKRMSPPITSIQVFSSMSDEKSFVLENK

re9 :N-----------FKFVAYIKGD-ELIIKWKVIKEENIPI---------------------------DVKKFKIKTMRPPITSIQVFSSMSDEKSFVLENK

ga1 :T-----------FKFIAVIKDD-ILTLKWEVTDSTEPKK---------------------------EMKKYRIPKFEKPITSIQVHT--GNSTANIIESK

vi10 :N-----------FKLIVYLKGN-ILTLKWKVLPSSDH------------SAAGTKA----------DVRKFKLPQLERPFSSIQVYT--ADVKAGLIESK

cy9 :N-----------FKLILYLKDN-ILTLKWKVLPSSAEDH----------SEDTTKA----------DVRKFKIPQLERPFTSIQVYT--ADVKAGLIESK

fi7 :N-----------FKLILYLKDN-ILTLKWKVLPSSAADSSEEDSSAVDHSAATTKA----------DVRKFKIPQLERPFTSIQVYT--ADVKSGVIESK

so7 :N-----------FKLILYLKDN-ILTLKWKVLPSSAEDSSEEDH-----SVATTKA----------DVRKFKIPQLERPFTSIQVYT--ADVKSGVIESK

in5 :N-----------FKLILYLKDN-VLTIKWKVLPSSATNP----------SAASTKA----------DVRKFKIPQLERPFTSIQVYT--ADVKAGFIESK

hy5 :N-----------FKLVLYLKDN-VLTLKWKVLPSSAEDA----------SAATTKA----------DVRKFKIPQLERPFTSIQVYT--ADVKAGFIETK

co5 :N-----------FKLIVYLKEN-ILTLKWNVFPSTEEDH----------SDVTTKA----------DVRRFKIPQLERPFTSIQVYT--ADVKAGVIESK

kn4 :N-----------FKLILYLKDN-ILTLKWNVLPSLSADD----------SDSTTKA----------DVRKYKLPSLERPFTSIQVYT--ADAKEGIIETK

fr3 :N-----------FKLILYLKDN-ILTLKWNVLPSSEEND----------SATNTKA----------DVRKFKIPQLERPFTSIQVYT--ADAKAGIIESK

go7 :T-----------FKLILYLKNN-ILTLKWKVNPPPAAVG----------HKVHTKE----------DVRRFKIPQLDRPFTSIQVYT--ADVKSEIIETK

ma8 :T-----------FKFIIYLTDN-ILTLKWKVYPPVSSTTTVKV-----------------------DIRKYRVPKLDRPFTAIQVYT--ANVKAGLIESK

ov5 :T-----------FKLVAYIKDD-MLMLKWKVSPPPPDTPANMKA----------------------DVRKFRIPQLDRPFTSIQVYK--ANVKEGIIESK

yo3 :A-----------FAFISYIKDN-ILTLKWKVFTPPSDLFANEVVKEKILSTVPEEPITEDESPVLVDVRKYRLPPLERPFTSIQVYK--ANTKQGLLETK

be3 :A-----------FAFISYVKDN-ILILKWKVFAPPSDLFANDEVSKQILSAVSVTSDAE---SSVVDVRKYRLPQLDRPFTSIQVYK--ANPKQGLLETK

ch3 :A-----------FAFISYVKDN-ILTLKWKVFVPPADLFANDVVSKQILSSVSEEPSS----PQVVDVRKYRLPQLDRPFTSIQVYK--ANTKEGILETK

fa6 :H-----------FKLVVYITHN-VLNLKWKTYKPNEESKSEDS-----------------------DVRKYRIPKLERPFTSIQVYT--ANSKAGVIETK

re6 :H-----------FKLVVYITHN-VLTLKWKTYKPNEESKSKVS-----------------------DVRKYRIPTLERPFTSIQVYT--ANSKAGVIETK

vi11 :NNS---------FKLIVHTSDD-ILTLKWKVTGEGAAPGNKA------------------------DVKKYKLPTLERPFTSVQVHS--ANAKSKIIESK

cy10 :NNN---------FKFVVHTSDD-ILTVKWKVTGEGAAPGNQE------------------------DVKKYKLPPLDRPFTSVQVHS--ANAKSKIIESK

fi8 :NNN---------FKFVVHISDD-ILTLKWKVNGEGAAPGNKE------------------------DVKKYKLPSLERPFTSVQVHS--ANAKSKIIESK

so8 :NNN---------FKFVVHISDD-ILTLKWKVTGEGAAPGNKE------------------------DVKKYKLPSLERPFTSVQVHS--ANAKLKIIESK

in6 :NSN---------FKFVVHTRDD-ILTLKWKVTGEGEVPGNKE------------------------DVKKYKLPSLDRPFTSVQVHS--ANTKSKIIESK

hy6 :NSN---------FKLVVRTRDD-ILKIKWKVTGEGAAPGNKE------------------------DVKKYKLPSLDRPFTSVQVHS--ANTKAKIIESK

co6 :NNK---------FKFVVHTSDD-ILTLKWKVTGEGAAPGKDNKE----------------------DVKKYKLPPLERPFTSVQVHS--ANAKSKIIESK

kn5 :NNN---------FKFVVHTRDN-ILTLKWKVTGKGESTGNEE------------------------DVKKYKLPSLERPFTSIQVHS--ANAKSKIIESK

fr4 :NNN---------FKFVVYTDDN-MLTLKWQVVGEGAAPGKKE------------------------DVKKYKLPTLDRPFTSVQVHS--ANAKSKIIESK

go8 :KDT---------FKLIIHTHDN-NLTLKWKVNGEGAGEGNKA------------------------DEKTYKLPSLERPFTSIQVHS--VNSKSKILESK

ma9 :NST---------FKFIAYVKDQ-ILTLKWLVIGEGADAGNKV------------------------DVKKYKIPVLDKTFSSVQVHT--ANAEKNLIESK

ov6 :STT---------FNFVVYIEGD-VLTLKWRVNSDRLIEGENGNTA---------------------DVRKYKIPTNDSPLTAIQVYT--ANVKDKLIESK

yo4 :NDT---------FKFTAKIEED-ILKIKWKTYTNETDQEPKS------------------------DIKKFRLPDLAKDLTSIQIFT--TNTKENIIESK

be4 :NDT---------FKLTAKIEED-ILKIKWETHTNETDQNPKR------------------------DIKKFRLPDLSKGLTSIQIFT--ANAKENIIESK

ch4 :NDT---------FKFSAKIKDD-ILKIKWKTQTNETDQGHKK------------------------DVKQFRLPDLSKEPTSIQIFT--ANAEEKIIESK

fa7 :NKY---------FKLVLYIKNN-ILILKWKVQDKDSKPTNIDV-----------------------DVKKYKIPKLDRPFTSIQVYT--VNTEHGLIESK

re7 :NQH---------FKLVLYIKNN-ILTLKWKVQDEDSKPTNIDV-----------------------DVKKYKIPKLDRPFTSIQVYT--VNTEHGLIESK

vi12 :-------------------------------------------------------------------MKINQVAQYVLF-ALVA-HLVKRVKTNILVNCF

cy11 :-------------------------------------------------------------------MKINQIAQYVLL-VLVA-HLVKRVKSNIIVNCF

fi9 :-------------------------------------------------------------------MKINQIAQYILF-ALVA-HLVKRVQSNILVNCF

so9 :-------------------------------------------------------------------MKINQIAQYVLF-ALVA-HLVKRVKSNILVNCF

in7 :-------------------------------------------------------------------MKINQIVQCVLF-ALVA-HLVKRVKSNILVNCF

hy7 :-------------------------------------------------------------------MKINQIAQYVLF-ALVA-HLAKRVQSNILVNCF

co8 :-------------------------------------------------------------------MKISQIAQYVVF-ALVA-HLVKRVKSNILVNCF

kn6 :-------------------------------------------------------------------MKINQIAQYVVF-TLVA-HLVKRVKSNIIINCF

fr5 :-------------------------------------------------------------------MKINQIVQYILF-ALVA-HVVKRVKSNIIVNCF

go9 :-------------------------------------------------------------------MKANEIKYYLLF-TFVV-HLVKRVNSNILVNCF

ma10 :-------------------------------------------------------------------MKISKIKYYLFF-FFIA-HLVKTVKSRVLVNCF

ov7 :-------------------------------------------------------------------MKKATYLMLLLLFALLA----ERVKSKILVNCF

yo5 :-------------------------------------------------------------------MKTYKIKYFLLI-SFFI-NLIKQAKSKIIISCF

be5 :-------------------------------------------------------------------MKTYKIKYFLLL-SLFI-NLINQAKSKIIISCF

ch5 :-------------------------------------------------------------------MKTYKIKYFLLI-SLFI-NLIKQAKSKIVISCF

fa8 :------------------------------------------------------------------------------------MFSLCTVHSNMLINCF

re8 :------------------------------------------------------------------------------------MFSLCTVHSNMLINCF

ga2 :-------------------------------------------------------------------MI-----YYAFF--IFLYSFGKRXKSNLTISCH

ga3 :-------------------------------------------------------------------ME--KIKIINYILLLFLIFITKKVKSNLVITCS

392 :-------610-------620-------630-------640-------650-------660-------670-------680-------690-------700

392 :-----------######################-------------------------------------------------------------------

570 :#####################################--------#######------------------------------------------------

vi1 :AYLLRQDIPRTCERIASSCFLSGNVDIEGCYKCTLLSEDTK-LGSPCFSYLPPEV-KH------------------------------------------

cy1 :AYLLRQDIPKTCERIATNCFLSGNVDIEGCFKCTLLWENTK-LGSPCFSYLPPDV-KH------------------------------------------

fi1 :AYLLRQDIPNTCERIATNCFLSGKVDIEGCYKCTLLSENTE-LGSPCFSYLPPDV-KH------------------------------------------

so1 :AYLLRQDIPSTCERIATNCFLSGKVDIEGCYKCTLLSENTK-LGSPCFSYLPPDV-KH------------------------------------------

in1 :AYFLRQDIPNTCERIATNCFLSGNVDIEGCYKCTLLSENTE-LSNRCFSYLPPDV-KR------------------------------------------

hy1 :AYFLRQDIPDECEDIATNCFLSGNVDIEGCYKCTLLSENTE-LSNRCFNYLPPDV-KR------------------------------------------

co1 :SYFLKDDMPAKCDLIARNCFLSGNLDIEGCYKCTLLSENTE-LSSPCFNYLPPDF-KQ------------------------------------------

kn1 :AYFLRKDIPRTCERIVTSCYLSGNVDIHKCFQFTLLMENHD-TMNECSKYVSSEV-TD------------------------------------------

vi2 :EYILADDMPAQCDLIAANCFLSGSLDIEGCYKCALLSENAE-LSSPCFDYLSPDV-KN------------------------------------------

cy2 :SYFLIDDMPAKCGLIATNCFLSGSLDIEGCYKCTILSENTE-LDSPCFSYLPPDV-KH------------------------------------------

fi2 :LYFLTNDMPDKCDLIATNCFLSGNLDIEGCYKCTLLSENTE-VGSPCFSYLPPDF-KD------------------------------------------

so2 :SYFLMNDMPAKCDLIATNCFLSGNLDIEECYKCTLLSENTE-LGGPCFSYLTPDF-KD------------------------------------------

in2 :SYFLKDDMPAKCDLIATNCFLSGNLDIEACYKCTLLSENTE-LDSPCFSYLPDDV-QH------------------------------------------

hy2 :SYFLKDDMADKCDVIARNCFLSGNFDIEACYKCSLLSENTE-LDSPCFSYLSDDF-KH------------------------------------------

co2 :SYFLKDDMPPKCDLIARNCFLSGNLDIEGCYKCTLLYENTE-LNSPCFNYLSPDF-KQ------------------------------------------

go1 :IYILKNDIPEKCDLIATGCFLSGNIDIESCYKCALLFQNTD-PTSPCFNYISPDD-KE------------------------------------------

go2 :NYNLGNTFPEKCAAYANNCFLSGITDIEKCYKCTLLVKEVE-KSDVCYNYILKKE-NT------------------------------------------

vi3 :NFSIMNEIPEKCDAIANECFMSGVLDVQKCYHCSLLLQKKE-SAQECFKFVSPKI-KN------------------------------------------

vi4 :NYSVMEEIPEKCDAIANECFLSGVLDVQKCYHCTLLLEKKE-NAQECFKFISPEI-RD------------------------------------------

vi5 :NFAVMNQIPEKCDAIANECFLSGVLDVQKCYHCTLLLQKKE-NAEECFKFVSPTI-KN------------------------------------------

cy3 :NFSVMNDIPEKCDAIANECFMSGVLDIQKCYHCTLLLQKKE-SAQECFNFVSPEI-RS------------------------------------------

cy4 :NYSVMEEIPEKCDAIANDCFMSGVLDIQKCYHCTLLLQKKK-NAEECFKFVSPEI-KN------------------------------------------

cy5 :NFSVMNDIPEKCDAIANECFMSGVLDIQKCYHCTLLLQKKD-SAQECFNFVSPEI-RS------------------------------------------

fi3 :NFSVMNDIPEKCEAIANECFMSGVLDIQKCYHCTLLLQTKE-KAQECFKFVSPKI-KN------------------------------------------

so3 :NFSVMNDIPEKCESIANECFMSGVLDIQKCYHCTLLLQTKD-KAQECFKFVSPKI-KN------------------------------------------

in3 :NFSLMNDIPEQCDAIANECFMSGILDVQKCYHCTLLMQKKD-DVQECFKFVSPDI-KN------------------------------------------

hy3 :NFSLVNDIPEQCDAIANECFMSGVLDVQKCYHCTLLMQEKQ-NVQECFKFVAPEI-RN------------------------------------------

co3 :NFSIMNDIPEKCDAIAKECFMSGVLDIQKCYHCTLLLQEKK-KAEECFTYVSPQI-KD------------------------------------------

kn2 :NFSVMNDIPEKCDAIANQCFMSGVLDIQKCYHCTLLLQEKE-KAEECFKFVSTEV-KN------------------------------------------

go3 :NYSVANDIPEKCDALANDCFLSGILDVQKCYHCTLLMQEKQ-NAKECFKYISPEI-RN------------------------------------------

vi6 :SYYVNRDIPDKCDVIATDCYLNGNVDIEKCFQCTLLMENSD-TTNECFKYVSSQV-TD------------------------------------------

cy6 :SYYVKRDIPEKCDTIATACYLNGNVDIEKCFQCTLLMDNND-TTNECFKYVSSEV-TD------------------------------------------

fi4 :SYYVKRDIPEKCDTIATDCYLNGNVDIEKCFQCTLLMKNND-TSDECFKYVSSEI-TD------------------------------------------

so4 :SYNVKRDIPEKCDTIATDCYLNGNIDIEKCFQCTLLMKNND-TTDECFKYVSSEI-TD------------------------------------------

fr1 :SYYVKRDIPEKCDTIATDCYLNGNVDVEKCFQCTLLIQNKD-TTDECFKYVSSEV-TD------------------------------------------

go4 :SYYFKSNIPEKCDVIATDCYLNGNVNIEKCFQCTLLVQNND-ASNECFKYVSSEI-TD------------------------------------------

vi7 :SYALKKDIPDQCDAMVTNCFLSGILDVEKCYHCSLLVSSKD-TSNMCFNYASPDI-KE------------------------------------------

vi8 :DYSVKTDIPEKCEQVASACFLSGNTDIESCYTCNLLIHNED-TSDKCFDYVSSEF-KN------------------------------------------

cy7 :DYSIKNDIPENCQQTMSACFLSGNTDIESCYTCNLLIHNND-TNDKCFDYVSADF-KK------------------------------------------

fi5 :DYSIKNDIPENCQQVISACFLSGNTDIESCYTCNLLMHNDN-TNDKCFDYVSADF-KK------------------------------------------

so5 :DYSIKNDIPENCQQVISACFLSGNTDIESCYTCNLLMHNDN-TNDKCFDYVSEDF-KK------------------------------------------

in4 :DYSLKNDIPEMCQAYMSSCFLSGNTDIESCYTCNLLIHNDN-TNDKCFDYVSADF-KK------------------------------------------

hy4 :DYSLKNDIPELCQGYISSCFLSGNTDIESCYTCNLLIHNDN-TNDKCFDYVSEDF-KN------------------------------------------

co4 :DYSIKNDIPKTCQQVLSACFLSGNTDIENCYTCNLLINKED-TNDKCFDYVSADF-KN------------------------------------------

kn3 :DYSIKNDIPETCEQVLSACFLSGNTDIENCYTCNLLLHKEN-TDDKCFDYVSADF-KK------------------------------------------

fr2 :DYSIKKDIPETCQQIMSACFLSGHIDIENCYTCNLLMYQKD-TNDECFDYVSADF-KN------------------------------------------

go5 :DYSINKEIPEKCEQIASSCFLSGNTDIESCYTCNLLINNDN-VNDECFNYVSKAF-KE------------------------------------------

vi9 :NYALSSGIPEKCDAVATNCFLSGSVYIEKCYRCTLKMKKVD-PSDVCYNYIPKVE-S-------------------------------------------

cy8 :NYALSNDMPEQCDAIATNCFLSGSVYIEKCYRCTLKMKKLN-PSDVCYNYIPKVE-N-------------------------------------------

fi6 :NYALSNDMPEKCDAMASNCFLSGSVYIEKCYRCTLKMKNVN-PSDVCYNYVPKVE-NTPSQESIPAVDNTPSQESIPAVDNTPSQDSIPTVEN-------

so6 :NYALSNDMPEKCDAMASNCFLSGSVYIEKCYRCTLKMKNLN-PSDVCYNYVPKVE-N-------------------------------------------

go6 :NYALSNDIPEKCDAIAANCFLSGNVYIEKCYKCTLKMNDEN-ETDVCYSYVPKDA-K-------------------------------------------

ma1 :NYSITNSVPEKCELMAMNCFLSGSLNMENCYSCAVLLENDNIQNNECFNYTSAFI-KE------------------------------------------

ma2 :NYPLGNVMPDRCDIMATNCFFSGISDIEKCYKCTLLTKNLS-PSDECSKYASKET-DT------------------------------------------

ma3 :NYSISN-MPEKCDAIASDCFFGGIIEIEKCYQCALFLESKE-KASECLNYVSADM-RQ------------------------------------------

ma4 :GYSIKSNMPEMCELIAINCFLSGNIDIDKCYQCTLLTEHAD-KNNECFKYVSSEV-KD------------------------------------------

ma5 :NYAIKKGIPEKCETIATNCFLSGFTDIEKCYKCTLRVRGEQ-HSDVCYKFISKVE-DT------------------------------------------

ma6 :DYSIKNDIPVKCEQIASACFLSGNTDIEKCYTCNLLIQNTP-TSDKCFNYVSSDI-KE------------------------------------------

ma7 :NYSISKAVPEKCNFIASECFLHGSIFIEKCYKCTLKAKNID-ANDECYNYVQKLT-SV------------------------------------------

ov1 :NYTLKKDFPDQCDAIASNCFMSGNVDIEKCYHCTLLIENTD-TSNVCFNYVSPEV-KE------------------------------------------

ov2 :NYAFGNTMPEKCDNIASNCFLSGYTDIEKCYKCTLVYRKME-PSDVCYKYASSVD-AK------------------------------------------

ov3 :DYVIKKDMPLKCENVANNCFLSGNTDIEKCYTCSLLIKDVD-TSDVCFNYVSSEF-KE------------------------------------------

ov4 :NYSISKDIPAKCDLIASNCFLSGSIYIEKCYKCTLQVKDVD-PSDVCYNYVQKVESTPISDSTTSPQGNSGQAGSISEHSSSSEQAASPGQHVSVQPENN

yo1 :NYTINDDLPEKCEAIASDCFLHGNVDTEKCYKCALLYRDTP-ITDKCYDYLPDDY-KE------------------------------------------

be1 :NYTINDNLPEKCEAIASDCFLHGNVDIEKCYKCALLYRKNP-LTDECYNYLSNDY-KK------------------------------------------

ch1 :NYTINDHLPEKCDEIASDCFLHGHAEIEKCYKCALLYQKTP-LTDECYNYVPRDY-KE------------------------------------------

yo2 :DYSLINDMPKKCDKIATKCFLDGKTDIEPCYTCSLLTENFP-RTDECYNYSSPLV-KE------------------------------------------

be2 :DYSLIDDIPKKCDIIATKCFLSGKTDIEPCYTCNLLTENIP-RNDECYNYSSPLV-KE------------------------------------------

ch2 :DYALKNEMPKKCDQVASKCFLNGQTDIEQCYTCSLLTEKIP-TTDECYNYSSPLV-RE------------------------------------------

fa1 :NYVIKTDIPETCDVMATNCFLSGNINIEKCLECTLLVQNND-TSSECFTYVSNDV-RE------------------------------------------

re1 :NYVIKTDIPETCDAMATNCFLSGNINIEKCLECTLLVQNND-TSSECFTYVSNDV-RE------------------------------------------

fa2 :DYNLEDQIPEKCEAIASDCFLSGNVDIEKCLQCTLLVIKAD-KNDECLKYVSKNV-KD------------------------------------------

re2 :DYNLQDQIPEKCEAITSDCFLSGNIDIEKCLQCTLLVTKAD-KNDECLKYVSKNI-KD------------------------------------------

fa3 :DYSLGNNLPEKCDAIASDCFLSGNINVEKCLKCTLKVKKVE-ASDECYKYVSKDKPKETKLAVSG-----------------------------------

fa4 :DYVLGEGMPEKCDAIATDCFLSGFTDIGKCFQCKLLMQEKN-INDSCFKYVSSNQ-KE------------------------------------------

re4 :DYVLGEGMPEKCDAIATDCFLSGFTDIGKCFQCKLLMQEKN-INDSCFKYVSNNQ-KE------------------------------------------

fa5 :NYAIGSDIPEKCDTLASNCFLSGNFNIEKCFQCALLVEKEN-KNDVCYKYLSEDI-VS------------------------------------------

re5 :NYALGSDIPEKCDALASNCFLSGNFNIEKCFQCALLVKPKN-ENDVCYKYLSEDI-IN------------------------------------------

fa9 :NYVVTNNMPEKCEALATNCFLSGNIDIEKCYQCSLLVENDS-KSDVCYNFISREN-KE------------------------------------------

re9 :NYVVTNNMPEKCEALATNCFLSGNIDIEKCYQCSLLVENDS-KSDVCYNFISREN-KE------------------------------------------

ga1 :NYAIKNAIPEKCDAITTECFLSGNLDIAKCFHCTLLARNTD-TSNECFNYVSPDN-KA------------------------------------------

vi10 :NYALGADIPDKCNAIATDCFLNGNVNIDKCFQCALLVQGGDATSNECFNYVSKDL-QD------------------------------------------

cy9 :NYALGADIPEKCNSIATDCFLNGNINIDKCYQCTLLVQNGDPTSNECFNYISKDL-QD------------------------------------------

fi7 :NYALGADIPDKCNSIATDCFINGNVNIDKCFQCTLLVQNGEQTSNECFNYIPKNL-QE------------------------------------------

so7 :NYALGADIPDKCNSIATDCFLNGNVNIDKCFQCTLLVQNGDQTSGECFNYIPKDL-QD------------------------------------------

in5 :NYALEADIPDKCNSIATDCFLNGNVNIDQCYQCTLLIKGVDSTSKECFNYISKDL-QE------------------------------------------

hy5 :NYALGADIPAKCDSIATDCFLNGNVNIDQCYQCSLLVQGADSTSKECFNYISKDL-QE------------------------------------------

co5 :NYALGADIPDKCNSIATDCFLNGNINIDKCYQCSLLVQNSDQTSDECFNYVSKDL-QD------------------------------------------

kn4 :NYALGADIPDKCNAIATDCFLNGNINIDKCYQCTLLVQNSEQNSEECFNYVSKDL-QE------------------------------------------

fr3 :NYALDADIPDKCNAIATDCFLHGNINIDKCYQCTLLVQSADQTSGECFKYVSKDL-QD------------------------------------------

go7 :NYALQTDIPEKCNSIATDCFLNGNVNIEKCYQCTLLIQNSDPSSNECFNYVSKDM-KD------------------------------------------

ma8 :NYAIGSDIPGKCSAIATECFLNGNFNIESCYQCTLLVQNKD-LSDECFKYVSGDM-KS------------------------------------------

ov5 :NYVIASEIPEKCSAIATNCFLNGNLNIENCYQCTLLVQNSD-TSSECFNYVSSDM-KA------------------------------------------

yo3 :NYILKNAIPEKCSKISMDCFLNGNVNIENCFKCTLLVQNAK-PTDECFQYLPSDM-KN------------------------------------------

be3 :NYILKNAIPEKCSKISMNCFLNGNVNIENCFKCTLLVQNAK-PTDECFQYLPSDM-KN------------------------------------------

ch3 :NYVLKNAIPEKCSKISMDCFLNGNVNIENCFKCKLLVQNAK-PSDECFKYLPSDM-KE------------------------------------------

fa6 :NYNIRTDIPDTCDAIATDCFLNGNVNIEKCFQCTLLVQKKD-KSHECFKYVSSEM-KK------------------------------------------

re6 :NYNIGTDIPDTCDTIATDCFLNGNVNIEKCFQCTLLVQKKD-KSHECFKYVSSEM-KK------------------------------------------

vi11 :FYDIGSGMPAQCSAIATNCFLSGSLEIEHCYHCTLLEKKLA-QDSECFKYVSSEA-KE------------------------------------------

cy10 :FYDIGSSMPAQCSVVATNCFLSGSLDIEHCYHCTLLEKKMA-QNSECFKYVSKEA-KE------------------------------------------

fi8 :FYDIGSSMPAQCSAVATNCFLTGSLDIEHCYHCTLLEKKIA-KDSECFKYVSREA-KE------------------------------------------

so8 :FYDIGSSMPAQCSAVATNCFLTGSLDIEHCYHCTLLEKKIA-KDSECFQYVSREA-KE------------------------------------------

in6 :IYDIDRSIPAQCSAVATKCFLSGSLDIEHCYHCTLLENNMA-KDSECFKYLSREA-NE------------------------------------------

hy6 :IYDMDRSIPAQCSAVATKCFLSGSLDIEHCYHCTLLEKKMA-KDSECFKYLSREA-KE------------------------------------------

co6 :FYDIDSSMPAQCDLIARNCFLTGSLDIEHCYHCTLLEKKMT-EGNECFNYVSTEA-KE------------------------------------------

kn5 :FYDLGSSMPAKCDLIAMNCFLKGSLDIEQCYHCTLLEKKMN-EDSECFKYVSIEA-KE------------------------------------------

fr4 :FYDIGSSMPAQCSAIATNCFLTGSLDIEHCYHCTLLEKKMA-EDNECFEYVSKEA-KE------------------------------------------

go8 :FYDIKNSMPDQCSIIATNCFLSGNLDIEKCYHCTLLEKKVD-NDNECFKYVSKDA-QE------------------------------------------

ma9 :NYHISSVIPDQCSIITTNCFLSGSLDIEHCFHCTLLAQKYE-SENECFKYVSEEA-KE------------------------------------------

ov6 :NYDIRSQMPDQCNQIVANCFLRGSLDIEHCYHCTLLANKYS-NDSECFNYVTKEG-KK------------------------------------------

yo4 :TYDIDKNIPEKCSAISASCFLGGSLNIESCYHCTLLAQKYP-NDDECFNYISSEL-KN------------------------------------------

be4 :TYDIDNNIPEKCSVISANCFLGGSLNIESCYHCTLLAQKYP-NDDECFNYISSEL-KN------------------------------------------

ch4 :TYDIDKNIPEKCSAISANCFLGGSLNIESCYHCTLLAQKYP-SDDECFNYISSES-KD------------------------------------------

fa7 :NYDINSEIPEQCEAISTNCFLNGSLDVENCYHCTLLAKKVD-SNNECFNYVSKEA-KE------------------------------------------

re7 :NYDINSEIPEQCEAISTNCFLNGSLDVENCYHCTLLAKKVD-SNNECFNYVSKEG-KE------------------------------------------

vi12 :GMLHC----KICHIAIRNCFLSGTSNLTKCLQCEEKYY----NIQPCTHQTENFL---------------------------------------------

cy11 :GMLHC----RICHTAIRNCFLSGTSNLTKCIQCEEKYY----DIQPCTHHTENFL---------------------------------------------

fi9 :GMLHC----KICHIAIRNCFLSGTSNLTKCIQCEEKYY----NIQPCTHHTENFL---------------------------------------------

so9 :GMLHC----KICHIAIRNCFLSGTSNITKCIQCEEKYY----NIQPCTHHTENFL---------------------------------------------

in7 :GMEHC----KICHTALRNCFLSGTSNLTKCIECEEKYY----KIPPCIHHTENFL---------------------------------------------

hy7 :GIEHC----KICHTALRNCILSGTKNLTKCIECEEKYY----QIRPCTHHSESFL---------------------------------------------

co8 :GTLHC----KICHIAIRNCFLSGTSNLTKCIECEERYY----NIQPCTHHTENFL---------------------------------------------

kn6 :GTLHC----KICHIAIRNCFLSGTNNLTKCIECEEKYY----NIQPCTHHTENFL---------------------------------------------

fr5 :GTLHC----KICHIAIRNCFLSGTSNLTKCIQCEEKYY----NIQPCTHHTENFL---------------------------------------------

go9 :GVLHC----KMCHVAIRNCFLSGTSNMSKCIKCEENYY----NIRPCTHQIENFI---------------------------------------------

ma10 :GLLHC----KICHTIIRNCFLSGTSDLSKCIACEESYY----SIKPCTHHTDEYL---------------------------------------------

ov7 :GMLHC----MVCNTIMRNCFLSGTSNLAKCIACEEKYY----EARPCTHHTENFL---------------------------------------------

yo5 :GKQHC----KICHIIIRNCFLSGTSNLEKCIACEENYY----ETRKCIKQEESFF---------------------------------------------

be5 :GKQHC----KICHIIIRNCFLSGTSNLQKCIACEENYY----NTRTCIKHEESFF---------------------------------------------

ch5 :GKQHC----KICHVIIRNCFLSGTSNLQKCIACEESYY----NTRQCIRQEESLF---------------------------------------------

fa8 :GVLHC----NICRTVLRNCFLSGTSDLQKCISCGEKYY----KISPCTQNHESFL---------------------------------------------

re8 :GVLHC----NICRTVLRNCFLSGTSDLQKCISCGEKYY----KISPCTQNHESFL---------------------------------------------

ga2 :GKYHC----KICHTIMRNCFLAGSLDFSKCILCEEKFY----NIQPCIQHPQNFL---------------------------------------------

ga3 :DTNHC----QICHQILRSCFLSGTSEIAKCVSCEEKHH----NILPCLEYSKEIL---------------------------------------------

392 :-------710-------720-------730-------740-------750-------760-------770-------780-------790-------800

392 :----------------------------------------------------##############----------------------############

570 :----------------------------------------------------##############--------------------##############

vi1 :--------------------NYEKVKMKAQDEGDLG-------------EAQLEELIRRILHAVQRKGKSNPP----------RK-GATEEDYPNDNLRE

cy1 :--------------------NYEQIKMKAQDEGDKE-------------EAQLDEFIRRVLQMVQKMEKNNTP----------RK-GKNKGDYPNDNLKE

fi1 :--------------------NYEQIKMKAQDEGDPE-------------KDQLEEFIRRILQLVQKMEKNNTH----------RK-GERKVEYPNDKLRE

so1 :--------------------NYEKIKMKAQDEGDPE-------------KYQLEEFIRRILQLVQKMGKNNTP----------RK-GETKVHFPNDNFRE

in1 :--------------------NYEQIKIKAQDEGDPE-------------ESQLKEFIGRILQVVQKMGENNTP----------TK-GETKVEYPNGNLKE

hy1 :--------------------NYEQIKIMGQDEGDPE-------------EAELEEFIGRVLEVVPQMGENNTP----------RK-GETEIEYPNDNLKE

co1 :--------------------NYEQIKRKTHDEIDPE-------------AVQLEEFIGRILQVVLKIGENDTP----------TE-GETKVEYPNDDLKE

kn1 :--------------------KFKGIKIEAQDNVDPE-------------EIQLRETIDRILNWIYRI--DEDG----------KK-VLIMQEELDLSMKE

vi2 :--------------------DYEEIKRKAQQQGDLK-------------EVQLAASIGKILQGVFKKGE--AG----------LN-ELVTFDQADAALKE

cy2 :--------------------NYEQIKSKAHQVDQK--------------EVQFAVSIGKILQGVYKKGE--TN----------LN-QLLTFDEADAALKA

fi2 :--------------------NFEQIKRKAQQEGNLK-------------EDPFAVSIGKILQGVYKKGE--TD----------HN-EFISFDQADTALKA

so2 :--------------------NYEQIKRKAQKEGDQK-------------EVQFAMSIGKILQGMYKKGK--TD----------LS-ELLRFDETDTDLKA

in2 :--------------------NYEQIKRKAQQKEDPK-------------EVQFAVSIGNIHQGMYKLGE--TG----------LN-ELLSFDEADTSLKA

hy2 :--------------------NYQQIKRKAQKRDDPK-------------EVQFQVSIGKILQGMYKLGE--TG----------LN-ELLSFDEADTALKE

co2 :--------------------NYEQIKRKTQQEKDPI-------------EVQLATSINKVLQGMYKKGE--MG----------LN-ELLSFDKADTTLKE

go1 :--------------------TFEDIKTKAQDKDDPR-------------EVELEASIYKILKGVYKLDT--NE----------EM-DLINFSKADAALKE

go2 :-------------------ANEEGISTEGEDEDSN--------------MLQLDASIDSILNQIYNTGN--NN----------AK-ELMNISELGNSLKV

vi3 :--------------------SFDEIQAKGEDEENPN-------------EEELQKTIDNIFVKVYKKGE--NL----------YK-EVDQLAIIDSSFKS

vi4 :--------------------RFDDIKTKGEDEEDPN-------------EAELEESIHTILEKMYQN-G--GA----------NN-EVNHLAILDDTLKE

vi5 :--------------------RFEDIQTKGEDEENPN-------------VVELEETIDLLLNKIYKNGE--GE----------SN-EVDQLAIIDSSFQS

cy3 :--------------------RFDDIQAKGEDEENPN-------------EVELEGTIDKIFIKIYKKGE--NH----------YK-EVDQLAIMDSSFKS

cy4 :--------------------RFDDIQVKGEDEQDPN-------------EVELEQSIHNILDKMYNKVE--GG----------KH-EVNHLAILDATFKE

cy5 :--------------------RFDDIQAKGEDEENPN-------------EVELEGAIDKIFIKIYKKGE--NH----------YK-EVDQLAIMDSSFKS

fi3 :--------------------EFNDIQAKGEDEENPN-------------EVELEETIDNIFTKIYKKGE--NL----------YN-EVDQLAIMDSSFQS

so3 :--------------------EFDEIKAKGEDEENPN-------------EIELEETIEKIFIQIYKKGE--NL----------YN-EVDQLAIMDSSFKS

in3 :--------------------RFDDIQTKGEDEEDPN-------------EVKLEETIDDIFIKIYKKGE--NL----------YK-VVDQLAILNSSFKS

hy3 :--------------------RFDDIQTKGENAEDPN-------------EVELEQSIHSIMDKIYKKGE--NL----------YK-EVDQLAILDSSFKS

co3 :--------------------NFEKIQQTKAEDEEDPS------------EAELEKTIHNIFTKIYEEGG--NL----------YK-EVNQLAIMDYSLKY

kn2 :--------------------RFEEIQTKGEDEKNPN-------------EVELEETIDNIFTKIYGGEE--NL----------HK-EVNQLAILDYSLKY

go3 :--------------------RFDDIKTTGQDEDEAD-------------KVELEETIDNILKNIYKNED--GE----------NK-EINNFDLADNSLKQ

vi6 :--------------------KFKDIKVNAQDEEDPA-------------EVELAASIGKILQGVFKKGE--AA----------HI-ELLTFDEVDAALKE

cy6 :--------------------KFKEIKVNAQDEEDPT-------------EVELATSIGKILQGVYKKGE--ND----------IN-ELLTFDEADAALKE

fi4 :--------------------KFNDIKVNAQDEEDPI-------------EAELATSIGKILQGVYKKGE--TD----------HN-ELVNFYEADTALKA

so4 :--------------------KFNDIKVNAQDEEDPI-------------EVELAASIGNILQGVYKKGE--TN----------HN-ELLSFEEADTTLKE

fr1 :--------------------KFQDIKVNAQDEEDTL-------------EDVLTESIGKILQGMYKKGQ--TG----------MN-QLLSFDQANKALKA

go4 :--------------------KFNHIKVNAQDEDDSN-------------EVELKASIDKILEGLYKYGE--NN----------KK-YLINLDEVDVSLKS

vi7 :--------------------KFNEIKVEGQDDEESS-------------EYKLAQAIDAVLSGIYKKDQ--NG----------KK-ELLTWGEVDPNVKE

vi8 :--------------------QFQDIKVKGQDDEESS-------------EYKLAQAIDAVLSGIYKKDQ--NG----------KK-ELLTWGEVDPNVKE

cy7 :--------------------EFLDIKVKGQDDEESS-------------EYKLAQVINEVLNGIYKTDP--EG----------NK-ELITSEELDENVKK

fi5 :--------------------EFQEIKVKGQDDEESN-------------EYKLEQAIDAVLNGIYKIDS--NG----------NK-ELITWEELDENVKE

so5 :--------------------EFQDIKVKGQDDEESS-------------EYKIAQAIHEVLNGIYKTDS--NG----------NK-ELITWEELNANVKE

in4 :--------------------EFLDIKVKGQDNEQSS-------------EYKVAQAIDAVLNGVYKTDP--NG----------KK-ELITSEELDANVKE

hy4 :--------------------EFLDIKVKGQDDEESS-------------EYKVAQAIDAVLNGLYKTHP--NG----------KK-ELITSEELDENVKK

co4 :--------------------EFQEVKAKGQDDEESN-------------EYKLAQAIDAVLSGIYKTHI--NG----------NK-ELITSEELHANVME

kn3 :--------------------EFEDIKLKGQDDEDSG-------------EYKLAQAIDAILSGVYKTGA--NG----------KK-ELITSEELDANFME

fr2 :--------------------QFKEVKVQGQDDEDSN-------------EYKLAQAIDGVLNSVYKIDT--NG----------NQ-QLITAQEVDANVKE

go5 :--------------------DFLTIKTKGEDDEDSS-------------EHKLVQSIDSILDVVYKTNE--KG----------DK-ELITLEELDLSLKD

vi9 :--------------------AASQEAIPAKASDEESS------------QEELTASIGKILQGVYKKGE--NG----------LN-EVLTFNEADAALKA

cy8 :--------------------APSQESISAQASDEESM------------QEKLAASIGMILQGMYKKGE--TG----------LN-ELLIFDEADAALKE

fi6 :--------------------TSSQESIPAVASDEDSK------------QEELAVSIGKILQGVYKKGE--TG----------LN-ELISFDQADTALKE

so6 :--------------------TPSQESIPAVASDEESK------------QDELAASIGNILQGVYKKGE--TN----------HN-ELLSFEEADTALKA

go6 :--------------------AENQETIPAKGSNEDSS------------EAELDASIDKILEGVYKYDK--TD----------KK-ILVNFFEAEASLKA

ma1 :----------------------NIDNIKARAQDDDENPT----------QMELTQKIDNILKKMYKMDN--NN----------NM-QLITLEGLDDTLKG

ma2 :---------------------QNEQNVLTTGNDEESV------------QYKLMESINNILDLIYKIDV--NN----------NK-ELVKMEEMDNTLKG

ma3 :----------------------RFDEIKTEGQDDENYN-----------EIQLTEAIDSILMGIKDNNK--NN----------KK-DLAIPQDLDSTLKR

ma4 :----------------------KFNEIKAKGQDDENPT-----------HIELTQVIDNILKKMYKMDE--NN----------NM-QLIIFEGLDDKLKS

ma5 :---------------------QKDADILTAGSSEDSD------------EYKLKESIDNILNLIYKTDV--NN----------NK-ELIKLEELDNTLKM

ma6 :--------------------NLNDIEIIAQDDEGSN-------------EYKLTESISNIMKSIYKTDK--GN----------KK-ELKKFEDLDDISKA

ma7 :--------------------STEGNAVTQAADDNTN-------------EVELTESINNILDLIYKVDV--NN----------NK-ELVKMEEMDNTLIG

ov1 :--------------------KFKEIKINAQEEEDPL-------------EVELKQSIETILEEISKQKI--AN----------NS-TENLHYHLSDLLKR

ov2 :-------------------------LLEGGIVTVSNDDNSD--------EYEFKESVNNILGKMYKTDE--NN----------GK-VFISVEEMDNNLKE

ov3 :--------------------RLEEIKIKGQDDDQSD-------------EYELTETIDNILKGIYKTDS--KG----------NK-ELIHWEDVDAALKE

ov4 :TGDVSTTHTGSTTQSHTTEAEASTEEIVSLQRNIFTAASDENSK-----EDQLMQFIDSLLKGVYKTGE--GN----------NK-ELINLEELSSELKE

yo1 :-------------------------SLKTDRLVTAQSEDEI--------NYELMGHIDNVLEGIYNIDEDNNNN---------NK-ELKKFEELDDEIKK

be1 :-------------------------LLNTEVV-TAQSEDEI--------NYELMGHIDNILEGIYNIDENNN-----------NK-ELKKYEELNDEIKK

ch1 :-------------------------SLKADLARAQSDDEI---------NYEVMEHIENILDGIYNVNDN-------------NK-ELKTFEELSDETKK

yo2 :-----------------------YNQVLAVGQSDEDNVDDK--------NLSLVDSINNILNDIYKTDEQN------------NK-VLIDVEDLSTNLKK

be2 :-----------------------YNQILTIGQSDEDNIENQ--------NLSLVDSINNILNDIYIIDEQN------------NK-ILIDVEDLNTNLKK

ch2 :-----------------------YTQVLTTAQSDEDNVEKN--------NTALVESINNLLNGVYKVDEHN------------NK-VLIDEEELSNDLKK

fa1 :----------------------NFNQIKAEAEDDENFR-----------NYHLTDTINNILKRIYKINKN-EG----------KK-ELITLEELDNFLKE

re1 :----------------------NFNQIKAEAEDDENFR-----------NYHLTDTINNILKRIYKININ-EG----------KK-ELITLEELDNFLKE

fa2 :----------------------RFEEILTKGEDDADSD-----------EYDFIAPANYILKNIYKKNDT-NG----------KK-ELLHFKDINNNLKL

re2 :----------------------RFEEILTKGEDHEDSD-----------EYDLIASANYILKNIYKKNDT-NG----------KK-ELLHFKDINNNLKL

fa3 :---------------------SEVKEVKAASVDHSNDK-----------EYELSQSINNILNKMYKKESNDEKNN--------KK-ELIKLEDADDSLQK

fa4 :-------------------LIKKQLKITAQDDEESS-------------EYHLSESIKNLLKNIYKKNNDDNK----------KK-ELLHFENVNSALKS

re4 :-------------------LIKKQLKITAQDDEESS-------------EYHLSESIKTLLKNIYKHNNDDNK----------KK-ELLHFENVNSDLKS

fa5 :----------------------KFKEIKAETEDDDEDDYT---------EYKLTESIDNILVKMFKTNENND-----------KS-ELIKLEEVDDSLKL

re5 :----------------------KFTEIKAETEEDDEDDYT---------EYKLTESIDNILIKMFKTNENND-----------KS-ELIKLEEVDDSLKL

fa9 :------------------KLNEERFKVKGSTEYEESV------------ESELKGAIDLLISSFYKSDGENG-----------EN-VLISFEDLNSTLED

re9 :------------------KLNEELFKVKGSTEYEESV------------ESELKEAIDLLISSFYKSHGENG-----------EN-VLISFEDLNSTLED

ga1 :---------------------KFEQSIITESSDEDSN------------EFKLEETIDNIMKKIYKTDPISK-----------KK-ELITLEDFNDELRE

vi10 :--------------------KHSNAKVKGQDELSPK-------------EYELTESIDIILKNIYKVDTINK-----------KK-VLISMDDFDDVLKA

cy9 :--------------------KHNNAKVKGQDELSPK-------------EYELTEAIDIILKNIYKVDAQNK-----------KK-VLISMEDFDDVLKA

fi7 :--------------------KHNNAKVKGEDELSPK-------------EYELTESIDMILKNIYKVDTINR-----------KK-VLISMDDFDDVLKA

so7 :--------------------KHNNAKVKGEDELSPK-------------EYELTESIDMILKNIYKIDKVNK-----------KK-VLISVDDFDDVLKA

in5 :--------------------KHDNAKVKGQEDMNPK-------------EYEITESIDIILKNIYKVDTVNK-----------KK-VLITMDDFDDVLKA

hy5 :--------------------KHDNAKVKGQEDMNPK-------------EYELTESIDIILKNIYKVDRVNK-----------KK-VLITMDDFDDVLKA

co5 :--------------------QHNNAKVKGQDDVNPK-------------EYELTESIDIILKNIYKVDKIKK-----------KK-VLIGMDDLDDVLKV

kn4 :--------------------QHNNAKVKGQDDLNPK-------------EYELTESIDIILKNIYKVDKVKK-----------KK-VLIGMDDLDDVLKV

fr3 :--------------------QHNNAKVQAQDAVNPK-------------EYELTEAIDIILKNMYKVDRIKK-----------KK-VLISMDDLDDVLKA

go7 :--------------------KCNNIKMIGQDDLNPK-------------EYELTESIDVILKNIYKVDNINK-----------KK-VLITLDDFDDVLKA

ma8 :--------------------KYNEIKVMGQDDQDSK-------------EYQFNESIDIILKNIYKVDISKN-----------SK-VLITLDDFDDVLKI

ov5 :--------------------KFNDIKTKAQDEENSK-------------ENELTESIDIILKNIYKMDEKKK-----------KK-VLITIDDFDDVLKA

yo3 :--------------------NLDQIKISGQSDEDTK-------------ENDLIESIEILLNSFYKVDKKTK-----------KM-SLITMDDFDDVLRT

be3 :--------------------NLNEIKVTAQSDEDSK-------------ENDLIESIEILLNSFYKADKKAK-----------KL-SLITMDDFDDVLRA

ch3 :--------------------NLNDIKITAQSDEDNK-------------EIDLIESIDILLSKFYKVDLKTK-----------KL-GLITIDDFDDVIKV

fa6 :--------------------KMNEIKVKAQDDFNPN-------------EYKLIESIDNILSKIYKKANKPFEI---------SK-DLINLEDLDYQFKN

re6 :--------------------KMNEIKIKAQDDFNPN-------------EYKLIESIDNILSKIYKKANKPFEI---------SK-DLINLEDLDYQFKN

vi11 :-------------------LIEKDTPIKAQEEDANSA------------DHKLIESIDVILKAVYKSDKDEE-----------KK-ELITPEEVDENLKK

cy10 :-------------------LIEKDTPIKAQEEQAHSA------------DHKLIESIDVILKAVYKSDKDDE-----------KK-ELITLEEVDENLKK

fi8 :-------------------LTEKDMPIKAQEEDGHSA------------DHKLIESIDVILKAMYKSDKDDE-----------KK-ELITLEEIDENLKK

so8 :-------------------MIEKDMPIKAQEEDGHSA------------DHKLIESIDVILKAMYKSDKDDE-----------KK-ELITLEEVDSNLKK

in6 :-------------------FIEKDTPIKAQEEEAQSA------------DHKLIESIDVILKAVYKSDSDDE-----------RK-ELITLEEVDANLKK

hy6 :-------------------LIEKDTPIRAQQEEAQSA------------DHKLIESIDVILKAVYKSDLDDE-----------RR-ELITLEEVDANLKK

co6 :-------------------MIKKDTPIKAQEEDANSA------------EHKLIESIDVILKAMYKSDKDYE-----------KK-ELITLEEVDAKLKK

kn5 :-------------------FIKKDTPIKAQEEDANSA------------EHKLIESIEVILKAMYKSDKDYE-----------KK-ELISLDQVDTNLKK

fr4 :-------------------LIKKDTTIKAQEEDVNST------------EHKLIESIDVILKAMYKSDKDYK-----------KK-EIITLEQVDENLKK

go8 :-------------------LILKDTTVKGE-DDEQSA------------DYKLIESIDAILKMVYKSDKDDE-----------KK-ELITVEEISEDLKN

ma9 :-------------------RIAMDIPTIAQDEEYSVE-------------YRLIESIDIILKSIYKTDENGG-----------KKEELINFEDLENDLKS

ov6 :-------------------NISKDIQTRGDDDAEETEDVEKQQVKEETADHKLTESIETILKKIFKNDKYG------------KK-ELILFEELDESLKG

yo4 :-------------------KINNDTIIKGEDDLDENSD-----------EKILREHIYKILKKMYNNDSDYCGNNRCN-----KK-MIKNIKELDTDLQI

be4 :-------------------KINNDTIIKGEDDLDEN---------------ILRENIYKILKKMYNKDSDYCDNNRCN-----KK-MITNIKELDTDLQI

ch4 :-------------------KINKDTIIKGEDESDENSD-----------EYMLKESIYKILKKMFTNDSDYCDGSRCN-----KE-MITDIKELDADLRI

fa7 :--------------LINKNLEEKNKTFKGEDEDLDSN------------EQKLEESIDNILSNIYKIYESKQDKERKKSHYNNKK-ELVTIEELNSVLKI

re7 :--------------LINKNWEEKNKTFKGEDEDLDSN------------EQKLEESIDNILSNMYKIYESKQDKERKKSHYNNKK-ELVTIEELNSVLKI

vi12 :----------------------KTSRDKGAFVEMKDH------------DYLTDGKVDDLISEVVKISLERQ-----------KN-AVPAAEDRNEDFHK

cy11 :----------------------QTSRDKGAFVEMKDH------------DYLTEDRVDELISEVIKLSLEKQ-----------KN-AVPATEHTNEEFHK

fi9 :----------------------QTSRDKGAFVEMKDH------------DYLTEDKVDDLISEVVKLSLERQ-----------KN-AIPATEHMNEEFHK

so9 :----------------------QTSMDKGAFVEMKDH------------DYLTEDKVDDLISEIVKLSLERQ-----------KN-AVPSTEHMNEEFHK

in7 :----------------------QTSREKGAFVEMKDH------------DYLTEDKVDDLISEVVKLSLERQ-----------RN-AVPGTEDTNEDFHK

hy7 :----------------------QTSRDKGAFVELKDH------------DYLTEEKVDDLISEVVKLSLERQ-----------RN-AVPGTEDANEDFHK

co8 :----------------------QTSRDKGAFVEMKDH------------DYLTEEKVDDLISEIVKLSLERQ-----------KK-AVPATEQANEDFRK

kn6 :----------------------QISRDKGAFVEMKNH------------DYLTEAKVDDLISEIVKLSLERQ-----------KN-AVPETEQTKQDFQK

fr5 :----------------------QASRDKGAFVEMKDH------------DYLTEDKVDDLISEIVKLTLERQ-----------KS-AQAETEYTNEEFQK

go9 :----------------------QTSTDKENFVELKDH------------DYLTENKVDALISEVVKMSSEMH-----------KN-EASARENLSEDLKK

ma10 :----------------------MDQKSKGAFVELQDH------------EYLSDEKMAILLSEIIKISIDRH-----------KK-GVAGGSTLDNDLKK

ov7 :----------------------NGKREKGAFVELNDH------------DYLTEGKITHLISEVIEASVTRY-----------KD-GLTDEASMSENYKK

yo5 :-----------------------SYQGNNALLELRDQSLQ---------DSVSEESMNELISNILDVAISRY-----------EN-QKTDVNSMDDDYKS

be5 :-----------------------NYKGTNALLELRDQSLQ---------DNVSEESLSELISKILDVAIFRY-----------EN-KKTDANSMDDDYKS

ch5 :-----------------------NNKENNALLELKDESLQ---------DSISEDSLDGLVSSILDIAMLRY-----------KN-KTTDAGSMDDDYKT

fa8 :---------------------MSKIDMNNTFVELKDH------------EEVTDEKMKNLITKIIEIAIDRH-----------TL-GLHDFSSVSDEYKE

re8 :---------------------MSKKDMNNTFVELKDH------------EEVTDEKMKNLITEIIEIAIDRH-----------TL-GLHDFSSVNDEYKE

ga2 :---------------------LSK-GEIKAFIELQEE------------DYLTREQMRALIEEIIKISLYRI-----------KK-RLNDEHSMSDDLKK

ga3 :---------------------MSQ-SEIEAFIELHEE------------DYLKTENFDSLIEEIVDFSKEVL-----------GK-TTEDEKSDDELKKK

392 :-------810-------820-------830-------840-------850-------860-------870-------880-------890-------900

392 :#####################################----------------####################---------------------------

570 :###################################################--########################-----------------------

vi1 :LLLRYCQVMKKVDRSGTLEEHEVGNEMDVLANLEGLLRKHPKEEIPALREK--LKNPAICMKDAAKWVEQKRGLALPLFEYKHLQRR-VTTAVDSQESN-

cy1 :LLLSYCQMMKKVDTSGTLEEHELGNEVDVLTNLEGLLRKHSNEEIAVLREK--LKNPAICMKDADMWIVQKRGLALPTFEYKHLQRRRVTTPVDSKEDNK

fi1 :LLLSYCQMMKKIDTSGTLEENELGNEMDVLTNLKGLLRNHSNEEVPILREK--LKNPAICMKDAGKWVLQKRGLALPLFEYKHLQKRRVTTPVDSQEENK

so1 :LLLSYCQMMKKMDSSGTLEEHELGNEMDVLTNLKGLLRNHSNEEIPVLREK--LKNPAICMKDASKWVLQKRGLALPLFEYKHLQRRRVTTPVDSQEDNK

in1 :LLLSYCQVMKEVDTSGTLQQHELGDEMDVLSNLRDLLRKHSNEEIPALREK--LKNPAICLKDAGKWVLQKRGLELPPFQYKHLQRR-VTTPVDSQEQNK

hy1 :LLLSYCQLMKMVDTSGTLEEHEIGNEMDVLSNLRNLLRKHSNEEIATLREK--LKNPAICMKYPDKWILQKRGLALPPFHYKHLQRS-VTIPVDSQEQNK

co1 :LLLSYCQVIKKVDKSGTLEVHQMGNQMDVLTNLKGLLKNHSKEEIPALREK--LKNPAICMKDASKWVVQKRGLALPPFEYKHLQRR-VTTPVDSQEEHK

kn1 :DLTNYCQIMKKVDKSGTLEEHQIGGEMDVLTNLKGVLKNHSHEELPTLEGK--LKIPAICMKDASKWVVNKRGLVLPPFEYKHLQRR-VITSVDSQEENK

vi2 :ELLNYCALMKEVDASGVLDQYQLGSEEDIFANLTSILKNHAGETKSTLQNK--LKNPAICLKNANEWMESKKGLLLPSLSYTNVEATLPENAPEKE----

cy2 :ELLNYCASMKEVDSSGVLENYELGSEEEVFANLTNILKNHAGETKSTLQTK--LKNPAICLKNADEWVKSKKGLLLPSLSYTHVEATLPATAPENEEKKE

fi2 :ELLNYCALMKEVDTSGVMDNYELGTEEDVFFNLTSILMNHAGETKSTLQNK--LKNPAICLKNAHKWVESKKGLLLPSLSHTPVEATRSAIAPEKEEEKE

so2 :ELINYCALMKEVDTSGVMDKYELGTEEEVFSNLTSILMNHAGETKSTLQNK--MKNPAICLKNADKWVESKKGLLLPRLSHTHVEATLPETAPEKE----

in2 :ELLNYCASMNEVDASGVLDSFELGTEEDVFANLTRILRNHAGETKSMLQNK--LKNPAICLKNADEWVERKKGLLLPSLSHTHVEATPPANAQEEEAKKE

hy2 :ELLNYCASMKEVDASGVLDNYELGTEQDVFANLTRILRNHEKETKSILQNK--LRNPAICLKNADEWVESKKGLLLPSLSPTHVGATSPATTPEEEVKEE

co2 :ELLNYCASMKKVDTSGVLENYELGSEEDVFSNLTNILKNHAGETKSTLQDK--LKNPAICLKNANQWVGRKKGLVLPSLSHTHGEGTLSAIATEKEGKKE

go1 :ELLKYCSLMKELDTSGTLDNYDLGTVEEIYINLKKLLEENSDHDKVALKNK--LKNAAICMKNTEEWVGNRKGLLLPILSNTKVDSPHYEAAKEEEK---

go2 :ELTNYCNLLKEIDSSGVLDNYEIGNETDVFNNLSKLLKKHTNDNFSTLKDK--MKNVAICMKNVKEWVSNKKGLKLPQLKYTKLEDTASHTYDNPKQSTC

vi3 :ELLKYCSLMKEVDTSGALDNHQLGNAEDVFAHITTMLHSNSDLDVFSLKSK--LKNAALCLKNGDEWVGSKTGLVLPNLSPQNFEDSPTQTFDGGDEQAA

vi4 :EIQNYCAMMKEMDTTGVLENYEMGNADDVFGNIAKMMGKNGNYSVSSLPNK--MKNAAICLKSPDDWVEGKTGLMLPNLVPPYLVHNNVEDVTTSLETSS

vi5 :DLLKYCSLMKEVDTSGSLDNHQLGDAEDVFANLTHLLQSNSDHDVPSLKNK--LKSPAICLKNVGHWVGSKTGLVLPTLEYSTSQDNAES----------

cy3 :ELLKYCSLMKETDSSGALDNHELGNAEEVFAHITTMLQSNSDLNVYSLKNK--LKNPAICLKKVGHWIGSKTGLVLPILEHSSVHDSANT----------

cy4 :ELLKYCDLMKEMDISGVLENYEMGNTDDVFAHIANMLEKNGNYAVTLLPNK--MKNVAICLKNPDEWVDGKTGLLLPKLAFPHLVHKNVQDGSNSVETTV

cy5 :ELLKYCSLMKETDSSGALDNHELGNAEEVFAHITTMLQSNSDLNVYSLKNK--LKNPAICLKKVGHWIGSKTGLVLPILEHSSVHDSANT----------

fi3 :ELLKYCSLMKEVDVSGALDNHELGNAEEVFAHITTMLQSNSELNVFSLKKK--LKNPAICLKNVGHWIGSKTGLLLPTLEYNNVQNNVDL----------

so3 :ELTKYCSLMKEVDASGALDNHELGNAEEVFAHITTMLQSNSELDVFSLKKK--LKNPAICLQNVGHWIGSKTGLLLPTLEYNSVQNNADL----------

in3 :ELLKYCALMKELDASGALDNHELGNAEEVFSHITTMLQSNNDLDAFSLKNK--LRNPSLCLKKVDHWIGGKTGLVLPSLKYNSGQNNEDSSDK-------

hy3 :ELLKYCSLMKEIDASGALENHQLGNADEVFSHITTMLQNNSDHDAFSLKSK--FKNPALCLKKVDHWIGGKTGLVLPSLEYSNEQN--------------

co3 :ELLKYCSLMKEVDASGTLDNHELGNPEEVFAHITTILHNNSELDVFSLKNK--LKNPALCLKKVGHWIGSKTGLTLPSLENSSVQNNADS----------

kn2 :ELLKYCLLMKEMDASGTLDNQELPNPEDVFAHITTLLQNNNELNVFSLKNK--MKNPALCLKEVDQWIGSKTGLTLPALEQSTVQNNEET----------

go3 :ELIKYCQLLKQTDTSGVLDNHEMANEEEAFTNLTNMIEMNSNYDINDLKGK--LKNVAICMHNPNEWVSIKTGILLPVLSHNLGDGKDITLNTNKEGNDT

vi6 :ELLNYCSSMKEVDASGVLDQYQLGSEEDIFANLTSILKNHAGETKSTLQNK--LKNPAICLKNADEWVESKKGLLLPSLSHTNGEVTPPET-----PKED

cy6 :ELLNYCASMKEVDSSGVLENYELGSEEEVFANLTNILKNHAGETKSTLQTK--LKNPAICLKNADEWVESKKGLLLPSLSHTHVEATFPAT-----TKAE

fi4 :ELLNYCALMKEVDTSGVMDNYELGTEEEIFSNLTSILMNHAGETKSTLQNK--LKNPAICLKNAHKWVESKKGLLLPSLSHTHVEATLPAT-----FEVA

so4 :ELLNYCALMKEVDTSGVMDNYELGTEEEVFSNLTSILMNHAGETKSTLQNK--LKNPAICLKNANEWVESKQGLLLPSMSHTHVEDTLPAT-----SEVV

fr1 :ELLNYCASMKEVDSSGVLDNYELGSAQDVFVNITNMLHMNSDCSVSSLQNK--LKNPALCLKNTDQWVDSKKGLLLPSLAQAHVEATPSAT-----PKVE

go4 :ELLKFCTLLKNTDSSGALENYTLGNAEDIFTNLTNILKKNTDETKSAIQTK--LKNVSICMKNVDEWIQNKKGVQIPTLSHTSEEFTNSTITKEEEGKTV

vi7 :QISSYCHLLKDVDTSGTLDVHQMGSQEHIFNNLITLLQKHAEEKQESLQNK--LKNAAICLKQAHNWVANKVGLALPQLAYNPGKGVEQVEENPPNGESA

vi8 :QISSYCHLLKDVDASGSLEVHQMGSEMDIFNNLITLLQKHSEEQKATLEWK--LQNPALCLKDANNWVVNKKGLLLPLLQNGGSAINFGESNHVEDVKKD

cy7 :HIGNYCQVLKEMDISGTMEVHQMGNEMDVFKNLVQLLQKHGEEKNSTLEWK--LQNPALCLKNVNDWVVNKKGLVLPLLQNGGSDIYFGESNLVEGGQKS

fi5 :QIGNYCHVLKEVDISGTLEVHQMGNEMDVFNNLVKLLQKHGEEKKLTLEWK--LQNPAICLKNVNDWLVNKKGLVLPPLQNGGSDIYFGESNHVEDEKKC

so5 :QIGNYCHVLKEVDTSGTLEVHQMGNEMDVFNNLVNLLQKHGEEKKLTLEWK--LQNPAICLKNVNDWVVNKKGLVLPLLQNGSSDIYFGESNHVEDEKKC

in4 :QIGKYCHVLKEVDTTGTLEVDQMGNEIDVFNNLVKLLQKHGEEKKSTLEWK--LQNPALCLKNVNDWVVNKKGLVLPPLQNGDNDIYFGDSNDVEDEQKY

hy4 :QIGNYCHVLKELDTTGTLKVHEMGNEIDVFNNLVKLLQKHGEEKKSTLEWK--LQNPALCLKNVNDWVVNKKGLVLPTLQNGGSDIHFGDSNDMEDEKKS

co4 :QISNYCHVLKEVDTSGTLEVHQMGNEIEVFNNLVNFLQKHGEEKYSTLEWK--LQNPALCLKNVNDWLVNKKGLVLPLLPNGGSHIYLGDNNHVQDEQKC

kn3 :QIGNYCQIMKMVDKSGTLKEYQMGTEVDVFNNLVKFLQTHEEEEYSTLEWK--LQNPVLCLKNVNDWVVNKKGLLFPLLQNDDSHIHLGEYDKEGEEQKC

fr2 :QLSYYCHVLKEVDTTGTLDVHTIGCVMEVFNNLVKLLQKHGEENYSTLELK--LQNPAICLKNVNDWVLNKKGLVLPQLQNAGGHVYFGENIQAQNEQNC

go5 :ELKNYCQLLKEVDTSGTLEVHQMGSETDVLKNITKILQKHKDEKKISLHLK--LQNPAICIKNVDEWMVHKKGLTLPILQNGHGDDYSGEQSNVGDKQND

vi9 :ELLNYCSLMKKVDASGVLGHYQLGSEEDVHANLTNMLQTNSDHVLSSLQNK--LKNPAICLKNADEWVGSKTGLLLPNLFYNHLEGST-----PSTSNVT

cy8 :ELLNYCALMKEVDTSGVLENYELGSEENVFANITNMLQKNSDYSVSSLQNK--LKNPAMCLKNADEWVGSKMGLVLPNLPYNHLEVPH-----PSTPEVA

fi6 :ELLNYCALMKEVDTSGVMDNYELGTEEDVFSNITNMLQTNSDYSLSSLQNK--LKNPAICLKNADEWVESKKGLLLPSLSHTYVEATL-----PATSEVA

so6 :ELINYCALMKEVDTSGVMENYELGTEEEVFYNLTNMLQTNSDYSLSSLQNK--LKNPAICLKNADKWVESKKGLLLPILSHTHVDATL-----PATSEVV

go6 :ELLNYCSLLKTVDNSGILENYELGNEEQIFSNLTKILKKHPEETKSTLYNK--LKNVAICMKNAEEWMENKKGLLLPTLSFEDMKLNHNYTAKEGEEKTV

ma1 :ELVQYCKLLKEVDTSGTLESYEMGNETEIFNNLTRLLAKHADETYASLQHK--LRNAAICMKNVNDWIKNKRGLSLPQFEYHNLENNSNNLQNYENSTQN

ma2 :ELMKYCKLLKQEDVSGTLENHELANHVETYSNLTKLLEKHNEEKKSSLLYK--LKNPAICMKYAENWITNKTGLVLPNLSYKNNENKIQLQTTKVSETSS

ma3 :QITNYCNLLKGLDMTRMLENYELGNEMEVLNNLNRMLKMHKGEKYISLKNK--LKNAAICMKNVSNWGENRTGLVLPDLPYNKLEGKNVMHFNYDVELKD

ma4 :ELINYCKLIKEIDTSGALENYEMGNETDIFNNLTTLLQTHAEETYSSLQHK--LTNAAICMKNVNDWIKNKTGLILPVLSYNTIENQGNPLSSEQSEVED

ma5 :ELINYCNLLKQVDMSGTLENHELANDIETYNNLTKLLIKHDNEKKTSLHDK--LKNPAICMKNVHEWIKNRRGLVLPQMTYKNVADINNVHLNQNEELQD

ma6 :ELMNYCMLLKEVDTNGTLEMNELGKETDVFNNISRLLKNHIQENNTALVKK--LKNAAMCMKYIDNWVVNKTGLILPELSYKKLENTNDVYLNENGELQI

ma7 :ELMKYCKLLKQEDVSGTLENHELANHVETCSNLTKLLEKHNEEKKSSLLYK--LKNPAICMKYAENWITNKTGLVLPNLSYKNNENKIQLQTTKVSETSS

ov1 :EMVKYCQMLKEADTSGVFEYVQMGNEADIFYNLTKLLKKHEDEMDFLLQRK--LRNAAICMKNADEWVTRKMGLILPQLPTNNMEYTNGTYYSEDKEREK

ov2 :QLLNYCTFLQKMDNTGTLGNNTVGNEVDIYHNLMNMLKNHKNEDQFTLQNK--LKNAAVCMKNVDDWVSNRRGLVLPDLSYNMEDNYDTLDVEKNFENNE

ov3 :ELTKYCMLLKGLDTSGMLDYFHLGNEIDVFNNLSKFLKKHKEEKHFLLKKK--LKNAVMCIKSVDEWVKNKTGFVLPQLSYSILDNTNGQQQSDKKE---

ov4 :ELKTYCNLLKEVDTSGILENYQMGDVTDIFYNLTKLLKAHPDEKKYILQNK--MMHPAICMKNVEEWAKNKTGLVLPHLSSNDLEHVKGTYYSEDDMLQN

yo1 :DMLLYCKELKESDVSGTLEEYALGDVEDIFYNLIQLINNNTGVVISKLKNK--LMNPAICLKDVNQWGEKKKGLVLPELSSNNFDNLNDKNDDTTINS--

be1 :DILLYCKELKESDVSGTLEEFVLGDVEDIFYNLTKLITNNTEISISKLKNK--LMNPAICLKDVNQWGEKKKGLVLPELISKDFENLNGKNKNDDTTMN-

ch1 :DILLYCKELKESDVTGTLEDYASGDAEDVYANLTKLITNNMEDSISKLKSK--LMNPAICLKDVNQWGEKKTGLVLPELSSNNVDNSNENDDTKTDLES-

yo2 :ELTYYCQILNEIDTSGTLDIYKIGNSVEIFNNLIRLLKNHQDENKSYMINK--LKNPAICMKHVEQWVVNRKGLKLPIILDDSETENKNSEESKNTNISI

be2 :ELTYYCQILSEVDTSGTLDIYKIGNSVEIFNNLIRLLKNHQNENRLYIINK--LKNPAICMKHVEQWVANRKGLKLPIILDDAETENKNLEELNNTNSTI

ch2 :ELTYYCQVLSEIDTSGTLENYKVGNSVEIFNNLAKLLKNHENENKLYLINK--LKNPAICMKDVEQWVVNRKGLKLPVALDDAE---------KKTDNAI

fa1 :SITDYCKILREIDTNGTLVNHELGNNVDVFNNLIRLLKLHKNESISTLHNK--LRNSAICMKYPDKWIEKKTGLILPNVVNNNIIYNNKYEKLNEEKKRK

re1 :SITDYCKILREIDTNGTLVNHELGNNVDVFNNLIRLLKLHKNESISTLHNK--LRNSAICMKYPDKWIEKKTGLILPNVANNNIIYNNKYEKLNVEKKRK

fa2 :ELINYCNLLKDNDVSGILTYEKLGNVQDIFNNLTKLLEEHKEENNYVLYHK--MKNEVLCLKNANDWMKNKTGLVLPQLKYSLNKFNKNKENYIKENIFE

re2 :ELINYCNLLKDNDVSGILTYEKLGNVQDIFNNLTKLLEEHKEDNNHVLYHK--MKNEVLCLKNVNDWMKNKTGLILPQLKYSLYTFNKNKENYIKENIFE

fa3 :ELNKYCNSLKEVDLNGVLSKNEVGNEKDVFNNLTTLLKEHMLESHHVVFEK--LKNSALCLKNIDDWLKNKNGLIVPPSKYKLKDTNEKKELNNNVEVIE

fa4 :ELLNYCNLLKEVNMNGVLKDHQLGNVQDVFNNLTKLLEEHKEENDNVLYHK--MKNEALCLKNVNDWMKNKTGLLLPQLSYDLTYKNNNFTEFTQNKSY-

re4 :ELLNYCNLLKEVNMNGVLKDHQLGNVQDVFNNLTKLLEEHKEENDNVLYHK--MKNEALCLKNVNDWMKNKTGLVLPQLSYDLTYKNNNFTEITQNKTY-

fa5 :ELMNYCSLLKDVDTTGTLDNYGMGNEMDIFNNLKRLLIYHSEENINTLKNK--FRNAAVCLKNVDDWIVNKRGLVLPELNYDLEYFNEHLYNDKNSPEDK

re5 :ELMNYCSLLKDVDTTGTLDNYEMGNEMDIFNNLKRLLIYHSEENINTLKNK--FRNAAVCLKNVDDWIVNKRGLVLPEINYDLEYFNEYLYNDKNSPEDN

fa9 :DIINFCSLLKLVDLSGTFMNHQIGTTADAFNNIVKLLKEHKEEDEFVLRTK--LRNPALCMKNVDDWVKSRCGLLTSDDSYVVIKKNSDTNGKNENNESN

re9 :DIINFCSLLKLVDLSGTFMNHQMGTTVDAFNNIVKLLKEHKEEDELVLRTK--LRNPALCMKNVDDWVKSRCGLFTSDDSYVVIKKNYDTDGENENNESN

ga1 :EIRNYCDLLKEVDVSGTLENHELGNETEVFNNLTRLLRAHDEQRDTTLPNK--LKNAAICIKNVDEWIINKRGLVLPEVTNEYFNNVKDMPEVENEEEYE

vi10 :ELLNYCKLLKEMDVKGTLDNCELGNEVDIFNNMVRLLSMHPKENIITLQDK--LRNTAICLKNVDEWVENKRGLVLPEGGEKAALEGNP-TEVV----DD

cy9 :ELLNYCKLLKEMDDKGTLENCDLGNEVDIFNNMIRLLSMHPNENIITLQDK--LRNTAICLKNVDEWINNKRGLLLPEGEEMSSFEGTPTTQVDK----D

fi7 :ELLNYCKLLKEMDVKGTLENCDLGNEVDIFNNMIRLLSMHPKENIITLQDK--LRNTAICLKNVDEWVKNKRGLLLPEGEEKSSVEANR-TQVD----ED

so7 :ELLNYCKLLKEMDVKGTLENCDLGNEMDIFNNMIRLLSMHPKENIITLQDK--LRNTAICLKNVDEWIKNKRGLLLPEGKEKSSLEGNP-TQV----EEE

in5 :ELLNYCKLLKEMDVKGTLENYDLGNEVDIFNNMIRLLSMHPKENIITLQDK--LRNTAICLKNVDEWVKNKRGLLLPEGKEMSSLEGSP-TQVD--EDED

hy5 :ELLNYCKILKEMDVQGTLENYELGNEVDIFNNMIRLLSMHPTENIITLQDK--LRNTAVCLKNVDEWVKNKRGLSLPEGEEMSTLQGSS-TQED----EE

co5 :ELLNYCKLLKEMDVKGTLENCELGDEIDIFNNVIRLLSMHPKENIITLQDK--LRSTAICLKNVDEWVEKKRGLVLPEGEESSSHEGNP-TKVDKEDDDN

kn4 :ELLNYCKLLKEMDVKGTLDNLDLGDEIDIFNNMIRLLSMHPKENIITLQDK--LRNTAICLKNVDDWIENKRGLLLPDEDESSSLAKNP-TQVDKEDESD

fr3 :ELLNYCKLLKEMDVKGTLDNFELGNEVDIFNNMIRLLSMHPKENIITLQDK--LRNTAICLKNVDEWVENKRGLVLPEGGEEKSSLEEKSTQVD----ED

go7 :ELLNYCKLLKEMDEKGTLENYELGNELEIFNNIIRLLKMHPKENIITLQDK--LRNTAICIKNVDEWVENKRGLVLPKEGEISSLESSS-TDSKT--NET

ma8 :ELMNYCKLLKEMDVKGTLENHQLGNEVDIFNNLIRLLSIHPNENIITLQDK--LRNTAICLKDVDKWIENKRGLILPVLEDNTISGNSNSSSNIEEEEDD

ov5 :ELTNYCKLLKEMDSKGTLESYELGNEIDIFNNVIRMLSMHPKESIITLQDK--LRNTAICIKNVDEWVQNKRGLVLPESTHASKLENPSNISIMDENEDE

yo3 :ELFNYCKLLKELDTKKTLENAELGNEIDIFNNMLRLLKTNEGETKLNLYKK--LRNTAICLKDVNTWAEKKRGLILPEELTQDQMTEGQNEEPNDEDPDD

be3 :ELFNYCKLLKELDTKKTLENAELGNEIDIFNNLLRLLKTNEEESKHNLYKK--LRNTAICLKDVNKWAEKKRGLILPEEVTQDQMAIGQNEEPYDEDPDD

ch3 :ELYNYCKLLKGLDTQKTLENVEMGDEMDVFNNLLRFLKTNEGETKLNLYKK--LRNTAMCLKDVNTWAEKKRGLILPEETTEQ-FASAQSEGFYEEDPDD

fa6 :ELLEYCKLLKKVDTSGTLEEYELGNAEDIYNNLTRLLKSHSDENIVTLQGK--LRNTAICIKNVDEWILNKRGLTLPSESPSESSSKSDSYLNTFNDKDK

re6 :ELLEYCKLLKKVDTSGTLEEYELGNAEDIYNNLTRLLKSHSDENIITLQGK--LRNTAICIKNVDEWILNKRGLTLPSESSSE----SDSYLNIFNDKDK

vi11 :ELANYCTLLKEVDTSGTLNNHQMANEEETFRNLTRLLRMHSEENVVTLQDK--LRNAAICIKHIDKWILNKRGLTLP---EEGYPS-EGYPPEEYPPEEL

cy10 :ELANYCTLLKEVDTSGTLNNHKLANEIETYRNLTRLLRMHSEENVVTLQDK--LRNAAICIKDIDKWIINKRGLSLP----DGTPYYSEENTKEYLAEEF

fi8 :ELANYCTLLKEVDTSGTLNNHKLADEVETYKNLTRLLRMHREENVVTLQDK--LRNAAICMKHIDKWIVNKRGLTLP----DGTPY-SEENTEEYLPEEL

so8 :ELANYCTLLKEVDTSGTLNNHKLANEVETYRNLTRLLQMHREENLVTLQDK--LRNAAICMKHIDEWIVNKRGLTLP----DGVPY-NEDNTEEYLPEEL

in6 :ELANYCTLMKEVDTSGTLNNHKLANEVETYKNLTRLLRMHKEENVVTLQDK--LRNAAICMKHIDKWILNKRGLTLP----DSIPY-SEDNTEEYLPEEL

hy6 :ELANYCTLMKEVDTSGTLDNHKMANEIETYNNLTRLLRTHKEENIVRLQEK--LRNAAICMKHIDKWILNKRGLTLP----D-SPY-REDNTEGYLPEEL

co6 :ELANYCSLLKQVDTSGTLKNHQLANEEETYRNLTRLLQMHKGENVITLQDK--LRNAAICMKDIDKWIVYKRGLTLP----EGVPY-SEDNTKEYLPEEL

kn5 :ELSNYCQLMKQVDTSGTLKNYKLANEVETYRNLKKMLQMHKEENIITLQDK--LRNAAICMKDIDKWIINKRGLTLP----DGVTY-SEDNTKEYLSEEL

fr4 :ELASYCQLLKEVDTSGTLKNHKLANEVETYKNLTRLLQMHKGENIVTLQDK--LRNPAICMKDIDKWIINKRGLTLP----DGIPH-SEENTKEYLHEEL

go8 :ELLNYCVLMKEIDTSGTLDNHELANEIETFNNLTRLLQMHSEENIVTLHDK--LRNTAICIKNVNEWIVNKRGLNIP----DGSTT-HNEQIEPNQIEEL

ma9 :ELKNYCALLKEMDESGTLKNFELANEEETFNNLSRLLKVHPNENMATLRLK--FRNPAICVKNVDEWIVNKRGLTLHNETYSAEKVSHDQANADSGAADK

ov6 :ELMKYCNLLKDVDVSGTLQKHELANNIETFNNLTRMLKMHAEENVVTLQDK--LRNAAICVKNINEWVINKRGLLLPDEMHNLENTHYDVDEVDDTHYDA

yo4 :YIKNYCDILKKVDKSGTLDSHEIANEVEAFNNLVRLLNSHTSENIYILFEK--LKSPAICVKNINEWIVRKRGLVIS-NEYDINSYDTTEEPNIKNILSD

be4 :YLKNYCDILKKVDKSGTLDAHEIANEVEAFNNLIRLLNSHTNEKIYILYEK--LRNPAICVKNINEWIIKKRGLVIS-NEYDINLHNVTQESNITNILNE

ch4 :DLKNYCDILKKVDKSGTLEVHEIASEVEAFNNLVRLLNTHTNENIYILYEK--LKNPAICIKNVNEWIIKKRGLVIS-KEHDVNPYNNPDNPKIKNILNE

fa7 :ELLNYCKLLKEVDRSGMLDHHEIGNEIDIFNNLIRLLKAHPGESTYVLNEK--LRNPALCFKNIEEWLVNKKGLLLS-NEKIQNL-----STTNYNVTDL

re7 :ELLNYCKLLKEVDTSGMLDHHEIGNEIDIFNNLIRLLKAHPGESTYVLNEK--LRNPALCFKNIEEWLVNKKGLLLSSNEKMKNL-----STPNYNMTEL

vi12 :KIMQLCLYSNFNDNYENAKKHTQASVEEVERHIHKIINMYMRESNNMEHIINSLKNPALCLKNPAEWTKDRAGYKDND-VPSVGII--------------

cy11 :KIMQLCLYSNFNDNYENAKKHTQANAEEVEKHIHKVVSMYMKESNNMEHIINSLKNPALCLKSPEEWTKDRAGYKDNDDVPSV-II--------------

fi9 :KIMQLCLYSNFNDSYENAKMHTQASAEEVEKHINKIVTMYMKESNNMEHIINSLKNPALCLKDPAEWTKDRAGYKDND-VPSVGII--------------

so9 :KIMQLCLYSNFNDSYENAKKHTQANAEEVEKHINKIVIMYMKESNNMVHIINSLKNPALCLKDPAEWTKDRAGYKDND-VPSVGII--------------

in7 :KIMQLCLYSNFNDNYENAKKHTQASAEEVEKSIHRIVSMYIHESNNMEHITNSLKNPALCLKDPAEWTKDRAGYKDND-VPSVGII--------------

hy7 :KIMQLCLYSNFNDNYENAKKHTQASAEEVEKSIHRIVSMYIHESNNMEHIANSLKNPALCLKDPSEWTKDRAGYKDND-VPSVGII--------------

co8 :KIMQLCLYSNFSDHYENAKKHTQADAEEVEKHIIRIVNMYIKENNNMEHITNSLKNPALCLKDPAEWTKDRAGYKDNN-VPSVGII--------------

kn6 :KIMQLCLYSNFSDHYENAKKHTQANAEEVEKHINKIVIMYMNESNNMEHIINSLKNPALCLKDPAEWTKDRAGYKDND-VPSVGII--------------

fr5 :KIMQLCLYSNFSDNYENAKKHTQANAEQVQKHINRIVLMYMKESNNMEHIINSLKNPALCLKDPAEWTKDRAGYKDND-VPSVGII--------------

go9 :KIMQLCLYSNFSDNYENANKHEQASVEDVEKHIHKIVTMYIDGSNHMEHIINSMKNPALCLKNPAEWTKDRAGYKDND-IPSVGII--------------

ma10 :KIMQLCLYSNFNDNYENAKSHTQATPEEVEEHIQKIIDIYIKETNNMEHILNSLKNPALCLKDPTQWVKDRAGYKDID-SPSAGII--------------

ov7 :KIMQLCLYANFNDNYENAKTHPQASIEEVDKHIQKIIKTYINESNNMERIQYSLKNPALCLKDPGLWVKDRAGYKDND-VPSAGII--------------

yo5 :KIANLCLYLNFKDNYESAEKHKQTDVEHIEPHIQHIVKTFIHTNDNIEHMKNALKNPALCFQNPSEWVQDRLGYKENNEIPSVGII--------------

be5 :KISNLCLYLNFKDNYESAKNHKQTDVEHIESHIQHIVKTFIHANDNIEHMKNALRNPALCFQNPLEWVQDRLGYKENDEIPSVGII--------------

ch5 :KIDNLCLYLNFKDNYENAEKHDQTDAEHVETHIQHIIQMFIHANDNMEHMRNALKNPALCFQNPAEWVQDRLGYKENAEIPSVGVI--------------

fa8 :KIKMLCMFSNYKDNYENANNHRQAKVEIVEEHIHKIVESYINEENNMEHMKDLLKNPALCLKNPNQWVKDRAGFKDDD-KPSVGII--------------

re8 :KIKMLCMFSNYKDNYENANNHRQAKVEIVEEHIRKIVETYINEENNMEHMKDLLKNPALCLKNPNHWIKDRAGFKDDD-KPSVGII--------------

ga2 :KIKKLCLYSNFYDNYENARNHMQASVVEVEQHIRKIVKKFMNEKDNIEHMYDSLSNPALCLKDPGQWIRDRAGYKDID-IPSAGII--------------

ga3 :KIRQLCLYVNFHDTYENAKLHEQASVEKVQENIKNIIDKYTTDTIDMEKVKKSLNHPALCLQNPEDWITDRMGLDDLD-VPSSGVI--------------

392 :-------910-------920-------930-------940-------950-------960-------970-------980-------990------1000

392 :----------------------------------------------------------------------------------------------------

570 :----------------------------------------------------------------------------------------------------

vi1 :SADKENIIKGEHIA------------------------------------------------------------------------------AYRAVIDL

cy1 :ETDRRNVIKDMYIP------------------------------------------------------------------------------GYRAVIDL

fi1 :PRDKKNVFKDMHFP------------------------------------------------------------------------------GYRAVIDL

so1 :PRDKKNVFKDMHIP------------------------------------------------------------------------------GYRAVIDL

in1 :STDKKSVVKGMHIP------------------------------------------------------------------------------GYRAVIDL

hy1 :STDKESLVKGMHIP------------------------------------------------------------------------------GYRAVIDL

co1 :ATNKKNVIEDMHIP------------------------------------------------------------------------------GYRAAIDL

kn1 :LADKEGVIKDMHMP------------------------------------------------------------------------------GYRAVIDL

vi2 :DPPKGSQKIQTN--------------------------------------------------------------------------------GYDGIINF

cy2 :DTPKGSEKIQTN--------------------------------------------------------------------------------GYDGVIDF

fi2 :DTPKGSEKIQTK--------------------------------------------------------------------------------GYDGVINF

so2 :DTPKGSEKIQTN--------------------------------------------------------------------------------GYDGVIDF

in2 :DTPEGSEKIQTN--------------------------------------------------------------------------------GYNSVINF

hy2 :DTPNESEKTQTN--------------------------------------------------------------------------------GSNSVINF

co2 :DKLKGSKKVQTN--------------------------------------------------------------------------------GYDGVINF

go1 :TLKTASSNEQNN--------------------------------------------------------------------------------CYDGVLDF

go2 :KNKGTDSKNGEHG-------------------------------------------------------------------------------AYDIVINL

vi3 :LEA-----------------------------------------------------------------------------------------GEDGVVDL

vi4 :DGTTAFKQ------------------------------------------------------------------------------------DDDGTIDL

vi5 :-----FDE------------------------------------------------------------------------------------GEEDTSNS

cy3 :-----YVE------------------------------------------------------------------------------------TEGSTSNS

cy4 :DGTPAFKE------------------------------------------------------------------------------------DEDGIIDL

cy5 :-----YVE------------------------------------------------------------------------------------TEGSTSNS

fi3 :-----FDE------------------------------------------------------------------------------------SEDFNSYS

so3 :-----FEE------------------------------------------------------------------------------------SGDYISDW

in3 :--------------------------------------------------------------------------------------------SEDNTFDS

hy3 :--------------------------------------------------------------------------------------------NADSTSDA

co3 :-----FGE------------------------------------------------------------------------------------SEDDISDS

kn2 :-----FDE------------------------------------------------------------------------------------SEDDISGS

go3 :LKQ-----------------------------------------------------------------------------------------GMGSSEDL

vi6 :HMSDADEGQQSV--------------------------------------------------------------------------------PYDAVINL

cy6 :HMNETNGGKENV--------------------------------------------------------------------------------PYDAVINL

fi4 :HMNDTNGGKQNV--------------------------------------------------------------------------------PYDAVINL

so4 :HMNDTNGGKQNV--------------------------------------------------------------------------------PYDAVINL

fr1 :HMSHTNDGMQNV--------------------------------------------------------------------------------PYDAIINL

go4 :NYNTKIVRAQNS--------------------------------------------------------------------------------SYDDVINL

vi7 :NGESAHDHTHEQ--------------------------------------------------------------------------------GFDGVIDL

vi8 :NLSTYQE-------------------------------------------------------------------------------------GPDGVIDL

cy7 :GLSTYQV-------------------------------------------------------------------------------------GDDGIIDL

fi5 :CPSTYQE-------------------------------------------------------------------------------------GPDGIIDL

so5 :GLSTYQE-------------------------------------------------------------------------------------GADGIIDL

in4 :KLSTYEE-------------------------------------------------------------------------------------GADGIIDL

hy4 :KHSTYEE-------------------------------------------------------------------------------------GADGIIDL

co4 :CPSTYQE-------------------------------------------------------------------------------------GDDGIIDL

kn3 :CSSTYQE-------------------------------------------------------------------------------------GDDGIIDL

fr2 :CHSTYEE-------------------------------------------------------------------------------------GADGIIDL

go5 :KGYTFQE-------------------------------------------------------------------------------------DKDGIIDL

vi9 :HVDDSSEDVQSG--------------------------------------------------------------------------------GYDGVIDF

cy8 :NVEDTSEDTQSG--------------------------------------------------------------------------------GYDGVIDF

fi6 :HMNDTSEDAQSD--------------------------------------------------------------------------------GYDGVIDF

so6 :HMNDTSEDAQID--------------------------------------------------------------------------------GYDGVIDF

go6 :SNNNINEDEPNG--------------------------------------------------------------------------------HFNGVIDL

ma1 :VESKNVNSVKQNE-------------------------------------------------------------------------------GYDGVIDF

ma2 :VNQNNIKDTFND--------------------------------------------------------------------------------GIDGIIDL

ma3 :YKNVGNSIYKE---------------------------------------------------------------------------------DDDGVVDL

ma4 :NKTGTENTE-NG--------------------------------------------------------------------------------NYDGVINL

ma5 :SNNTSRNIYKE---------------------------------------------------------------------------------GNNEIIDL

ma6 :HKNGYGHIDKE---------------------------------------------------------------------------------GDNNKGDL

ma7 :VNQNNIKDTFND--------------------------------------------------------------------------------GIDGVIDL

ov1 :YKG-----------------------------------------------------------------------------------------NCDGTVDL

ov2 :NVNEDMLKE-----------------------------------------------------------------------------------DDNGIIDL

ov3 :--------------------------------------------------------------------------------------------GEFGISNL

ov4 :KKIVTEGTSKA---------------------------------------------------------------------------------GYHGVIDL

yo1 :KLDEKLEEE-----------------------------------------------------------------------------------GFDGVIDL

be1 :HESDKKLQE-----------------------------------------------------------------------------------GFDGVIDL

ch1 :--DEKLKDE-----------------------------------------------------------------------------------GFDGVIDL

yo2 :-----IYEE-----------------------------------------------------------------------------------GPDGIIDL

be2 :-----NYEE-----------------------------------------------------------------------------------GLDGIIDL

ch2 :-----KFEE-----------------------------------------------------------------------------------GPDGIVDL

fa1 :IYDNKDDS------------------------------------------------------------------------------------KISDIINI

re1 :IYDNKDDS------------------------------------------------------------------------------------KISDIINI

fa2 :E-------------------------------------------------------------------------------------------DENGIVDL

re2 :E-------------------------------------------------------------------------------------------DENGIVDL

fa3 :DMFKA---------------------------------------------------------------------------------------NEHGIVDL

fa4 :--------------------------------------------------------------------------------------------TSQNIVDK

re4 :--------------------------------------------------------------------------------------------TSQNIVDK

fa5 :DN------------------------------------------------------------------------------------------KGKGVVHV

re5 :DN------------------------------------------------------------------------------------------KGKGVVHV

fa9 :DE------------------------------------------------------------------------------------------EYDGVIDL

re9 :DE------------------------------------------------------------------------------------------QYDGVIDL

ga1 :KIINVTEDMYEKDEN--------------------------------------------------------------------------------DIIDL

vi10 :EEEEHAGEGKLLDQDMYKKDED-------------------------------------------------------------------------GTIDL

cy9 :EEEKQVEENILLDQDMYRKDDD-------------------------------------------------------------------------GTIDL

fi7 :EEEKHVDENELLNQDMYRKDED-------------------------------------------------------------------------GTIDL

so7 :EEEKHIDENELLNQDMYRKDED-------------------------------------------------------------------------GTIDL

in5 :EEQEHLGQNKLLDQEMYRKDED-------------------------------------------------------------------------GTVDL

hy5 :EEQEHGGENKFLDEEMYRKDED-------------------------------------------------------------------------GTVDL

co5 :EEEGHVKESELLNQDMYRKDED-------------------------------------------------------------------------GTIDL

kn4 :EEEDHVKESELLNQDMYRKDKD-------------------------------------------------------------------------GTIDL

fr3 :GDEKHVKQSELFNQDMYRKDED-------------------------------------------------------------------------GTIDL

go7 :EEEDNYKENNYLHEDMYRSDDD-------------------------------------------------------------------------GVIDL

ma8 :DDEYKNGNYLNEDIYRKDGD---------------------------------------------------------------------------GVIDL

ov5 :E--YQDDKHLNDDIYRNDED---------------------------------------------------------------------------GVVDL

yo3 :RVDLLELFDDNQDEN-IVDKD--------------------------------------------------------------------------GIIDM

be3 :RVDLLELFDDNQNEN-IVDKD--------------------------------------------------------------------------GIIDM

ch3 :KVNLLDLFDNDQDED-VVDKD--------------------------------------------------------------------------GIIDM

fa6 :NEDKDDMSKNSKEEFKNDDKENSDDQNNNDSNKKDDENNINNGDTNYVYDFDDDDYDNNSYEKDMYESPIKENKN--------------------GVIDL

re6 :KEDKDDMSKNSKEDFKNDDKEYSDDQNNNDSNKKDDENNINNGDTNYVYDFDDDDYDNNNYEKDMYESVIKENKN--------------------GVIDL

vi11 :LKEIEKEKSALNDEAFAK-DTN-------------------------------------------------------------------------GVIHL

cy10 :LKEIEKEKNACDDDAFDK-DTN-------------------------------------------------------------------------GVIDL

fi8 :LKEIEKEKSVCDDDAFDK-DTN-------------------------------------------------------------------------GVIDL

so8 :LTEIEKEKSICDEDAFVK-DTN-------------------------------------------------------------------------GVIDL

in6 :LQEIEKEKSVYDDDVFDK-DTN-------------------------------------------------------------------------GIINL

hy6 :LQEMEEERRVYDDNLFDK-DTN-------------------------------------------------------------------------GIINL

co6 :LTEIEKEKSVYDDDVFDK-DTN-------------------------------------------------------------------------GVIDL

kn5 :LKELEKEKSVFDDDVFDK-DKN-------------------------------------------------------------------------GVIDL

fr4 :LKDIEEEKNVFDDEAFEK-DKN-------------------------------------------------------------------------GVIDL

go8 :FKEFEKENS-ISDEAFEK-DEN-------------------------------------------------------------------------GIIDL

ma9 :GRAESDTTTRGASDSGSADELMAENFEEEEIVSDDIFEKDSS-----------------------------------------------------GIIDL

ov6 :DEVDSAHYDADEVDRAHYDVNEVDRAHYDVDEVDDTHYDADEVDSAHYDADEVDRAHYDVNEVEDAHYDVENARSIDAGDDITDGAFERDEN---GIINL

yo4 :AFNEEDTEAIGNDIQNNEIDDE-------------------------------------------------------------------------NEIIN

be4 :TYNEEDTEEIGNDIQNNEIDNE-------------------------------------------------------------------------NEIMD

ch4 :KYNEENTEAVGNDIIENESDNE-------------------------------------------------------------------------NEIVN

fa7 :EESEYDYERFISDDMFEK-DMN-------------------------------------------------------------------------GVIDL

re7 :EESEYDYEKLISDDMFEK-DMN-------------------------------------------------------------------------GVIDL

vi12 :-----------------------------------------------------------------------------------------------PERKL

cy11 :-----------------------------------------------------------------------------------------------PERKL

fi9 :-----------------------------------------------------------------------------------------------PERKL

so9 :-----------------------------------------------------------------------------------------------PERKL

in7 :-----------------------------------------------------------------------------------------------PERKL

hy7 :-----------------------------------------------------------------------------------------------PERKL

co8 :-----------------------------------------------------------------------------------------------PERKL

kn6 :-----------------------------------------------------------------------------------------------PERKL

fr5 :-----------------------------------------------------------------------------------------------PERKL

go9 :-----------------------------------------------------------------------------------------------PERKL

ma10 :-----------------------------------------------------------------------------------------------SEKKL

ov7 :-----------------------------------------------------------------------------------------------SEKKL

yo5 :-----------------------------------------------------------------------------------------------PEKKL

be5 :-----------------------------------------------------------------------------------------------PEKKL

ch5 :-----------------------------------------------------------------------------------------------PEKKL

fa8 :-----------------------------------------------------------------------------------------------PERKI

re8 :-----------------------------------------------------------------------------------------------PERKL

ga2 :-----------------------------------------------------------------------------------------------PEKKL

ga3 :-----------------------------------------------------------------------------------------------NHNEI

392 :------1010------1020------1030------1040------1050------1060------1070------1080------1090------1100

392 :---------------------############-##################################################----------------

570 :---------------------############-###################################################---------------

vi1 :SQRD--QMN-HSNLSGEM---FCNEDYCDRWKD-KSSCFSSIETEEQGNCNLSWLFTSKTHLETIRCMKGYDHLGSSALYVASCSKRGK--KS-------

cy1 :SQKS--EMN-HSNPSGEM---FCNEDYCDRWKD-KNSCFSSIETEEQGNCNLSWLFASKTHLETIRCMKGYDHLGSSALYVANCSKRGN--KS-------

fi1 :SQKS--EMN-HSNLSGEM---FCNEDYCDRWKD-KNSCFSNIEAEEQGNCNLSWLFTSKTHLETIRCMKGYDHLGSSALYVANCSMRGS--KS-------

so1 :SQKS--GMN-YSNLSGDM---FCNEDYCDRWKD-KNSCFSNIETEEQGNCNLSWLFTSKTHLETIRCMKGYDHLGSSALYVANCSKRGS--KS-------

in1 :SQKS--EMN-HSNLSGDM---FCNEDYCDRWKD-KNSCFSSIEAEEQGNCNLSWLFTSKTHLETIRCMKGYEHVGSSALYVANCSKRGN--ES-------

hy1 :SQKN--EMD-HSNLSGDM---FCNEDYCDRWKD-KNSCFSSMEAEEQGNCNLSWLFTSKTHLETIRCMKAYDHVGSSALYVANCSKRAS--ES-------

co1 :SQKG--KVS-HSDLSGGM---FCNEDYCDRWKD-KNSCFSHIETEEQGNCNLSWLFASKLHLETIRCMKGYDHLSSSALYVANCSERGN--KS-------

kn1 :SQKG--GVN-HSDHSGEM---FCNEDYCNRWKD-RNSCFARIETEEQGNCNLSWLFASKLHLETIKCMKGYDHVATSALYVANCSSRGN--KS-------

vi2 :DSNEETNMQ-STSFIDNM---YCNDEYCDRWKD-SSSCVAKIEVEDQGACSNSWLFASKVHLESMKCMNGHDHMATSALYVANCSGKEE--KD-------

cy2 :VSREETNMQ-STSFIDNM---YCNVEYCNRWKD-PSSCMAKIEAGDQGDCATSWLFASKVHLETIKCMKGHDHIASSALYVANCSSKEA--KD-------

fi2 :VSREETNMQ-STSFIDNM---YCNDEYCNRWKD-ASSCMAKIEAGDQGDCATSWLFASKVHLETIKCMKGHDHVASSALYVANCSNKEA--KD-------

so2 :VSREETNMQ-STSFIENM---YCNDEYCNRWKD-ESSCMAKIEAGDQGVCATSWLFASKVHLETIKCMKGYDHVASSALYVANCSSKEA--KD-------

in2 :VSREETNMQ-STSFIDNM---FCNDEYCDRTKD-TNSCMAKIEAEDQGVCATSWVFASKMHLETIKCMKGHEHVPSSALYVANCSNKDA--KD-------

hy2 :ESREETNMQ-STSFIDSM---FCNDEYCDRTKD-TSSCMAKIQAEDQGDCATSWLFASKMHLETIKCMRGHDHVASSALYVVNCSNNEQ--ED-------

co2 :LSHEKTNMQ-STSFIDSM---YCNDEYCNRWKDDTSSCLAKIEPQDQGDCATSWLFASKLHLETIKCMKGYEHVPSSALYVANCSNKETP-TD-------

go1 :VSGEESNMQ-STHFTDSM---FCNTEYCDRWKN-KNDCLAKIEVEEQGNCATSWLFTSKLHLETIKCMKGYDHVPSSALYVANCSQKQG--DA-------

go2 :NPAGVGSHQ-SPSFADSM---YCNDIYCDRWKD-KNGCLAKIESADQGDCATSWIFASKLHLESIKCMKGYDHVPSSALYVANCSQKEG--DA-------

vi3 :TSLQSVDVS-PFLVTDKL---FCNDDYCDRAKD-TSSCVAKIEVQDQGDCATSWLFASKVHLETIKCVKGYDHVGASALYVANCSGTEA--ND-------

vi4 :TNLDCVDVH-PSVLADKM---HCNDDYCDRAKD-TSSCMAKIEVEDQGDCATSWLFASKMHLETIKCMKGYDHVASSALYVANCSDKEE--SD-------

vi5 :TTTQSADVH-PLNVSDKL---FCNDEYCDRAKD-SSSCIAKIEAGDQGDCSTSWLFASKVHLEAIKCMKGHDHVASSALYVANCSGKEA--ND-------

cy3 :ATTQSADVH-LLHVSDKL---FCNADYCDWTKD-TSSCIAKIEAQDQGDCATSWLFASKVHLETIKCVKGYEHVPSSALYVANCSSKEA--KD-------

cy4 :TTLDCVDVH-PSVLADKL---HCNDEYCDRWKD-SSSCMAKIEAGDQGDCATSWLFASKVHLETIKCVKGYEHVPSSALYVANCSSKEA--KD-------

cy5 :ATTQSADVH-LLHVSDKL---FCNADYCDWTKD-TSSCIAKIEAQDQGDCATSWLFASKVHLETIKCVKGYEHVPSSALYVANCSSKEA--KD-------

fi3 :TITQSAHVH-SSNIFDKL---FCNYDYCDWTKD-SSSCIAKIEAGDQGDCATSWLFASKVHLETIKCVQGYDHVASSALYVANCSNNEA--KD-------

so3 :TTTQSANVH-PLHVSDKL---FCNDDYCDWTKD-SSSCIAKIEAGDQGDCATSWLFASKVHLETIKCMKGYDHVSSSALYVANCSNKEA--ND-------

in3 :TTLQSVDVH-PLHVKDKL---FCNGDYCDRTKD-TSSCMAKIEAGDHGMCATSWLFASKLHLETIKCMKGHEHVPSSALYVANCSNKDA--KD-------

hy3 :TTLQTLDVN-PLHVKDKL---FCNEDYCDRTKD-TSSCMAKIEAEDQGDCATSWVFASKMHLETIKCIKGYEHVPTSALYVANCSNKDA--KD-------

co3 :TTTEGADSY-PLHFSDKL---FCNNDYCDRTKD-TSSCFAKIEPQDQGDCATSWLFASKLHLETIKCMKGYDHVPSSALYVANCSNKETP-TD-------

kn2 :TTTEGADLY-PLHFSDKL---FCNHDYCDRTKD-TSSCLSKIEVQDQGNCATSWLFASKLHLETIKCMKGYKHIPSSALYVANCSNKETLNKD-------

go3 :TTLENVDIQ-PIKVTDTL---FCNDEYCDRTKD-TSSCMSKIETEDQGICATSWIFASKLHLESIKCVKGYAHVPSSVLYVANCSQKES--DA-------

vi6 :VSAEEKNNQ-SSLIEDST---FCTEDYCNRWKD-DTSCVSKIEAEDQGVCSTSWLFASKVHLETIKCMKGYDHIATSALYVANCSSKEA--KD-------

cy6 :VSAEEKNNN-SSLIEDSM---YCTEEYCNRWKD-DTSCVSKIEAEDQGVCATSWLFASKVHLETIKCMKGHDHVASSALYVANCSNKEA--KD-------

fi4 :VSAEEKNNH-SSLIEDSM---YCTEEYCNRWKD-DTSCVSTIEAEDQGVCATSWLFASKMHLETIKCMKGHDHIASSALYVANCSNKEA--KD-------

so4 :VSAEEKSNH-SSFIEDSM---YCTEEYCNRWKD-DTSCVSKIEAEDQGVCATSWLFASKMHLETIKCMKGYDHVSSSALYVANCSNKEA--KD-------

fr1 :VSAEEKNNG-SSLIEDSM---YCNDEYCDRWKD-DTSCMSKIEAEDQGVCATSWLFASKLHLETIKCMKGYEHIPSSALYVANCLNNKA--HD-------

go4 :VSAEERKNY-NVIIEDTM---YCNDEYCDRWKD-DSSCISKIEAEDQGNCATSWLFASKLHLETIKCMKGYDHVPTSALYVANCSHKKG--KE-------

vi7 :PPFGTSGAQ-ASVYSDSM---YCNGDYCDRAKD-SSSCMAKIEAGDQGDCANSWLFASKAHLEAIQCMKGHDHVGASALYVANCSGKEA--KD-------

vi8 :SVVHQNAHASSTPFTNHM---FCNSDYCDRAKD-TSSCMAKIEVQDQGDCSTSWLFASKVHLEAIKCMKGHDHVGASALYVANCSNKEA--KD-------

cy7 :SIEQKNSHTSSTPFTNHM---FCNADYCDWTKD-SSSCMAKIEAGDQGDCATSWLFASKVHLETIKCMKGYDHIASSALYVANCSSKEA--KD-------

fi5 :SIMHKNANTSSTPFTNHM---FCNVDYCDWTKD-TSSCMSKIEAGDQGDCATSWLFASKVHLETIKCMKGHNHVSSSALYVANCSNKEA--MD-------

so5 :SVVQKNAHASSTPFTNHM---FCNVDYCDWTKD-TSSCMSKIEVGDQGECATSWLFASKVHLETIKCMKGYNHIASSALYVANCSNKEA--KD-------

in4 :SIVHKNVHASSIPFSNNM---FCNGHYCDRTKD-TNSCMAKIEAGDQGACTTSWVFASKVHLETIKCMKGYEHVPSSALYVANCSNKDA--KD-------

hy4 :SVVHKNAHASSTQFSNPM---FCNRDYCDRTKD-TTSCMAKIEAGDQGLCATSWVFASKVHLETIKCMKGYEHVPSSALYVANCANKDA--KD-------

co4 :SVVHKNAHAPSTPFTNHM---FCNYDYCDRTKD-TSSCLAKIETQDQGDCATSWLFASKIHLETIKCMKGYEHVPSSALYVANCSNNETP-TD-------

kn3 :SVVHKNAHASSTPFTNHM---FCNYDYCDRTKD-TSSCLSKMEVQDQGNCATSWLFASKLHLETIKCMKGYKHIPSSALYVANCSNKETLNKD-------

fr2 :SIVQTNPHASSTPFTNHM---FCNYDYCDKTKD-TSSCMSKIEAEDQGVCATSWLFAAKLHLETIKCMKGYEHIPSSALYVANCLNNKA--HD-------

go5 :SVIGKNSNASSTPFTNHM---FCNTEYCDRSKD-KSSCLSKIEAEEQGTCATSWIFASKLHLESIKCMKGYDYVPSSALYVANCSQKEG--DA-------

vi9 :ATAGKTNFST-SQYADKM---HCNAEYCDRAKD-AGSCVAKMEVQDQGDCANSWLFASKVHLETIKCVKGYDHVGASALYVANCSGKEA--ND-------

cy8 :STASKTNFST-SQYVDKM---HCNGEYCDRTKD-TSSCIAKIKAGDQGDCATSWLFASKVHLETIKCMKGHDHVASSALYVANCSGKEA--KD-------

fi6 :ATASKTNFST-SQYADKM---HCNLEYCDRWKD-DTSCVSKIEAGDQGDCATSWLFASKMHLETIKCMKGHDHVASSALYVANCSSNEA--KD-------

so6 :AMASKTNFST-SQYADKM---HCNMEYCDRWKD-DTSCVSKIEAGDQGDCATSWLFASKVHLETIKCMKGHDHVASSALYVANCSNKEA--KD-------

go6 :ETAEKTNFSP-SQFADKM---YCNAEYCDRTKE-ENGCISKINVEDQGNCATSWIFASKLHLESIKCMKGYDHIPSSALYVANCSQKEG--DA-------

ma1 :SDYNGTNMN-SSQLTDTM---YCNKEYCDRWKD-NDSCFSKIEAAEQGNCAISWLFASKLHLETIRCMNRYDHVKSSALYIANCSKRNP--KD-------

ma2 :EAVEQDNKQ-SFYFTDNM---FCNDEYCDRWKD-KNTCISKIEAQDQGSCATSWIFASKLHLETIRCMKGYEHASSSALYVANCAHKEA--KD-------

ma3 :TKYEEADMN-TARFTDRM---YCNEDYCDRWKD-KNSCVSKIEAEDQGNCATSWLFASKLHLETIRCMKGYDHASGSALYVANCSKIEP--DE-------

ma4 :VSLEESNVN-TSHVTDTM---YCNEDYCDRWKD-KNSCVSKIETKDQGNCATSWIFASKVHLETIRCMKGYDHASISALYVANCANKEA--MD-------

ma5 :TDNKNKNVD-ASHFTDSM---FCNDEYCDRLKD-KNSCVSKIEVQDQGNCSISWIFASKLHLESIKCMKGYEHGSISALYVANCANKEA--MD-------

ma6 :TNVDKDSKVLSIPVTNGM---FCNEEYCDRWKD-KNSCVSKIEAQDQGSCATSWIFASKLHLETIRCMKGYEHASSSALYVANCANKEA--KD-------

ma7 :EAVEQDNKQ-SYYFTDSM---FCNDEYCDRWKD-KNTCISKIEAQDQGNCATSWIFASKLHLETIRCMKGYEHASSSALYVANCANKEA--KD-------

ov1 :ETVANVDMS-SSHFVEKM---FCNEEYCDRWKD-KNGCFSKIGASDQGNCATSWIFASKMHLETIKCMKGYDHVSSSALYVANCSEKDV--KE-------

ov2 :TTGGNVTIN-PFNFTNNI---HCNDEYCDRSKD-KNSCISKMVPEDQGDCGISWIFASKLHLETSMCMKGYYPFQSSALYIANCSHIDP--KQ-------

ov3 :INETKDIND-LHPPITDM---FCNAEYCDRWKD-KNGCFSKIGASDQGNCATSWIFASKMHLETIKCMKGYDHVSSSALYVANCSEKDV--KE-------

ov4 :KPVDMKNAH-PSHIADRM---YCNEEYCDRWKD-KNGCFSKIGASDQGNCATSWIFASKMHLETIKCMKGYDHVSSSALYVANCSEKDV--KE-------

yo1 :PLPHENEFEGYTTIADFQ---YCNNEYCDRLKD-NNSCISKIDVEEQGNCATSWLFASKFHLDTIGCMKGHENFSASALYVANCSKKD--SKD-------

be1 :PLPHENEFAGYTTIEDLQ---YCNDEYCDRLKD-NNSCISKIDVEEQGNCATSWLFASKFHLDTIGCMKGHENFSASALYVVNCSKKD--SKD-------

ch1 :PLPHENEFAGYTTIEDFQ---YCNNEYCDRLKD-KNSCISKLNVEEQGNCATSWLFASKLHLDTIGCMKGHENFSASALYVANCSKTD--SKD-------

yo2 :TKTIDGNTVSGLGVLANKLDAFCNNDFCDRLKD-NNSCISNIDVEEQGNCATSWIFASKFHLETAICMKGHDNFTTSALYVANCSKKD--PKD-------

be2 :TKTIDDEPVSPLGLLANKLDAFCNNEFCDRLKD-KTSCVSKIDVEEQGNCATSWIFASKYHLETAICMKGHDNFNTSALYVANCSKKD--PKD-------

ch2 :TKTVDGNPITTTAVLANKLDAFCNNEFCDRLND-KTSCISNINVEEQGNCATSWVFASKLHLESSICMKGYDHTSASALYVANCSKKD--SKD-------

fa1 :KKYIFTNNTLKYFNNDKQ---FCNSSFCNRLKD-ENNCISKIQIEDQGNCAISWIFASKYYLETLKCMKGYEPHAISALYIANCSKRK--HKN-------

re1 :KKYIFTNNTLKYFNNDKQ---FCNSSFCNRLKD-ENNCISKIQVEDQGNCAISWIFASKYYLETLKCMKGYEPHAISALYIANCSKRK--HKN-------

fa2 :TKFPVDTSYSSYNYADSL---YCNREYCNRLKD-HNNCISKINVEDQKNCALSWAFASIYHLETIKCMKGYEPLNASVLYVTNCLKNK--NND-------

re2 :TKFPVDTSYSSYNYADSL---YCNHDYCNRLKD-HNNCISKINVEDQKNCALSWAFASKYHFETIKCMKGYEPINTSALYVANCLKNK--NKD-------

fa3 :TKFPIDTNYSSYKHIDHT---YCNNDYCNWSKD-KNSCISKINVEDQKNCALSWAFASKYHLETIKCMKGYEHIPISSLYIANCSKNE--KKD-------

fa4 :L--------------------YCNHEYCNRLKD-HNNCISKINVEDQKNCALSWAFASKYHLETIKCMKGYEPLNASVLYVTNCLKNK--NKD-------

re4 :L--------------------YCNHDYCNRLKD-HNNCISKINVEDQKNCALSWAFASKYHLETIKCMKGYEPLNASVLYVANCLKNK--NKD-------

fa5 :DTTLEKEDTLSYDNSDNM---FCNKEYCNRLKD-ENNCISNLQVEDQGNCDTSWIFASKYHLETIRCMKGYEPTKISALYVANCYKGE--HKD-------

re5 :DTTLEKEDSSSYDNSDNM---FCNKEYCNRLKD-ENNCISNLQVEDQGNCDTSWIFASKYHLETIRCMKGYEPTKISALYVANCYKGE--HKD-------

fa9 :STNENGNVK-SIQFNDDI---YCNDDYCNRWKN-ENNCISKIGVEDQGNCASSWAFASKLHFETLRCMKGYDHLGISALYLTNCSKGK--ISK-------

re9 :STNENGNVK-SIHFNDDI---YCNDDYCNRWKN-ENNCISKIGVEDQGNCASSWAFASKLHFETLRCMKGYDHLGISALYLTNCSKGK--ISK-------

ga1 :TKKDKEVKLLSPYFIPNK---YCNDEYCDRWKD-NTSCISKIEVEEQGHCGVCWVFASKLHLETIRCMRGYGHFRSSALYVANCSKRK--AKY-------

vi10 :VKAGKELKLRSPYFKNSK---YCNYEYCDRWKD-KTSCISNIEVEEQGNCGLCWVFASKLHLETIRCMRGYGHFRSSALYVANCSKRN--PKD-------

cy9 :VKAGKELKLRSPYFKNSK---YCNYEYCDRWKD-KTSCFSNIEVEDQGNCGLCWIFASKLHLETIRCMRGYGHFRSSAMYVANCSKRN--PKE-------

fi7 :VKAGKELKLRSPYFKNSK---YCNYEYCDRWKD-KTSCISNIEVEEQGNCGLCWVFASKLHLETIRCMRGYGHFRSSPLYVANCSRRN--PKD-------

so7 :VKAGKELKLRSPYFKNSK---YCNYEYCDRWKD-KTSCISNIEVEEQGNCGLCWVFASKLHLETIRCMRGYGHFRSSPLYVANCSRRN--PKD-------

in5 :VKAGKELKLRSPYFKNSK---YCNYEYCDRWKD-KTSCISSIEVEDQGNCGLCWVFASKLHLETIRCMRGYGHFRISALYVANCSKRN--RKQ-------

hy5 :VKAGKELKLRSPYFKNSK---YCNYEYCDRWKD-KTSCISSIEVEEQGNCGVCWVFASKLHLETIRCMRGYGHFRSSALYVANCSKRN--KKE-------

co5 :VKAGKELKLRSPYFKSSK---YCNYEYCDRWKD-KTSCISNIEVEEQGNCGLCWVFASKLHLETIRCMRGYGHFRSSALYVANCSRRN--PKD-------

kn4 :VKAGKELKLRSPYFKNSK---YCNYEYCDRWKD-KTSCISNIEVEEQGNCGLCWIFASKLHLETIRCMRGYGHFRSSALYVANCSRRN--RKD-------

fr3 :VKAGKELKLRSPYFKNSK---YCNYEYCDRWKD-KTSCISNIQVEEQGNCGVCWVFASKLHLETIRCMRGYGHFRSSALFVANCSKRN--RKD-------

go7 :VKAGKELKLRSPYFKNSK---YCNYEYCDRWQD-KTSCISNIEVEEQGNCGLCWVFASKLHLETIRCMRGYGHFRSSALFVANCSKRK--KKD-------

ma8 :VKAGKELKLRSPYFKNSK---YCNKEYCDRWKD-KTSCISNIEVEEQGNCGLCWVFASKLHLETIRCMRGYGHFRSSALFIANCSKRK--PKH-------

ov5 :AKAGKEIKLRSPYFKSSK---YCNYEYCDRWKD-KTSCISNIEVEEQGNCGLCWVFASKLHLETIRCMRGYGHFRSSALYVANCSKRK--PKD-------

yo3 :SMAIKYAKLKSPYFTSSK---YCNYEYCDRWQD-KTSCISNIDVEEQGNCSLCWLFASKLHIETIRCMRGYGHNRASALYVANCSERT--GEE-------

be3 :SIAIKYAKLKSPYFNSSK---YCNYEYCDRWQD-KTSCISNIDVEEQGNCSLCWLFASKLHLETIRCMRGYGHNRSSALYVANCSKRT--AEE-------

ch3 :SLAIKHAKLKSPYFNSSK---YCNYDYCDRWQD-KTSCISNIDVEEQGNCSLCWLFASKLHLETIRCMRGFGHNRGSALYVANCSERK--GEQ-------

fa6 :EKYGNQIKLKSPYFKNSK---YCNYEYCNRWRD-KTSCISQIEVEEQGNCGLCWIFASKLHFETIRCMRGYGHFRSSALYVANCSKRK--PID-------

re6 :EKYGNQIKLKSPYFKNSK---YCNYEYCNRWRD-KTSCISQIEVEEQGNCGLCWIFASKLHLETIRCMRGYGHYRSSALFIANCSKRK--PID-------

vi11 :DKPPNEMKFKSPYFKKSK---YCNNEYCDRWKD-KTSCMSNIEVEEQGDCGLCWIFASKLHLETIRCMRGYGHFRSSALFVANCSKRK--PED-------

cy10 :NKIPGEMKFKSPYFKKSK---YCNNEYCDRWKD-KTSCISNIEVEEQGDCGLCWIFASKLHVETIRCMRGYGHFRSSALFVANCSKRS--PED-------

fi8 :NKIPSEMKFKSPYFKNSK---YCNNEYCDRWKD-KTSCISNIEVEEQGDCGLCWIFASKLHLETIRCMRGYGHFRSSALFVANCSKRS--PED-------

so8 :NKIPSEMKFKSPYFKNSK---YCNNEYCDRWKD-KTSCISNIEVEEQGDCGLCWIFASKLHLETIRCMRGYGHFRSSALFVANCSKRS--PED-------

in6 :NKIPSEMKFKSPYFKKSK---YCNNEYCDRWKD-KTSCISNIEVEEQGDCGLCWIFASKLHVETIRCMRGYGHFRSSALFVANCSKRK--SED-------

hy6 :SELPSEMKFKSPYFKKSK---YCNNEYCDRWKD-KTSCISNIEVEEQGDCGLCWIFASKLHVETIRCMRGYGHFRSSALFVANCSKRN---RE-------

co6 :NKVPSEMKFKSPYFKKSK---YCNNEYCDRWKD-KTSCISNIEVEEQGDCGLCWIFASKLHLETIRCMRGYGHFRSSALFVANCSKRD--PEE-------

kn5 :NKVPSEMKFKSPYFKKSK---YCNNEYCDRWKD-KTSCISNIEVEEQGDCGLCWIFASKLHLETIRCMRGYGHFRSSALFVANCSNRD--PGE-------

fr4 :NKVPSDMKFKSPYFKKSK---YCNDYYCDRWKD-KTSCISNIEVEEQGDCGLCWIFASKLHLETIRCMRGYGHFRSSALFVANCSKRD--PEE-------

go8 :NKVKNKMKLKSPYFNKNK---YCTNEYCDRWKD-KTSCISNIEVEEQGDCGLCWIFASKLHLETIRCMRGYGHFRSSALFVANCSKRK--PEE-------

ma9 :SKMKTEMNFKSPYYQKSK---YCNKDYCDRWKD-KTSCISNIEVEEQGDCGLCWIFASKLHVETIRCMRGYGHFRSSALFVANCSSRN--PEE-------

ov6 :RKFPRKMKLKSPYFKNSK---YCNNEYCDRWKD-KTSCISNIEVEEQGECGLCWIFASKLHLETIRCMRGYGHFRSSALFIANCSKRK--HED-------

yo4 :LKSSSNKKLTSAYFNSSR---YCNKDYCDRWQD-KTSCISNIEVEEQGECGICWIFASKLHLETIRCMRGYGHYRSSALYVANCSDRK--KSE-------

be4 :LKSLSNKKLASAYYTSSR---YCNKDYCDRWKD-KTSCISNIEVEEQGECGVCWVFASKLHLETIRCMRGYGHYRSSALYVANCSQRK--KSE-------

ch4 :LKNSSNKKLTSAYFNSSR---YCNKDYCDRWQD-KTSCMSNIEVEEQGECGVCWVFASKLHLETIRCMRGYGHYRSSALYVANCSERD--KEE-------

fa7 :SLFDNEKKLKSPYFRRNK---YCNNEYCDRWKD-KTGCISKIEVEEQGNCGLCWIFASKLHFETIRCMRGYGHFRSSALYVANCSDRD--SDE-------

re7 :SLFNNEKKLKSPYFRRNK---YCNNEYCDRWKD-KTGCISKIEVEEQGNCGLCWIFASKLHFETIRCMRGYGHFRSSALYVANCSDRD--SDE-------

vi12 :FKPYEVKSLMSSLYSYKS---NCNRQFCDRFSD-PNECEHSIRVLNQGTCGNCWAFASSQVISAYRCRKGLGFAEPSVKYVTLCRNRHSDDYEENP-LGH

cy11 :FKPYEVKSLKSSLYSYQS---NCNRQFCDRFSD-PNECENSIRVLNQGICGNCWAFASSQVISAFRCRKGLGFAEPSVKYVTLCKNKNADNFQENP-FGH

fi9 :FKPYEVKSLMSSLYSYKS---NCNRQFCDRFSD-PNECENSIRVLNQGTCGNCWAFASSQVISAFRCRKGLGFAEPSIKYVTLCKNKNADNFEENP-FGH

so9 :FKPYEVKSLMSSLYSYKS---NCNRQFCDRFSD-PNECENSIRVLNQGTCGNCWAFASSQVISAFRCRKGLGFAEPSIKYVTLCKNKNADNFEENP-FGH

in7 :FKPYQVKSLMSSLYSYKS---NCNRQFCDRFSD-PNECENSIRVLNQGTCGNCWAFASSQVFSAYRCRNGLGFAEPSVKYVTLCKNRHSDDYQENA-FHH

hy7 :FKPYKVKSLMSSLYSNNS---NCNRQFCDRFSD-PNECENSIRVLNQGTCGNCWAFASTQVISAYRCRNGLGFAEGSIKYVTLCKNRHSDDYQENP-FHH

co8 :FKPYEVKSLMSSLYSYSS---NCNRQFCDRFSD-PNECENSIRVLNQGTCGNCWAFASSQVISAYRCRKGLGFAEPSIKYVTLCKNKNADNFEENP-FGH

kn6 :FKPYEVKSLMSSLYNYSS---NCNRQFCDRFSD-PNECENSIRVLNQGTCGNCWAFASSQVISAYRCRKGLGFAEPSIKYVTLCKNKHSNDLEENP-FGH

fr5 :FRPYEVKSLMSSLYSYKS---NCNRTFCDRFSD-PNECESSIRVLNQGTCGNCWAFASSQVISAFRCRKGLGFAEPSIKYVTLCKNKHANDYEENP-FTH

go9 :FKPYQVKSLMSSLYSHNS---NCNRQFCDRFSD-PNECENKIRVLNQGTCGNCWAFASSQVISAYRCRKSLGFAEPSVKYVTLCKNKQLGDNDYSL-FGH

ma10 :FKPYKIKSLMSSLYSSKS---NCTMQFCNRFAD-SNECESKIRVLNQGTCGNCWAFASSTTISAFRCRKGLGFAEPSAKYVTLCKNKHINTDDGYV-SGH

ov7 :FKPYEIKSLMSSLYSSKS---NCNRQFCDRFSD-PNECEHNIRVLNQGTCGNCWAFASSTTIAAFRCRKGLGFSEPSVKYVTLCKNKFSMEWENNI-YGH

yo5 :FKPFKNKSLMSSLYNANS---KCNRTHCNRFAD-PNECEYNIRPLNQGTCGNCWAFASSTTVSAFRCRKGLGFAEPSIKYVTLCKNKYLVDDDSHI-FGH

be5 :FKPYTNKSLMSSLYNANS---KCNRTYCNRFSD-PNECEYNIRPLNQGTCGNCWAFASSTTISAYRCRKGLGFAEPSIKYVTLCKNKYLDDEDSQT-FGH

ch5 :FKPHKHSSLMNSLYNPNS---KCNRKYCNRFAD-PNECEHNIRPLNQGTCGNCWAFASSTTISAFRCRKGLGFAEPSIKYVTLCKNKYLVDEEPPV-FGH

fa8 :FKPYDIKTLKSSLYASST---NCDRQFCDRFSD-SNECEHRIRVLNQGKCGNCWVFASSVVIAAYRCRKGLGFAEPSIKYVTLCKNKHLMDIDNNP-FGH

re8 :FKPYDIKVLKSSLYASSS---NCDRQFCDRFSD-SNECEHRIRVLNQGKCGNCWVFASSVVIAAYRCRKGSGFAEPSIKYVTLCKNKHLMDIDNNP-FGH

ga2 :FKSYMLNSLNNSLYSSKY---NCNRQFCNRFAD-TNECEHNIRVLNQGKCGNCWAFASSTVISAYRCRKDLGFAEPSVKYVTLCKNKYNNNGIDL--FGH

ga3 :FESYDDHNLKNDFSDNNS---KCNRDYCNRFDD-TNECEYNIRVLNQGTCGNCWAFAASTVVSAYRCRSGLGFAEPSVKYTTLCKSKFFGGQSRRSYYPG

392 :------1110------1120------1130------1140------1150------1160------1170------1180------1190------1200

392 :----#####################################-##-################-------------##########----############

570 :----#####################################-##-################------------###########--##############

vi1 :----RCVSGSNPYEFLTIVEESGFLPPAVWLPYSYGDVGNG-CP-KREDHWHNLWEGVKLLEPAD-EPNSVSTKGYTSYESDDF--RGDIQTFVNMVKSQ

cy1 :----KCTSGSNPYEFLTIVEENGFLPPALLIPYSYADVGNG-CP-RKENHWQNLWANVKLLEPSD-EPNSVSTKGYTSYESDDF--RGNIDVFIDLVKRE

fi1 :----RCTSGSNPYEFLTIVEENGFLPPALLMPYSYGDVGNG-CP-KQEDHWQNLWENVKLLEPSD-EPNSVSTKGYTSYESDDF--RGNIEAFIDLVKRE

so1 :----RCTSGSNPYEFLTIVEENGFLPPALSVPYSYGNVGNG-CP-KQEDHWQNLWANVKLLEPSD-EPNSVSTKGYTSYESDDF--RGNIEAFVNLVKRE

in1 :----RCTSGSNPYEFLTIVEENGFLPPALLLPYSYRHVGND-CP-KQDDHWQNLWENLKLLEPSD-EPNSMSTKGYTSYESDDF--RGNMEAFVNLVKRE

hy1 :----RCTSPSNPYEFLTIVEENGFLPPASLLPYSYRQVRNE-CP-KHEGHSQNLWENVKLLEPSD-EANSVSTKGYTSYESDDF--KGNMEAFVNLVKRE

co1 :----KCTSASNPYEFLTIVEENGFLPPALSVPYSYGKVGNS-CP-KQEDHWQNLWANVKLLEPSN-DPNAVSTKGYTSYESDDF--RGNIETFVNLIKRE

kn1 :----KCISGSNPYEFLTIVEENGFLPPALLLPYSYGKVGNT-CP-KQQDHWQNLWSNVKLLEPSY-EPNSVSTKGYTSYESDDF--RGNIERFVNLVKRE

vi2 :----KCHVASNPLEFLDILEETQFLPAESDLPYSYKAVNNV-CP-QPKSHWQNIWADVKLLDKQD-DPNAVSAKGYAAYQSDHF--KGNMDAFIKLVKSE

cy2 :----KCQAPSNPLDFLNTLEETKFLPAESDLPYSYKAVNNV-CP-EPKNHWQNLWADVKLLDKQY-QPNSVSTKGYTAYQSDHF--KGNMDAFIKLVKSE

fi2 :----KCKAPSNPLQFLDILEESKFLPAESDLPYSYKQVGNV-CP-EPKSHWKNLWADVKLVDKQY-QPNSVSTKGYTAYESANF--EGNMDAFIKLVKSE

so2 :----KCQAPSNPLEFLDTLEETKFLPAESDLPYSYKAVNNV-CP-DPKSHWQNLWTNVKLLDKQY-QPNSVSTKGYTAYESANF--EGNMDAFIKLVKSE

in2 :----KCQVPSNPLEFLDILEETKFLPAESDLPYSYKSVNNV-CP-EPKSHWKNLWANVKLLNKQY-TPNSVSAKGYTAYESANF--EGNMDAFIKLVKSE

hy2 :----KCEALSNPLKFLDVLEETKFLPSESDLPYSYKSVNNV-CP-EPKSHWKNLWADVKLLDPQN-EPNSVSTKGYTAYQSDHF--KGNMDAFIKLVKSE

co2 :----KCHAASNPLEFLHTLEETKFLPTESNLPYSYKAVNNV-CP-EPKNHWQNLWANVKLLDPQY-QPNAVSTKGYTAYQSANF--NGNMDAFIKLVKSE

go1 :----KCHAASNPLEYLNILDGEKFLPSASDMPYSYKLVGDV-CP-KPMNHWTNLWNNIKLLNHEN-VPNSVGTKGYTAYQSEHF--KNNMDEFIKIVKSE

go2 :----KCLAASNPLEFLNIIDGEKFLPSASDMPYSYKLVGDA-CP-KPKHHWANLWNNIKLLDPAN-EPNSLSTKGYTSYQSAQF--KDKMDEFINLVKAE

vi3 :----KCHAASNPLEFLNTLEETKFLAAESDLPYSYKAVNNA-CP-EPKSHWKNLWENVKLLDPTN-EPNSVSTKGYTAYQSDHF--KGNMDAFIKLVKSE

vi4 :----KCQAASNPLEFLDILEETKFLPAESDLPYSYKQMGNA-CP-EPKSYWQNLWADVKLLEKQY-EPNAVSTKGYTAYQSDHF--MANMDAFIKMVKSE

vi5 :----KCHAASNPLEFLNTLEETKFLAAESDLPYSYKAVNNA-CP-EPKSHWKNLWENVKLLDPTN-EPNSVSTKGYTAYQSDHF--KGNMDAFIKLVKSE

cy3 :----KCQAASNPLEFLNILEETKFLPAESDLPYSYKAVNNV-CP-EPKSHWQNLWENVKLLDKQY-QPNSVSTKGYTAYQSDHF--KGNMDAFIKLVKSE

cy4 :----KCQAASNPLEFLNTLEETKFLPAESDLPYSYKAVNNV-CP-EPKSHWQNLWENVKLLDKQY-QPNSVSTKGYTAYQSDHF--KGNMDAFIKLVKSE

cy5 :----KCQAASNPLEFLNILEETKFLPAESDLPYSYKAVNNV-CP-EPKSHWQNLWENVKLLDKQY-QPNSVSTKGYTAYQSDHF--KGNMDAFNKLVKSE

fi3 :----KCQALSNPLEFLHTLEETKFLPAESDLPYSYKEVGNV-CP-EPNNHWQNLWANVKLLGPTN-VPNSVSTKGYTAYESANF--EGNMDAFIKLVKSE

so3 :----KCHAPSNPLEFLNTMEETKFLPAESDLPYSYKAVNNV-CP-EPKSHWQNLWANVKLLDPQN-KPNSVSTKGYTAYESANF--EGNMDAFIKLVKSE

in3 :----KCQVPSNPLEFLDILEETKFLPAESDLPYSYKSVNNV-CP-EPKSHWKNLWANVKLLNKQY-TPNSVSAKGYTAYESANF--EGNMDAFIKLVKSE

hy3 :----KCQAPSNPVEFLDILEETKFLPAESDLPYSYKQVGDV-CP-DTKTHWKNLWANVKLLNKQN-GPNALNTKGYTAYESAKF--EGNMDAFTKLVKSE

co3 :----KCHAPSNPLEFLHTLEETKFLPTESNLPYSYKAVNNV-CP-EPKTHWQNLWANVTLLPPQY-QPNAVSTKGYTAYQSANF--NGNMDAFIKLVKSE

kn2 :----KCHTPSNPLEFLHTLEKTKFLPAESDLPYCYKKVGNDICP-EPKSHWKNLWTNIKLVEAQY-QPNSISTKGYTEYQSKYF--KGNMDAFIKLVKSE

go3 :----KCHTASNPLEFLNILDGEQFLPSASDMPYSYKLVGDV-CP-KPKHHWVNLWENVKLLNHKK-MPNTVGTKGYTAYQSAQF--KDNMDEFIKIVKSE

vi6 :----KCHAASNPLEFLDILEETQFLPAESNLPYSYKAVSNA-CP-EPKSHWQNLWENVKLLEKQY-EPNAVSTKGYTAYQSDHF--KGNMDAFINLVKSQ

cy6 :----KCQVPSNPLEFLNILEETKFLPAESNLPYSYKAVNNV-CP-EPKSHWQNLWENVKLLDKQY-QPNSVSTKGYTAYQSDHF--KGNMDAFIKLVKSE

fi4 :----KCQAPSNPLEFLDILEETKFLPAESDLPYSYKQVGNV-CP-EPKSHWQNLWANVKLLDPQN-EPNSVSTKGYTAYESANF--EGNMDEFIKLVKSE

so4 :----KCQAPSNPLEFLDILEGTKFLPAESDLPYSYKQVGNV-CP-EPKSHWKNLWANVKLLDPQN-EPNSVSTKGYTAYQSDHF--KDNMDAFIRLVKSE

fr1 :----KCQAPSNPLEFLNTLEETKFLPTNSNLPYSYKAVNNV-CP-QPNSHWKNLWANVTLLDPQY-QPNSVSTKGYTAYQSAHF--NGNMDAFIKLVKSE

go4 :----KCNTASSPLEFLKILDGEQFLPSSSDMPYSYKLVGDV-CP-KPKHHWTNLWNNIKLLEPTY-KPNSLSAKGYISYQSDKF--KNNIDQFINLVKSE

vi7 :----KCHVASNPLEFLNTLEETKFLPAESDLPYSYKAVNNV-CP-QPKSHWKNLWANVKLLEKQY-EPNAVSTKGYTAYQSDHF--KGNMDAFIKTVKSE

vi8 :----KCHSPSNPLEFLNTLEETKFLPAESDLPYSYKAVNNV-CP-EPKSHWKNLWENVKLLGPTN-EPNSVSVKGYTAYLSDHF--KGNMDAFIKSVKSQ

cy7 :----KCQAPSNPLEFLNILEGTKFLPAESDLPYSYKEVNNV-CP-EPKSHWKNLWENVKLLDKQY-QPNSVSTKGYIAYQSDHF--KGNMDAFIKLVKSE

fi5 :----KCQAPSNPLQFLDILEETKFLPAESDLPYSYKQVGNV-CP-EPKSHWQNLWADVKLLDPQN-EPNSVSTKGYTAYLSDHF--SGNMDAFIKLVKSE

so5 :----KCQDPSNPLEFLDILEETKFLPSDSDLPYSYQAVNNV-CP-EPKSHWQNLWANVKLLDKQY-QPNSVSTKGYTAYQSDHF--NGNMGTFIKLVKSE

in4 :----KCQVPSNPLEFLDILEETKFLPAESDLPYSYKSVNNV-CP-EPKSHWKNLWANVKLLNKQY-TPNSVSAKGYTAYESANF--EGNMDAFIKLVKSE

hy4 :----KCEVPSNPLEFLDILEKTKFLPSESDLPYSYQQVENV-CP-EPKSHWKNLWANVKLLDPQN-GPNSVSTKGYTAYQSDHF--KGNMDAFIKLVKSE

co4 :----KCHAPSNPLEFLHTLEETKFLPTESNLPYSYKAVNNV-CP-QPKTHWQNLWENVTLLAPQY-QPNAVSTKGYTAYQSANF--NGNMDAFIKLVKSE

kn3 :----KCHTPSNPLEFLHTLEETKFLPSESDLPYCYKKVGNDICP-EPKSHWQNLWTNIKLVDAQY-QPNSISTKGYTEYQSKYF--KGNMDAFIKLVKSE

fr2 :----KCQAPSNPLEFLNTLEETKFLPTNSNLPYSYKAVNNV-CP-QPNSHWKNLWANVTLLDPQY-QPNSVSTKGYTAYQSAHF--NGNMDAFIKLVKSE

go5 :----KCHAASNPLEFLNIIDSKQFLPSASDMPYSYKLVGDV-CP-KPKHHWTNLWNNIKLLNHKN-VPNSVGTKGYTAYQSEHF--KNNMDEFIKIVKSE

vi9 :----KCHSPSNPLEFLNTLEETNFLPADSDLPYSYKQVGNA-CP-EPKGHWQNLWENVKLLGPTN-EPNSMSTKGYTAYQSDHF--KGNMDSFIKLVKSE

cy8 :----KCQTPSNPLDFLNTLEETKFLPAESDLPYSYKEVNNV-CP-EPKSHWQNLWENVKLLDKQY-QPNSVSTKGYTAYQSDHF--KGNMDAFNKLVKSE

fi6 :----KCQAPSNPLEFLDTVEETKFLPAESDLPYSYKALNNV-CP-EPKSHWQNLWANVKLLDPQN-EPNSVSTKGYTAYQSDHF--NGNMDAFIKLVKSE

so6 :----MCHAPSNPLEFLNTMEETKFLPAESDLPYSYKAVNNV-CP-EPKSHWQNLWANVKLLDPQN-EPNSVSTKGYTAYQSDHF--NGNMNAFIKLVKSE

go6 :----KCNTASSPLEFLNILDGEHFLPSASDMPYSYKLVGDV-CP-KPKHHWTNLWNNIKLLNHKN-VPNSIGTKGYTAYQSEHF--KNNMDEFIKIVKSE

ma1 :----KCISGSNPFEYLSVIEENGFLPLEHNHPYSYKEVAND-CP-KSENHWTNLWAGAKLVDSKD-EPNSLGAKGYTAYESDKF--RGNIDTFIKKIKHE

ma2 :----KCHVGSNPLEFLKIIDENKFLPLEVNLPYSYAKVGNT-CP-NPQNHWTNLWANVELLDSKD-EPNSLGAKGYTAYESDKF--RGNMDTFIKKIKHE

ma3 :----RCKVGSNPLEFLRIIDEKKILPLESNLPYSYKNVAND-CP-NPKYHWTNLWTDTKLMDYKY-EPNSLGAKGYTAYESYKF--RGNMDTFIKKIKHE

ma4 :----KCHVGSNPLEFLKIIYENKFLPLEYNLPYEYAKVGNE-CP-NPQNHWTNLWANVELLDSKD-EPNSLGAKGYTAYESDKF--KGNIDAFINIIKHE

ma5 :----KYHVGSNPLEFLKIIDDKKFLPLEYNLPYAYAKVGND-CP-NPQKHWTNLWTGAKLVDSKN-EPNSLGAKGYTAYESDKF--RGNMDTFIKKIKHE

ma6 :----KCHVGSNPLEFLKIIDENKFLPLEVNLPYSYANVGNE-CP-NPQNHWTNLWANVELLDSKD-EPNSLGAKGYTAYESDKF--RGNMETFIKKIKHE

ma7 :----KCHVGSNPLEFLKIIDENKFLPLEVNLPYSYAKVGNA-CP-NPQNHWTNLWANVELLDSKD-EPNSLGAKGYTAYESDKF--KGNMDAFIGIIKKE

ov1 :----KCTVGSNPLEFLNIVNGKKFLPSEANLQYSYAKVSDD-CP-KSKSNWVNLWTGIKLLDYVP-TPNSVGTKGYTAYESAKF--KGNMDSFIKKVKSE

ov2 :----RCLVGSNPLEFLNIVKENKNLPLEENLPYSHTKVGNE-CP-QKNTNWVNLWGNIELLDAPN-DVNALGTKGYTAYESSKF--KENMDAFIKIVKNE

ov3 :----KCTVGSNPLEFLNIVNGKKFLPSEANLQYSYAKVSDD-CP-KPKSNWVNLWTGIKLLDYVP-TPNSVGTKGYTAYESEKF--KGNMDSFIKKVKSE

ov4 :----KCTVGSNPLEFLNIVNGKKFLPSEANLQYSYAKVSDD-CP-KPKSNWVNLWTGIKLLDYVP-TPNSVGTKGYTAYESEKF--KGNMDSFIKKVKSE

yo1 :----KCLTGSNPLEFLNIIDENKFLPTTSNLPYSYKKVGEE-CP-KAMDNWTNLWKDVKLLKHEN-NDKSLNANGYISYQSKDF--KDNFSEYINLIKQE

be1 :----KCLIGSNPLEFLNIIDENKFLPTTSNLPYSYKKVGEE-CP-KTMDNWTNLWKDVKLLKYEN-NDKSLNANGYISYQSEDF--KDNFSEYINLIKQE

ch1 :----KCLTGSNPLEFLNIIDENKFLPTTSNLPYSYKKVGEE-CP-QTMENWTNLWKDVKLLKSEN-NDKSLNANGYISYQSEHF--KENFNEYVNLIKQE

yo2 :----KCLTGSNPLEFLDILQANAYLPNESNYPYNYKNVGDS-CP-ETNESWVNLFDKVKLENSND-DTKSII-KGYTSYESKDY--KSNMNEFVDIIKNK

be2 :----KCLSGSNPLEFLDILQANNYLPTESNYPYNYKHVGDS-CP-ETNQTWVNLFDKLKLENSND-GNNSII-KGYTSYESKDY--KSNMNEFVGIIKNK

ch2 :----KCLAGSNPLEFIDILQTNNYLPTESNYPYNYKNVGDA-CP-ETNESWVNLFDKVKLEDSNK-DNKSII-KGYKSYESKNY--KTNMNEFVDIVKNN

fa1 :----RCNVGSNPLEFLQIIEENQFLPMDTNYLYSYTKVGND-CP-DEEKNWVNLLKHTRMLNYNNKHRSTLSTKAYRAYESEHF--KDKMDTFIKLIKDE

re1 :----RCNVGSNPLEFLQIIEENQFLPMDTNYLYSYTKVGND-CP-DEEKNWVNLLKHTRIINYNNKHRSTLSTKAYRAYESEQF--KDKMDTFIKLIKDE

fa2 :----VCTEGSNPLVFLETIEEKGFLPTESNYPYDQSKVGDV-CP-QVQNDWDNVFENTKVLEYNN-APFSVGTKGYIAYESEVF--QKDIDSFVKLIKDE

re2 :----VCTEGSNPLVFLETVEEKGFLPTESNYPYDQSKVGDI-CP-QVQNEWDNVFENTKVLAYNN-APFTVGTKGYIAYESEAF--QKDMHSFVKLIKDE

fa3 :----VCTEGSNPLKVLQMIVEKGFLPTEGDYSYEQSKVGET-CP-EVQNGWVNLWANAKLLEQNNDEHNSLSTKGYTAYESEAF--QKDMHSFVKLIKDE

fa4 :----VCTEGSNPLVFLETIEEKGFLPTESNYPYDQSKVGDI-CP-QLQNDWDNVFENTKVLDYNN-GPFSVGTKGYIAYESEAF--QKDMHSFVKLVKDE

re4 :----ICTEGSNPLVFLETVEEKGFLPTESNYPYDQSKVGDI-CP-QIQNDWDNAFENTKVLDYNN-APFSVGTKGYTAYESEHF--KDKMDTFIKLVKDE

fa5 :----RCDEGSSPMEFLQIIEDYGFLPAESNYPYNYVKVGEQ-CP-KVEDHWMNLWDNGKILHNKN-EPNSLDGKGYTAYESERF--HDNMDAFVKIIKTE

re5 :----RCDEGSSPMEFLQIIEDYGFLPTESNYPYNYVKVGEQ-CP-KVQDHWMNLWDNGKILYNKN-EPNSLDGKGYTAYESERF--HDNMDAFVKIIKTE

fa9 :----RCTEGGNPSEFLEIVSNSGFLPTEFDYPYFYALVENS-CP-SGLNKWINLWEDVKLFDYKY-EPNSIGSKGYSVYKSEMF--KDNMDEYVKMIKSE

re9 :----RCTEGGNPSEFLEIVSNSAFLPTEFDYPYFYALVENS-CP-SGLNKWINLWEDVKLLDYKY-EPNSIGSKGYSVYKSEMF--KDNMDEYVKMIKSE

ga1 :----RCIVGSSPIEFLQIIDETKFLPLESDHPYSYTDVGKD-CP-QPKKKWVNLWGSTKLLEYVN-YSQFTYSKGYIAYESAKY---TNIDTYIGIVKRE

vi10 :----ICTVGSNPIEFLKIVQDTGFLPLEADHPYLHKNAGNE-CP-APKENWINLWGNSKLLFHKM-YGHFMSYKGFISYESSHF--KDNMHIFIDLIKRE

cy9 :----ICTVGSTPLEFLKIVQDTGFLPLEADHPYLHKNVMNQ-CP-KPKENWINLWGNSKLLFHKL-YGHLMSYKGFISYVSLHF--RDNMHIFIDLIKRE

fi7 :----ICTVGSNPVEFLQILQETGFLPLEADHPYLHKNAGNE-CP-TPKENWINLWGNSKLLSNKL-YGHFMSYKGLISYETRHF--KDNMHIFIDLVKRE

so7 :----ICTVGSNPVEFLQILQETKFLPLEADHPYLHKNAGNE-CP-TPKENWINLWGNSKLLSNKL-YGHFMSYKGLISYESRHF--KDNMHIFIDLVKRE

in5 :----ICDVGSNPIEFLKIVQDTGFLPLESDHPYLHKNAENE-CP-QPKENWINLWGNSKLLFHKM-YGHLMSYKGFIAYRSPHF--RDNMHIFIDLIKRE

hy5 :----ICTVGSNPIEFLKIVQDTGFLPLESDHPYLHKHAENE-CP-KPKENWINLWGDSKLLFHKI-YGHLMSYKGFISYESMHF--RDNMHVFIDLIKRE

co5 :----ICAVGSNPIEFLQIVRDIGFLPLESDHPYLHTKAGNE-CP-KIKDNWINLWGNSKLLSHKM-YGHFMVYKGLISYQSRHF--KDNMHIFIDLVKRE

kn4 :----ICEVGSSPTEFLQIVRDTGFLPLESDHPYLHKNAGNE-CP-KIKENWINLWGNSKLLSHKM-YGHFMVYKGLISYQSRHF--KDNMHIFIDLVKRE

fr3 :----VCDVGSNPIEFLQIVRDTGFLPLESDHPYLHKNVINQ-CP-KIKENWVNLWGNTKLLSHKL-YGHFMMYKGLICYETRHF--KDNMHVFIDLVKRE

go7 :----ICTVGSNPIEFLKILEKTGFLPLEADHPYSYKNAGNE-CP-KPKENWVNLWGNSKLLFHKM-YGHFMSYKGFISYESSHF--SDNMHLFIDLVKRE

ma8 :----RCTVGSNPIEFLQIVDERKFLPLESDYPYSIINAGNA-CP-KPKNNWTNLWGNSKLLFHKM-YGHFMSYKGFISYESNHF--RDNMDLFIDLVKRE

ov5 :----RCAIGSNPIEFLQILDEQKFLPLESELPYFYKNAVNE-CP-KPKDKWTNLWGNTKLLFDKM-YGHFMSYKGFISYETLQF--RDNMNLFVDIVKRE

yo3 :----VCNDGSNPLEFLKILEKNKFLPLESNYPYLWKNVSGT-CP-NPQNDWTNLWGNTKLLYNNM-YGQFIKHRGYIVYNSRFF--AKNMDVFIDIVKRE

be3 :----ICNDGSNPLEFLKILEKNKFLPLESNYPYLWKNVSGK-CP-NPQNDWTNLWGNTKLLYNNM-FGQFIKHRGYIVYSSRFF--AKNMNVFIDIIKRE

ch3 :----VCNEGSNPLEFLKILEKNRFLPLESNYPYLWKNVSDT-CP-RPEEHWTNIWGNTKLLYNNM-YGQFIKHRGYIVYNSRYF--AKNMDVFIDIVKRE

fa6 :----RCEEGSNPLEFLRILDEKKFLPLESNYPYSYTSAGNS-CP-KLPNSWTNLWGDTKLLFNKK-VHRYIGNKGFISHETSYF--KNNMDLFIDMVKRE

re6 :----RCEEGSNPLEFLRILDEKKFLPLESNYPYSYTNAGNS-CP-KLQNSWTNLWGDTKLLFNKK-VQRYVGNKGFISHETSYF--KNNMDLFIDMVKRE

vi11 :----RCNVGSNPTEFLQIVKDTGFLPLESDLPYSYSDAGNS-CP-NKRNKWTNLWGDTKLLYHK-RPNQFAQTLGYVSYESSRF--EHSIDLFIDILKRE

cy10 :----RCNVGSNPTEFLQIVKDTGFLPLESDLPYNYSQAGNS-CP-NKRSKWTNLWGNTKLLYHK-RPNHLFQTFGYVSYESKRF--ENNIDLFIDILKRE

fi8 :----RCNVGSNPTEFLQIVNDTGFLPLESDLPYSYKEAGNS-CP-NKRNKWTNLWGNTKLLYHK-RPNKIVQTFGYVSYESRRF--QDNIDLFIDILKRE

so8 :----RCNVGSNPTEFLQIVKDTGFLPLESDLPYSYKDAGNT-CP-NKRNKWTNLWGNTKLLYHK-RPNKILQTFGYVSYESKRF--QDNIDLFIDILKRE

in6 :----ICTVGSNPTEFLQIVEETGFLPLESDLPYRWSDAGNS-CPYNRRSMWTNLWGNTRLLYHG-RSHQLVQTHGYVAYESRRF--EYNIDLFIDILKRE

hy6 :----ICTVGSNPTEFLQIVKETGFLPLESDLPYRWSDAGNS-CPYNRRSSWTNLWGNTRLLDHR-RPRQLVQTHGYVAYESRHF--EYNIDLFIDIVKRE

co6 :----RCSVGSNPTEFLEIVKDTGFLPLESDLPYSYKHAGKS-CPKNR-NKWTNLWGDTKLLYHV-RSNVFYKTLGYVSYESKLF--HTNMDLFIDILKKE

kn5 :----RCSVGSNPTEFLQIVKDTGFLPLESDLPYKYTDAKNS-CPMNR-NKWTNLWGNTKLLYHV-RPNLFLKTLGYVSYESKFF--RKNIDLFIDMLKRE

fr4 :----ICTVGSNPIEFLQIVNDTGFLPLESDLPYSYKDAANS-CPSNR-NKWTNLWGHTKLLYHK-RPREFMQSLGYVAYESGNF--DGKMDLFVDILKKE

go8 :----RCTVGSNPTEFLQIVQDTKFLPLESDLPYNINNAGNS-CP-MKRNKWTNLWGNTRLLYHK-MYHNYLNTLGYIAYDSDTF--KDNMDLFIDILKKE

ma9 :----ICIVGSNPTEFLQIVNDNKFLPLESDLPYSYNDAGNA-CP-TKRNKWTNLWGNTKLLLHK-RDYSFLNTLGYIAYESINF--KDKMELFINIIKQE

ov6 :----ICNVGSNPIEFLQIVHETKFLPLESDYPYSYSNASNS-CP-KKRNKWTNLWGNTKLLYHK-KHHYFMNTLGYVSYESLHF--KHNLPLYIELLKRE

yo4 :----ICDIGSNPIEFIQILHDVKYLPLESDYPYSYYNVGDS-CP-TPKSNWTNLWGNNKLLYFKSGSVEFMSSYGFIAITSSNH--LDDFDTYVQIIKNE

be4 :----ICDIGSNPIEFLQILHDAKYLPLESNYPYSYYRVGGS-CP-RPKSDWTNLWGNNKLLYFKSRSIGFMSSYGFIAITSSNH--IYDFDTYIQIIKNE

ch4 :----ICDVGSNPVEFLQILHDKKHLPLESDYPYSYYRVGGS-CP-SPKNSWTNLWGNNKLLYYKSRSVDFTSSYGFIALSSSNY--QDDLDTYIQIIKNE

fa7 :----ICFVGSNPVEFLEIVEETGFLPLESDVPYYYTDAGND-CP-EPEKNWINLWGSTELLNHK-RPRQRMTTKGYISYESSYF--SDNMDLFIKIIKRE

re7 :----ICFVGSNPVEFLEIVEETGFLPLESDVPYYYTDAGND-CP-EPEKNWINLWGSTELLNHK-RPSQRMSTKGYISYESSYF--SDNMDLFIKIIKRE

vi12 :YNDNICREGGHLSYYLDTVDKTGMLPTSYDVPYNEPLRGAD-CP-ESTAKWANIWEKVNPLTRIL-NG-YL-YRGYFKISFHEYARSGKTQELISLIKDY

cy11 :YNDNICSEGGHLSYYLETLDKTKMLPTSYDVPYNEPLKGAE-CP-EANAQWDNMWDQVNPLTRIM-NG-YL-YRGYFKISFHEYARSGRTDELISLIKDY

fi9 :YNDNICNEGGHLSYYLDTLEKTKMLPTSYDVPYNEPLKGAE-CP-ESNEKWANMWENVNSLTRIL-NG-YL-YRGYFKISFHEYAKDGKTQELISLIKDY

so9 :YNDNICNEGGHLSYYLDTLEKTKMLPTSYDVPYNEPLKGAE-CP-ESNENWANMWENVNSLTRIL-NG-YL-YRGYFKISFHEYARDGKTQELISLIKDY

in7 :YNDNICKEGGHLSFYLETLEKTRMLPTSYDVPYNEPLKGAE-CP-QSSVDWANMWDQVNPLTKIM-NG-YI-YRGYFKISFHDYSRSGKMEELIRLIKDY

hy7 :YNDNICKEGGHLSYYLDTLDKTKILPTSYDVPYNEPIKGAD-CP-QSNDKWANIWDQVNPLTKIL-NG-YL-YRGYFKISFHEYSRSGKVDQLISLIKDY

co8 :YNDNICREGGHLSYYLDTLDKTKMLPTSYDVPYNEPIKGVD-CP-ETSDKWTNMWENVNPLTRIL-NG-YL-YRGYFKISFHEYANSGKTDELISVIKDY

kn6 :YNDNICREGGHLSYYLETLDRTKMLPTSYDVPYNEPIKGVD-CP-ESSDKWANMWEHVNPLTRIL-NG-YL-YRGYIKISFHEYAQSGKTDELISLIKDY

fr5 :YNDNICREGGHLSYYLDTLDKTGMLPTSYDVPYNEPIKGAD-CP-QTSDKWANMWEKVNPLTRIL-NG-YL-YRGYFKISFHEYARNGKTQELISLIKDY

go9 :YNDNICREGGHLSYYLETVDKSGILPTSYDVPYNEPLKGAD-CP-ENNKTWSNMWEKVNLIDRIL-NG-YL-YRGYFKISFHEYTRNGKTEELIRLLKDY

ma10 :YNDNICREGGHLSYYLETLDNTKMLPTSHNVPYNEPIKGAD-CP-DSKPSWSNMWDEVNLVDRIF-NG-YI-FSGYFKLSFGEYVRNGMTKDLIKIIKEY

ov7 :YNDNICREGGHLSSYLETLSQSGVLPTSYDVPYNEPIRGAN-CP-DSSSTWSNIWDKVNLIEKIL-NG-YL-YRGYVKISFSDYAMSGRTQELIRLLKDY

yo5 :YNDNVCHEGGHLSSYIEILDASKMLPTSFDVPYNDPIKGGE-CP-AEASTWGNIWDGVSSLSKIL-NG-YI-YKGYFKISFLDYVQAGKTHELINILKDY

be5 :YNDNICHEGGHISSYIEILDASKMLPTSFDVPYNEPIKGGE-CP-TEVSTWGNIWNGVSSLSKIL-NG-YI-YKGYFKISFLDYVQAGKTNELINILKDY

ch5 :YNDNICHEGGHLSSYIEILDLSKMLPTSFDVPYNEPIKGGD-CP-TEVSTWGNLWNGVTALDKIL-NG-YI-YKGYFKISFLDYTQAGKTNELINILKDY

fa8 :YNDNICKEGGHLSYYLETLEKTRMLPTSHDVPYNEPITGSE-CP-DNKETWSNIWKGVNLMDRIY-AG-YI-YHGYFKVSFKDYVVSNRTNDLINIIKDY

re8 :YNDNICKEGGHLSYYLETLEKTRMLPTSHDVPYNEPIIGSD-CP-DNKETWSNIWEGVNLMDRIY-AG-YI-YHGYFKVSFKDYVVSNRTNDLINIIKDY

ga2 :YNDNICKEGGHIVFFLNTVEKTGMLPTSHDVPYYAPLKSSE-CP-KSKSSWSNIWDQVNLFDKIY-NG-YM-YHGFLKISFGDYVEQGKTTELINIIKDY

ga3 :YTTSACKEGGHLVQFLKVLEHTGTLPTSSNIPYFPPLNTPQ-CP-YVDPSWPNIWERVTLLNTGN-YG-YT-YQGFWKISFREYAQRNRTQDLINILKDL

392 :------1210------1220------1230------1240------1250------1260------1270------1280------1290------1300

392 :###############################-----################################################################

570 :#################################--#################################################################

vi1 :VRSKGSVMAYIKVLDILSYDFNGKEVHSLCGDK--RPDHAVNIIGYGNYVTPEGVKKSYWLMRNSWGKYWGDGGNFKVDTHGPPGCQHNFIHTAAVFNLS

cy1 :VRGKGSVMAYVKALGVLGYNMNGKEVHSLCGDK--RPDHAVNIIGYGNYINTQGLKKSYWLVRNSWGKYWGDEGNFKVDMHGPADCQHNFIHTAAVFNLH

fi1 :VRSKGSVMAYVKALGVLGYDMNGKEVHSLCGDK--RPDHAVNIIGYGNYINAEGLNKSYWLMRNSWGKYWGDEGNFKVDMHGPAHCQHNFVHTAAVFNIH

so1 :VRSKGSVMAYVKALGVLGYDMNGKEVHSLCGDK--RPDHAVNIIGYGNYINAEGLKKSYWLVRNSWGKYWGDEGNFKVDMHGPAHCQHNFVHTAAVFNVH

in1 :VRSKGSVMAYVKALGVLGYDMNGKEVHSLCGDK--TPDHAVNIIGYGNYINAEGLKKSYWFMRNSWGKYWGDEGNFKVDMHGPAHCQHNFVHTAAVFNVD

hy1 :VRSKGSVMAYVKALGVLGYDMNGKEVHSLCGDK--SPDHAVNIIGYGNYINAEGLKKSYWLLRNSWGKHWGEDGNFKVDMHGPDGCQHNFIHTAAVFNLD

co1 :VRSKGSVMAYIKALGVLGYDFNGKEVQSLCGDK--RPDHAVNIIGYGNYITAGGHKKSYWLIRNSWGKYWGDGGNFRVDVYGPPECQHNFIHTAAVFNVD

kn1 :VKNRGSIMAYVKAMGVLGYDFNGKEVQSLCGDR--RPDHAVNIIGYGNYINVNGEKRSYWLIRNSWGYYWGNEGNFRVDVYAPPGCQHNFIHTAAAFNVD

vi2 :VMKKGSVIAYVKADDQMSYDLNGKKVLSLCGSE--EPNLAVNIVGYGNYISAEGVKKPYWLLRNSWGKHWGDDGTFKVDMHGPPGCQHNFIHTAAVFNLH

cy2 :VMNKGSVIAYVKAAGALSYDLNGKKVLSLCGGE--TPDLAVNIVGYGNYINGEGVKKSYWLLRNSWGKHWGEDGNFKVDMDSPPGCQHNFIHTAAVFNVD

fi2 :VMNKGSAIAYVKAAGALSYDLNGKNVLSLCGGE--GPDLAVNIVGYGNYINGEGVKKAYWLLRNSWGNYWGDKGNFKVDMDGPLGCQHNFIHTVAVFNVD

so2 :VMSKGSVIAYVKADELMGYDLNGKKVLSFCGGE--TPDLAVNIVGYGNYINGEGVKKSYWLLQNSWGKHWGENGNFKVDMDSPPGCQHNFIHTAAVFNVD

in2 :VMNKGSVIAYVKAAGALSYDLNGKKVLSLCGDE--TPDIAVNIIGYGNYINGEGVKKSYWLLRNSWGKHWGDEGNFKVDMHGPPGCQHNFIHTAAIFNVD

hy2 :VMNKGSVIAYVKAAGALSYDLNGKKVHSLCGGE--NPDLAVNIIGYGNYINGEGVKKSYWLLRNSWGKYWGEDGNFKVDMHGPAGCKHNFIHTAAVFNVD

co2 :IMNKGSVIAYVKAAGALSYDLNGKKVQSLCGSEE-TPDLAVNIIGYGNYINGEGIKKSYWLLQNSWGKYWGDGGHFKVDMHGPPECQHNFIHTAAVFNVD

go1 :VMKKGSVIAYVKTNQVMDYDINGKTVHGICGGE--VPDLSVNIIGYGNYINAEGVKKSYWLLRNSWGKYWGDNGNFKVDMHTPAHCQHNFIHTAAVFNLD

go2 :VMKKGSVIAYVKVDEMMDYDINGKAVHNICGSE--VPDLAVNIIGYGNYINAEGVKKSYWIIKNSWGKYWGDEGNFKVDMDTPDQCQHNFIHTAAVFNLD

vi3 :VMKKGSAIAYVKAQGALSYDLNGKKVLSLCGGE--TPDLAVNIVGYGNYISAEGVKKPYWLLQNSWGKHWGDKGTFKVDMHGPPGCQHNFIHTAAVFNLD

vi4 :VMKKGSAIAYVKAAGVMTHDMNGVDVHSLCGGE--TPDLAVNIVGYGNYISPEGVKKSYWLMRNSWGKYWGDDGTFKVDMHGPPGCQHNFIHTAAVFNLD

vi5 :VMKKGSAIAYVKAQGALSYDLNGKKVQSLCGGE--TPDLAVNIVGYGNYITAEGQKKSYWLLQNSWGKHWGDDGNFKVDMHGPDHCQNNFIHTAAVFNLD

cy3 :VMNKGSVIAYVKAQGVMSYDLNGKKVLSLCGGE--TPDLAVNIVGYGNYINGEGVKKSYWLLQNSWGKHWGDKGYFKVDMHGPDHCQKNFIHTAAVFNVD

cy4 :VMNKGSAIAYVKAQGVMKYDLNGLDVHSLCGGE--KPDLAVNIVGYGNYITEEGQKKSYWLLRNSWGKYWGDDGNFKVDMHGPPGCQQNFIHTAAVFNLD

cy5 :VMNKGSAIAYVKAQGVMSYDLNGKKVLSLCGGE--TPDLAVNIVGYGNYINGEGVKKSYWLLQNSWGKHWGDKGKFKVDMHGPAHCQQNFIHTAAVFNVD

fi3 :VMNKGSAIAYVKAAGALSYDFNGKKVQSLCGGE--TPDLAVNIVGYGNYINGEGVKKSYWLLQNSWGNHWGDNGNFKVDMDSPPGCQHNFIHTVAVFNLD

so3 :VMNKGSVIAYVKAAGALSYDFNGKEVQSLCGGE--TPDLAVNIVGYGNYINGEGVKKSYWLLQNSWGKHWGENGNFKVDMYSPPGCQHNFIHTAAVFNVD

in3 :VMNKGSVIAYVKAAGALSYDLNGKKVLSLCGDE--TPDIAVNIIGYGNYINGEGVKKSYWLLRNSWGKHWGDEGNFKVDMHGPPGCQHNFIHTAAVFNVH

hy3 :VMNKGSVIAYVKAAGALSYDLNGKKVLSLCGGE--TPDLAVNIIGYGNYINEEGVKKSYWLVRNSWGKYWGEDGNFKVDMDGPAGCKHNFIHTAAVFNVD

co3 :IMNKGSVIAYVKAAGALSYDLNGKKVLSLCGSEE-TPDLAVNIIGYGNYINAEGIKKSYWLLQNSWGKYWGDEGNFKVDMHGPPECQHNFIHTAAVFNLH

kn2 :IINKGSVIAYVKAAGALSFDLNGKKVQNLCGEEG-TPDLAVNIIGYGNYINEEGIKKSYWLLQNSWGYYWGNEGTFKVDMHGSDHCEHNFIHTAAVFNLD

go3 :VMKKGSVIAYVKAKDVLNYNFNGKVVHSICGSE--KPDLVVNIIGYGNYINSEGQKKSYWLVRSSWGKYWGDKGNFKVVMDTPDQCQHNFIHTAAVFNLD

vi6 :VMSKGSVIAYVKADELMGYDFNGKNVHSLCGSE--TPNHAVNIIGYGNYVSAEGVKKSYWLLRNSWGKYWGDDGNFKIDMHGADHCQHNFIHTAAVFNLE

cy6 :VMSKGSVIAYVKADELMGYDFNGKKVHSLCSSE--TPNHAVNIIGYGNYITEEGQKKSYWLLRNSWGKYWGDDGNFKVDMHGPAHCQQNFIHTAAVFNLD

fi4 :VMSKGSVIAYVKADELMGYDFNGKKVHILCSSE--TPNHAVNIIGYGNYMNAEGQKKSYWLLRNSWGKYWGDDGNFKVDMDSPPGCQHNFIHTAAVFNLD

so4 :VMSKGSVIAYVKADELMGYDFNGKKVHILCSSE--TPNHAVNIIGYGNYITAEGQKKSYWLLRNSWGKYWGDDGNFKVDMDSPPGCQHNFIHTAAVFNLD

fr1 :VMNKGSVIAYVKAAGALSYDLNGKKVLSLCGGK--TPDLAVNIIGYGNYINEEGMKKSYWLLRNSWGSHWGDDGNFKVDMHGPHGCQQNFIHTAAVFNLD

go4 :VMKKGSVIGYVKSDEVMDYFLNGKKVLNLCGDK--TADHALNIIGYGNYINGEGVKKSYWLLRNSWGKYWGDNGNFKVDMHTPVHCQHNFIHTAAVFNLD

vi7 :LMKKGSAIAYVKAAGALSYDLNGKKVLSLCGSE--TPDLAVNIVGYGNYISAEGQKKSYWLLQNSWGKHWGDKGTFKVDMLGPDHCHHNFIHTAAVFNLD

vi8 :VMSKGSVIAYVKADDQMSYDLNGKKILSLCGSE--TPDLAVNIVGYGNYITAEGQKKSYWLLQNSWGKHWGDKGTFKVDMHGSAHCQHNFIHTAVVFNLD

cy7 :VMNKGSVIAYVKADELMGYDLNGKKVLSLCGGE--TPDLAVNIIGYGNYIHGEGVKKSYWLLQNSWGKHWGDKGKFKVDMDGLPGCQHNFIHTAAVFNLD

fi5 :VMNKGSVIAYVKADEFMGYDLNGKKVLSLCGGE--TPDLAVNIVGYGNYINGEGVKKSYWLLKNSWGNHWGDKGNFKVDMHGPPGCQHNFIHTAAVFNLD

so5 :VMNKGSVIAYVKADELMGYDLNGKKVLSLCGGE--TPNLAVNIVGYGNYINGEGVKKAYWLLRNSWGKHWGDEGNFKVDMDSPPGCQHNFIHTAAVFNLD

in4 :VMNKGSVIAYVKAAGALSYDLNGKKVLSLCGDE--TPDIAVNIIGYGNYINGEGVKKSYWLLRNSWGKHWGDEGNFKVDMHGPPGCQHNFIHTAAVFNLD

hy4 :VMNKGSVIAYVKASGALSYDLNGKKVLSLCGGE--APDLAVNIIGYGNYINGEGVKKSYWLVRNSWGKHWGDNGNFKVDMHGPDGCQHNFIHTAAVFNLD

co4 :IMNKGSVIAYVKATGALSYDLNGKKVQSLCGSEE-TPDLAVNIIGYGNYINGEGVKKSYWLLQNSWGKYWGDGGHFKVDMHGPEHCQHNFIHTAAVFNLD

kn3 :IMNKGSVIAYVKATGALSFDLNGKKVQNLCGEEG-TPDLAVNIIGYGNYINEEGIKKSYWLLQNSWGYYWGNEGTFKVDMHGPDDCEHNFIHTAAVFNLD

fr2 :VMNKGSVIAYVKAAGALSYDLNGKKVLSLCGGQ--TPDLAVNIIGYGNYINEEGVKKSYWLLRNSWGKYWADGGNFKVDMHGPHGCQQNFIHTAAVFNLD

go5 :VMKKGSVIAYVKVDEMMGYDMNGKAVHGICGGE--VPDLAVNIIGYGNYINAEGVKKSYWLLRNSWGKYWGDNGNFKVDMHTPEHCQYNFIHTAAVFNLD

vi9 :VMKKGSVIAYVKVAGALSYDLNGKKVLSLCGSE--TPDLAVNIVGYGNYISAEGVKKPYWLLQNSWGKHWGDKGTFKVDMNGPPGCHHNFIHTAAVFNLD

cy8 :VMNKGSVIAYVKAQGVMTYDLNGKKVLSLCGGE--TPDLAVNIVGYGNYINGEGVKKSYWLLQNSWGKHWGDKGKFKVDMDGPPGCQHNFIHTAAVFNVD

fi6 :VMSKGSAIAYVKAAGALTYDFNGKKVQSLCGGE--TPDLAVNIVGYGNYINGEGVKKSYWLLQNSWGNHWGDKGNFKVDMHGPPGCQHNFIHTAAVFNLD

so6 :VMSKGSVIAYVKADELMGYDLNGKKVLSLCGGE--TPNLAVNIVGYGNYINWEGVKKAYWLLRNSWGKHWGDKGNFKVDMDGPPGCQHNFIHTAAVFNMD

go6 :IMKKGSVIAYVKTNQMMDYDINGKTVHGICGGE--VPDLAVNIIGYGNYINAEGMKKSYWLVRNSWGKSWGDKGNFKVDMHTPADCQHNFIHTAAVFNLD

ma1 :VMHKGSVIAYVKAENAMDYDLNGKKVLSLCGDK--NADHAINIIGWGNYTNDEGKKKSYWIIRNSWGKYWGDEGNFRVDMHGPEQCEHNFIHSAVIFNIH

ma2 :VMHKGSVIAYVKVENVMGYDLNGKKVLSLCGGK--HPDHAVNITGYGNYINSKGEKKSYWIVRNSWGKYWGDEGNFMVDMHGPEQCEHNFMHSVVMFNID

ma3 :VMHKGSMIAYVKAEKIMSYDFNGKKVYSLCGEK--MPDHAVNIIGWGNYISDKGMKKSYWIMRNSWGKYWGDEGNFKVDIYGPEKCEHNFIHSAVMFNID

ma4 :IMHKGSVIAYVKAKSVMGYNLNGKKVLNVCGDK--NPDHAVNIIGWGNYINIEGNEKPYWIIKNSWGKYWGDDGNFKVDMYGSEQCEHNFIHSVAIFNID

ma5 :VMHKGSVIAYVKVENVMGYDLNGKKVLSVCGDK--NADHAVNIIGWGNYINDEGKKKSYWIVRNSWGKYWGDDGNFKVDIHGPEECEHNFIHTVAMFNID

ma6 :VMHKGSVIAYVKVENVMGYDLNGKKVLSVCGDK--NADHAVNIIGWGNYINDEGKKKSYWIVRNSWGMYWGEEGNFKVDIYGPEKCEHNFIHSVVKFNID

ma7 :ILLKGSVIAYVKTKNAMGYDLNGKKVLSLCGDK--HPDHAVNITGYGNYINSKGEKKSYWIVRNSWGKYWGDEGNFMVDMHGPEQCEHNFMHSVVMFNID

ov1 :IKNKGSVIAYVRAENALSYDTNGGKIHKLCGSE--NPDHAVNIVGYGNYISADGNKKSYWIVRNSWGENWGDNGNFKVDMDTPAECKHNFVHTAAVFNLD

ov2 :VKIKGSVIAYVNAENTLGYNLNGKEVQNLCGSE--TPDHAVNIIGYGNYINNDGEKKSYWIVRNSWGENWGDNGNFKVDMDTPAECKHNFVHTAAVFNLD

ov3 :IKNKGSVIAYVRAENALSYDTNGGKIHKLCGSE--NPDHAVNIVGYGNYISADGKKKSYWIVRNSWGENWGDNGNFKVDMDTPAECKHNFVHTAAVFNLD

ov4 :IKNKGSVIAYVNAEDVMGFDFNGKKVHNLCGDG--VPNHAVNIVGYSNYINANGEKKSYWIVRNSWGKHWGDEGNFKVDMYTPAECKNNFVHTAAVFNLD

yo1 :IQNKGSIIAYINSKDIISYDFNGSKIHKLCGSD--TPDMAVNIIGYGKYINENNQVKSYWLVRNSWGKHWADEGNFKVDIETPENCEHNFIHTFATFNIY

be1 :IQNKGSVMAYVNSKYITSYDFNGSKVHKLCGSV--TPDMIVNIIGYGKYINENNEVKSYWIVRNSWGKHWADEGNFKVDIETPENCKHNFIHTFATFNIY

ch1 :IQNKGSVIAYVNSKDINSYDFNGSKVHKLCGSA--TPDLAVNIIGYGNYANENNEVKSYWLVRNSWGKHWADEGNFKVDIETPENCEHNFIHTFATFNIY

yo2 :IKKLGSVIAYVKFSDFMGYDFNGNKVHKLSGSG--TPDMIVNIIGYGKYINENNQVKPYWLVRNSWGKHWADEGNFKVDMETPENSEHNFIHTVLVFDLD

be2 :IKKLGSVIAYAKFNDFMGYDFNGNKVHKLCGSG--TPDMIVNIIGYGKYINENNEVKSYWIVRNSWGKHWADEGNFKVDIETPENCEHNFIHTALVFDFD

ch2 :IKKLGSVIAYVKFNDSMGYDFNGSKVHKLCGSA--TPDMAVNIIGYGKYINENNEVKSYWLVRNSWGKHWADEGNFKVDIETPEDCEDNFIHTALVFDLD

fa1 :IMNNGSVIAYVKAENVLGYELNGKNVQNLCGDK--TPDHAVNIVGYGNYINDEDEKKSYWIVRNSWGKYWGDEGYFKVDMYGPSTCEDNFIHTVVVFNIN

re1 :IMNKGSVIAYVKAENVLGYELNGKKVQNLCGDK--KPDHVVNIVGYGNYINDEDEKKSYWIVRNSWGKYWGDDGYFNVDMYGPSTCEDNFIHTVVVFNIN

fa2 :IMNKGSVIAYVKAKNVLGYELNGKKVQNLCGDK--KPDHAVNIVGYGNYINNKGEKKSYWIVRNSWGKYWGDDGYFKVDMYGPPTCEDNFIHSVVVFNVE

re2 :IMNKGSVIAYVKAENVLGYELNGKKVQNLCGDK--KPDHAVNIVGYGNYINNEDEKKSYWIVRNSWGKYWGDDGYFKVDMYGPSTCEDNFIHSVVIFNVE

fa3 :IMNKGSVIAYVKADKIMAYEFNGKKVQNLCGDK--TPDHAVNIIGYGNYINDEHQKKSYWIVRNSWGKHWGDKGHFKVDMYGPSDCEDNFIHSVVIFNVD

fa4 :IMNKGSVIAYVKAENVLGYELNGKKVQNLCGDK--TPDHVVNIVGYGNYINNKGEKKSYWIVRNSWGKYWGDDGYFKVDMYGPSTCEDNFIHTVVVFNVQ

re4 :IVNKGSVIAYVKAENLLGYELNGKKVQNLCGDK--KPDHVVNIVGYGNYINDEDEKKSYWIVRNSWGKYWGDDGYFKVDMYGPSTCEDNFIHSVVIFNVE

fa5 :VMNKGSVIAYIKAENVMGYEFSGKKVQNLCGDD--TADHAVNIVGYGNYVNSEGEKKSYWIVRNSWGPYWGDEGYFKVDMYGPTHCHFNFIHSVVIFNVD

re5 :VMNKGSVIAYIKAENVMGYEFSGKKVQNLCGDD--TADHAVNIVGYGNYVNSEGEKKSYWIVRNSWGPYWGDEGYFKVDMYGPSHCHFNFIHSVVIFSVD

fa9 :IKNKGSVIAYINAQSVLTYDFNGKNILNMCGSK--TTDHSINVIGYGHYKNKEGEEKSYWLVRNSWGYYWGDEGNFKVDMYGPSNCNYHFINTVLVFNID

re9 :IKNKGSVIAYINAQSVLTYDFNGKNVLNMCGSK--TTDHSINVIGYGYYKNKEGEEKSYWLVRNSWGYYWGDEGNFKVDMNGPSNCNYHFINTVLVFNID

ga1 :IQNKGSVIVYIKAENVFGYDLNGKGVHSLCGYK--HPDHAATIIGYGKYINIKGEKRSYWLVRNSWGYYWGDEGNFKIDMHGPSTCKYNFIHTALVFKVN

vi10 :VQNKGSVIIYIKTKDVIGYDFNGRGVHNMCGDK--TPDHAANIVGYGNYINTKGVKRSYWIVRNSWGYYWGNEGTFKVDMLGPKGCKYNFIHTAVVFKVN

cy9 :VQNKGSVIIYIKTKGVIGYDFNGTGVQNMCGHK--IPDHAANIVGYGNYINRKGEKRSYWIIRNSWGYYWGNEGTFKVDMLGPKECKYNFIHSAVVFKVN

fi7 :VQNKGSVIIYIKTKDVIGYDFNGRGVHNMCGDK--IPDHGANIVGYGNYINTEGVKRSYWIIRNSWGYYWGNEGTFRVDMHGPKGCKYNFIHTAVVFKVN

so7 :VQNKGSVIIYIKTKDVIGYDFNGRGVHNMCGDK--IPDHGANIVGYGNYINTEGVKRSYWIIRNSWGYYWGNEGTFRVDMHGPKGCKYNFIHTAVVFKVN

in5 :VQNKGSVIIYIKTKDVIGYDFNGRGVHSICGDK--IPDHAANIVGYGNYINTKGVKRSYWIIRNSWGYYWGNEGTFKVDMLGPEGCTYNFIHTAIVFKLN

hy5 :VQNKGSVIIYIKTKDVLGYDFNGRGVQNMCGDR--MPDHAANIVGYGNYINTNGVKRSYWIIRNSWGYYWGNEGTFKVDMLGPKECKYNFIHTAVVFKVN

co5 :VQNKGSVIIYIKTKDVIGYDFNGKGVHNMCGDK--VPDHGANIVGYGNYINVDGEKRSYWLIRNSWGYYWGNEGTFKVDMLGPKGCKYNFIHTAVVFKVN

kn4 :VQNKGSVIIYIKTKDVIGYDFNGNGVQSLCGDK--TPDHGANIVGYGNYINVNGEKRSYWLIRNSWGYYWGNEGTFKVDMHGPPGCKYNFIHTAVVFKVN

fr3 :VQNKGSAIIYIKTKDEIGYDFNGKVVHNMCGHR--VPDHAANIIGYGNYISVTGEKRSYWLIRNSWGYYWGNEGTFKVDMLGPKGCKYNFIHTAIVFKVN

go7 :IQNKGSVIIYIKTKDVIGYDFNGRGVHTICGDK--MPDHAANIVGYGNYINTEGEKKSYWIVRNSWGYYWGNEGTFKVDMHGPKNCKYNFIHTAVVFKVN

ma8 :IQNKGSVIIYIKTKDVIGYDFNGRGVHSMCGDR--VPDHAANIIGYGNYINTDGKKRSYWIVRNSWGYYWGTEGNFKVDILGPPHCKYNFIHTVVVFKLN

ov5 :VQNKGSVIIYIKTKDVIGYDFNGRIIHNMCGDS--LPDHGANIVGYGNYINTAGEKRSYWLIRNSWGYYWGNEGTFKVDMLGPKHCKYNFIHTAVVFKMN

yo3 :IRNKGSVIAYIKTQGVIDYDFNGRYISNICGHS--HPDHAVNIIGYGNYISDNGEKRSYWLIRNSWGYYWGHEGNFKVDVLGPDNCVHNVIHTAIVFKID

be3 :IRNKGSVIAYIKTQGVIDYDFNGRYISNICGHN--HPDHAVNIIGYGNYISESGEKRSYWLIRNSWGYYWGHEGNFKVDVLGPDNCVHNVIHTAIVFKID

ch3 :IRNKGSVIAYIKTKDVIDYDFNGRYISNICGDS--HPDHAVNIIGYGNYISDKGEKRTYWLIRNSWGYYWGHEGNFKVDVLGPDDCVHNFIHTAIVFKID

fa6 :VQNKGSVIIYIKTQDVIGYDFNGKGVHSMCGDR--TPDHAANIIGYGNYINKKGEKRSYWLIRNSWSYYWGDEGNFRVDMLGPKNCLYNFIHTVVFFKLD

re6 :VQNKGSVIIYIKTQDVIGYDFNGKGVHSMCGDR--SPDHAANIIGYGNYINKKGEKRSYWLIRNSWSYYWGDEGNFRVDMLGPKNCLYNFIHTVVFFKLD

vi11 :IQNKGSVIIYIKTNNVIDYDFNGRVVHSLCGHK--DADHAANLIGYGNYISAGGEKRSYWIVRNSWGYYWGDEGNFKVDMYGPEGCKRNFIHTAVVFKID

cy10 :VQNKGSVIIYIKTENVIDYDFNGKVVHSLCGHN--DADHAANIIGYGNYINVHGEKRSYWLVRNSWGYYWGDEGNFKVDMYGPDGCKRNFIHTAVVFKID

fi8 :IQNKGSVIIYIKTNDVIDYDFNGRIVHSMCGHN--DADHAANIIGYGNYISVGGEKRSYWLVRNSWGYYWGDEGNFKVDMYGPEGCKWNFIHTAVVFKID

so8 :IQNKGSVIIYIKTNDVIDYDFNGRTVHSMCGHN--DADHAANIIGYGNYISVNGEKRSYWLVRNSWGYYWGDEGNFKVDMYGPEGCKWNFIHTAVVFKID

in6 :IQNKGSAIIYIKTENVIDYDLNGRFVHSICGHN--DADHAANIIGYGNYIGLGGKKSSYWIVRNSWGYYWGDEGNFKVDMYGPEGCKWNFIHTAVVFKID

hy6 :IQNKGSVIVYIKTENVIDYDLNGAFVHNLCGHN--DADHAANIIGYGNYLGLGGKKRSYWIVRNSWGYHWGDEGNFKVDMYGPKGCKRNFIHTAVVFKID

co6 :IQNKGSVIIYIKTENTIDYDFNGRIVHSLCGHN--DADHAANIIGYGNYINVDGEKRSYWLIRNSWGYYWGDEGNFKVDMHGPAHCQHNFIHTAVVFKID

kn5 :IQNKGSVIIYIKTEDNIDYDFNGKIVHSLCGHK--DADHAANIVGYGNYINVNGEKRSYWLIRNSWGYYWGDEGNFKVDMYGPPGCKYNFIHTAVVFKID

fr4 :IQNKGSVIIYIKTEDTIDYDFNGKTVHLICGKL--DADHAANLIGYGDYITPYGEKRSYWLIRNSWGYYWGDEGNFKVDMYSHQYCRRNFIHTAVVFKID

go8 :IQNKGSVIIYIKTENVIDYDFNGRIIHNLCGDN--DADHAANIIGYGNYISSNGEKKSYWLVRNSWGYYWGDEGNFKVDMYGPKKCEHNFIHTAVVFKID

ma9 :IQNKGSVIVYVKTNNVIDYDFNGKVVHSICGDN--EADHAANIIGYGNYINIKGEKRSYWLVRNSWGYYWGDEGNFKVDMYGHDECLYNFIHTAVVFKID

ov6 :IQSKGSIIVYIRTNNVIDYDFNGRIVHNLCGDD--EADHAANVVGYGNYINNQGEKRSYWIIRNSWGYYWGDEGNFKVDMYGPEECKYNFIHTAVVFKMD

yo4 :IRNKGSVIAYIKTNNVIDYDFNGKIIHNLCGDDNDDADHAATIIGYGNYISNTGEKRSYWLIRNSWGYYWGDEGNFKVDIYGPQYCKYNFIQTVVFFKLD

be4 :VRNKGSVIAYIKTNGVIDYDFNGKIIHSLCGDDNDYADHAATIIGYGNYISESGEKRSYWLIRNSWGYYWGDEGNFKVDIYGPQHCKYNFIQTVVFFKLD

ch4 :VRNKGSVIIYIKTNNVIDYDFNGKIIHNLCGDDDDDADHAAAIIGYGNYISEKGQKRSYWLIRNSWGYYWGDEGNFKVDMYGPRHCKYNFIHTVVIFKLD

fa7 :IQNKGSVIAYIKTENVIDFDFNGKGVHNMCGDK--EPDHAANIIGYGNYIDEEGEKKSYWLIRNSWGYYWGDEGNFRVDMYGPSYCKYNFIHTVVVFKVD

re7 :IQNKGSVIAYIKTENVIDFDFNGKGVHNMCGDK--EPDHAANIIGYGNYIDEDGEKKSYWLIRNSWGYYWGDEGNFRVDMYGPSYCKYNFIHTVVVFKVD

vi12 :IMDQGSLFVSMEVNEKLSFDHDGEKVMMNCEYK-ASPDHALTLIGYGDYIKPSGEKSSYWLIRNSWGSHWGDKGNFKVDMYGPPNCNGAVLCNAFPLLLQ

cy11 :IIDQGSLFVSMEVNEKLSFDHDGEKVMMNCEYR-ASPDHALALIGYGDYLKPSGERSSYWLLRNSWGSHWGDRGNFKIDMYGPSDCNGAVLCNAFPLLLQ

fi9 :IMEQGSLFVSMEVNEKLSFDHDGEKVMMNCEYN-ASPDHALTLIGYGDYLKPSGEKSSYWLIRNSWGSHWGDRGNFKVDMYGPGDCNGAVLCNAFPLVLQ

so9 :IMEQGSLFVSMQVNEKLSFDHDGEKVMMNCEYN-ASPDHALTLIGYGDYLKPSGEKSSYWLIRNSWGSHWGDKGNFKVDMYGPGDCNGAVLCNAFPLVLQ

in7 :IIEQGALFVSMQVNEKLNFDHDGEKVMMNCEYN-ESPDHALALIGYGDYLKPSGEKSSYWLIRNSWGSHWGDKGNFKVDMYGPANCNGAVLCNAFPLMLR

hy7 :IIEQGALFVSMEVNQKLNFDHDGEKVMMNCEYN-EAPDHALALIGYGDYLKPSGEKSSYWLIRNSWGSHWGDKGNFKMDMYGPSNCNGAVLCNAFPLMLQ

co8 :IIEQGSLFVSMEVNEKLSFDHDGEKVMMNCEYK-ASPDHALALIGYGDYIKPSGEKSSYWLLRNSWGSHWGDRGNFKIDMYGPPDCNGAVLCNAFPLVLQ

kn6 :IIEQGSLFVSMEVNEKLSFDHDGEKVMMNCEYR-ASPDHALTLIGYGDYLKPNGEKSSYWLIRNSWGSHWGDKGNFKIDMYGPSDCNGAVLCNAFPLFLQ

fr5 :IMDQGSLFVSMEVNEKLSFDHDGEKVMMNCEYK-AAPDHALTLIGYGDYLKPSGEKSSYWLIRNSWGSHWGDKGNFKVDMYGPADCNGAVLCNAFPLVLQ

go9 :IIEQGSLFVSMQVNEKLSFDHDGEKVMMNCEYN-ESPDHALVLIGYGEYMKPSGEKSSYWLIRNSWGSHWGDKGNFKVDMYGPGNCNGAVLCNAFPLLLQ

ma10 :IIEQGALFVSMKVNGKLSFDHDGEKVMMNCEYE-ESPDHALVLIGYGDYIKPSGEESSYWLIRNSWGSHWGDKGNFKIDMYGPSNCNGQVLCNAFPLLLQ

ov7 :IMEQGSIFVSMEINEKLTFDHDGEKVMLNCEYG-QAPDHALVLIGYGDYITSSGSKSSYWLVRNSWGSHWGDRGNFKIDMYGPSNCNGNVLFNAFPLLLE

yo5 :IIEQGAIFVSMEISQKLNFDHDGEQVMLNCEYG-EAPDHALVLIGFGDYIKSSGEKSSYWLIRNSWGSRWGDNGNFKMDMYGPGNCNGRVLFNAFPLLLK

be5 :IIEQGAIFVSMEISQKLNFDHDGEQVMLNCEYG-EVPDHALVLIGFGDYIKSSGEKSSYWLIRNSWGSRWGSNGNFKMDMYGPRNCNGRVLFNAFPLLLK

ch5 :IIEQGAIFVSMEISKKLSFDHDGEQVMMNCEYG-EAPDHALVLIGFGDYIKSSGEKSSYWLIRNSWGSRWGDNGNFKMDMYGPRNCNGRVLFNAFPLLLK

fa8 :IIQQGSVFVSMEVTDKLTFDHDGTKVMMSCEDN-DSPDHALVLIGYGDYIKTNGKKSSYWLLRNSWGSHWGDKGNFKLDMYGPNNCNGKVLYNAFPLLLN

re8 :IIQQGSVFVSMEVTDKLTFDHDGTKVMMSCENN-DDPDHALVLIGYGDYIKTNGKKSSYWLLRNSWGSHWGDKGNFKLDMYGPNNCNGAVLYNAFPLLLN

ga2 :IIDQGALFVSMTITEKLSFDHDGEKVMISCQHN-ETPDHALALIGFGDYIMPSGKKSSYWLIRNSWGAHWGDKGNFKLDVYGSPNCNGHVLYNAFPLLLE

ga3 :VMKQGGIFVTMQISGKLSYDHDGSRPMMNCEYG--RPEHAITLIGYGNYTGYGGRQSSYWLIRNSWGQKWGDNGNFKIDMYGPSNCNGAVLADAYPLNLK

392 :------1310------1320------1330------1340------1350------1360------1370------1380------1390------1400

392 :#---------------------------------------------------------------------------------------------------

570 :##---------------------------###############--------------------------------------------------------

vi1 :MPM---------AEGPS---KGEA-HLYDYYLKSSPDFLGNIYHKISGRRSGAVEKGRTTGNQSLT----------------------------------

cy1 :VPM---------SEGPS---KREA-HLYNYYLKSSPDFLGNIYYKNIRRMSGVAEKGRTGRHQSLA----------------------------------

fi1 :VPV---------AESPS---KREA-HLYNYYLKSSPDFLGNIYYKNIGRKSGAAEKNRTGRNQCLA----------------------------------

so1 :VPL---------AESPS---KREA-HLYNYYLKSSPDFLGNIYYKNISRKSGTAEKGRTGRNQSLA----------------------------------

in1 :VPV---------AENPS---NREA-HLYKYYLKSSPDFLGNIYYKNIGRRSAATGKDQTDRNQGLP----------------------------------

hy1 :VPV---------AENPS---KSEA-DLYKYYLKNSPDFLGNIYYKNIGSKSGAAGKDQTGRNESLP----------------------------------

co1 :VLV---------AENPS---QREA-PLYNYYLKSSPDFWGNIYYKNIGRKDGAELKDRTGGNQNLT----------------------------------

kn1 :VPV---------VKSTS---QREP-PL*NYYLKSSPDSFGSIYYNNIGPKDDSALKNRTGANQNLA----------------------------------

vi2 :IPP---------MENVE---KKKP-LLYNYYMKSSPDFYNHIFYRGVQTGEESEMGISGQEKVSISA---------------------------------

cy2 :LPL---------LENSE---KKRP-MLYNYYMKSSPDFYNHILYRGVQT-EEREIGISGEDKTSSFV---------------------------------

fi2 :IPL---------LENTE---KKGP-MLHNYYMKSSPDFYNHILYRGVQTEEKRKIGISGKDKLISSV---------------------------------

so2 :FPL---------LENIA---KKRP-MLYNYYMKSSPDFYNHILYRGVQTEDKRKIGICGEDKLISSV---------------------------------

in2 :VPL---------LENLE---KKRP-MLYNYYMKSSPDFYNHILYKGVQTEEDREMGISQEDKMISPV---------------------------------

hy2 :VPL---------LENLE---KKRP-MLYNYYMKSSPDFYNHILYRGVKSEEDGEIGISPEEKMISPV---------------------------------

co2 :LPL---------LENVE---KKGP-MLYNYYMKSSPDFYNHILYRGVQTEEDREIGISGQDKIISSV---------------------------------

go1 :LLP---------FETVS---KKNS-ELHNYYMKNSPDFYNNLYYMNLDAKEGSENGNNEKAKAHSSM---------------------------------

go2 :IPQ---------FESGS---KKNS-ELNNYYLNSSPDFYSNLYHNALDTEKVGGSISKKAHSKDTT----------------------------------

vi3 :IPV---------VVPAP---NSDP-EINNYYLKNSPDFFSNFYLNKYEAGSDGNSDDAKSVSGNST----------------------------------

vi4 :MPL---------ATHSI---NKDP-EISDYYLKNSSDYYENLYYKKFQGSGATGGAGKQWVQGAST----------------------------------

vi5 :VPV---------VAPAP---SSDP-EINDYYMKNSPDFFSNFYFNKYEAEKANGLGEEKGPVNNSV----------------------------------

cy3 :IPV---------VIPAP---NSDP-EINNYYLKKSPDFFSNFYFNKLEAESEGNSGGAKSASNNLT----------------------------------

cy4 :IPL---------ATHPI---NKDP-EINDYYLKNSPDYYDNLYYKKFQENGATGGVSKECVQGAST----------------------------------

cy5 :IPV---------IVPAP---NSDP-EINNYYLKKSPDFFSNFYFNKLEAESDGNSGGAKNASNNLT----------------------------------

fi3 :IPV---------VVPTP---NSDP-EINNYYLKNSPDFFSNFYFNKLEADKANGLGDQKGPLHDSV----------------------------------

so3 :IPV---------VVPAP---NSDP-EINNYYLKNSPHFFSNLYLNKLEAEKANGLGDGKGHVHNSV----------------------------------

in3 :IPV---------VVPAP---NSDP-EINNYYLKNSPDFFTNFYLNNREAEKANGLGNEKGPIHNSV----------------------------------

hy3 :IPV---------VVPAP---NTDP-EINNYYLKHSPDFLNNFYLNKREAEKVNGLGDGKGPIHNSV----------------------------------

co3 :IPV---------VIPTP---NSDP-EINSYYLKNSPDFFSNFYLNKLEAEKANGLSHQKGPIHNSV----------------------------------

kn2 :IPV---------VIPAP---NGDP-EINSYYLKNSPDFYKNLYYNALDGECGNTPCHT------------------------------------------

go3 :IPQ---------FEITA---NKEP-EIQQYYMKNSPDFYNNLYFKNFDALKANNSSNGIGSHNNFV----------------------------------

vi6 :MPL---------VQSAA---KKDN-HLYSYYLKGSPDLYQNMYYKGFGSQGGNAKGGEKGQVGTPV----------------------------------

cy6 :IPL---------AQSAA---KKDN-QLYSYYLKGSPDLYQNLYYKGFGSQGGNAKEGEKGKVGSPV----------------------------------

fi4 :VPL---------AQSAA---KKDN-QLYSYYLKGSPDLYQNLYYKGFGSQGGNAKEGEKGQVGSPL----------------------------------

so4 :VPL---------SQSAT---KKDN-QLYSYYLKDSPDLYQNLYYKGFYSQGGNVKAGEKGQVGNPV----------------------------------

fr1 :VPI---------AQSVT---KKNN-QLYSYYLKGAPDLYENLYYKGFGSQCKNTNEGQKGQVGNLV----------------------------------

go4 :IPP---------FETGS---KKNS-ELNNYYLNSSPDFYSNLYYNNFGAKSRGTKISEKDLVGNSL----------------------------------

vi7 :MPQ---------ANPPP---V-EF-QLSNYYLKSSPDFYTNLYYKNVKATPG----------G-AK----------------------------------

vi8 :VPL---------SQSPA---K-DT-ELYSYHLKSSPDFYKNLYYNAVGGEKG-------SVLS-NA----------------------------------

cy7 :VPP---------FQSPA---K-DT-ELYSYQLKSSPDFYKNLYYNAVGEAKG-------SAPS-QA----------------------------------

fi5 :MPQ---------ASLSI---V-ES-QLSNYYLKSSPDFYTNLYYKNFNAPPG-------AVAGGAK----------------------------------

so5 :VSP---------FHSPA---K-DT-ELYSYHLNSSPDFYKNLYYNALGEKSG-------SALS-HA----------------------------------

in4 :VPP---------FQSPA---K-EA-KLYNYYLKSSPDFYKNLYYNALGEQRG-------RAPS-HA----------------------------------

hy4 :VSP---------FHSPA---K-EA-KLYNYSLKSSPDFYKNLYYNALGEQKG-------KAPS-HA----------------------------------

co4 :VPH---------FQSPA---K-DT-QLYSYHLKSSPDFYKNLYYNALGGECG-------SAPC-QA----------------------------------

kn3 :VPD---------FQSPM---K-DT-ELHSYYLKNSPDFYKNLYYNALDGECG-------SAPC-HT----------------------------------

fr2 :MPI---------AQSVT---KKNN-QLYSYHLKSSPDLYKNLYYSALGGS-G-------SSPN-HA----------------------------------

go5 :IPH---------FESAS---KKNT-EMYSYYLSSSPDFYDNLYYNAVDGVKGNATKNGKHLPGGYT----------------------------------

vi9 :MPV---------EENPQ---KEDA-QIYNYYLKSSPDFWGNIYYKNVGGQESASSKNATEGAHESV----------------------------------

cy8 :IPV---------VEKPT---KEDA-QIYNYYLKSSPDFWGNIYYKNVGGQTNTSAKNATGVANESV----------------------------------

fi6 :IPV---------VEKPT---KEDA-QIYNYYLKSSPDFWGNIYYKNVGGQKSASAKNATGVSHESV----------------------------------

so6 :IPV---------VEKPT---KEDA-QIYNYYLKSSPDFYGNIYYKNVGGQKSASAKNATGGVHESV----------------------------------

go6 :MPQ---------MDVNT---KQEP-EIYNYYLKNSPDFYNNLYYKNFDALKASASNKQNGFHNNSV----------------------------------

ma1 :MPI---------AQVPT---KKGA-EIYNYYLKTSPDFYSNLYYNNFTPEEESVPLKRNDLSQNSL----------------------------------

ma2 :MPL---------TNVNK---KKEA-ELYNYYLKTSPDFYSNLYFNSLSAEKANDLNNKNVITANSV----------------------------------

ma3 :MPL---------AKVNT---KKEA-ELYNYYLKISPDFYSNLYYKNFSSDKTYYSNSRKGLYWNSM----------------------------------

ma4 :MPM---------DDVTA---EKDA-GLYNYFLKTSPDFYNNLYNKNFNQETYNASYNEKALAGNSE----------------------------------

ma5 :MPV---------AQVVK---NKDA-EIFNYYGKTSPDFFSNLYYKSLESVKNSNSSGGTFLGQNSM----------------------------------

ma6 :IPL---------SKVNT---KKEA-ELYNYYLKTSPDFYSNLYFNSLSAEKANDLSTNKVLDQMT-----------------------------------

ma7 :MPL---------TNVNK---KKEA-ELYNYYLKTSPDFYSNLYFNSLSAEKANDLNNKNVITANSV----------------------------------

ov1 :LPI---------VEKSV---KGEA-ELYNYYLKSSPDLYSNLYYSIYSKEGNEQPNYSKVAAENSA----------------------------------

ov2 :LPT---------VKNSA---NKEP-ELYNYYLKNSPDFYSNLYYQNFNPEDNLSPVENTT----------------------------------------

ov3 :LPI---------VEKPV---KAEA-ELYNYYLKSSPDFYSNLYYKNYSKAKNGVVNNGSVSAQSDS----------------------------------

ov4 :FPI---------VEKPV---KAEA-ELYNYYLKSSPDFYSNLYYKNYSKAKNGVANNGSVSAQSDT----------------------------------

yo1 :IPF---------VKKNI---PSES-EINLYYSKNSPDFYNNLYFKNLDATDTNKDSI-------------------------------------------

be1 :IPF---------VKKSI---QSES-EINLYYSKISPDFYNNLYFKNLDAADTSEDSI-------------------------------------------

ch1 :VPF---------VKKNT---QTES-EINLYYSKNSPDFYNNLYLKNLDADNTNEDSL-------------------------------------------

yo2 :LPV---------AQKI----NNDA-PIYNYYLKNSPNFYKNLYYNHVSRKNAKAQADADAA---------------------------------------

be2 :LPP---------AQKN----NNDA-SIYNYYLKNSPNFYKNLYYNHVSKTNAETA---------------------------------------------

ch2 :LPV---------VAKN----TNEA-PIYNYYLKTSPSFYKNLYYNHVNKTHGEVSDDAA-----------------------------------------

fa1 :MPK---------SKKSPV--KITF-PLYNYYLKYSPDFYHNLYYKNFNSKKSMKLVNASDEHKN------------------------------------

re1 :MPK---------SKKSPV--KITF-PLYNYYLKYSPDFYHNLYYKNFNSKKSMKLVNASDERKN------------------------------------

fa2 :VPV---------NENFD---KKEH-DIYKSYLKNSPDFYHNIYYKNYNFENYVSPISTWSEVLHNTLYNNNL----------------------------

re2 :VPV---------NENFD---KKEH-DIYNYYLKNSPDFYHNIYYKNYNLENHVSPISTWSKGLHNKLYNDNL----------------------------

fa3 :LPI---------NQESV---KKEP-KIYNYYLKASPDFYHNLYYKNFDSQKGKADQAENKKSY-------------------------------------

fa4 :VPI---------NEKFD---KKEH-DIYNYYLKTSPEFYHNLYYKTFNSNKEEKSMNKNSY---------------------------------------

re4 :VPV---------NENFH---KKEH-DIYNYYLKTSPEFYHNLYYKTFNSNKEEKTLNKNSY---------------------------------------

fa5 :LPM---------NNKTT---KKES-KIYDYYLKASPEFYHNLYFKNFNVGKKNLFSEKEDNENNKKLGNNYI----------------------------

re5 :LPM---------NNKTT---KKES-KIYDYYLKASPEFYHNLYFKNFNVGKNNLFSEKQDNENNKKLGNNYI----------------------------

fa9 :IPM---------MNNNE---NNKK-GLYNYFPKYTHEFYHNFYDQNFGSDNKISLINSNGSNKNDVNNAYT-----------------------------

re9 :IPM---------MNNNE---NNKK-GLYNYFPKYTHEFYHNFYDHNFGSDNKISLINSNGSNKNDANNAYT-----------------------------

ga1 :LGM---------VELPDE--KSAPNPF-NYFLKNGPEFYHRLYYKNFSPLKSKNLSKKNENNEEDQTNFTNNEDNGNDKTDLGENPQIYGTDNDV-----

vi10 :LGA---------IDIPNK---DVD-LNRTYFPKHNSDFFHSLYFNNYDEEADGEEK-PVFQGARSSEEGGSGSGSGSGGSGGGSGT--------------

cy9 :LGA---------IDIPNK---DVD-LNRTYFPKHNSDFFHSLYFNNYDDEADGE-KK-IVFQKDRSNDDGGSGSGSGSGSGKGKGKG-------------

fi7 :LGA---------IDIPNK---DVDDLNRTYFPKHNSDFFHSLYFNNYDDEADD--KK-NVFQKYRRNDDRGSRSGSD-----------------------

so7 :LGA---------IDIPNK---DVDDLNRTYFPKHNSDFFHSLYFNNYDDEADN--KKSVFQKYRRNFDSGSGS---------------------------

in5 :LGP---------IDIPNK---DVD-LNRTYFPKHSSDFFHSLYFNNYDDEADDEEKN-VFQKDRSNDHGGSGNDSGSEKANGKGSGGKGTDSSSGSGS--

hy5 :LGP---------IDIPNK---DVD-LHHTYFPRHSSDFFHSLYFNNYDDEADDEENN-VPQKGRSNKRGGSGKGSAS-----------------------

co5 :LGA---------IDIPNK---DVD-LNHTYFPKHNSDFFHSLYFNNYDDEAANQ-KK-IIPKYRNKDYSGSA----------------------------

kn4 :LGA---------VDIPNK---DVD-LNHTYFPKHNADFFHSLYFNNYDDEADNQ-KK-IFPKYRNMDYSGSG----------------------------

fr3 :LGA---------IDLPNK---DVN-INRTYFPKHSSDFFHSLYFNNYDDEADKP-RK-IFHKHRNKDNKRHVHGW-------------------------

go7 :LGS---------IDIPNQ---DID-LHSSYFSRHNSGFFHSLYFNNYDDEADGEEK-WAFQKSDISDHNGDINNDNSNGNNSYSNSDSNSDSNSDSNSDS

ma8 :LGI---------VNISNN--KIND-KA-PYFSKHSSDFFHSLYFNNYNEYEDESPFDNDDDMNVVNPGGNKYKGFSQKEPRKGI----------------

ov5 :LGA---------VNVSNK--KDVT--N-AYFPKHNADFFHSLYFNNYNDMHEALPFDNYYSEGSGTGKSKYSRKKRSYGSKWGTTWEHKKGS--------

yo3 :MEPDNA------SKNDDQ---LVDESDKSYFPQLSSNFYHSLYYNNFEGYEAKNDDENDQNYDKVD----------------------------------

be3 :MEPDSDSNNNNAIKNRDQ---LIDEDNKSYFPQLSSNFYHSLYYNNYEGYEAKNDDENDQNYGNLD----------------------------------

ch3 :MEPDNHGG----IKNKDQ---SIDENKKSYFPQLSSNFYHSLYYNNYEGHEAKSDDENDRDYDNAD----------------------------------

fa6 :LGT---------IHVPKK----KSWKKNVYFLRHNPDFMYSLYYNNYEPETSQDFESENDYDNAF-----------------------------------

re6 :LGT---------IHVPKK----KSWKKNVYFLRHNPDFMYSLYYNNYEPERSQDYLSENDYDNTF-----------------------------------

vi11 :LGI---------VEVPKK----DEGSIYSYFVQYVPNFLHSLFYVSYGKGAD--------KGAAV------------VTGQA---GGAV-----------

cy10 :LGI---------VEVPKK----DDRPFYNYFIEYVPNFLYNLFYSSYDKGDDSNTLCENAKGGEA------------VGGDSVVDGQAV-----------

fi8 :LGI---------VEVPKK----DDRPFYSYFIQYVPNFLHNLFYVSYDKGADANALFEKGKGGTAG------------GGDAIVDGQAV-----------

so8 :LGI---------VEVPKK----DDRPFYSYFAQYAPNFLHNLFYVSYDKGADANALFEKGKGGAAG-----------VG-DAIVDGQAV-----------

in6 :LGI---------VEIPKK----EDRSFYRYFVQYVPNFLHSLFYVSYDKGVNGKKHSENRKGGIAGGGGLVDGKAGQAGGGSLVDRQAV-----------

hy6 :LGI---------VEIPKK----EHRPFYRYFVQYVPNFLHSLFYVSYDKGTNRKRHGENGKRGAS-------------AGYALFDGQAV-----------

co6 :LGI---------VEVPKKKK--RDQSLYNYFVKYVPNFLYNLFFVCFDARAYIDDDDNVLGEKGK------------EGASGG--ENAV-----------

kn5 :LGI---------VEVPKK----GEKPPHNYFAKYVPNFLYNLFFVCFD-GGAADDDDIELGQNGK------------VGAAGG-KENAV-----------

fr4 :LGM---------VEVPKK----EDPPFYSYFAKYLPNFLYNLFYDNYDKGAEVYALCESGNAG--------------EA-TGG---NAV-----------

go8 :LGI---------VEVPQK----DEVPMYNYFSKYVPNFLHSLFYANYDKDSDAPSFFQGCKGE-----------------------EAI-----------

ma9 :LDM---------VEVPKKNNNNNNNSVYNYFHKYVPDFFYNLFYVNYDKDKDKDKGESFLYDNGNREEIQQNYSKDSI----------------------

ov6 :LGI---------VEVPKK-----NNNVINDSLKYVPNFFYSLFYYNYDKKLGDYTTLGWDSKDGKDGKDGKDGKDGKDSKDDKDGEKKGNT---------

yo4 :LGV---------IEGPQN-----NNQVYNYFSRHIPAFFTNLFYSSYDKRWDELSSDEELNNYNKNIS--------------------------------

be4 :LGV---------IEVPKK----KNNQVYKYFSNNIPAFFTNIFYSNYNKRWDQFRSSEELNNYNNNIS--------------------------------

ch4 :LGE---------IEAPQK-----DNQVYNYFAKHIPAFFTNLFYSNYNKRWDEFYAKEELNNYNKDIS--------------------------------

fa7 :LGI---------IEVPKKE---KESEYFSYFLKYTPNFLYNLFFNNYTTNDEYKLNNRLKTNQHNNKKNKKDRY--------------------------

re7 :LGI---------IEVPKKE---KESKYFSYFLKYTPNFLYNLFFNNYTTNDEYKLNNQLKTNQHNNKKNKKERY--------------------------

vi12 :MNGKK-------ITKPLP----KDLASTDNKVRYDHSIFNRDNNRAQSRRYNQNREQDRPNGRA---DNN-PFDNPKYNE-IYDDDDRD----APAFDPF

cy11 :MNGNK-------ITKPLP----KDLASTDSRIRYDHSMFTRDQNRRQSRRYNSGEEQDRPNGQA---DNN-PFDNSKNND-TYDGDDRN----WQDFDPF

fi9 :MNGKK-------ITKPLP----QDLASTDNKVSYNHSYFTRDKNRRNSRRYNSPNEQERPNGWE---DNN-PFDNPKNND-IYDDDDRD----WPNFDPF

so9 :MNGKK-------ITKPLP----QDLASTDNKVSYDHSYFTREQNRRHSRRYNSGKEQDRPNGWE---DNN-PFDNPKNND-MYDGEDRE----WPNFDPF

in7 :MNGEK-------ITKPLP----DDLASTDNRTRYNHSDFTRDKNRRSSRRYKSDKERDQPNGWA---DNN-PFDNPKNNE-IDDGDKGD----WPPSNPF

hy7 :MNGEI-------ITKPLP----NDLASTDNRVRYNHSDFTRDRNGRQSRRYNSDREADQPNGRE---DKYNPFGDPKNNE-IDDGDNSD----WPHSNPF

co8 :MNGKK-------INKPLP----DDFASTDNKTRYDHSYFTRDRNRRQSRRYNSNKEQDQPNDQA---HYD-PFDNPKNNE-IYDGEDRD----WPAFDPF

kn6 :MNGDK-------ITKPLP----DDLASTDNKTRYDHSYFTRDRNRRASRRYNPNNERDQPNNQE---DKD-PFANPNNND-NYDGNDRD----WPPFDPF

fr5 :MNGKK-------INKPLP----NDLASTDNKVRYDHSSFTRDQNRRQSRRYNSNKAQDRPSDRA---DYY-PFDKPKNNNDIFDGDDRED---WPAFDRF

go9 :MRGNK-------INKPLP----NDMASTDNKTRYDHSYFNRDRNRNNSRRYNYSGNNQVNPNGRRGGDNNNPFDIPNPND-TFDDQNRY----EPNFDPY

ma10 :MRGKK-------IDNPLP----NDLASTDTRMRYNHSSFDMSRRRKYPEQRDNQINDDRMNPNNPYDNDRYDKNRIDNDRNDNNPFVNPRYEDMYDEHNE

ov7 :MHGER-------IDKPLP----KDMASTDNSIRYNDFNGNSDRRRNKQQQYDNTDNDKVKPNNRYDYMDDDRVNPNNRYDYMDDDRINPNNRYDYIPNGY

yo5 :MTENK-------ISKPLP----NDMASTDIKMRYNHSEFSKNKKN------KNRKITPNNYDDPFNVNPKIVDPPNNNHND---------HNSIDNNDEE

be5 :MKENN-------ISKPLP----NDISSTDVKIRYKHSDFTKNKKN---QRDQNRKITPNNYDDPYNFKPKTVDPPNNNNNDYV----NPDYNSIDANDRQ

ch5 :MTENN-------ISKPLP----NDMSSTDVKIRYNHSDFTKNKKNKTSQRDRNRKVTPNSYDDPYDINPRTVDPSNNNQNDYVNPYYNPDYNPIDNNDRQ

fa8 :MAHNP-------IDVPLP----NDLASTDIRVRYRQSDFNQNRNRNNYPQYDKNSNDNDRNYINPYNKNDNNYNPYNKPHYNDKENDAYYEKNDDYNNAH

re8 :MSHNP-------IDVPLP----NDLASTDNRVRYRQSDFNQNRNRNNYPQYDKNSNDYDRNNINPYNKNDNNYNPYNKARYNDKGNDAYYDNHDDFNNAH

ga2 :MNGKK-------INAPLP----NDTASTDVKTRYDFHGFGSHKENNFSPHNMNIDRNRLYPNPFGDSYPFRKPNHNTYYDPINPYAIPRDQNYNPNKDSS

ga3 :MEGSR-------IDVQLP----NDYASKDSHKKNDPRPNPNPYPRPGPYPGPTPSPNPNPYPGPTPSPNPNPYPRPNPYPGPTPSPYPNPYPRPNPYPGP

392 :------1410------1420------1430------1440------1450------1460------1470------1480------1490------1500

392 :----------------------------------------------------------------------------------------------------

570 :----------------------------------------------------------------------------------------------------

vi1 :----------------------------VQGQEGWSDLLEGTTPSTTANPNPVGELKGAQDVPKRGNTGKRISEDPQEEVLNEELIPQLPLAPISQAAAT

cy1 :----------------------------VQGQEGQSDLLEGSTPSGTANQVDEPKGAREDAPKR-GDQGKCMSEDPPIEVVNEELISESTLTLIPQAA--

fi1 :----------------------------VQGQEGQSELLEGIIPSATANRVEELKGARKDAPKR-GVMGKRISEDPPIKVVNEELISESPLTPIKQAA--

so1 :----------------------------VQGQEGQSELLESSNPSATANRVEEPKGARENAPKR-GNAGKRISEDPQIEVVNEELISESPLAPIPQEA--

in1 :----------------------------VQGQDEQSEILEDSNPSKTANPVEEPKGEREDASKG-GDAGKRSSEDPPIEVVNEELVSESPLTPIPQAA--

hy1 :----------------------------VKGQDEESEILEDSTPSETANPVEEPKREREHVPKE-GDAGKRSSEDPSMEVVNEELISESSLTPITKAA--

co1 :----------------------------VQGQEEQTKLLEDSDLTTTANPVKVPKGAQEDAPKR-GDAGKRISEGPTIGEISEELISESPVTPIQQAT--

kn1 :----------------------------VQGKGKHSELHKDSDPSVITNPVREPKGSQEVAPEM-GYTGKSIFENAEIEEIKGEFISKSPLTSFGEIA--

vi2 :----------------------------VSANTSAADTLDGVDHSSVVVEGKEKNPAGEGESAQGVESPPEAEEQDEEEGDAESEEEGEDEPEEEGDGEQ

cy2 :----------------------------VSGQNSGGDTLDGAEQSSVVVEGKEKPAEGGDESTQEVESPPKAGTDESKEADEQDEEVEEEEPEEEGDGEQ

fi2 :----------------------------VSGQTSTADTLDGAKQFSVVVEGKEDPSTGGGESTQKADWALEAGTDESKEAEEEEEEEEEEEEGDSEAAKK

so2 :----------------------------VSGQTSKADTLDGVEQSSVVVEGKEKPATGGGELTQESESECEAGTDESKEAEEEEEEEEEEWEDEGVAEPE

in2 :----------------------------VSGQKSKADTLNGAEKSSVVVEGKENPAVGVGDSTQEVESPPEAVTDKSKEAEKQDQKEADEESEEERKDKA

hy2 :----------------------------VPEQKSEADTLNGAEKTSVAVEGKENPTAGGGESTEKVESPLEEVTDQSKEAEKQEEEVADEESDEECEDEA

co2 :----------------------------VSGQTSAEDTLDSEEQSSVVGEGKGNTTTEGAESTEEAKSPPEAGTDESIETEEQDEQEEEEGDEFEEKGYG

go1 :----------------------------VSGQEA-EEDVESDETQSLLLDEGEDAAPVPDELATVTESIPEDVPERAKESEELAEKSILEPVTEPDARNP

go2 :----------------------------LYGSSTEDEKVESQKGINGSDDVERSVELSVTLTSEGTQPEAASSSQPSQGDSPTEGVENPEEKDEDDSDGT

vi3 :----------------------------VQGPDGPAGPSGPDGNVGQKGAAARTGEPSTAVERSQTADGVTTPPGGGGANGGRQGAEASVQERNAVDSGS

vi4 :----------------------------VYGQEGDPKVADEAGGEAAQSITTHSRAEPAGDAPPSGPPHAGTDSEQGEGTGPTDGEGPTRVVDEKAQAGG

vi5 :----------------------------LYGQSGEETSALPASVGDQSGKVVAQTAEGLGAQQGSGGGLGAAAGPTGQEGVGVAAPGGRGTGGEAIAAAA

cy3 :----------------------------VQGQEGSTEESTSVQQSQNGGGESGQSAARGADGQGQSEAVAQVQEGGAVVSSQPQADTIPALPTNGVPSAE

cy4 :----------------------------VYGQEDDQKEITEEERKVADSKDTQPTGEVAAGSLSSGSTQTRADSGQEAGTEIGTADGGSTPVVNPNEQTG

cy5 :----------------------------VQGQEEPPVVPTEAQASHSGGSGSGQSAGGSANGQVQPGPGTPVPEGSAVGSSPLQTVPSTSLPTNPDASAA

fi3 :----------------------------LYGQTDDARSGEQESRVDVPGTVVGQAPGVGAVGGATIGGATAGGVGTAGGVGAAGAGEPGAVQPVAETQPG

so3 :----------------------------LYGQSGGETSEQRVSTGDVVGTGVNPASGGGGAGGATATVGRSSQAGAEQPGTHTQTGETSVSGGAGTAGTV

in3 :----------------------------LYGPSVEGTSEPKVAAGGESRQTVSSPSPTGVSVAGANGVGASAASAPGTVPSAENTKPGDASVTGDTNLVK

hy3 :----------------------------LYGQTEEGQEGKLPEGSTGEPGVAGAKVAEGEPGVAGAAGSPAGAGVPGAAGSPGEPGEQEEKSKEEAEEET

co3 :----------------------------LYGQSGEGESEEQVSTSIESGTGVETASDGLVAGGTAATGGPNSPTVPVTSEAVTQPGGEAAPVSTNLVNDA

kn2 :----------------------------VEGQDTSEEEPSEKVTEVGTGVGTVTTTGTEAASGTGAGAGAKAGAEAGAEAGAGAGAGAGAGAGAGAGAGA

go3 :----------------------------IHGQSGEEKPSLGDLAGSALSLITSLAGEVAKTTAEAAVAALKVGVAPGVGVAPGVGVAPAVGVAPGVGVAP

vi6 :----------------------------VSGQEEEEVASDRQDQVREQSHVEASAGPDGLQAGQVDVKAEEEDTKAGQGGEDSAATQDGGSHGPGAVQPL

cy6 :----------------------------VSGQEQGEEEAGGSVQVEVKNQVEVPEVTVDVQADGGSVPPGEVSVQAGQGDVKDGVAQNGGPPGAGAVETL

fi4 :----------------------------VSGQTQEEVSEV--SGKVEGDNQGKEQAERVNEQTEGDIVQTRGGTVQEEGGDVQERGDVQEKGVSEQEEGD

so4 :----------------------------VSGQTQE-VAEVDESGKVEGDNRVEEQTEGDGVQVGQDNANAVAEQNGGSHDAGVVQSLTEESGQREVPPSD

fr1 :----------------------------VSGQTEEVEAHESGQGEGQNQAEVQAGQGGAHDG--------------------------------------

go4 :----------------------------FYGQEDGEKVEPKKEGGDSSELKAGVTPSGEADAAVGQGAQVDAAQKGNDQVSSGNDAAQVAPTQPSDSVAS

vi7 :----------------------------VYGSEGESEVTEGAQNRGAQTAQDRGKVSPGTEQPEKAGGNAAPEAAQITEGGGNRASEASPGIAVPPTPQD

vi8 :----------------------------VQGQDTPQEEGTSPGTAAEGEGRPGPVGPGGQQPQEHASSAGGPNSNEQQLDVARQQQQEGAEKESSQQLPQ

cy7 :----------------------------VHGQDAPEETAVGGGENSVSTVQGQQPQPQVPSGVSTDTKAPEGADNTVSTSEISVGPSQQAQAPQSPGEGQ

fi5 :----------------------------VYGSEAEAEATREDAQNGHNTRQAGEDGVPGAPVTAPGIAGPQTAQNLAASTPAAGAEGESSVASQQAADGT

so5 :----------------------------VHGQDAPQEEGESLGTVVGEGISESTVQEVQQTQLQVPSVMSTNPRVEGQQEQAVVVDQASSHQRAEQVEAS

in4 :----------------------------VQGQDATPVTPAEVGAGDSVSAAQGGQQPQSQVPPAANTGLNGSSAQVPSQQQQVDVAGQPLPAQQAAPGED

hy4 :----------------------------IHGQDEMHVIELGEEDEEEGSPHSTDPKQPPQSPAPPVENTDSIGKEAGVPPPPQEAVEGQPSPAQPTAGGE

co4 :----------------------------VHGQDTPKPVVEVGVEYLESAAEGEQNISQVSPVGSAVSDTQVQVTQQQQQQLQQHPPPPPPEEEHQNGVEH

kn3 :----------------------------VEGQEGTEQDTTVEEGTGQVAAVGGQIAGTPTEGQQTQSQVEGLKTSVSNDQLNGQLEPQVQQEQQQEQQQE

fr2 :----------------------------IHGQEETVDGATSTLTVQGPETAPKGSVVGNTVSSEQAPEAEHTQVEGQPQQTGGSPTPPPSTPQQPTEKVE

go5 :----------------------------VSGQDSTSNGADATTVVQGSETTVQAESTRETGETGNPGSSGVAGPAAPVTVTPAVEEPSQEQVSTPSNVED

vi9 :----------------------------LHGQE---VAEA---AVNGAGGEPAPSHSGAVQGEREGTANGQGPEGVPPLPAKQEVTEASNGEATGLGAVN

cy8 :----------------------------LHGQADTEVEV----KV--------AGEDPATPLSTQATGGATAGQVSTVVPSLPVQVEVTVARNVGAPGDQ

fi6 :----------------------------LYGQEAT-VEGANS-TV----------QGPALAHNAAEIDGRTTEQGSTSVQSSPAQQPVTVARQV------

so6 :----------------------------LYGQGDA-VEGENS-AL----------QEPASLQSTAEIEGTTTGPGSTHVSPVRAPQQVTVVRDLSARVVN

go6 :----------------------------IHGQSDDKSQGFGNLAV----------------DLLSSFTKLAGKAAEVAVDTAINAAKDVMGR--------

ma1 :----------------------------VYGQDEPAPALPTGSASGERASPGVGGVQGDVSSTLPKSTEQNGPQPGNAAAAGQIQSTHQESQPGQRTPVE

ma2 :----------------------------IYGSDNSASGTQPGVGVAPGVGVAPGVGGAQGGLDTKENDKLNEATT-------------------------

ma3 :----------------------------IHGQTDEPSFPSTVERQGIPGGAVMSPGRTQLLVTSVSVGREGTEKSTHEVRPTGGYAPILEGEHPRSESPR

ma4 :----------------------------IYGSEEAAEASQLAGAAVSPVSPAKEEVPRKHPEEVLSTTQPRASTAASGEQTLSPTPVSPPSAPVPPSAPV

ma5 :----------------------------VHGNSGSDTKAQEVSASSSSTTTTTGEEGTNHHQTSPVVGGAAGGTEDTTGKQTLPTEESATVEKQDGATEK

ma6 :----------------------------VHGQAVEESSEGTSGQHGQHGQPGQPESTSSSETVAESSAQGLDSAASDVPDV-------------------

ma7 :----------------------------IYGSDNSASGTQPGVGVAPGVGVAPGVGVAPGVGVAPGVGVSSNTGGPGAEVGTNGSQGSEIEKAVPDSTSP

ov1 :----------------------------VYGQVDGNETDGVGSVAGGESYVEAGNTASYVAGREGGVPGQASLSSSAAATGEEKAGPSGSDVGKKMEETL

ov2 :----------------------------VQGQADAGNSGTDGVTSRGTQVQTQSNTIQTGNGQGTAISHGSGTNTVPTSATS------------------

ov3 :----------------------------VYGQATEQTQASPTSEAAQVLELGGARGVLGGDTSTELNQGESSGGKSLELSKDASGAPSGGALTGETSTGG

ov4 :----------------------------VHGEADNVSAGSVITLSRLTPSSVQPSQESSQASSPGAGPESGPGAGTGSGPEAGTGSGPGPAQEKTPGAEE

yo1 :----------------------------IEGQNEPAKVADSEETNQLTGDGQLKSQQGGETKELSGSDGEKKENEGVESETLPTDSEGETQKLKQEQQQQ

be1 :----------------------------IEGQDEPASISTQEETHQPTGEGNQEESKLPEEKTPKEVGRSEIPSTGTQEKSQELEQQPQPQPQPQPQSQP

ch1 :----------------------------IQGQDEPAKVPTSGDTEQSTKDVDPKSSQKAGTEEKLGSAELKETIQDSGSSSKATLQSTDLSTNPPPKGQE

yo2 :----------------------------ISGQAEEGPTKVEGVSNDKGQTLQSGGGSSQDGTANNVPGSQSGSGITGNGATVKDGATVQAAQGQVANGQT

be2 :----------------------------IFGQDETVQLSNAAGGGSNQVATTINTVTSQLASGATTQINQPSTTTQGQAPQSQTCPSSAVTPSQTPSGSS

ch2 :----------------------------IYGQAEPEAPGKNNQSGSTTDTTKVTTANSESGSDAGGKTKTGESGTSATGKETPAVAGTDGQTNKVTDSSA

fa1 :----------------------------IYSQEDKVNHKKGSKILNSEVTTSLLSQEISQRRGEDDDIDTLIGDSPDINEQEKNIKDEFNKSSITHNSVS

re1 :----------------------------IYSQEDKVNHKKGSKVLNSEVTTSLSSQEISQRRDEDDDIDTLIGHRPDIKEQEKNIKDEFNKSSITHNSVS

fa2 :----------------------------IWGQETTDPIGEGKLPASEAGHLRGEGKGNSEENQGQRSNANGAISSNQSTGGKITKQGESKDDGVALPVST

re2 :----------------------------IWGQEITDPIGEGKLPASEAGHLRGEGKGNSEENQGQGSSANGAISSSQSTDGKNTKQGESKDDGVALSAST

fa3 :----------------------------LYGQEESTSEQLPSSLSSPQNNKQSERSK-------------------------------------------

fa4 :----------------------------VYGQDTTPVENEAPRSGVQKPTELSSTESQTVSPPNESQTESLLSGGSQVTNPTLTQSTSSSSGQQETGPLS

re4 :----------------------------VYGQDTTQVGNKAPVSGEQHPTGSSLSGSQTGSSLSGSQTGSSLSGSETVSPSPVSQTVSSPNGSQTGSSLS

fa5 :----------------------------IFGQDTAGSGQSGKESNTALESAGTSNEVS------------------------------------------

re5 :----------------------------IFGQDTTAPGQTEQGSNTQLASGEAPKEVS------------------------------------------

fa9 :----------------------------VHGQSGETEVQPSQLRSTANQDSNRGAVTSDPLRTQPETTSTSV----------------------------

re9 :----------------------------VHGQSGETEVQHSQLRSTANQDSNRGAVTSDPLRTQPETTSTSV----------------------------

ga1 :----------------------------LTAEAAAVAAVS------------------------------------------------------------

vi10 :-----------KSKKGSKKKQ-------ISGQDAAAATAGATGGASSIARSG-LPPKSPKPSPSAE----------------------------------

cy9 :----------SKSKKGSKRKQ-------ISGQDEGGATGGASSGAASGARSS-LPPQSAKPSPSAE----------------------------------

fi7 :----------SKSKKSWKWKQ-------ISGQDEGGDKGGASSGAASGASST-VRTKSNKSSSSAE----------------------------------

so7 :-----------KSKRSWKWKQ-------ISGQDVGDATGGASTGAASGASST-VPSKSAKPSSSAE----------------------------------

in5 :-----------RWMNGQKKKK-------ISGQDGGAATGGVSSGEASGAHST-VPPQSDKPSSSAE----------------------------------

hy5 :-----------RWR-GQKKKQ-------ISGQDAGDDIEDASSEEASGANSNV-TPPQSAKPSSSAE---------------------------------

co5 :----------YKWKKVAKRWQ-------IAGQDGDDHTGGASSGEASGKNSN-VPPKSAKPTASAE----------------------------------

kn4 :----------YKWKKVSKKWQ-------ISGQDEDITEGASSEETSSNDSS-VAPKTAKPSPSAE-----------------------------------

fr3 :----------YKWKKGPNGWS-------ISGQDGDDAIEGASWGEYYEGELRGIPPQSPKLSSYAE----------------------------------

go7 :NSDSDKRSYQKGKKKTSDSSKKGSKKKQITAQDYDNTSSYAAASSSTSNQSAKANTSAE-----------------------------------------

ma8 :----------------------------IRGQDDPSTGGSDATEGGGSSSSGSNGSNSSSNSNSSSSSSSSSSSSSSSSSSSSSSSSSSSSSSSSSSSSS

ov5 :----------------------------VYGQDEPTDGNQNGAKNYSVE---------------------------------------------------

yo3 :----------------------------VSGQSEGNQTDLKNGQTDPQKTQTDRTVTQPGASPTENPGTAGSNLTTQNTGGSSPTVTDTSSPTTTVTETP

be3 :----------------------------VSGQSENHQNDPKNPQPQANSTVTQLGVCPAENQRTADSNPNAQSTPSPNTTVTDTVNSNTANSNTANSNTA

ch3 :----------------------------VSGESETSDDSSGDSSENGSEESQPQSGGSENPVSSPEAATSIPAPVDSPTPNIATIE--------------

fa6 :----------------------------VHGQSNESDETNKEGKNVHNSVE-------------------------------------------------

re6 :----------------------------VHGQSNESDETNTEGKNVQNSVE-------------------------------------------------

vi11 :----------------------------VTGQTETPTPEAAKNGDQPGAQGSEAEVAEGGQAGNEAPGGLQESAVSSQTSEVTPQ-------SSITAPQI

cy10 :----------------------------VTGQTETPTPGAAQDGAQPGAVGSEVNAAAGDNAGIVPPVGSQTGAVAQESGAVTPETGAVAQESGVVAPQA

fi8 :----------------------------VTGQTETPLPADAENGTQPGAEGSGANTAAGVQAGIVAPEGSQMSPVLPQTGAVAPQTGS------------

so8 :----------------------------VTGQTETSIAGDDKNGTQPGAEGSEANAAAGTETEIVAPEGSQMSPVLPQTGAVTTQ---------------

in6 :----------------------------VTGQTETPTSEAAKNGAQPGAEGSEANAAMGGKAGIVAPVGSQMSPVSPQTSPVSPQTSPVSPQTSP-----

hy6 :----------------------------VTGQTETPTQEADKNGAQHGAEGSEEKA--------------------------------------------

co6 :----------------------------VDGQAAVAGQTENPSSVANKAGIISPVGS-------------------------------------------

kn5 :----------------------------VDGQAVVTGQTENTLSVASKNGAQPETNVSEVNAVSVGQP--------------------------------

fr4 :----------------------------VDGQAAVKGQTETPTSTDAKNGDKPGAKGSEANV--------------------------------------

go8 :----------------------------VSGQTSEETTEGSPTDLTQVGEAISESSTQGQTPNGEPGSDSSTQGQTSDGADISV----------------

ma9 :----------------------------VSGQTEPESAKVIPAPQPTKSAE-------------------------------------------------

ov6 :----------------------------LLGQSEEGEEAEIEAVRKVESVAESGGVAGRVDVPTAKGAR-------------------------------

yo4 :----------------------------ISGQTDNQINTNNDFYNEDTDYINEISTSSSKNISIQ-----------------------------------

be4 :----------------------------ISGQTDNQINANNDFYNEDIDHINEISTSS-KNISIQ-----------------------------------

ch4 :----------------------------ISGQTDNQVNTNDDVYSEDTN---DVSTPSSKNTSIQ-----------------------------------

fa7 :----------------------------ISAQDEPPTDNVESQAENN-----------------------------------------------------

re7 :----------------------------IYAQDEPSTDNVESQVENN-----------------------------------------------------

vi12 :RGDYVYPHMDNANGAERGNE------------GMNDNRG------GKI----------------------------------------------------

cy11 :REGYVYPYMDNRRDGARRNEEDRNNETDRSNEGMGDNRG------SKI----------------------------------------------------

fi9 :K-DYVHPYMDNSNDSERDNGGMSDSRGMSDNRGMSDNRS------SKI----------------------------------------------------

so9 :KEDNVNPYMDNRKDSEH-DNE-----------GISDNRGMSDNRSSKI----------------------------------------------------

in7 :ERDYVDPYKDNDNDAERSDSE-------RGDAGMSDNRG------SMI----------------------------------------------------

hy7 :ERDYLDPYRDNRNDAERRASE--RRASERGDAAISDNRG------SKM----------------------------------------------------

co8 :K-DYVYPYMDNNKDAEGGNG------------GISDNRS------SKI----------------------------------------------------

kn6 :K-DYVYPYMDNRKDAERDNG------------GISDNRS------NKI----------------------------------------------------

fr5 :KGDYVYPYMDNGKYAEGGKP------------GMSDNRS------SKI----------------------------------------------------

go9 :NGNNNYPHVDNKKDMAR-NN----------NAGISDNNR---NR--NI----------------------------------------------------

ma10 :DRYGPNYQPFNDRNAENEYSPEEAGADYPGIGDNRKNST-------------------------------------------------------------

ov7 :DPNRFDPSRFDPSGYDPRRYDPDRYDPSNYDPRNYDPSSYDPRNYDPSNYDPSNYDPSNYNPHNFYPFHFDPFYNPDHGRMHDDHDHRVNPYYSPFDAPR

yo5 :NNERFYPPYIDPEYGPSYPSMNNNRRFDPSDIINHRNR--------------------------------------------------------------

be5 :NNERFYPPSRDPEYDPSYPSMNNNRRFDPSDIINYRNK--------------------------------------------------------------

ch5 :NNDRH-PPFRDSEYDPAYPSMNNNRRFDPNDIINHRNR--------------------------------------------------------------

fa8 :IRRNTIRF--------------------------------------------------------------------------------------------

re8 :IRRNTIRF--------------------------------------------------------------------------------------------

ga2 :YGNNDYAYPRDNSRDRSGIIYNRDIRN-------------------------------------------------------------------------

ga3 :TPNPSPYPRPNPNPSPYPNPNPYPRPNPNPSPYPNPSPYPRPNPNPSPYPNPNPYPRPNPNPSPYPNPNPYPRPNPNPSPYPNPSPYPRPNPNPSPYPNP

392 :------1510------1520------1530------1540------1550------1560------1570------1580------1590------1600

392 :----------------------------------------------------------------------------------------------------

570 :----------------------------------------------------------------------------------------------------

vi1 :LGEGQVEATPLPPLTVAASLPLRGDVLLERDGTNEGSKEA------------------------------------------------------------

cy1 :RREGQVEANPAPPLTVAASLPLRGDVLLVRDGTDEGSTEA------------------------------------------------------------

fi1 :LREGQVQANHAPPLTVAAFLPLWEDGLLVRDGTHEGSTEA------------------------------------------------------------

so1 :LREGQVEANPAPPLTVAASLPLRGNELLVRDGTDEGITKA------------------------------------------------------------

in1 :LREGQVEANPAPPLIVAAPPPLGGDALLVRDGTDEESTEA------------------------------------------------------------

hy1 :LKEGQVEANPAAP------PPLSGDTLLVRGASDEESTEA------------------------------------------------------------

co1 :LREGQVEANPASPLTVTASLPTERDVLLVRDGTDEEKTEA------------------------------------------------------------

kn1 :LGGGQLEANSAPSLTIAASVTPERDVLLV*GDSDEEKTES------------------------------------------------------------

vi2 :EEEGDDESEEVDEEAEEAGAEAEEGEEESEEGGVEAEEGDSEAANNDVDAAEPSQVAAPGASPGGEKPQTVAPPSASNPATPPSAAPAPTR---------

cy2 :EEERDDESEEEEEDEEQEEGVAEQEVGGADAEVGVAEQEVGGAEAEEGGAEAEEGEAEVGEAEVGGAEAEVGRAEPEEGDSEAAKKGVEEPQTVASPSAS

fi2 :GVEGEEASQIAVSDASPKGMKEPQTIASLSVSVNSTPPSAAPAQTR------------------------------------------------------

so2 :EGDSEAAKKGVEAGEPSHVAVSNASLKGMEEPKTVASSSASVNSTPPSAAPAPAR---------------------------------------------

in2 :EEEMHGEKQEEGDDEEWEEEGEDEAEEEAEEQEEAEEQEEPEEQKEPEEQEEAEEEEEEEEAEEEEEAEEAEEEEEAEEEEEAEEEEEGEEEEEAEEEET

hy2 :EEEMHGEEQEEGDDEEWEEEDEEEEEEEEEEEEEEEEEEEEEEEEESENGGLEPEEGAA-----------------------------------------

co2 :EQEEEGDDEFEEEGYDKFEEEGDDEQEVEEDNEPEEEGAEYEEGYTETGKESVETEEPSRVAMSDESSGEMEEPHTMASPSGSVNSTPSSAAPASTR---

go1 :EQSQVVVPHANPRDSSKSVQEPRAATTHTH----------------------------------------------------------------------

go2 :DSDEASEVEL------------------------------------------------------------------------------------------

vi3 :PQAVTRPTPPPERVASGLASVQHSGDTTLPQSTTSSVTQNPPVATRPAPQTTHQTAPAQPATP-------------------------------------

vi4 :PLTSPGTLQDAVAPGGPQGPPEQGSPGSPGPQETPGPQGPPVQQGSPGPQLPPVQPTAPVPPVLPVPPVLPVPPAA------------------------

vi5 :VVVVGGGGSGSAGERGTGVGVAPGVGAAGRSVGEGQQLPAGAASPGTGTQHVGGSVSGGTNSGNGATGNSGPNTQRGQVSQTVNEAAPS-----------

cy3 :ASGQQNGEAGQSVSNTLTETQNSSVTTETTEQTTQQNTQQTAQVPSAQSA--------------------------------------------------

cy4 :GLTAGTGALPQTPAPNVETVLPTPPPPIGPPAAPAAPAPNGPHTPSESPAPTVQNEPTVQTAE-------------------------------------

cy5 :PVQQNGGADQQVSPTLPGTPNSLATTGTTGQAPEQPTVQPTPQTVQVPSAQPA-----------------------------------------------

fi3 :GTSVPVVTNLATGASGNSGLSTGGGQVTSTTVTTTDTPPGTTTVTTTGTTTA------------------------------------------------

so3 :QHVAVTQPGVVSVPEVTNLPNAEPGSGGLSTGNGQITSTPVTTTVTHPT---------------------------------------------------

in3 :GTPKNSRSSTGGENVTPTANQAVSSVTTAV----------------------------------------------------------------------

hy3 :EEEEDEEDEDDEEDDGPKEEATTVTVPTGTGTNPGEQPDAVDTNSVNSNSGNSAPTANQAASSVTTDL--------------------------------

co3 :PGNSATSTEGVQVTPPSNEPVPPVTDTVTGPA--------------------------------------------------------------------

kn2 :EAEAAKATDAQEKSKLQEEQQTQPQPQPAQEGLTNQNAQQADGGVVQGAPGQAVTTQPVQSGEGNGTGGLTVPGNTNLVEGTPQNSGPSAEIGQANTNTN

go3 :GVGVAPGVGVAPGVGVAPGVGVAPGVGVAPGVGVAPGVGVAPGVGVAPGVGVAPGVGVAPGVGVAPGVGVAPGVGNPTPGGTGSVSAVTQEVSPAGGSQS

vi6 :AEQSDTRGVASNLPDGSQLPGSGHEAGQSGHGVAESGSEVPESRPEAPPGEPGAAHSEPQVTESEHGVPPSGSGVPPGGPGVPPSGPEVGRSETGETLSQ

cy6 :TEQPAGTVVPNNSPGG------------------------------------------VQVPPSDPAVSMSGPGAAQSGPEVAQSGPEVTQSETVVTLAP

fi4 :EKAGLGDSNAVAAHNGGSHEAGAEQPPTVQPEQRGVEQNLPDGPEVPPNEPEVPPREPAVSPSEPI--------VTQSETEVGRSETEVGRSETEVTLSP

so4 :PAVSQSRPGEAEGESGVEESGPEVAQREPQVGHGDENAEATQNGVSHGAGGVQPSTEQPPQTE---------------VQNRLPGEPGVAQSETGVTLSP

fr1 :----------------------------------------------------------------------------------------VAQSGTGVTLSP

go4 :SPSEEQGTPPVTTAFGPVVTPTDPSVRLTSPADTPTDP-------------------------------------------------VVAPTQNDAVQTQ

vi7 :PAGGDVSAGTGVESPVSLPQAAGGTPSASLNTQESSSQAQDPQSPAREEQVAAPPQPDVPTPTPQQASTSALVKAPLAQSADA-----------------

vi8 :QQPLTQSQQSTQPGVGGAASTPLSQGTPGQANSNGEAENA------------------------------------------------------------

cy7 :VVSTQQQPGGSTPAPEPSAPQPAQQVDASTPVATVTPGGANPNGGAENA---------------------------------------------------

fi5 :DSTSVTTEESSHQAQALQSPREEEGQGVLPQPQPEVSTPAQPSSSQQPTEQEQASTPVATGTPGAANSNAAVQSA-------------------------

so5 :TLGAANTQESGPKVTEGAQNGDAQSTGIAGVQTAPGTAPAIGAQTAQNTVGGETAAGGQGEGSVVSQLAADGTDSRGITTDISSPQAQTLQPPGGQGVST

in4 :STTPAPNSTEETESSAEEEDQ-------------------------------------------------------------------------------

hy4 :APTTPATDATEGEKSEEEEENP------------------------------------------------------------------------------

co4 :QAGGPEVQTVPGGENPNAGMEIE-----------------------------------------------------------------------------

kn3 :QQQEQQQQQQQEQQQQQQQQQPQPPQQAQPETAQEGLTNENSPHTGGTEVHDVLEEANSNAELQKA----------------------------------

fr2 :NPTAPSSDASGDNSIEEVVNE-------------------------------------------------------------------------------

go5 :S---------------------------------------------------------------------------------------------------

vi9 :PSGEEQPGPPGPPGTSGLAEQPGPEGPSGEVGSTGSVGQPGQPGSSGTPRSEGQTGPSGTSGSSAAQGQSGSSGTPGSDGPSGHTGPSSTPVQEAPLSKA

cy8 :AGSPGPAGPTGPAGSPGSPGQEGQEGPAG---------------------------------PPGTPGPEGPEGSAGPTGPEGPEGSAGPTGPPGPSGTP

fi6 :--GTTGEAVNPSGDQSRSSGDGQPAPEGTTGQAGPQGEAGSPGSAGLPGSAGTPGSVGTPGSVGTPEQPGPSGTTGADGTT----ETPGPAGPSGQPGPD

so6 :PSDRNQSSSSGDGQPGQVGSLASSVEQGTAGPQGPQGPSGPQGPPGPPGPPGTPGPSGTPGPSGTPGPSGPPGEAGTPEQTGLPAETPSQPGPDGTTGAA

go6 :--------------------------------------------GAATTGGGGTSGTESAGTGGEQGQPGVASMSGNSLETSGSQPSGDTAAVAGPEQTP

ma1 :TSVDPKQSVLPGSQTVAAKNAPAAT---------------------------------------------------------------------------

ma2 :----------------------------------------------------------------------------------------------------

ma3 :SVTDARVPPSSPVSSYNFLYSQVD----------------------------------------------------------------------------

ma4 :PTSTPVSTSAPVPPSAPVPTSTPVSTSAPVPTSTSGQAPQTSAVKAEVATTPKSTTV-------------------------------------------

ma5 :EVSPPATGTVKGTTSGEPSAALSKGGDTVPTVNDPSKEEVAGNGKGTQGSQANKGANSVENAASS-----------------------------------

ma6 :----------------------------------------------------------------------------------------------------

ma7 :V---------------------------------------------------------------------------------------------------

ov1 :IQPTGDPDSPTA----------------------------------------------------------------------------------------

ov2 :----------------------------------------------------------------------------------------------------

ov3 :ASVAQDGAGTSEKEESASDP--------------------------------------------------------------------------------

ov4 :IAGNSRGFVSLDRSVASLPSGSLIKEGLPSNSNGATTT--------------------------------------------------------------

yo1 :QQQQQQQLHSQPQPEPQPEPQPAPQVKVEGQEQVQEQVQEQRADSKQVDGAEITKNVTGQEEKKEIGTENSSSKGKEQQPAAKASEGQQPVNVPEQQAGR

be1 :EPQAKVEGQGQVVEQAQGQVQEREQPNGGKQVSGTETTKNVTGQEETQVNSTGAESSKAKAPSTSVNSSEQQTGKVTEQQPAQQTCQQPARAQIPNFIPQ

ch1 :QVQEQVQEQEQKAKQEDKGKTDKKAGEGTDSKAGQAGKKDGTTEDEASKTKKDSPPAKPEGPQASQPQADQPQVVQPQAPGQPASGAKARLPAG------

yo2 :GPGSAVTPSQTVQPSPGGSGLPPTGSDAGSRSPPTGTPSGSRFPPKGSPTVTVLTSSGGNGAQTQTNLNPASGSVQSNSATTAVNTESQANST-------

be2 :SGSGPTPSGGNNTSHQNNPGQSGASVQPNPSTTVETPENGGGSTERQTNST-------------------------------------------------

ch2 :GQSPSVPTGASGPTPQAPAGPQSPSNPASGVTQDPSNPTPGSNGEQADQAVTETEADAT-----------------------------------------

fa1 :SSNITKSNKNT-----------------------------------------------------------------------------------------

re1 :SSNITKSNKNT-----------------------------------------------------------------------------------------

fa2 :DGKPNTSSSVVDTNDQRSLPNPRATSLQPPSVQIPNHEGTSAPGNSRTPSIVSPTAAEKS----------------------------------------

re2 :DGKPNTSSSAVETNDQRSLPNPGATSLQPPSVQIPNHEGTSAPGRTRTPQVVSPTDAEKS----------------------------------------

fa3 :----------------------------------------------------------------------------------------------------

fa4 :TQGLSPATGDPKGKEQEASPAEGLSGVLNPTKEVTSE---------------------------------------------------------------

re4 :GGSQVTNSSLTESTSSSSGQQENGSLSTQGSSPATGNPKGTEQETSTAEGLSEVLPTAKEDTTE------------------------------------

fa5 :----------------------------------------------------------------------------------------------------

re5 :----------------------------------------------------------------------------------------------------

fa9 :----------------------------------------------------------------------------------------------------

re9 :----------------------------------------------------------------------------------------------------

ga1 :----------------------------------------------------------------------------------------------------

vi10 :----------------------------------------------------------------------------------------------------

cy9 :----------------------------------------------------------------------------------------------------

fi7 :----------------------------------------------------------------------------------------------------

so7 :----------------------------------------------------------------------------------------------------

in5 :----------------------------------------------------------------------------------------------------

hy5 :----------------------------------------------------------------------------------------------------

co5 :----------------------------------------------------------------------------------------------------

kn4 :----------------------------------------------------------------------------------------------------

fr3 :----------------------------------------------------------------------------------------------------

go7 :----------------------------------------------------------------------------------------------------

ma8 :SSSSSSSSSSGSGSGSSTSSSGSGSSTSSSGNNSGDSGTDVNSSVE------------------------------------------------------

ov5 :----------------------------------------------------------------------------------------------------

yo3 :GVTTSAATASIE----------------------------------------------------------------------------------------

be3 :SANAASIE--------------------------------------------------------------------------------------------

ch3 :----------------------------------------------------------------------------------------------------

fa6 :----------------------------------------------------------------------------------------------------

re6 :----------------------------------------------------------------------------------------------------

vi11 :GAVAPQIGAAAPQ-IDVA------APQ-IDVV------APQTRSVDAPQTSSV------------AAHPPNVTPQNVTLGEGQHAGGVGSLIPADNSGE-

cy10 :GAIATQNGADTPQAGDIATQTGADTPQTGDIATQNGADTPQTGDIATQNGADTPQNGVPAPQTTAAASPPSVTPLNVTVEEGQRVGGVVSLIPVDNSGE-

fi8 :--------------------------------------------VDPQTDAVDPQISEAAPQISEAASPPSVTPLNVTVGEDQRVGGVVSLIPVDNSGE-

so8 :------------------------------------------------IDAVEPQNGELPPQMSEDASPPSETPLNVTVGEGQRVGGAVSLIPVDNSGE-

in6 :--------------------------------------------VSPQTSVISPQIGAVAPQIGAVASPPNVTPLNVTAGEDQRVGGVVSLTPMDNSGE-

hy6 :-----------------------------------------------AVGGKAGIASPVGSQMGAI-SP-------------Q-IGEVASLTPVDNSRE-

co6 :----------------------------------------------------------------------------------QMST-VTSLTPVDNSGE-

kn5 :----------------------------------------------NIASPMGSQVNAIAPQVGSVASPTNVAPLNVTVREGQHVGGAVTLTPVDNSGE-

fr4 :-----------------------------------------------AVAGKAGIVAPVGSQTSASASPTNVTPLNVKVGESQVVEDAVLLNPVENSGE-

go8 :-----------------------------------------------------------------------ETPQANPTEVVQNAVPGNVSVPEDNSGE-

ma9 :----------------------------------------------------------------------------------------------------

ov6 :----------------------------------------------------------------------------------------------------

yo4 :----------------------------------------------------------------------------------------------------

be4 :----------------------------------------------------------------------------------------------------

ch4 :----------------------------------------------------------------------------------------------------

fa7 :----------------------------------------------------------------------------------------------------

re7 :----------------------------------------------------------------------------------------------------

vi12 :----------------------------------------------------------------------------------------------------

cy11 :----------------------------------------------------------------------------------------------------

fi9 :----------------------------------------------------------------------------------------------------

so9 :----------------------------------------------------------------------------------------------------

in7 :----------------------------------------------------------------------------------------------------

hy7 :----------------------------------------------------------------------------------------------------

co8 :----------------------------------------------------------------------------------------------------

kn6 :----------------------------------------------------------------------------------------------------

fr5 :----------------------------------------------------------------------------------------------------

go9 :----------------------------------------------------------------------------------------------------

ma10 :----------------------------------------------------------------------------------------------------

ov7 :EDRRFSDMSDDEAYPGLSDNRKNNTI--------------------------------------------------------------------------

yo5 :----------------------------------------------------------------------------------------------------

be5 :----------------------------------------------------------------------------------------------------

ch5 :----------------------------------------------------------------------------------------------------

fa8 :----------------------------------------------------------------------------------------------------

re8 :----------------------------------------------------------------------------------------------------

ga2 :----------------------------------------------------------------------------------------------------

ga3 :SPYPRPNPNPSPYPLPSPAPTPNPYPLPSPAPTPNPYPLPSPSPMPGPYPFPTPFPRPYMRPYGHRYARYFPYSGYYPYRHYYNRGLGNSSDNSEINEDE

392 :------1610------1620------1630------1640------1650------1660------1670------1680------1690------1700

392 :----------------------------------------------------------------------------------------------------

570 :----------------------------------------------------------------------------------------------------

vi1 :----------------------------------------------------------------------------------------------------

cy1 :----------------------------------------------------------------------------------------------------

fi1 :----------------------------------------------------------------------------------------------------

so1 :----------------------------------------------------------------------------------------------------

in1 :----------------------------------------------------------------------------------------------------

hy1 :----------------------------------------------------------------------------------------------------

co1 :----------------------------------------------------------------------------------------------------

kn1 :----------------------------------------------------------------------------------------------------

vi2 :----------------------------------------------------------------------------------------------------

cy2 :VNSTPPSTEPAPKH--------------------------------------------------------------------------------------

fi2 :----------------------------------------------------------------------------------------------------

so2 :----------------------------------------------------------------------------------------------------

in2 :EEGGADPEQAGADPEQAGAEPEEGGAKSEEEGAKSEEEGAKSEEGGAKSEEEGAKSQEGGAKSEEEGAKSQEGGAKSEEEGAKSEEEGAKSQEGGAKSQE

hy2 :------------------------------------------------------KPEEGAAKPEEGAAKPEEGAAKPEEGAAKPEEGAEKPEEGAEKPEE

co2 :----------------------------------------------------------------------------------------------------

go1 :----------------------------------------------------------------------------------------------------

go2 :-------DEHPADEPSKEEEPTA-----------------------------------------------------------------------------

vi3 :-------REPLSSLKGSTGVNVT-----------------------------------------------------------------------------

vi4 :-------PLPGAPTNSTVPTTES-----------------------------------------------------------------------------

vi5 :-------TVEKPKSIDSTGVISE-----------------------------------------------------------------------------

cy3 :-------RAPLSTLTGSTGVIVT-----------------------------------------------------------------------------

cy4 :-------PVPGAPTNSTSATVES-----------------------------------------------------------------------------

cy5 :-------RAPLSTLTGSTGVIVT-----------------------------------------------------------------------------

fi3 :-------RVELSNLTGSTGINVQ-----------------------------------------------------------------------------

so3 :-------RVELSSLTGSTGINVQ-----------------------------------------------------------------------------

in3 :-------GGDPSKRTGSTSLNVE-----------------------------------------------------------------------------

hy3 :-------PVDPSKGTGSTSVDVK-----------------------------------------------------------------------------

co3 :-------PVQLSELTGSTGINVQ-----------------------------------------------------------------------------

kn2 :GAEPPVSDPVETPA-PVELSKLTGTTGISVD---------------------------------------------------------------------

go3 :STSTTTPKAPLSSTTGTSGVNVT-----------------------------------------------------------------------------

vi6 :PAESPPLPNPPSNTA-------------------------------------------------------------------------------------

cy6 :PAESPALSTPPSNAA-------------------------------------------------------------------------------------

fi4 :PAEAPSPSPPPSNAA-------------------------------------------------------------------------------------

so4 :PAESPSPSPPPSDAA-------------------------------------------------------------------------------------

fr1 :PAASSSLSASPSNAA-------------------------------------------------------------------------------------

go4 :HEVHKIAGTHLKNSN-------------------------------------------------------------------------------------

vi7 :----------------------------------------------------------------------------------------------------

vi8 :----------------------------------------------------------------------------------------------------

cy7 :----------------------------------------------------------------------------------------------------

fi5 :----------------------------------------------------------------------------------------------------

so5 :PAQPPSPQQPTEPVHASTSLVTGTPGAANPNAGVQSA---------------------------------------------------------------

in4 :----------------------------------------------------------------------------------------------------

hy4 :----------------------------------------------------------------------------------------------------

co4 :----------------------------------------------------------------------------------------------------

kn3 :----------------------------------------------------------------------------------------------------

fr2 :----------------------------------------------------------------------------------------------------

go5 :----------------------------------------------------------------------------------------------------

vi9 :PGTDSPPVAPEAAV--------------------------------------------------------------------------------------

cy8 :GTEGQPGTAPEAAV--------------------------------------------------------------------------------------

fi6 :GQDVPPSTEPEAAV--------------------------------------------------------------------------------------

so6 :GQDVPPSTEPEAAV--------------------------------------------------------------------------------------

go6 :TQTASVTPPVNTNA--------------------------------------------------------------------------------------

ma1 :----------------------------------------------------------------------------------------------------

ma2 :----------------------------------------------------------------------------------------------------

ma3 :----------------------------------------------------------------------------------------------------

ma4 :----------------------------------------------------------------------------------------------------

ma5 :----------------------------------------------------------------------------------------------------

ma6 :----------------------------------------------------------------------------------------------------

ma7 :----------------------------------------------------------------------------------------------------

ov1 :----------------------------------------------------------------------------------------------------

ov2 :----------------------------------------------------------------------------------------------------

ov3 :----------------------------------------------------------------------------------------------------

ov4 :----------------------------------------------------------------------------------------------------

yo1 :AQAADSITKLQA----------------------------------------------------------------------------------------

be1 :LQVG------------------------------------------------------------------------------------------------

ch1 :----------------------------------------------------------------------------------------------------

yo2 :----------------------------------------------------------------------------------------------------

be2 :----------------------------------------------------------------------------------------------------

ch2 :----------------------------------------------------------------------------------------------------

fa1 :----------------------------------------------------------------------------------------------------

re1 :----------------------------------------------------------------------------------------------------

fa2 :----------------------------------------------------------------------------------------------------

re2 :----------------------------------------------------------------------------------------------------

fa3 :----------------------------------------------------------------------------------------------------

fa4 :----------------------------------------------------------------------------------------------------

re4 :----------------------------------------------------------------------------------------------------

fa5 :----------------------------------------------------------------------------------------------------

re5 :----------------------------------------------------------------------------------------------------

fa9 :----------------------------------------------------------------------------------------------------

re9 :----------------------------------------------------------------------------------------------------

ga1 :----------------------------------------------------------------------------------------------------

vi10 :----------------------------------------------------------------------------------------------------

cy9 :----------------------------------------------------------------------------------------------------

fi7 :----------------------------------------------------------------------------------------------------

so7 :----------------------------------------------------------------------------------------------------

in5 :----------------------------------------------------------------------------------------------------

hy5 :----------------------------------------------------------------------------------------------------

co5 :----------------------------------------------------------------------------------------------------

kn4 :----------------------------------------------------------------------------------------------------

fr3 :----------------------------------------------------------------------------------------------------

go7 :----------------------------------------------------------------------------------------------------

ma8 :----------------------------------------------------------------------------------------------------

ov5 :----------------------------------------------------------------------------------------------------

yo3 :----------------------------------------------------------------------------------------------------

be3 :----------------------------------------------------------------------------------------------------

ch3 :----------------------------------------------------------------------------------------------------

fa6 :----------------------------------------------------------------------------------------------------

re6 :----------------------------------------------------------------------------------------------------

vi11 :----------------------------------------------------------------------------------------------------

cy10 :----------------------------------------------------------------------------------------------------

fi8 :----------------------------------------------------------------------------------------------------

so8 :----------------------------------------------------------------------------------------------------

in6 :----------------------------------------------------------------------------------------------------

hy6 :----------------------------------------------------------------------------------------------------

co6 :----------------------------------------------------------------------------------------------------

kn5 :----------------------------------------------------------------------------------------------------

fr4 :----------------------------------------------------------------------------------------------------

go8 :----------------------------------------------------------------------------------------------------

ma9 :----------------------------------------------------------------------------------------------------

ov6 :----------------------------------------------------------------------------------------------------

yo4 :----------------------------------------------------------------------------------------------------

be4 :----------------------------------------------------------------------------------------------------

ch4 :----------------------------------------------------------------------------------------------------

fa7 :----------------------------------------------------------------------------------------------------

re7 :----------------------------------------------------------------------------------------------------

vi12 :----------------------------------------------------------------------------------------------------

cy11 :----------------------------------------------------------------------------------------------------

fi9 :----------------------------------------------------------------------------------------------------

so9 :----------------------------------------------------------------------------------------------------

in7 :----------------------------------------------------------------------------------------------------

hy7 :----------------------------------------------------------------------------------------------------

co8 :----------------------------------------------------------------------------------------------------

kn6 :----------------------------------------------------------------------------------------------------

fr5 :----------------------------------------------------------------------------------------------------

go9 :----------------------------------------------------------------------------------------------------

ma10 :----------------------------------------------------------------------------------------------------

ov7 :----------------------------------------------------------------------------------------------------

yo5 :----------------------------------------------------------------------------------------------------

be5 :----------------------------------------------------------------------------------------------------

ch5 :----------------------------------------------------------------------------------------------------

fa8 :----------------------------------------------------------------------------------------------------

re8 :----------------------------------------------------------------------------------------------------

ga2 :----------------------------------------------------------------------------------------------------

ga3 :NGEILGIYDQGKLSGSNFDDSLLDIKPESSNADNEGVLQKHNISSDNSDIITLDIETELDPQIGTIEDSTNKKSLEQ-----------------------

392 :------1710------1720------1730------1740------1750------1760------1770------1780------1790------1800

392 :----------------------------------------------------------------------------------------------------

570 :-----------------------------------------------------------------------------------------------#####

vi1 :-------------------------------------------------------------------------------------------ATVEVMHIL

cy1 :-------------------------------------------------------------------------------------------ATVEVLHIL

fi1 :-------------------------------------------------------------------------------------------ATVEVLHIL

so1 :-------------------------------------------------------------------------------------------ATVEVLHIL

in1 :-------------------------------------------------------------------------------------------ATVEVMHIL

hy1 :-------------------------------------------------------------------------------------------STVEVMHIL

co1 :-------------------------------------------------------------------------------------------ATMEVMHIL

kn1 :-------------------------------------------------------------------------------------------ATTEVLHIL

vi2 :-----------------------------------------------------------------------------------NASLIKVKQITEVIHIM

cy2 :-----------------------------------------------------------------------------------NTSLIKVKQIMEVVHII

fi2 :-----------------------------------------------------------------------------------STSLIKVKQIMEVIHIL

so2 :-----------------------------------------------------------------------------------NTSLIKVKQIMEVIHIL

in2 :GGAKSQEGGAKSQEGGAKSQEGGAKSQEGDSETAKKGVEAEQPSKVAVSDASPEGVKGPQTVASPSASVNSTPPPSSTPAPTRKTSLIKVKQIMEVIHII

hy2 :GAEKPEEGAEKPEEGGAKPEEAGAKTEEGDPETAKKGVETEEPNKVAVSDAPPEGVKEPQTVASPKESVNSTPPLSSAPVATHKPSLIKVKQIMEVIHIL

co2 :-----------------------------------------------------------------------------------NISLIKVKQIMEVIHIM

go1 :-----------------------------------------------------------------------------------NISMSKIVQITEVIHIL

go2 :-------------------------------------------------------------------------------------------NKIQLVHIL

vi3 :-------------------------------------------------------------------------------------------EVKEALHFL

vi4 :-------------------------------------------------------------------------------------------EVKEVLHIL

vi5 :-------------------------------------------------------------------------------------------GITGVFHFL

cy3 :-------------------------------------------------------------------------------------------EVKEALHFL

cy4 :-------------------------------------------------------------------------------------------EVKEVLQIL

cy5 :-------------------------------------------------------------------------------------------EVKEALHFL

fi3 :-------------------------------------------------------------------------------------------GITGVLHFL

so3 :-------------------------------------------------------------------------------------------GITGVLHFL

in3 :-------------------------------------------------------------------------------------------GITRVLHFL

hy3 :-------------------------------------------------------------------------------------------GITGVLHFL

co3 :-------------------------------------------------------------------------------------------GVTKVLHFL

kn2 :-------------------------------------------------------------------------------------------GVTRVLHFL

go3 :-------------------------------------------------------------------------------------------GVTGVLHFL

vi6 :-------------------------------------------------------------------------------------------KITQVLHIL

cy6 :-------------------------------------------------------------------------------------------KITQVLHLL

fi4 :-------------------------------------------------------------------------------------------KITQVLHIL

so4 :-------------------------------------------------------------------------------------------KITRVLHIL

fr1 :-------------------------------------------------------------------------------------------KITQVLHIL

go4 :-------------------------------------------------------------------------------------------KIIQVLHIL

vi7 :-------------------------------------------------------------------------------------------ESKEVLHIL

vi8 :-------------------------------------------------------------------------------------------NISQIIHVL

cy7 :-------------------------------------------------------------------------------------------KMSHIIHVL

fi5 :-------------------------------------------------------------------------------------------KISQIIHVL

so5 :-------------------------------------------------------------------------------------------KISQIIHVL

in4 :-------------------------------------------------------------------------------------------KISQIVHVL

hy4 :-------------------------------------------------------------------------------------------KLPHIVHVL

co4 :-------------------------------------------------------------------------------------------KIVQIIHVL

kn3 :-------------------------------------------------------------------------------------------KMVQIIHVL

fr2 :-------------------------------------------------------------------------------------------QLPQIIHVL

go5 :-------------------------------------------------------------------------------------------KKTHVIHIL

vi9 :-------------------------------------------------------------------------------------------LGSEVTHVL

cy8 :-------------------------------------------------------------------------------------------LDTQVSHVL

fi6 :-------------------------------------------------------------------------------------------LGTQVTHVL

so6 :-------------------------------------------------------------------------------------------LGTQVSHVL

go6 :-------------------------------------------------------------------------------------------TSTQMLHIL

ma1 :-------------------------------------------------------------------------------------------AKTGVLHVL

ma2 :-------------------------------------------------------------------------------------------KKLQFVHVL

ma3 :-------------------------------------------------------------------------------------------EKTKILHIL

ma4 :-------------------------------------------------------------------------------------------ADVKVFHVL

ma5 :-------------------------------------------------------------------------------------------ESIEIIHIL

ma6 :-------------------------------------------------------------------------------------------QKFEVVHIL

ma7 :-------------------------------------------------------------------------------------------NNKKIVHIL

ov1 :-------------------------------------------------------------------------------------------DNVEVLHML

ov2 :-------------------------------------------------------------------------------------------PNIEVVHIL

ov3 :-------------------------------------------------------------------------------------------KKVEVVHIL

ov4 :-------------------------------------------------------------------------------------------SNAEVVHIL

yo1 :-------------------------------------------------------------------------------------------VKIEFMHIL

be1 :-------------------------------------------------------------------------------------------IRLEFMHIL

ch1 :-------------------------------------------------------------------------------------------PKLEFMHVL

yo2 :-------------------------------------------------------------------------------------------KNTEVFHVL

be2 :-------------------------------------------------------------------------------------------QNTEIFHVL

ch2 :-------------------------------------------------------------------------------------------QNTEVFHVL

fa1 :-------------------------------------------------------------------------------------------NKVKIYHII

re1 :-------------------------------------------------------------------------------------------NKVKIYHII

fa2 :-------------------------------------------------------------------------------------------RKAQIFHVL

re2 :-------------------------------------------------------------------------------------------RKAQIFHVL

fa3 :-------------------------------------------------------------------------------------------EKVDIFHVL

fa4 :-------------------------------------------------------------------------------------------EKIQIIHLL

re4 :-------------------------------------------------------------------------------------------EKIQIIHVL

fa5 :-------------------------------------------------------------------------------------------ERVHVYHIL

re5 :-------------------------------------------------------------------------------------------ERVHVYHIL

fa9 :-------------------------------------------------------------------------------------------KKMDVFQVL

re9 :-------------------------------------------------------------------------------------------KKMDVFQVL

ga1 :-------------------------------------------------------------------------------------------QKETIRHIL

vi10 :-------------------------------------------------------------------------------------------KKIQILHVL

cy9 :-------------------------------------------------------------------------------------------KKIQILHVL

fi7 :-------------------------------------------------------------------------------------------KKIQILHVL

so7 :-------------------------------------------------------------------------------------------KKIQILHVL

in5 :-------------------------------------------------------------------------------------------KKIQILHVL

hy5 :-------------------------------------------------------------------------------------------KKIQILHVL

co5 :-------------------------------------------------------------------------------------------KKIQILHVL

kn4 :-------------------------------------------------------------------------------------------KKIQILHVL

fr3 :-------------------------------------------------------------------------------------------KKIQILHIL

go7 :-------------------------------------------------------------------------------------------KNIQILHIL

ma8 :-------------------------------------------------------------------------------------------KNIQILHIL

ov5 :-------------------------------------------------------------------------------------------KNIQIMHIL

yo3 :-------------------------------------------------------------------------------------------KKIQILHVL

be3 :-------------------------------------------------------------------------------------------KKIQILHVL

ch3 :-------------------------------------------------------------------------------------------KTIQILHIL

fa6 :-------------------------------------------------------------------------------------------KKIQILHIL

re6 :-------------------------------------------------------------------------------------------KKIQILHIL

vi11 :-------------------------------------------------------------------------------------------KNIPVMHLL

cy10 :-------------------------------------------------------------------------------------------KKIPVMHIL

fi8 :-------------------------------------------------------------------------------------------KKIPVMHIL

so8 :-------------------------------------------------------------------------------------------KKIPVMHIL

in6 :-------------------------------------------------------------------------------------------KKIPVMHIL

hy6 :-------------------------------------------------------------------------------------------KKIPVMHIL

co6 :-------------------------------------------------------------------------------------------KKIPVMHIL

kn5 :-------------------------------------------------------------------------------------------KKIPVTHIL

fr4 :-------------------------------------------------------------------------------------------NKIPITHIL

go8 :-------------------------------------------------------------------------------------------KKINVVHIL

ma9 :-------------------------------------------------------------------------------------------KIFKVLHIL

ov6 :-------------------------------------------------------------------------------------------KKIDVLHIL

yo4 :-------------------------------------------------------------------------------------------KKLDITHVL

be4 :-------------------------------------------------------------------------------------------KKIDITHVL

ch4 :-------------------------------------------------------------------------------------------KKLDVTHVL

fa7 :-------------------------------------------------------------------------------------------KKTEIYHIL

re7 :-------------------------------------------------------------------------------------------KKTEIYHIL

vi12 :-------------------------------------------------------------------------------------------RRKVFKTSL

cy11 :-------------------------------------------------------------------------------------------RRKVFKTSL

fi9 :-------------------------------------------------------------------------------------------RRTVFKTSL

so9 :-------------------------------------------------------------------------------------------RRTVFKTSL

in7 :-------------------------------------------------------------------------------------------RRKVFKTTL

hy7 :-------------------------------------------------------------------------------------------RRRVFKTTL

co8 :-------------------------------------------------------------------------------------------RRTVFKTSL

kn6 :-------------------------------------------------------------------------------------------RRTIFKTTL

fr5 :-------------------------------------------------------------------------------------------RRTVFRTSL

go9 :-------------------------------------------------------------------------------------------RHKVFRTTL

ma10 :-------------------------------------------------------------------------------------------FRRTFLTNL

ov7 :-------------------------------------------------------------------------------------------LRKFFKTTF

yo5 :-------------------------------------------------------------------------------------------LRRVFQINL

be5 :-------------------------------------------------------------------------------------------LRKIFQTDL

ch5 :-------------------------------------------------------------------------------------------LRRIFQTDL

fa8 :-------------------------------------------------------------------------------------------KKRIIKYSL

re8 :-------------------------------------------------------------------------------------------KKRIIKYSL

ga2 :-------------------------------------------------------------------------------------------LRRVFKSQI

ga3 :-------------------------------------------------------------------------------------------NEKTFKTDV

392 :------1810------1820------1830------1840------1850------1860------1870------1880------1890

392 :------------------------#############-###################----##############-######

570 :#####################---#############-###################----##############-######

vi1 :KYIKNGKVKLGIATYRDDSDIAGDHSCSRSLAQNSEQ-LAECIQFCHDEWPNCRGEA----SPGYCLAQRRRTGD-CFFCYV*

cy1 :KHIKHGKVKLGIVTYGDESDISGDHSCSRSLAQDSEQ-LTECIQFCHNEWPNCRDEA----SPGYCLAQRRKTGD-CFFCYV*

fi1 :KHIKHGKVKLGIVTYGYGSDITGDHSCSRSLAQDSEQ-LAECIQFCHDEWPNCRDEA----SPGYCLAQRRKMGD-CFFCYV*

so1 :KHIKNGKVKLGIVTYDDDSDIAGDHSCSRSLAQDSEQ-LAECIQFCHDEWPNCRDEA----SPGYCLAQRRKAGD-CFFCYV*

in1 :KYIKHGKVKLGIVTYGDYSGIAGDHSCSRSLAQDSEQ-LAECIQFCHDEWPNCRDEA----SPGYCLAQRRKTGD-CFFCYV*

hy1 :KHIKHGKVKLGIVTYSDYSGIAGDHSCSRSLAQDSEQ-LAECIQFCHDEWANCRDEP----SPGYCLAQRRKTGD-CFFCYV*

co1 :KHIKHGKVKLGIVTYGSYSNIAGDHSCSRSFAQDPEK-RDECIQFCHDEWNNCKGEA----SPGYCLAQRRKTGD-CFFCYV*

kn1 :KHIKNGKVKLGKTTYGNGSNITGYHSSSRSLA*NPGK-RTECIQFCHNEWNNCKDDI----SPGYCLAKRRKTGD-CFFCYV*

vi2 :KHIKSGKLRFGIATYEDDLGIANNHDCLRSYSQDPEK-LPECIQFCYDEWNNCKGAP----SPGYCLNQRRRKND-CYFCFV*

cy2 :KHIKNGKVRFGITTYEDDMGIANKHDCSRSYSQDPEK-LAECIQFCHDEWNNCKGEP----SPGYCLNKLRRKND-CFFCFV*

fi2 :KHIKSGKVRFGITTYEDDMGIANSHDCSRSYSQDPEK-LDECIQFCYDEWNNCKGEP----SPGYCLNQRRRKND-CFFCFV*

so2 :KHIKNGKLRFGITTYEDDMGIANKHDCSRSYSQDQEK-LDECIQFCHDEWNNCKGEP----SPGYCLNQRRRKND-CFFCFV*

in2 :KHIKNGKLRLGITTYEDDLGIANKHDCSRSYSRDPKK-LPECIQFCHDEWNNCKGEF----SPGYCLNQRRRKND-CFFCYV*

hy2 :KHIKDGKLRLGITTYEDDLGIANKHDCSRSYSKDPKN-LADCIQFCHDEWNNCKGEL----SPGYCLNQRRGKND-CYFCYV*

co2 :KHIKSGKLRLGITTYKDDLDLANKHDCSRSYSQDPEK-RDECIQFCHDEWNNCKGEA----SPGYCLTQRRRKND-CFFCYV*

go1 :KHIKNEKLRYGIVTYEDDTSIAHDHNCSRSYAEDSNK-LEECIQFCNDEWNNCKGDP----SPGYCLAKRRKKND-CYFCFV*

go2 :KHISNGKVKMNLLTYDTENAISGDHFCSRSHSVDPEK-HSECVSFCNEKWEECKSEP----SPGYCITQKKGNNH-CFFCFV*

vi3 :KSVKNGKVKSNFVAYVNADAMGDEKVCSRAFSTDADK-QTECIEFCEKNWDACKGKV----SPGYCLTKKRGSND-CFFCFV*

vi4 :KHIKKGKVKVGLVTYDTEKVIGAEKVCSRAYAVDHEK-QEECVEFCEANWDACKGKV----SPGYCLTKKRGSND-CFFCFV*

vi5 :KNVKKGKVSSNFVTYDNTKAIGD-KACSRVQSSDVDK-LDDCVKFCEANWNECKGKV----SPGYCLTKKKGNND-CFFCFV*

cy3 :KNVKNGKVKSNFVSYDNADALGDEKVCSRAFSTDVDK-QAECIEFCEKNWGACKGKV----SPGYCLTKKRGSND-CFFCFV*

cy4 :KHIKNGKVKVGLVTYDTEKRIGAEKVCSRAYAVDHEK-QEECVEFCEANWDACKSTV----SPGYCLTKKRGSND-CFFCFV*

cy5 :KNVKNGKVKSNFVSYDNADALGDEKVCSRAFSTDVDK-QAECIEFCEKNWDACKDKV----SPGYCLTKKRGSND-CFFCFV*

fi3 :KNVKNGKVTSNFVTYDNEKAIGD-KSCSRVQSSDIDK-LDDCVKFCEANWDACKGTV----SPGYCLTKKKGNND-CFFCFV*

so3 :KNVKNGKVTTNFVTYDNEKAIGD-KSCSRVQSPDIDK-LDDCVKFCEANWDACKGKV----SPGYCLTKKRGNND-CFFCFV*

in3 :KNVKNGKVTSNFVAYDSEKVIGD-KACSRVQTSDIDK-LDECVNFCEKHWNDCKGKV----SPGYCLTKKRGNND-CFFCFV*

hy3 :KNVKNGKVTSNFVAYDSDKVLGD-KACSRVQTSDIDK-LAECVNFCEENWNDCKGKV----SPGYCLAKKRGNND-CFFCFV*

co3 :KNVKNGKVSSSFVTYDDESAIGD-KSCSRVQSSDVDK-LEDCVKFCEENWNACKGSI----SPGYCLTKKKGNND-CFFCFV*

kn2 :KNVKNGKVTSSFVTYDNESAIGD-KSCSRVQSSDLDK-LDDCVKFCEENWDACKGSV----SPGYCLTKKRGNNE-CFFCFV*

go3 :KNVKNGKVKTGFVTYDKATAIGDAKVCSRAHSSDVEK-INDCIKFCEDKWDECKGTV----SPGYCLAKKKGNND-CFFCFV*

vi6 :KHVKQGKVKTRLVTYDSNAAMSADHVCSRAVASDPEK-QDECVKFCDDHWNDCKDKI----SPAYCLTQIRGKKD-CFFCYI*

cy6 :KHVKHGKVKTRLVTYDSDAAISAGHVCSRAVATDPEK-QDECVKFCDDHWNECKDKI----LPSYCLTTKRGNND-CFFCYI*

fi4 :KHVKQGKVKTRLVTYGSDAAMSAGHVCSRAVANDPEK-QDECVKFCDDHWNECKDKI----LPSYCLTNKRGTND-CFFCYI*

so4 :KHVKQGKVKTRLVTYGSDAAMSAGHVCSRAVANDPEK-QDECVKFCDDHWNDCKDKI----LPAYCLTKMMGTND-CFFCYI*

fr1 :KHVKHGKVKTGLVTYEGDAALSAGHACSRAVASDPDIGDDECVKFCDDNWNECKDKF----LPTYCLTNKRGKND-CFFCYV*

go4 :KHIKHGNVKTGLITYKLNEAMNTEHVCSRSSSDYPEK-QEECIKFCDDHWNECKDKI----SPAYCLTKKRGNND-CFFCYI*

vi7 :KHVKDNKVKTNLVTYSTTKSITDDHSCSRSHAQDPEK-QSECIDFCEKNWNACADKI----SPGYCLMQKRGKND-CIFCYV*

vi8 :KHIKKTKMVTRIVTYEGEYDLGD-HSCSRTQASSLEK-LDDCIKFCNDNLSMCERTV----STGYCLTKLRKAND-CIFCFV*

cy7 :KHIKQTKMVTRMVTYEGEYELGD-HSCSRTQASTVEK-LDECIRFCNENLYLCERTV----SAGYCLTKLRNAND-CIFCFV*

fi5 :KHIKQTKMVTRVVTYEGEYELGD-HSCSRTQASSLEK-LDECIRFCNENFSMCKSTV----SVGYCLTKLRQAND-CIFCFV*

so5 :KHIKQTKMVTRVVTYEGEYELGD-QSCSRTQALSLEK-LDECIRFCNDKLSTCKRTV----SVGYCLTKLRQTND-CIFCFV*

in4 :KHIKQTKMVTRIVTYEGDYEFGD-HSCSRTEATTLDK-LDECIRFCNENLSMCERTV----SPGYCLTNLRKAND-CIFCAV*

hy4 :KHIKQTKMVTRIVTYEGGYELGD-PSCSRTQATTVEK-LDECIGFCNENLDMCKRTV----SPGYCLTKLRKTND-CIFCSV*

co4 :KHIKQTKMVTRVVTYEGLYDLGD-PSCSRTEASSVEK-LDECIKFCNENYFRCKLTV----SPGYCLTKLRNAND-CNFCFV*

kn3 :KHIKQTKMVTRVVTYEGNYELGE-HSCSRTEASSVEK-LDECIKFCDEYYSECKLSI----SPGYCLTKKRNAND-CNFCFV*

fr2 :KHIKQTKMVTRAVTYEGDYELGD-HSCIRTEASSVEK-LDECLQFCNENFYKCKGTI----SPGYCLTKLHETND-CNFCSV*

go5 :KHIKETKMTTRVVTYNGEYELGD-HACSRTQAQSWEK-QEECLKFCNDNLPECPRNV----SVGYCLTKLRKSND-CIFCFV*

vi9 :KYIKKNKVKLNLVTYKNHEALSSGHDCWRSYSANPDK-YEECVKLCEANWSKCENDA----APGFCLYEHAKEED-CFFCYV*

cy8 :KYIKKNKVKMNVVSYKNHEAITTGHDCWRSYSVNPDK-YEECVKICEANWSKCENDV----APGFCLFEHGKDND-CFFCYV*

fi6 :KYIKKNKMKMNLITYKNHEAITTGHDCWRSYSVNPDK-YEECVKLCEANWSKCEGDA----VPGFCLYEHAKETD-CSFCYV*

so6 :KYIKKNKMKMNLITYKNHEVITTGHDCWRSYSVNPDK-YEECVKLCEANWSKCENDA----VPGFCLYEHAKNND-CSFCYV*

go6 :KRIKNSKMKTHLVMYKSHETITSGHDCSRSLSINPEK-FEECVKICNENWSKCEDDP----IPGICLSKHDADKY-CIFCYV*

ma1 :KHIKNGQIKMGLVKYEDNAIIIDNHACSRSYAMDVNN-LDECIKFCSEEWDNCKDEP----SPGYCLTKRQSTNK-CFFCYV*

ma2 :EHVKNGKVKVDIVKYDSDNPINADHICSRARSYPNYEIKDECVTFCNENWGACSEHK----SPGYCLTQKRKTDD-CFFCFV*

ma3 :KHVKDGKLKIGLAKYDILKSASGDHVCSRSYAYDPGK-QEECFQFCERHWNECRGSY----SPGYCLSKKRKKND-CYFCYV*

ma4 :KYIKNTKVKMNLVKYNTRAAIDADHVCSRSYSADQDK-LEDCIKFCESNWNECMGEI----SPGYCLTQKRGKND-CFFCYI*

ma5 :KHIKNSNVKMGLVKYDHEYEVGE-HSCMRTSSTNPEK-HSDCVKFCKEKFEECEKEP----IPGICLANLYKSDD-CFFCYV*

ma6 :KHIKNSKSKTTLVKYDYYYDFGD-HACSRTQASNPEK-LGDCISFCNNNWDTCKRSV----SPGYCLTKLKKTNK-CLFCFV*

ma7 :KLIKDDHVKWNLIKYKNKDSINEDHDCSRSYSPKEGK-YEYCVKLCDDKWNDCKDEE----LPGNCLSKFDTANE-CFFCYV*

ov1 :KNVKNNKVKISLVTYETSKGITADHVCSRAYSEDPDK-LEECAQFCESNWNDCANKI----SPGYCLTLKRKTND-CFFCYV*

ov2 :KHIKNTKVDMGLVKYEQAYGIGD-HSCSRAYSVDPDK-HEKCVDFCKQKLKECTNND----IPGICLSQKYEMDD-CFFCYV*

ov3 :KYIKNNKIQSSLIKYDYEYGLGD-HACSRSHAIDPEK-QDECISFCNDKWDDCKRTI----SPGYCLTKLKGTNE-CIFCFV*

ov4 :KHIKNTKRKVNIVKYKNAECISADHDCSRSYALKMEN-HEKCVQICEDKLSDCLKDASPSTSPGFCLSKFDEKKE-CFFCYV*

yo1 :KQIKNGRVQMNLVKYGDELNMIDDRMCSRVHAINYDK-KDECEKFCITKWSECQTAT----SPGYCLSKLYESND-CYFCYV*

be1 :KQIKNGKVKMNLVKYGSELSMIDDRICSRVYAINPEK-KNECEQFCAAKWSICQFDI----SPGYCLAKLYNSDD-CYFCYV*

ch1 :KQIKNGKVKMNLVKYGNELNLIDERACSRVYAVNPEK-KSECEEFCVNKWNECKDSE----VPGYCLADLYNSNN-CYFCFV*

yo2 :KVVRNKKVQTSLIKYNKYNEMG-EHSCSRAMSRDPLN-KEKCIQFCSDNWDHCYFDL----YPGYCLNKLKRDNE-CNFCAV*

be2 :KVIRKKKVQTSLIKYNTYDEVE-EHSCSRAMSTDSSK-KEKCVTFCSNNWDSCSFNL----YPGYCLNKLKGDNE-CNFCAV*

ch2 :KVVRNKKVQTSLLKYGTYTEMG-QHSCARAMSTDPTK-KEECVAFCNNNWDDCYFDL----YPGYCLNKLKGDNV-CNFCAV*

fa1 :KHVKNTKIKIGFVKYDNYNTIGTNHTCSRSYSEDQEK-HEGCIKFCELHWNECKDKT----SPGYCLTKLKGSNE-CFFCYV*

re1 :KHVKNTKIKIGFVKYDNYNTIGTNHTCSRSYSEDQDK-HEGCIQFCELHWNECKDKT----SPGYCLTKLKGSNE-CFFCYV*

fa2 :KHIKNSRIKMGLVKYDNSDNIGGDHVCSRTYAVNPEK-QEECVKFCEENWENCKNKP----SPGYCLAKLKNTNE-CFFCYV*

re2 :KHIKNSRIKMGLVKYDNSDNIGGDHVCSRTYAVNPEK-QEECVKFCDENWENCKNKP----SPGYCLAKLKNTNE-CFFCYV*

fa3 :KHIKDSKIKMGIVKYDHSDALGEDNVCSRSYSSNPEK-QEGCVKFCNENWGKCKDAA----SPGFCLSELEKTND-CFFCYI*

fa4 :KHIKNSKIRRGLVKYNHEFEVG-DNSCSRSTSKNAEM-HDECVNICEKYWPECRGTA----VPGYCLSTHDDKNE-CDFCYV*

re4 :KHIKNSKIRRGLVKYNHEFEVG-DNSCSRSTSKNAEM-HEKCVNICEKNWPECRGTA----VPGYCLSTYDDKNE-CDFCYV*

fa5 :KHIKDGKIRMGMRKYIDTQDVNKKHSCTRSYAFNPEN-YEKCVNLCNVNWKTCEEKT----SPGLCLSKLDTNNE-CYFCYV*

re5 :KHIKDGKIRMGIRKYMDTQDVKKKHSCTRSYAFNPDN-YEKCVNLCNENWQKCEEKT----SPGLCLSKLDTNNE-CYFCYV*

fa9 :KHVKNSKIKVGLIKYDNDDEIGAHHSCTRAYSFDPEK-YDECVEFCRKNWEQCKDKP----SPGFCLTKLDESKG-CFFCYV*

re9 :KHVKNSKIKVGLIKYDNDDEIGAHHSCTRAYSFDPEK-YDECVEFCRKNWEQCKDKP----SPGFCLTKLDETKG-CFFCYV*

ga1 :KDIKNKRIKSSLISYADEDDIGKEHSCSRTYSSDPAK-DAECKQFCVQNFDKCKSQY----SPGYCLTKLKKTDE-CFFCAV*

vi10 :KHIENSKIVRGLVKYENISETQNDHSCARAHSKNPDR-LDECKLFCEENWNSCKNHY----SPGYCLAKLYSGGN-CYFCYV*

cy9 :KHIDKSKIVRGLVKYENISETQNDHSCARAHSKNPDR-KDECKLFCEENWNSCKNHY----SPGYCLAKLYSGGN-CYFCYV*

fi7 :KHIENSKTMRGLVKYENISETQNEHSCARAHSKNPDR-MEECKLFCEENWNSCKNHY----SPGYCLAKLYSGGN-CYFCYV*

so7 :KHIENSKIVQGLVKYENISETQNDHSCARAHSKNPDR-MEECKLFCEENWNSCKNHY----SPGYCLTKLYSGGN-CYFCYV*

in5 :KHIDKSKIVRGLVKYDNISDTQNDHSCARAHSKNPGK-MDECKVFCEENWNSCKNHY----SPGYCLTKLYSGGN-CYFCYV*

hy5 :KHIDKSKIVRGLVKYDNISDTQNDHSCARAHSKNPDK-MDECKLFCEENWNSCKNHY----SPGYCLTKLYSGGN-CYFCYV*

co5 :KHIDKAKIMRGLVKYENISETQNDHSCARAHSKNPDK-MEECKLFCEENWNSCKNHY----SPGYCLTKLYNGSN-CYFCYV*

kn4 :KHIDKAKIMRGLVKYENISETQNDHSCARAHSKNPEK-IEECKLFCEENWNNCKNHY----SPGYCLSKLYSGSN-CYFCYV*

fr3 :KHIDNSKIMRGMVKYDNISETQNDHSCARVHSKNPDR-MEECKTFCEENWNSCKNHY----SPGYCLSKLYNGGN-CYFCYV*

go7 :KHIENSKIVKGLVKYENINETQNEYTCSRSHSKNPDR-YDECKQFCEENWNSCKNHY----SPGYCLTKLYNGSN-CYFCYV*

ma8 :KHIENSKITRSFVKYDNINETKEDHTCSRSHSKDPAK-HDECKQFCESNWNTCKGHY----SPGYCLTKLYNGGN-CYFCYV*

ov5 :KHIEKAKITKGLVKYDNINETKNDHTCVRSNSKDPDR-YEECKLFCEENWNACKKHY----SPGYCLTKLYNGAN-CFFCYV*

yo3 :KHIENYKMTRGLVKYDNLNDTKSDYACARSYAYDPKN-HNECKQFCEENWERCKDHY----SPGYCLTTLSGKNK-CLFCYV*

be3 :KHIEKYKMTRGFVKYDNLNDTKNDYTCARSYSYDPKN-HNECKQFCEENWERCKNHY----SPGYCLTTLSGKNK-CLFCYV*

ch3 :KHIENYKMTRGLVKYDNLNDTKHDYACARSSSNDPKK-VNECKQFCEENWERCKKHY----SPGYCLTALAGKSS-CLFCYV*

fa6 :KHIKDSQIKRGLVKYDNINETKDEHTCSRVNSQDAEK-YEECKKFCLTKWNECKDHY----SPGYCLTDLYKGED-CNFCYV*

re6 :KHIKDSQIKRGLVKYDNINETKDEHTCSRVNSEDSEK-YEECKKFCLTKWNECKDHY----SPGYCLTDLYKGED-CNFCYV*

vi11 :KYVKETKMTRAFVTYGSLSEIEGSHSCSRIYAKDEEG-HEECAQFCLKNWPACRGHY----SPGYCLTKMYTGSN-CLFCSV*

cy10 :KYVKETKMTRAFVRYDSLNEIESSHSCSRTYAKDEKG-HEECIQFCLKNWASCRGHY----SPGYCLTKMYAGSN-CFFCSV*

fi8 :KYVKKTKMTRTFVSYDSLSEIESSHSCSRTYAKDEKG-HEECTQFCLKNWASCRGHY----SPGYCLTKMYTGSN-CIFCSV*

so8 :KYVKKTKMTRAFVSYDSLSEIEDAHSCSRTYAKDENG-HEECTQFCLKNWASCSGHY----SPGYCLTKMYTGSN-CIFCSV*

in6 :KYVKETKMTKASVRYDSLREIEGSHSCSRSYARDGKG-REECTQFCLKNWNSCRGHY----SPGYCLTKMYTGSN-CIFCSV*

hy6 :KYVKETKMTKASVRYDSLREIEGTHSCSRSYARDGKG-REECTHFCLKNWPSCRGHY----SPGYCLTRMYKGSN-CIFCSV*

co6 :KYVKENKMTKASVKYNSLSEIEGSHSCSRTYALDEEG-HAECIQFCLKNWTSCKGHY----NPGYCLTKMYTGNN-CIFCSV*

kn5 :KYVKETKMTKTLVNYDSLSEIEDSHSCARTYAKDEVG-QEECIQFCLKNWSSCKGHY----NPGYCLTKMYTGNN-CIFCSV*

fr4 :KYVKGNKVTKTFVKYDSLSEIEGAHSCSRSYAKDGKG-QEECTQFCLENWASCKGHY----YPGYCLTKMYTGTN-CIFCSV*

go8 :KYIKETKIKRTFVRYDSLSEIESAHTCSRVYAKHDEK-YDECLQFCMQNWTTCKNHY----SPGYCLTKMYVGNN-CFFCNV*

ma9 :KNIKDGKIKRGFVKYENLKETENPYSCSRIYSKDPNK-MDECKQFCFKEWSKCEKHY----SPGYCLTKLYSGDN-CFFCNI*

ov6 :KYVKDKKVRTGFVKYDDIEETERGYSCSRIYSKNSEK-HNLCKEFCLKNWNVCKNHN----SPGYCLTELYPDDS-CFFCNI*

yo4 :KYVKNNKTQTSFIKYDDILEIEHEHNCSRVQSIGLKN-KNECKSFCLENWVKCKNHP----SPGYCLTTLYSGED-CFFCNI*

be4 :KYVKNNKTQTSFIKYDDILEIEYEHNCSRVQSIGLKN-KNECKSFCLENWIKCKNYT----SPGYCLATLYSGED-CFFCSI*

ch4 :KYVKNNQTQTSFIKYDDILEIEQEHNCSRVQSIGLKN-KNECKSFCLENWSTCKNHP----SPGFCLTTLYSAED-CFFCNI*

fa7 :KHIKDKKIKRGLVKYESLLETKKDHSCSRTHSIDPEK-HEECNQFCIDNWKACKDHY----SPGYCLTKLYTKDDNCFFCNV*

re7 :KHIKDKKIKRGLVKYESLLETKKDHSCSRTHSIDPEK-HEECNQFCIDNWKACKDHY----SPGYCLTKLYTKDDNCFFCNV*

vi12 :VVTIGDTQFKRDIFMKRKEEYKEQHSCLRTYSLDSLA-DVSCRNNCEQYIDLCKHYT----SIGRCLMRFANNYK-CVYCGM*

cy11 :VVNIGDTQFNRIIYMKRKDEYKEQHSCLRTYSLDSLA-DVSCRNNCNQYIDLCKHYT----SIGMCLMRFANNYK-CVYCGM*

fi9 :VVKIGDTQFKRNIYMKRKDEYKEQHSCLRTYSLDSLA-DVSCRDNCNQYIDLCKHYT----SIGRCLMRFARNYK-CVYCGM*

so9 :VVNIGDTQFKRNIYMKRKDEYKEQHSCLRTYSLDSLA-DVSCRNNCNQYIDLCKHYT----SIGRCLQRFARNYK-CVYCGM*

in7 :LLKIGDTHFRRDIYMRRKDEYKERHSCLRTYSLNSLA-DVSCRNNCHKYIDLCSHYT----YVGRCLMRHAENYK-CVYCGME*

hy7 :LLKIGDRHFRRDIYMRRKDEYKERHSCLRTYSLDSRA-DVLCRNNCHKHIDLCSHYT----SVGRCLMRYAANYK-CVYCGME*

co8 :VVNIGDTQFKRNIYMKKKDEYKEQHSCLRTYSLDSQA-DVSCRDNCNQYIDLCKHYT----SIGRCLMRFAPNYK-CVYCGM*

kn6 :VVNIGDTNFKRNIYMKKKDEYKEQHSCLRTYSLDSQA-DVSCRNNCDQHIDLCKHYT----SIGRCLMRFAPNYK-CVYCGM*

fr5 :VVNIGDTQFKRDIYMKRKDEYKEQHSCLRTYSLDSLA-DVSCRSNCNQHIDRCKHYS----SIGGCLMRLAPNYK-CVYCGM*

go9 :SLNIGNTQFKRNIHMKRKDEYKEKTSCLRTYSMDTHE-DISCRNNCERFIDKCQYYT----SVGGCLMKYAPNYK-CVYCGM*

ma10 :VVNIGNTQYKRTIYSRRKDEYKEKFSCLRTFSMDESS-DVLCRDNCERHIDMCKYNA----SIGECLMKFSSNYK-CIYCGM*

ov7 :VVSIGEKQYKRNVYSRRKDEYKEPTSCLRTFSIDPQS-DFACRKNCEEHIDVCKSSS----SIGECLIKHSPNYK-CVYCGM*

yo5 :TVTIGNFVYKRNIYSKRKNEYMEEFSCLRTYSMDAGL-DNICRENCEKHIDTCMRSA----VIGECLERNAPNYK-CVYCGM*

be5 :TVTIGNFVYKRNIYSKRKNEYMEQFSCLRTYSMDSNL-DNICRENCEKHIDTCMHSE----VIGDCLDRNAPNYK-CVYCGM*

ch5 :TVTIGNFVYKRNIYSKRKNEYMEEFSCLRTYSMDTNL-DNICRENCEKHIDTCMHST----VIGECLSRNAPNYK-CIYCGM*

fa8 :YARIGNTVYKRTIFSKRKDEYKEPYSCLRTFSFEKDS-DTKCRSNCEKYIDKCKHNS----SIGECLIQHSPNYK-CVYCGM*

re8 :YARIGNTVYKRTIFSKRKDEYKEPYSCLRTFSFEKDS-DSKCRSNCEKYIDKCKHNS----SIGECLIQHSPNYK-CIYCGM*

ga2 :IAQIGDFIYKRSIYSKRKDEIKEPYSCLRTFSMDSLS-DSTCRNNCHQFISECKYSS----SIGECLMKRSPNYK-CIYCGM*

ga3 :SVKINGVEYKRTVISKRKDEHKEIYSCLRTFSLDPTF-DSLCRINCELFLNNCKEFS----SIGECLRDYAPDDN-CIYCGM*
