# Supplementary material for: Clues to Evolution of the SERA Multigene Family in 18 Plasmodium Species
Source: PLoS One. 2011 Mar 15;6(3):e17775. doi: 10.1371/journal.pone.0017775 (PMC3058004; doi:10.1371/journal.pone.0017775)
Supplement: Figure S4 — Amino acid sequence alignments of Group IV Clade 6 SERA genes of P. vivax and P. vivax -related monkey malaria parasite species. The catalytic serine residue and other active site residues are shaded in green and yellow, respectively. Asterisks denote stop codons. Putative PfSUB1 recognition motifs are shaded in blue. The long deletion in truncated SERA genes of P. coatneyi, P. knowlesi and P. fragile are highlighted in black. (DOC) [file pone.0017775.s004.doc]

Figure S4 Amino acid sequence alignments of Group IV Clade 6 SERA genes of *P. vivax* and *P. vivax*-related monkey malaria parasite species. The catalytic serine residue and other active site residues are shaded in green and yellow, respectively. Asterisks denote stop codons. Putative PfSUB1 recognition motifs are shaded in blue. The long deletion in truncated SERA genes of *P. coatneyi*, *P. knowlesi* and *P. fragile* are highlighted in black.

vi9 :--------10--------20--------30--------40--------50--------60--------70--------80--------90-------100

vi9 :MKARLSLILILCVVCRDCAVRCTGTTEAQGAVEGAKGPKPGAEEAGANVGEAGTGGPGGPGADGGTEAGARAEEGEGAGTEAEPEAEPEAEAEPARGPEP

cy8 :MKARLSLILILCVVCRDCAVRCTGTNDNPGAETGKQAEERTEAGGGPGGVEGTGAGAQGVVGAAGGGVAGTGAVPGQGGAVPGPGSGEAVSLSQDAGDTP

fi6 :MKARLSLILILCVVCRDCAVRCTGNNGNPVAGTEPAPGPVTAPGGSGGQGSEQVAGGQARAEAGQGTEVGQRTGAQVQAGTGAGTGAGPGAG--------

so6 :MKARLSLILILCVLCRDCAVRCTGSNESPEVKPGPGGVAGEGTGEQAQEQEQAQVQVQAQAQAQAQVRSGTEAGGVPGVGGVPGVGGVPGVSGVGEAGAG

go6 :MKTRISLFLILCAVFSNSAIKCEGGTAGAGTTTTGQTSGISTENIQSQGGQAPQGTGEDTSQVQSPGIPSGATAPSPPINSDTPRAQDQIVTSPSP----

coT2 :MKAHLSLILILCAVCWDCAMRCTSTDGNEGQGAEEQVNADLGMDTGDNSVKGQPVVPQNGALPAENSQGVQGGPVDTSSQNPNQNEEVEKVEAGMVEA--

knT2 :MKARLSLILILCAVCRECTVRCTDTAINQGQDAVQQPEESLSQDASDNSLPGQPVASPNGAVQAEVSQLQEGAVESSSNSADASNPNANADVTDVEGEKA

frT2 :MKARLSLILILCVVCRDCVVRRTNTKDNSGEGAGAGLS*DANDKAGEDQLVASPDSTHSVETSQLAQGGTVETSNNAANNSNSN*NQNS-----------

vi9 :-------110-------120-------130-------140-------150-------160-------170-------180-------190-------200

vi9 :EPEAGGEGINRDAAGNQREGQLEAPSDSARPGAIPQVAPRDTVETSSDAADSSSPDQNPLPGADNTKVGNAATPPEGAKEETQVKSSLLKGHKGVKVTGP

cy8 :REGQPVASQDGALPAENSEDAQGRTDETSSNSVDSPSPDSNPNPVVDNAEAVTVTA-----------------PSEGPKEEIPVKSSLLKGYKGVKVTGP

fi6 :---------VVEGAGVPGVGAGG-VPGVGAGVPEGVPGGVPGGVPG-VPGVPGEVAEAEQAIA----------PSEGTKEEIQVKSSLLKGHKGVKVTGT

so6 :GEPGVGVGGVPGAGPVTGVPGAAPVTGEPAQAVEGGVPGVGAGVQGAVPEVPGAGEEVEQAIA----------PSEGAKEEIQVKSSLLKGYKGVKVTGT

go6 :-------------------------------------------------------------------------PSEGTKKEIQIKSSLLKGRKGVVVTGS

coT2 :-------------------------------------------------------------------------PSEGAKQEIQVKSSLLKGYKGVKVTGT

knT2 :AA-----------------------------------------------------------------------PSEGSKEGMQVKSSLLKGYKGVKVTGP

frT2 :-------------------------------------------------------------------------GISSATEDIQVKSSLLKGYNGVKVTGP

-------210-------220-------230-------240-------250-------260-------270-------280-------290-------300

vi9 :CGASFLVFFAPYLFIDVDTDSSNVYLGTDLSDLEVTEKMGIQDNGKN---KCEDKKTFKFVALIGEDHLTIKWKVYDPAVKTPTPNEKVEMRKYVMKNLS

cy8 :CSASFLVFFAPYLFIDVDAESSNIYLGTDLSDLEVTEKMGVGENNTN---KCDDGKTFKFVALIGDDHLTIKWKVYDPKVQTPPQDDTAEMRKYVMKSLN

fi6 :CNASFLVFFAPYLFIDVDAESSNIYLGTDLSDLEVTEKMGKEPNEKN---ICDVGKTFKFVALIGDDHLTIKWKVYDSAIQIPSPNDEVEVRKYVMKNLN

so6 :CNASFLVFFAPYLFIDVDAESSNIYLGTDLSDLEVTEKMGNEQDEKN---KCKEGSTFKFVAFIGDDHLTIKWKVYDSAVQTPSSDDQVEVKKYVMKNLS

go6 :CNASFLVFFVPYLFIDVDTESSNVYMGTDVNYLDVTEYMGNKDNKNDIKNVCENNKKFKFVTLIDDDNLILKWKVYGPNDKIPNPAEKVEVRKYKIKNLS

coT2 :CGASFLVFFAPYLFIDVDTDSSNIYLGTDLIDLEVTEQMGKEGDEKN---KCKDGKTFKFVAFIGDDHLTIKWKVYDSADQKPTQNDKAEMRKYVMKNLS

knT2 :CSASFLVFFAPYLFIDVDADSSNIYLGTDLTDLEITEKMGKGEDGKN---KCQEGKTFKFVAFVVNDHLTIKWKVYDSEDQTPTPNSNVEMKKYKVKNLS

frT2 :CNASFLVFFAPYLFIQVDAHSSNIYLGTDLSDLEVTEQMGKGEHEKN---KCEAGKTFKFVAFIEDDHLTIKWKVYDSEVQPPTPDDNVEVIKYVMKNLS

vi9 :-------310-------320-------330-------340-------350-------360-------370-------380-------390-------400

vi9 :GEFTAVQVHTVIQQNGSNVFESKNYALSSGIPEKCDAVATNCFLSGSVYIEKCYRCTLKMKKVDPSDVCYNYIPKVES----------------------

cy8 :GEFTSVQVHTVIQQNGSNVFESKNYALSNDMPEQCDAIATNCFLSGSVYIEKCYRCTLKMKKLNPSDVCYNYIPKVEN----------------------

fi6 :GEFTSVQVHTVIQQNGSNVFESKNYALSNDMPEKCDAMASNCFLSGSVYIEKCYRCTLKMKNVNPSDVCYNYVPKVENTPSQESIPAVDNTPSQESIPAV

so6 :GEFTSVQVHTVIQQNGSNVFESKNYALSNDMPEKCDAMASNCFLSGSVYIEKCYRCTLKMKNLNPSDVCYNYVPKVEN----------------------

go6 :GVFTAVQVHTALQQNGSTVFESKNYALSNDIPEKCDAIAANCFLSGNVYIEKCYKCTLKMNDENETDVCYSYVPKDAK----------------------

coT2 :GEFTSVQVHNVVQQNGSNVFESKNYALSNDMPEKCDAIAANCFLSGSVYIEKCYRCTLKMENANSSDVCYNYLPKVEK----------------------

knT2 :GEFTSVQVHSTIQQNGSNVFESKNYALSNDMPEKCDAIAANCFLSGSVYIEKCYRCTLKMEKVNSSDVCYNYIPKVDT----------------------

frT2 :GEFTSVQVHSVIQQNGSNVFESKNYALSNDMPEKCDAIASNCFLNGSVYIEKCYVCTLKMKNVNSSDVCYNYVPSVET----------------------

vi9 :-------410-------420-------430-------440-------450-------460-------470-------480-------490-------500

vi9 :--------------AASQEAIPAKASDEESSQEELTASIGKILQGVYKKGENGLNEVLTFNEADAALKAELLNYCSLMKKVDASGVLGHYQLGSEEDVHA

cy8 :--------------APSQESISAQASDEESMQEKLAASIGMILQGMYKKGETGLNELLIFDEADAALKEELLNYCALMKEVDTSGVLENYELGSEENVFA

fi6 :DNTPSQDSIPTVENTSSQESIPAVASDEDSKQEELAVSIGKILQGVYKKGETGLNELISFDQADTALKEELLNYCALMKEVDTSGVMDNYELGTEEDVFS

so6 :--------------TPSQESIPAVASDEESKQDELAASIGNILQGVYKKGETNHNELLSFEEADTALKAELINYCALMKEVDTSGVMENYELGTEEEVFY

go6 :--------------AENQETIPAKGSNEDSSEAELDASIDKILEGVYKYDKTDKKILVNFFEAEASLKAELLNYCSLLKTVDNSGILENYELGNEEQIFS

coT2 :----------------------------------------------------------------------------------------------------

knT2 :----------------------------------------------------------------------------------------------------

frT2 :----------------------------------------------------------------------------------------------------

vi9 :-------510-------520-------530-------540-------550-------560-------570-------580-------590-------600

vi9 :NLTNMLQTNSDHVLSSLQNKLKNPAICLKNADEWVGSKTGLLLPNLFYNHLEGSTPSTSNVTHVDDSSEDVQSG-------GYDGVIDFATAGKTNFSTS

cy8 :NITNMLQKNSDYSVSSLQNKLKNPAMCLKNADEWVGSKMGLVLPNLPYNHLEVPHPSTPEVANVEDTSEDTQSG-------GYDGVIDFSTASKTNFSTS

fi6 :NITNMLQTNSDYSLSSLQNKLKNPAICLKNADEWVESKKGLLLPSLSHTYVEATLPATSEVAHMNDTSEDAQSD-------GYDGVIDFATASKTNFSTS

so6 :NLTNMLQTNSDYSLSSLQNKLKNPAICLKNADKWVESKKGLLLPILSHTHVDATLPATSEVVHMNDTSEDAQID-------GYDGVIDFAMASKTNFSTS

go6 :NLTKILKKHPEETKSTLYNKLKNVAICMKNAEEWMENKKGLLLPTLSFEDMKLNHNYTAKEGEEKTVSNNNINEDEPNG--HFNGVIDLETAEKTNFSPS

coT2 :----------------------------------------------------------------------------------------------------

knT2 :----------------------------------------------------------------------------------------------------

frT2 :----------------------------------------------------------------------------------------------------

-------610-------620-------630-------640-------650-------660-------670-------680-------690-------700

vi9 :QYADKMHCNAEYCDRAKDAGSCVAKMEVQDQGDCANSWLFASKVHLETIKCVKGYDHVGASALYVANCSGKEANDKCHSPSNPLEFLNTLEETNFLPADS

cy8 :QYVDKMHCNGEYCDRTKDTSSCIAKIKAGDQGDCATSWLFASKVHLETIKCMKGHDHVASSALYVANCSGKEAKDKCQTPSNPLDFLNTLEETKFLPAES

fi6 :QYADKMHCNLEYCDRWKDDTSCVSKIEAGDQGDCATSWLFASKMHLETIKCMKGHDHVASSALYVANCSSNEAKDKCQAPSNPLEFLDTVEETKFLPAES

so6 :QYADKMHCNMEYCDRWKDDTSCVSKIEAGDQGDCATSWLFASKVHLETIKCMKGHDHVASSALYVANCSNKEAKDMCHAPSNPLEFLNTMEETKFLPAES

go6 :QFADKMYCNAEYCDRTKEENGCISKINVEDQGNCATSWIFASKLHLESIKCMKGYDHIPSSALYVANCSQKEGDAKCNTASSPLEFLNILDGEHFLPSAS

coT2 :----------------------------------------------------------------------------------------------------

knT2 :----------------------------------------------------------------------------------------------------

frT2 :----------------------------------------------------------------------------------------------------

vi9 :-------710-------720-------730-------740-------750-------760-------770-------780-------790-------800

vi9 :DLPYSYKQVGNACPEPKGHWQNLWENVKLLGPTNEPNSMSTKGYTAYQSDHFKGNMDSFIKLVKSEVMKKGSVIAYVKVAGALSYDLNGKKVLSLCGSET

cy8 :DLPYSYKEVNNVCPEPKSHWQNLWENVKLLDKQYQPNSVSTKGYTAYQSDHFKGNMDAFNKLVKSEVMNKGSVIAYVKAQGVMTYDLNGKKVLSLCGGET

fi6 :DLPYSYKALNNVCPEPKSHWQNLWANVKLLDPQNEPNSVSTKGYTAYQSDHFNGNMDAFIKLVKSEVMSKGSAIAYVKAAGALTYDFNGKKVQSLCGGET

so6 :DLPYSYKAVNNVCPEPKSHWQNLWANVKLLDPQNEPNSVSTKGYTAYQSDHFNGNMNAFIKLVKSEVMSKGSVIAYVKADELMGYDLNGKKVLSLCGGET

go6 :DMPYSYKLVGDVCPKPKHHWTNLWNNIKLLNHKNVPNSIGTKGYTAYQSEHFKNNMDEFIKIVKSEIMKKGSVIAYVKTNQMMDYDINGKTVHGICGGEV

coT2 :----------------------------------------------------------------------------------------------------

knT2 :----------------------------------------------------------------------------------------------------

frT2 :----------------------------------------------------------------------------------------------------

vi9 :-------810-------820-------830-------840-------850-------860-------870-------880-------890-------900

vi9 :PDLAVNIVGYGNYISAEGVKKPYWLLQNSWGKHWGDKGTFKVDMNGPPGCHHNFIHTAAVFNLDMPVEENPQKEDAQIYNYYLKSSPDFWGNIYYKNVGG

cy8 :PDLAVNIVGYGNYINGEGVKKSYWLLQNSWGKHWGDKGKFKVDMDGPPGCQHNFIHTAAVFNVDIPVVEKPTKEDAQIYNYYLKSSPDFWGNIYYKNVGG

fi6 :PDLAVNIVGYGNYINGEGVKKSYWLLQNSWGNHWGDKGNFKVDMHGPPGCQHNFIHTAAVFNLDIPVVEKPTKEDAQIYNYYLKSSPDFWGNIYYKNVGG

so6 :PNLAVNIVGYGNYINWEGVKKAYWLLRNSWGKHWGDKGNFKVDMDGPPGCQHNFIHTAAVFNMDIPVVEKPTKEDAQIYNYYLKSSPDFYGNIYYKNVGG

go6 :PDLAVNIIGYGNYINAEGMKKSYWLVRNSWGKSWGDKGNFKVDMHTPADCQHNFIHTAAVFNLDMPQMDVNTKQEPEIYNYYLKNSPDFYNNLYYKNFDA

coT2 :----------------------------------------------------------------------PTKEGAQIYDYYVKNSPDLEGNIHHKNVDG

knT2 :----------------------------------------------------------------------PTKEEAPIYDHCLKNSTNLGGSTSNKNVAD

frT2 :----------------------------------------------------------------------PIQEEAQIYNH-LKSSADVGDNIDYKNAGG

vi9 :-------910-------920-------930-------940-------950-------960-------970-------980-------990------1000

vi9 :QESASSKNATEGAHESVLHGQE---VAEA---AVNGAGGEPAPSHSGAVQGEREGTANGQGPEGVPPLPAKQEVTEASNGEATGLGAVNPSGEEQPGPPG

cy8 :QTNTSAKNATGVANESVLHGQADTEVEV----KV--------AGEDPATPLSTQATGGATAGQVSTVVPSLPVQVEVTVARNVGAPGDQAGSPGPAGPTG

fi6 :QKSASAKNATGVSHESVLYGQEAT-VEGANS-TV----------QGPALAHNAAEIDGRTTEQGSTSVQSSPAQQPVTVARQV--------GTTGEAVNP

so6 :QKSASAKNATGGVHESVLYGQGDA-VEGENS-AL----------QEPASLQSTAEIEGTTTGPGSTHVSPVRAPQQVTVVRDLSARVVNPSDRNQSSSSG

go6 :LKASASNKQNGFHNNSVIHGQSDDKSQGFGNLAV----------------DLLSSFTKLAGKAAEVAVDTAINAAKDVMGR-------------------

coT2 :QMNGSANNGAG-----VVQGQETAEKEVADAVKEPAPLDNTGATEGKVDEPESTHVPPLPEQEEVAGVTDVDTTEGDQPEQVELPEQPEQQGPAGPEGQP

knT2 :QEGASTINAAGGVHESVLQGQEAEEKEVKDVAVKATPESNTVVTEEKTNGQLSTEVPSLPEEAKVAEVPSAGTTEGEQPVPAVPEGQAGAPSTTGPDSTS

frT2 :EKSGSTNNATGVVHESVLQGQAAAEDGGKDAVQETAPPSSTAAVEGTTAGQVSTNAPPLPSQGEETGVLNMGTTGGEA-VNLSGDDQPDQHGTAGPDGSS

vi9 :------1010------1020------1030------1040------1050------1060------1070------1080------1090------1100

vi9 :PPGTSGLAEQPGPEGPSGEVGSTGSVGQPGQPGSSGTPRSEGQTGPSGTSGSSAAQGQSGSSGTPGSDGPSGHTGPSSTPVQEAPLSKAPGTDSPPVAPE

cy8 :PAGSPGSPGQEGQEGPAG---------------------------------PPGTPGPEGPEGSAGPTGPEGPEGSAGPTGPPGPSGTPGTEGQPGTAPE

fi6 :SGDQSRSSGDGQPAPEGTTGQAGPQGEAGSPGSAGLPGSAGTPGSVGTPGSVGTPEQPGPSGTTGADGTT----ETPGPAGPSGQPGPDGQDVPPSTEPE

so6 :DGQPGQVGSLASSVEQGTAGPQGPQGPSGPQGPPGPPGPPGTPGPSGTPGPSGTPGPSGPPGEAGTPEQTGLPAETPSQPGPDGTTGAAGQDVPPSTEPE

go6 :---------------------------------GAATTGGGGTSGTESAGTGGEQGQPGVASMSGNSLETSGSQPSGDTAAVAGPEQTPTQTASVTPPVN

coT2 :STSPEAA---------------------------------------------------------------------------------------------

knT2 :GTSPQPP---------------------------------------------------------------------------------------------

frT2 :STAPGAP---------------------------------------------------------------------------------------------

vi9 :------1110------1120------1130------1140------1150------1160------1170------1180------1190

vi9 :AAVLGSEVTHVLKYIKKNKVKLNLVTYKNHEALSSGHDCWRSYSANPDKYEECVKLCEANWSKCENDAAPGFCLYEHAKEEDCFFCYV*

cy8 :AAVLDTQVSHVLKYIKKNKVKMNVVSYKNHEAITTGHDCWRSYSVNPDKYEECVKICEANWSKCENDVAPGFCLFEHGKDNDCFFCYV*

fi6 :AAVLGTQVTHVLKYIKKNKMKMNLITYKNHEAITTGHDCWRSYSVNPDKYEECVKLCEANWSKCEGDAVPGFCLYEHAKETDCSFCYV*

so6 :AAVLGTQVSHVLKYIKKNKMKMNLITYKNHEVITTGHDCWRSYSVNPDKYEECVKLCEANWSKCENDAVPGFCLYEHAKNNDCSFCYV*

go6 :TNATSTQMLHILKRIKNSKMKTHLVMYKSHETITSGHDCSRSLSINPEKFEECVKICNENWSKCEDDPIPGICLSKHDADKYCIFCYV*

coT2 :--VLGTQLSHVLKYIKKNKVKMNLITYKNPEVISTGHDCSRSYSINPDKYEECVKICEANWSKCENDAVPGFCLYEHAKENDCFFCYV*

knT2 :--VLSTQLSHVLKYIKKNKVKMNLITYKNNEAVSSGHDCSRSYSVNPDKYEECVKICEANWSKCENDAVPGFCLYQYAKENECFFCYV*

frT2 :--LLGTQVLHLLKYIKKNKVKMNLITYKNHESISTGHDCSRSYSLNPDKYEECVKICEANWSKCEHDAVPGLCLYEHAKENDCFFCYV*
