# Supplementary material for: Clues to Evolution of the SERA Multigene Family in 18 Plasmodium Species
Source: PLoS One. 2011 Mar 15;6(3):e17775. doi: 10.1371/journal.pone.0017775 (PMC3058004; doi:10.1371/journal.pone.0017775)
Supplement: Table S1 — PCR Primers used. (PDF) [file pone.0017775.s006.pdf]

Table S1 PCR primers used.

	Forward primers (5' --->3')		Reverse primers (5' --->3')
**Degenerate primers**			
**For hypothetical protein genes conserved regions**			
Fb	CAAYRCCTTYGAAARRICWGAADTRGATTC	Rc	CCRYTRTTTATRTYTTCHRCRTCRTC
**For Group II-Group IV SERA gene conserved cysteine protease domains**			
FC	CAHACDGYWGTTKTDTTTAA	RA	TAYAYRYTRCARAAAADRCART
FE	AAHATMGAAGTRGAAGARCAAGG	RB	CTTDGTTAARCARTADCCDGG
FF	TAYTGTGAYAGRTGGMAAGATAAAAC	RC	TTAAAHAMAACWRCHGTDTG
FG	TGYAAYDANGANTAYTGYRAYMG	RD	CARTARTAHCCCCARCTRTTTC
FJ	ATNARRTGYATGARRGGNYA	RH	ATRTARTTNCCRTANCCNAYDATRTT
		RI	CNRCNGTRTGDATRAARTTRT
**For Group I SERA genes conserved regions**			
F1	GGAAAYTTYAARDTNGAYATGTATGGWCC	R1	TYACATKCCACARTADAYRCAYTTATARTT
F2	CAYTGYAADATATGYCRYAYA	R2	GADGCRAARRCCCARCARTTYCC
**For hesB gene conserved regions**			
FI	GTAGAAGCWGGDGGWTGYTCAGG	RII	CAYTTRGADAKRTTYTGWATATTYTC
FIa	GTWGAAGCWGGRGGDTGYTCAGG		
FII	ARYGAHGCWATVAAYAAAATGAAASAAATAAAT		
**specific primers**			
P. malariae			
PmaSERA1-5F	GGAAGTCTTTGCAACGTTCTACTTTATTT	PmaSERA1-3R	GGAATGAAGTAAAAAATGTTCTTAATGCTTTTACG
PmaSERA2-5F	CAGACCTTTTCTTCATATCAATGTTTTAATTTCCCTTAGGTA	PmaSERA2-3R	GGTTTTATACAAAACAGAAAAAGCAGTCATCTGTTTTCCTC
PmaSERA3-5F	CACCTCATTTTAGCTTTTACATTCAACAAAATGATGTATCCT	PmaSERA3-3R	CTGTTTCCGTTATGCGTAACTGCTATCTATGTTATATGC
PmaSERA4-5F	GATTTCTGCGACACTATATATTAATTCTGTACAACAAAAT	PmaSERA4-3R	CCCAATGTTATTTATTCTTCCAAACAGATAATCGCTTCT
PmaSERA5-5F	GAACGTAGTAATATGTGCTAATGATACAGTTACCTCCGTT	PmaSERA5-3R	GCTACTGCTGTTTCATAACGATAAAAAAATGCCGAGGAG
PmaSERA6-5F	GCAACAAGATGAAGTATGGTATTCTGTACATTTTTATG	PmaSERA6-3R	GTAAAATATTTCACACGAAACAGAAAAGGCATTTGTTCG
PmaSERA7-5F	CAGTTTACGATGTGTATGGTTCCATTAAATATATGAGCACA	PmaSERA7-3R	CTAAAAACAGGAAAGTAGCGTAGTATTATATTGTCCCTTTT
PmaG3-5F	CGTATAACCATTAATCTAACACGATGATACGACTTGTAT	PmaG3-3R	GGATTTCTCTTCTTAAAAGAGGAAAAATCAATGCATTTAA
PmaG2-5F	CACAGAACAATAATAATGTACAGGTTAAATACGTC	PmaG2-3R	CAATGACTGAAGCGATAAAGATCTCCTAC
PmaG1-5F	GATATCACGCATGATTTCTACACATTTGTCTA	PmaG1-3R	GAGGCCAAATTGGAACCACATTACATG
PmaHP1-F	GTTGGTATATGTGTATTTTTCCGTGTGAAAGGGAA	PmaG4-R	CATACGYTAAGCACCTTCTTTCCGTTAAGGTCAT
PmaSERA1-F	GCATGAAGTTATGCACAAAGGATCTGTAATTGCTTAT	PmaSERA2-R	GCCGAACTGCTCGCATGTTCATAACCCTT
PmaSERA2-F	CGTTGGAAAACCACGAACTAGCGAATCATGTAGAAA	PmaSERA3-R	GATGTGACAAGTAGTTGTGTTCGTCCTGGACT
PmaSERA3-F	GCCTTTGACAAATGTCAATAAGAAGAAAGAAGCCGAA	PmaSERA4-R	CATAGGCAATCACAGACCCTTTATGCATAATTTCATGCTT
PmaSERA4-F	GCTAAAGGATATACCGCCTATGAGAGTGACAAA	PmaSERA5-R	CACAGTTAGCTACGTATAAAGCCGAAATGCTTCCATGTT
PmaSERA5-F	GTAATGGGTTATGATCTTAACGGAAAGAAGGTGCTTA	PmaSERA6-R	CCCTTCATACATCTAATAGTTTCGAGATGTAATTTAGAT
PmaSERA6-F	CGGAAAGAAGGTGCTTAGCGTATGTGGTGATAA	PmaSERA7-R	GCGTCCATGTTTCCCTTAAATTTATCACTCTCATA
PmaSERA7-F	GCAGCCGATGATAATACGAACGAAGTTGAACTAACAGAAT	PmaSERA7'-R	CGTCAGACATTCAGAGCATCCTCTCCCTGTGTTCCTAA
PmaSERA7'-F	CAACAGTTTGAGCGCAGAAAAGGCTAATGATTTGAAGAAT	PmaG3-R	CAGTACATCTATGTTTAGGTTTCCTCTTAGAACAGTT
PmaG3-F	GGGGACAGAGTACCAGATCATGCAGCAAATAT	PmaG2-R	CTACTAGAACAGTTTGCAACAAACAATGCAGAACTTCTA
PmaG2-F	GCATCTAAGTTACATGTAGAGACTATTAGATGTAT	PmaG1-R	GTTGATGCTAAATCGTTAGGTAAGGGATTATCTA
PmaG1-F	GTAGAAGCTGGGGGTTGTTCAGGATTTCAGTA	PmaHP2-R	CAACAGCATGTTTATCTATAATTACAACACATTCCTTA
P. ovale			
PovSERA1-5F	GGATGAAGTCTCATGTTTCCATCATTGTAACACTAT	PovSERA1-3R	GTGTCAAGCATATGCACTTCATCCTTATTTAAAGT
PovSERA2-5F	GTGTAACGCAATGAAGTCACTTATTCCCTTCTT	PovSERA2-3R	GCATAAAGTCAATTTTAGTAGCAACGCTTTACACATA
PovSERA3-5F	CCTCTCTTCTTGGTGCTGTGTAAGGCCA	PovSERA3-3R	GCTTGCTGAATGCGCAATATTTTCCCTTACCTT
PovSERA4-5F	CTAAACTTACTCAGTGTTCACTAGTTGTATCCAAATGAAGA	PovSERA4-3R	CATAATCCGTTATATGCCGAAAGAGGAATATGCATA
PovG3-5F	CAAACATAGCACCTTTACCGACTGGTCGA	PovG3-3R	GAATTGGAATCCGTTTGTGCGTTCTTATACGTA
PovG2-5F	GTGCACAGAATGACACTTAAAATCTTCATAAAGAACTGT	PovG2-3R	GAGATGAGGATCATACGATGGACACTTTTTATA
PovG1-5F	CAAAATGAAGAAGGCTACTTATTTGATGCTACTCCTA	PovG1-3R	GGACAGTAATAACTCCCAGTTACATGCCGCA
PovHP1-F	CTTGAACATCGGATGAGCATCATTTTAACG	PovSERA1-R	CTTAGTCCCTACCGAATTAGGTGTAGGTACAT
PovSERA1-F	GTACAGTAGATCTGGAAACAGTAGCAAATG	PovSERA2-R	GTGTGATATTGCAGTTCCTTGTCCATTT
PovSERA2-F	GGGTTCTGTTATTGCTTACGTAAATGCAGAAAA	PovSERA3-R	CAGTTCTCACCCCAGCTGTTTCTAACAAT
PovSERA3-F	GGTAAAGAGCGAAATCAAGAATAAAGGTTC	PovSERA4-R	CTTAGTCCCTACCGAATTAGGTGTAGGTAC
PovSERA4-F	GGTAAAGAGCGAAATCAAGAATAAAGGTTC	PovG3-R	CATTTATCCTTAGGTTTAGGACACTCATTT
PovG3-F	CTGCTTTATATGTTGCGAACTGTTCAAAGAGAAAA	PovG2-R	GGATTAGAACCAACATTACATATATCTTCATGTTTTCTT

PovG2-F	CGCATTATTTATTGCAAATTGTTCGAAAAGAAAACATGAA	PovG1-R	GGTAACGGCTTATCAATTCGTTCTCCATGCATT
PovG1-F	GGGTTAGGATTCTCTGAACCCTCAGTTAA	PovHP2-R	CAGAAGCCTGTTTATCGATTATCACAACACATTCT

P. cynomolgi

PcySERA1-5F	GCCAAAATAGCATGAATCATATGGTCTCTG	PcySERA1-3R	GAACTACTTCTACGTGTATGCTCATTTATCC
PcySERA2-5F	CTACACAACAAAATGAAGTTGACCCTTC	SimG4-R1	CRWARCAGAARWARCARTCGTTTTTTTYTTC
PcySERA3-5F	CCAAGGATAATCCTTCGGCTTTTTTACC	PcySERA3-3R	CCTCTTTTAGTGACAACATGTTACACGAA
PcySERA4-5F	CTCCCTTTTACACACTTAAGAGTGGAAAA	PcySERA4-3R	GAAGTTGCACATACATACGTACCTACTG
PcySERA5-5F	GAAAGTTACACACGTACTCCAAGAGTATG	SimG4-R2	CACRAARCAGAAGAAGCADTCGTTGYTTCC
PcySERA6-5F	GAAGAGTAGAATGATTCATATCGTTTTCGT	PcySERA6-3R	CTTTTCATTGTACCGGTGAGAAGTAGCCA
SimG4-F4	GATGARGTCTAGTBTTYTYTTGTTGCTCGC	PcySERA8-3R	ATAAAAATGTGCCATTTTGATGACACAAAG
PcySERA9-5F	GATTGCAACAGAAGTAGTCAAGCCAGATT	PcySERA9-3R	CTATGCCTGACTCGTTTTACCCATTTC
PcyG3-5F	GGTTTAAAACAAATCCTACACCACACCTG	PcyG3-3R	CAAGTATCCTTTCGAAGGAACGCTAATTAA
PcyG2-5F	GTATGTGAAGTCTCAACGAATCTAGCTTGA	PcyG2-3R	CATAGGCACATCAGCCGAAAATTAACAT
PcyG1-5F	GCTTTACCTGTTTATCACTCCTGTCTACA	PcyG1-3R	CCTTATTACATGCCACAGTAAACACACTTATAG
SimHP1-F	CATATTTAAGMGAATCMRCTTCTGATTTT	PcySERA1-R	GGTCTTTTATCACCACACAGGCTATGT
PcySERA1-F	CAAATGTGAAACTGTTAGAACCCTCAGATG	PcySERA2-R	GACAGACGTTATTCACCTCTTTGTAAGAGTA
PcySERA2-F	GTTCTGTCATGACGAATGGAATAACTGC	SimG4-R2	CACRAARCAGAAGAAGCADTCGTTGYTTCC
PcySERA3-F	GGATCCGTTATCGCGTATGTGAAGGC	PcySERA4-R	GACAGACGTTATTCACCGCTTTGTAAGAGTAT
PcySERA4-F	GGATCCGCAATCGCGTATGTGAAGGC	PcySERA5-R	GGCAGACGTTATTCACCGCTTTGTAAGAGTAT
PcySERA5-F	CTTTCTCCACAGATGTCGATAAACAAGC	SimG4-R3	CCTTCACTCGTTYGCRTCYTTCAGTAG
SimG4-F3	CTACTGAARGAYGCRAACGGAGTGAAGG	PcySERA7-R	CTCGGGGAAAGAAGTGAAACGTTTTTAAT
PcySERA7-F	GGGTCCGTTATCGCCTACGTGAAGGC	PcySERA8-R	GACAGACGTTATTCACCTCTTTGTAAGAGTAT
PcySERA8-F	GGATCCGTTATCGCTTATGTGAAGGC	PcyG3-R	CTCATGAGATGGCCATACAATTTATGAAAGA
PcyG3-F	GGGTATGGAAACTATATCAATAGGAAAGGA	PcyG2-R	TAAAGCAGAGCTTCGAAAGTGACCATAT
PcyG2-F	GAGGATATGGTCACTTTCGAAGCTCT	PcyG1-R	ACTTCTCCTCGCATTGGATACACTTT
PcyG1-F	CTATGCTTCGTATACACTATGCTGCACATA	PcyHP2-R	AAAAGGAATACTGGAATCCTGAGCACC

P. fieldi

PfiSERA1-5F	GAAGTCGCAGGAAATTTAGTTCATTCAGG	PfiSERA1-3R	GTGTATGCTAATTTATCCCTGTGCGAATC
PfiSERA2-5F	GAGATCGTAGAATAGCTACTTTTAATTCCA	SimG4-R1	CRWARCAGAARWARCARTCGTTTTTTTYTTC
PfiSERA3-5F	CAAGATGAAGTTTCGCATTAGTGCCCTT	PfiSERA3-3R	GACGTAACATACACGTAGGTTCGTTTG
PfiSERA4-5F	GAATGATTCATATCATTTTCGTTGTCCCGC	PfiSERA4-3R	CTCTCATATGTAGCAGAAAAAGCAGTCGT
SimG4-F4	GATGARGTCTAGTBTTYTYTTGTTGCTCGC	PfiSERA5-3R	GTTTAACTTTCATGAAAAATGCTTCACGTGG
PfiSERA6-5F	CAACAGAAATAGTCAAGCCAGATTTGCAA	PfiSERA6-3R	GACTCGTTTTACCCATTTCGAGCTTTACA
PfiG3-5F	GTAGCAATAGTAAACTAATTCGTAGGAAGCAG	PfiG3-3R	GTTCACAATTTGGAGCTGCGTTTCTG
PfiG2-5F	GTTTCATACAAGTCAAACCAAGTGTGTAACT	PfiG2-3R	CATCAGCCGAAAATTAACATCAAAATGGAG
PfiG1-5F	GCGAAAGGACAAGTCCGATCATTACA	PfiG1-3R	CTTATTACATGCCACAGTAAACGCACTTATA
SimHP1-F	CATATTTAAGMGAATCMRCTTCTGATTTT	PfiSERA1-R	CTGCCTCTATGTTTGAGAAGCAACTAT
PfiSERA1-F	CGTTTTCAAAGATATGCACTTCCCTGGG	PfiSERA2-R	CTTCACCTCCACACAGAGAGAGAACA
PfiSERA2-F	CGAGTGTATACAGTTCTGTTATGACGAATG	SimG4-R2	CACRAARCAGAAGAAGCADTCGTTGYTTCC
PfiSERA3-F	CAGGAGCACTCAGTTACGACTTCAAC	PfiSERA4-R	CTGCGTGGTTAGGTGTCTCGCTACTG
PfiSERA4-F	GTTGGTAAAATCAGAAGTGATGAGCAAAGG	PfiSERA5-R	GAAGTGAGGCATTTTTAATTTCAAAAATGG
PfiSERA5-F	GTTATGGTAACTACATAAACGGGGAGGG	PfiSERA6-R	AAGTCGTAAGTGAGTGCTCCTGCCGCC
PfiSERA6-F	GAGTGACCACTTCAATGGCAACATGGA	PfiG3-R	GTCCATACAATTTATTAGACAGTAACTTGC
PfiG3-F	AACATTCAGAGTGGACATGCATGGA	PfiG2-R	CTCTACCTCAATGTTTGAAATACAACTC
PfiG2-F	GTGCACAGCATGTGTGGACATAATGA	PfiG1-R	TTGCATAACACTGCACCGTTACAAT
PfiG1-F	GACCTGTGCAAGCATTATACATCCATTG	PfiHP2-R	GTTTTATCCATTAGGGAAAAGGAATACTGG

P. simiovale

PsoSERA1-5F	CCAAAATAGCATGCATCATATGGTCTCTG	PsoSERA1-3R	CATATGTGTAGAAGTACTTCTACGTGTATG
PsoSERA2-5F	GCCCTTCCGTTCTTTTTCATACTAAGTAA	SimG4-R1	CRWARCAGAARWARCARTCGTTTTTTTYTTC
PsoSERA3-5F	GTATGAGGCAAGATGAAGTTTCGCATCT	PsoSERA3-3R	CATCACATTCTTTTACACAAAGCAGAAGAAG
PsoSERA4-5F	CATACTGCAAAATGAGGTCTTGCATGT	PsoSERA4-3R	CGCTGTTGTACTTGTCTCTTCAAAAATGC
SimG4-F4	GATGARGTCTAGTBTTYTYTTGTTGCTCGC	PsoSERA5-3R	CACGTAGCATTGAAGAAAAGGCTCATAAA
PsoSERA6-5F	GAAATAGTCAAGCCAGATTTGCAACCCA	PsoiSERA6-3R	CTGACTCGTTTTACCCATTTCGAGCTTTA
PsoG3-5F	CAAATACTACACCACACCTGAGCAACTT	PsoG3-3R	GATTCACAATTTGGAGTTTGGAGCTGC
PsoG2-5F	CTCAACGAATCTAGCTTAAAGGCAGTACG	PsoG2-3R	CTCTACACGCTGCAAAAAATGCAGTTA
PsoG1-5F	CGAAAGGACAAGTCCGATCATTACACAA	PsoG1-3R	GTCGAGCAAAATTATCATTTTGTGGCATTT
SimHP1-F	CATATTTAAGMGAATCMRCTTCTGATTTT	PsoSERA1-R	GGATTCGATCCAGAAGTGCATCTACTTT
PsoSERA1-F	CTTACGTCAAAGCGCTAGGCGTCTTG	PsoSERA2-R	TTGACATTTGTCCTTCGCTTCTTTACT

PsoSERA2-F	GTCATGATGAATGGAATAACTGCAAAGGAG	SimG4-R2	CACRAARCAGAAGAAGCADTCGTTGYTTCC
PsoSERA3-F	CGGACATAGATAAGCTAGATGACTGTGTT	SimG4-R3	CCTTCACTCGTTYGCRTCYTTCAGTAG
SimG4-F3	CTACTGAARGAYGCRAACGGAGTGAAGG	PsoSERA5-R	CTGACCAGTTGTGCATATCACTACATTG
PsoSERA5-F	GAAGAAGGTGCTCTCTCTGTGTGGAGG	PsoSERA6-R	GCCATTGAAGTGGTCACTCTGGTAG
PsoSERA6-F	GTTACGACCTGAACGGGAAGAAGGT	PsoG3-R	CGTATCCAATTACATCCTTCGTTTTGATG
PsoG3-F	GGTATGGTAACTACATCAATACGGAGG	PsoG2-R	CAAAGCGGAGCTTCGGAAGTGA
PsoG2-F	TGGCTACGGAAATTACATCAGTGTTAAT	PsoG1-R	CCATTCATTTGGAGCACTAGAGGAAAG
PsoG1-F	GACCTGTGCAAGCATTATACATCCATTG	PsoHP2-R	CAACGGATACCTTCAATGCTTTTGAGTT

P. inui

PinSERA1-5F	GAAGTCGCAGGAAATTTAGTTCATTCGGA	PinSERA1-3R	CAAATACTTCAACGTGTAGGCTCATTTATCC
PinSERA2-5F	GCTACTTTTAATTCTACACAACAAAATGCA	SimG4-R1	CRWARCAGAARWARCARTCGTTTTTTYTTC
PinSERA3-5F	GTGTAACAAAATGAAGTCTCGTTTGTGT	PinSERA3-3R	CATTAAGCGACGTAACATACACGTAGGT
PinSERA4-5F	GATGAAGTCTAGTCTTTTTTTGTTGCTCGC	PinSERA4-3R	CAAAGGTGATCACTCCTTATGCATACAAGA
PinG3-5F	GTTTAAAACAAATACTACACCACACGTGAG	PinG3-3R	GTTAACAATTTGGAGCTGCGTTTCTTCA
PinG2-5F	CATGCATTTCTGTGTATGTGAAGTCTCAA	PinG2-3R	CGGAAAATTAACATCAAAATGGAGCGAC
PinG1-5F	CTCGCCAGTTAACACAAAAGATCATCAC	PinG1-3R	GGAGAATATGCCTCCCTTATTCCATGC

SimHP1-F	CATATTTAAGMGAATCMRCTTCTGATTTT	PinSERA1-R	CAAATTTTCCCACAGATTCTGCCAATGAT
PinSERA1-F	CAAAGGATCCGTTATGGCTTATGTCAAAGC	PinSERA2-R	CGTCTTTATTGGAACAATTAGCCACGTA
PinSERA2-F	GAATGGAATAACTGCAAAGGAGAGTTCTC	SimG4-R2	CACRAARCAGAAGAAGCADTCGTTGYTTCC
PinSERAX-F	GTGTGCATACTTCTGCCAACATTTCCTC	PinSERA3-R	CATTAAGCGACGTAACATACACGTAGGT
SimG4-F2	GGAARCAACGAMTGCTTCTTCTGYTTYGTG	PinSERA4-R	CGTCTTTATTGGAACAATTAGCCACGTA
PhySERA4-F	GCTGTCAACATAATAGGCTATGGTAACTACAT	PinG3-R	GTTAAAATCGTATCCAATTACGTCCTTCG
PinG3-F	AAGTCTTCTGTGAGGAAAACT	PinG2-R	TATAAAAGGATCGGTCTTCCTTCTTTG
PinG2-F	AATTCTGCCTCAAGAACTGGAACTCCTG	PinG1-R	ATTGCATAACACAGCACCGTTACAATT
PinG1-F	GATCCAGCAGAGTGGACAAAGGATA		

P. hylobati

PhySERA1-5F	GAAGTCGCAGGAAATTTAGTTCATTCGGA	PhySERA1-3R	GAATTACTTCTACGTGTATGCTCATTTATCCC
PhySERA2-5F	CCTTCAGGTAGCTAACTTGAAAGGATAAG	PhySERA2-3R	GGTGGCCCACATTACAATATTTTACACG
PhySERA3-5F	GTTACAGTCACTTTTGCCTAATATCGTGTA	PhySERA3-3R	GGGATATAACATACACGTAGGTATGCTTTC
PhySERA4-5F	GGGAAGTGTTAATACAGATTTGACTTAACCG	PhySERA4-3R	CATCACATGCATGTTGATTAAGCAAC
PhyG3-5F	CAAATACTACACAACACATGAGCAACTAGG	PhyG3-3R	GGAACGCTAATTGAACATATGCATATGTTTAC
PhyG2-5F	GCCTGAACACACAGATGTTATGTTTCATAA	PhyG2-3R	GCCACATGAGCCTGAAATTAACATCAAAAT
PhyG1-5F	GCAGTTAGCACAAAGAATCATCACCTCAT	PhyG1-3R	CTTATTCCATGCCACAGTAAACGCATTTAT

SimHP1-F	CATATTTAAGMGAATCMRCTTCTGATTTT	PhySERA1-R	GGCCTCATCTGAGGGTTCTAAGAGT
PhySERA1-F	CTTAGAACCCTCAGATGAGGCCAATTC	PhySERA2-R	GAAGGTCCGATTCCGATGGCAAAAATT
PhySERA2-F	GACGAACATGCAGTCCACCAGTTTTATT	SimG4-R2	CACRAARCAGAAGAAGCADTCGTTGYTTCC
PhySERA3-F	CAAATTGGTAAAGTCAGAAGTGATGAACA	PhySERA4-R	GTCTTCTCTAGGATGTCCAAAAATTCCAAC
PhySERA4-F	CAAATTGGTCAAATCCGAAGTGATGAACA	PhyG3-R	CGACTGTACAGATCTCTTTTTTGTTCCTTT
PhyG3-F	GGGTGAAGAGATCCTACTGGATTATTA	PhyG2-R	TTGTTCCTCTACCTCAATGTTTGAAATG
PhyG2-F	TGCACCCATTTCTGCCTCAAGAACT	PhyG1-R	TAAATCGTTAGGAAGCGGTTTGGTAATTAT
PhyG1-F	GTCCTCTGTAGGAACAACTGCCATAA	PhyHP2-R	GAGGAACTCTCTATCATAAACGACTAACTC

P. coatneyi

PcoSERA1-5F	GAAGTCGCAGGAAATTTAGTTCATTCGA	PcoSERA1-3R	CTCACACGTAGCAGAAAAAGCAATCGC
PcoSERA2-5F	GTCGTAGAATAGGTACCTTTAGTTCTACAC	PcoSERA2-3R	GTGGCCTAAATTACAATATTTTACACGTAG
PcoSERA3-5F	CCATCACCCGTATATGACATGATGAAGG	PcoSERA3-3R	CACACGTATGTTTCTTTGCCCGATTTAA
PcoSERA4-5F	GATGAAGTATAGTTTCCTCTTATTGCTCGC	PcoSERA4-3R	GCATTAACATTTGCACACTCTCATGCAT
PcoTSERA2-5F	GACAAAATGAAGGCTCACCTTTCCTTAAT	PcoTSERA2-3R	CTCGTTTTATCCATTCCGACATTTACACATAA
PcoG3-5F	CTATACCACACCTAAGCAACTTGGCAG	PcoG3-3R	GTTTACAATTTGGAGCTGAGTTTCTGCG
PcoG2-5F	CAACGAATCTAGTTTGAAGGCAGTACGA	PcoG2-3R	GTTCCTTTTGAACCTCTCTAGACGCTG
PcoG1-5F	GCGAAAGGACAAGTCTGATCACTACAT	PcoG1-3R	CTGATTATTTGCCACGTCGAGCAGAA

SimHP1-F	CATATTTAAGMGAATCMRCTTCTGATTTT	PcoSERA1-R	CTCTATGTGTGAGAAGCAACTATTTTTATC
PcoSERA1-F	CCACGCAGTGAACATAATCGGCTAC	PcoSERA2-R	GCAAAAAGCCATGACGTGGCACAGTC
PcoSERA2-F	GTACAAACTAACGGTTACGATGGTGTTATC	PcoSERA3-R	GCGTTTCTTCACTTCCACATAGAGACAGT
SimG4-F2	GGAARCAACGAMTGCTTCTTCTGYTTYGTG	PcoSERA4-R	GTGTTACTTGTACTTGTGTATCCGAAACTG
PcoSERA4-F	GGAGTGAAAAAATCCTATTGGTTGTTGCA	SimG4-R5	CATTTCGAGCTYTAYACATAGCAGAAGRAAC
SimG4-F5	GHTATGYGAAGCYAACTGGAGCAAATGTGAA	PcoG3-R	CAGTTTGCAACGTACAATGCTGAGCTAC
PcoG3-F	GGTACACAACATGTGTGGTGATAAAGTA	PcoG2-R	CTTTCTTCTGGATCTCTCTTAGAGCAATTC
PcoG2-F	TGGTTACTGCTTAACTAAGATGTACACGG	PcoG1-R	GCATAACACTGCACCGTTACAAT
PcoG1-F	CCTGTGCAAACATTATACGTCCATT	PcoHP2-R	CTACAGATACCTTCAAGGCTTTTGAG

P. knowlesi			
PknSERA1-5F	GGAAATTTAGTTCATTCGATGAGACGCCA	PknSERA1-3R	GCCCATATTACAATGTTTTACACGTAGCAG
PknSERA2-5F	GCGCTAAGCATAATTTCTCTGCTTTTTCT	PknSERA2-3R	CATAGGTTTGTGTGCTCAATTTAGCAAGGA
PknSERA3-5F	CTTAATGCAGATTTAACTGAACCGAAGCAA	PknSERA3-3R	CATCAACACATGTATGTGCATCGAGAAAC
PknTSERA2-5F	CCCACCTCTTGATAGGACTAGGACAAAA	PknTSERA2-3R	GTGACTCTTTTTACCCATTTCGAGCTTTA
PknG3-5F	GACATCTGAGGAATTGTCAATCGAGCAA	PknG3-3R	CACATTTTGGAGCCAAGTTCCTGCTTAT
PknG2-5F	CAACGAATCTAGTTTGAAGGCAATACGAC	PknG2-3R	CTGGAGATAACCCAAATCATTGTGGTTAC
PknG1-5F	CATTACCTGTTCTTCACTCCTGTCTACG	PknG1-3R	CTATTCCTCAACCGTCTTGCCACGAATA
SimHP1-F	CATATTTAAGMGAATCMRCTTCTGATTTT	PknSERA1-5R	CCAGGTGAAAGAGATCCTGGGATTCAT
PknSERA1-3F	GATTGCTTCTTCTGCTACGTGTAAAACATT	PknSERA2-5R	CATTGTTACCTATTAGCAGAAGGGCACAG
PknSERA2-3F	GAAACAACGAATGCTTCTTCTGCTTTGT	PknSERA3-5R	GCGAGCAACAAAAGAAAACTAGACTTCATC
PknSERA3-3F	CGAATGACTGTAATTTTTGTTTCGTGTGAA	PknTSERA2-5R	GTATTAAGGAAAGGCGAGCCTTCATTTTG
PknTSERA2-3F	CAGTGCCAGGATTCTGTTTATACCAATA	PknG3-5R	CTAAAATAAGAATAAGGGCCACGGGTCG
PknG3-3F	GAAGAATGCAAACTGTTCTGTGAGGAAAAC	PknG2-5R	CTTAAAAGGACGGAAAAGCGAGAAACC
PknG2-3F	AAATAACTGCATTTTCTGCAGCGTGTAG	PknG1-5R	GAACACCACGTACTGTGCTATCTGATTTA
PknG1-3F	GACCTGTGCAAACATTATACATCCATTGG	PknHP2-R	GTTTGATATCAAACGAGTTGCCACAGG
P. fragile			
PfrSERA1-5F	CATATCCTTTTCATCGTCTCGCATCCTG	PfrSERA1-3R	CACTCGGTCTCCACAAAAACTGTATCAT
SimG4-F3	GATGARGTCTAGTBTTYTYTTGTTGCTCGC	PfrSERA2-3R	CATTCGCAGATGCATCAGAACGTGTATG
PfrTSERA2-5F	GAAATAGTCAAGCCAGATTTGCAATCCA	PfrTSERA2-3R	GACTCGTTTTACCCATTTCGAGCTTTTTA
PfrG3-5F	GGTTTAAAACAAATAACTACACACCACACCTG	PfrG3-3R	CCTCAGACATAGCAGAAGTAGCAGTTG
PfrG2-5F	GCATCCATTTCTGTGTATGTAAAGTGTCAAC	PfrG2-3R	GACTAAAATTAACATCAACATGGAGCGACG
PfrG1-5F	GCTTTACCCACTCTTCACTCCTGTCTA	PfrG1-3R	CAACAGGTACAACTTAGGGAGAATCTGTTT
SimHP1-F	CATATTTAAGMGAATCMRCTTCTGATTTT	PfrSERA1-R	CATTTGTCATGCGCTTTATTATTCAAACAGTTG
PfrSERA1-F	GAGGAGGGAATGAAAAAGTCCTACTGGTTGTTA	PfrSERA2-R	GAGTATCTCCCCATCAGATATAGCTCC
PfrSERA2-F	CTTCAACGGTAACATGGACGCGTTTAT	PfrG3-R	GTACATCATAAAATGGCCATACAATTTGTGAG
PfrG3-F	CCAACTGCTCCAAAAGGAACCGAAA	PfrG2-R	GATCTGCATCAAGTTTTCCACATATTAAG
PfrG2-F	TTAACGAAGATGTACACGGGCACTA	PfrG1-R	CCTGATAAGAAGCAATTCCGTATGGC
PfrG1-F	GGGTATCTGAAATGACTGCTACGATATGGG	PfrHP2-R	AAAGGAATACTGGAAGCCTGAGCAT
P. gonderi			
PgoSERA1-5F	CAATCGTAGAACAGTTTGTTTTAATTGTACACC	PgoSERA1-3R	GCAATATCTAACCACGCCCTTTAACTATATG
PgoSERA2-5F	GCAAGATGAAGTCTCATCTGTTGTTTCTCT	PgoSERA2-3R	CTTGCAAATTGAAAATAATTGCTCAAAGCG
PgoSERA3-5F	GAAGCTTCGAACTTACGTCCTTCTCATAAC	PgoSERA3-3R	CTGTTTTACACAAAGCAGAAGAAGCAGTC
PgoSERA4-5F	GTGTGAATAGTACGAGTCCTTAGAACATTT	PgoSERA4-3R	CGCACCTCTCAAATCATAAATTAGCTTTCA
PgoSERA5-5F	GCAACAAGATGAAGTATAGTATTCTCTTCCTT	PgoSERA5-3R	CATAAAAGGGTTCACATATTCTTGCATGCA
PgoSERA6-5F	GATAATAAGACGAAATGAAAACCCGTATTTCC	PgoSERA6-3R	GGAAAAGAAAATAGCTTACGCATTTCACAC
PgoG3-5F	CCAGTTTTGATGAAAATGATAATACGTCCC	PgoG3-3R	CCAGATCCTTTTAGTGAAAATCCTGAGC
PgoG2-5F	GAAACCAATAGGACAAAATGATTCGTCGT	PgoG2-3R	CACGTCCTACACATTACAGAAAAAGCAG
PgoG1-5F	GCTTCCTCAACTTAGTCTAAAATGAAAGCA	PgoG1-3R	CATTATGAATGAGGTGAAACCATCACCC
SimHP1-F	CATATTTAAGMGAATCMRCTTCTGATTTT	PgoSERA1-R	CAGACATCGCCAACCAGGTTGTACGAATA
PgoSERA1-F	GAATATTTGAACATTCTTGATGGCGAGAAG	PgoSERA2-R	CAGTTAGCCACATATAAAGCAGAACTAGGAAC
PgoSERA2-F	GTAGGTAGTCATCAATCTCCATCCTTTGCT	SimG4-R2	CACRAARCAGAAGAAGCADTCGTTGYTTCC
PgoSERA3-F	CCCGGGTATTGTTTAGCTAAGAAGAAGG	PgoSERA4-R	CGAGTTCTTCTTCGAACCAGTTTCGAAG
PgoSERA5-F	GTGAATATAATCGGCTATGGAAACTATA	PgoSERA6-R	CATTTGGTTAGTCTTTACATATGCGATGACTG
PgoSERA6-F	CTTACCATCTGCATCTGACATGCCATATTC	PgoG3-R	GAATTCTATAGGATTTGATCCCACTGTACATATATC
PgoG3-F	TAGATATGCACGGACCCAAGAATTGTAAAT	PgoG2-R	AACAGAGACCACAATCTCCTTGTTCT
PgoG2-F	TATGGGCCAAAAAAATGTGAACAC	PgoG1-R	GCATTACATAATACTGCACCATTACAA
PgoG1-F	GAACGATTTATTGACAAGTGCCAGTATT	PgoHP2-R	GCAGCCTGCTTGTCAATTATTACAATAC

* The location of each primers in the *Plasmodium* SERA gene family shown in Figure S5
