# Supplementary material for: Clues to Evolution of the SERA Multigene Family in 18 Plasmodium Species
Source: PLoS One. 2011 Mar 15;6(3):e17775. doi: 10.1371/journal.pone.0017775 (PMC3058004; doi:10.1371/journal.pone.0017775)
Supplement: Table S2 — SERA gene accession numbers in PlasmoDB database. (PDF) [file pone.0017775.s007.pdf]

Table S2 SERA gene accession numbers in PlasmoDB database [a]

	Plasmodium species (strain)				
Gene	*P. vivax* (SalI)	*P. falciparum* (3D7)	*P. berghei* (ANKA)	*P. yoelii* (17XNL)	*P. chabaudi* (AS)
SERA1	PVX_003850	PFB0360C	PB000108.03.0	PY00291	PCAS_030730
SERA2	PVX_003845	PFB0355C	PB107093.00.0	PY00292	PCAS_030720
SERA3	PVX_003840	PFB0350C	PB000107.03.0	PY00292	PCAS_030710
SERA4	PVX_003835	PFB0345C	PB000352.01.0	PY02062[b]<br>PY00294[b]	PCAS_030700
SERA5	PVX_003830	PFB0340C	PB000649.01.0	PY02063	PCAS_030690
SERA6	PVX_003825	PFB0335C			
SERA7	PVX_003820	PFB0330C			
Truncated SERA1	PVX_003815				
SERA8	PVX_003810	PFB0325C			
SERA9	PVX_003805	PFI0135C			
SERA10	PVX_003800				
SERA11	PVX_003795				
SERA12	PVX_003790				

[a] PlasmoDB; http://plasmodb.org/plasmo/

[b] The gene region of PY02062 and PY00294 was re-annotated and used as *P. yoelii* SERA4.
