# Supplementary material for: Clues to Evolution of the SERA Multigene Family in 18 Plasmodium Species
Source: PLoS One. 2011 Mar 15;6(3):e17775. doi: 10.1371/journal.pone.0017775 (PMC3058004; doi:10.1371/journal.pone.0017775)
Supplement: Table S3 — Primers and probes used for real time PCR. (PDF) [file pone.0017775.s008.pdf]

Table S3 Primers and probes used for the Real time PCR works

	Forward primer (5'--->3')	Reverse primer (5'--->3')	TaqMan probe (5'--->3')
P. cynomolgi			
PcyTBB	CCATGTTTAGAGGACGAATGTCAA	GGAATATCACAAACGCTGGACTT	CAGATGCTCAGCGTACA
PcySERA2	AAGCACGATTGTTCGAGGTCTT	GAACTGTATGCACTCGGCTAGCT	TTCGCAGGACCCAGAG
PcySERA3	TTGACGGGTAGCACAGGAGTT	CCAAGGCATCTGCATTATCGT	CTCCATTTCTTGAAGAACGT
PcySERA4	CACCGTCGAGTCCGAGGTTA	TCCACGGCATACGCTCTAGA	CCTTCAAATTCTGAAGCAT
PcySERA5	GAGGAGCGGATCAACAAGTATCA	TTGCCCTGTAGTTCCCGTAGTC	CATTACCAGGAACTCC
PcySERA6	TACGACAGTGATGCAGCGATAA	ACTCATTCCAGTGGTCATCACAA	CTCACGAGCAGTGGCA
PcySERA7	TGGTGACGAGAATGGTCACGTA	TCCAGCTTCTCCACAGTTGATG	CGAATTGGGAGACCAC
PcySERA8	CGCTGTGCTAGACACCCAAGT	GATAGCCTCGTGATTCTTGTACGA	TCACATGTCCTAAAGTAC
PcySERA9	GCTGAAGCATATTGACAAGTCCAA	CCTCACAGAAGAGTTTGCATTCG	ATGACCACAGCTGCG
PcySERA10	AGGGCATTCGTGAGGTACGA	CAGGAGGCCCAGTTCTTGAG	TGCATCCAATTCTG
PcySERA11	GCGGACCTACTCCCTGGATT	CATCCCAATGGACGTATAATGCT	CCTAGCGGACGTCTC
P. coatneyi			
PcoTBB	CAGATGCTCAGCGTGCAGAA	TGTCACAAACGCTGGACTTAGTG	CTACTTCGTCGAATGGA
PcoSERA2	CGGGTAGCCATGTCAGATGA	GAGCGAGATGTTACGGGTTGA	CACACCATGGCGTCG
PcoSERA3	GCTATCGGAATTAACCGGATCA	TGGCACTCTCATCATCGTACGT	CGTTCAAGGCGTAACAA
PcoSERA4	CAATGCAGGAATGGAAATCGA	TCCCAAGTCGTACAGACCTTCAT	TTCACGTTCTGAAACAC
PcoTSERA2	CGTGGATACGACAGAAGGTGATC	CATGTGACAGTTGGGTGCCTAA	CCAGCCTAGCACGTCA
PcoSERA5	ACTACGATGATGAGGCGGCTAA	TCCACTTGTAGGCACTACCACTGT	CAAAGTACCGAAACAAG
PcoSERA6	CACGTGCTTATATTGACGATGATG	CCTGACCGTCGACTACAGCAT	CGTGCTTGGTGAGAAG
PcoSERA7	GCGGACCTACTCCCTGGATT	AGACACCTCCCAATGGACGTAT	CCTGCAGGGACAACT
P. knowlesi			
PknTBB	CCTCCTACTTCGTCGAATGGAT	CCCTTTGGTGGAATGTCACAA	CACCAAGTCCAGCGTT
PknSERA1	GGTCAACTGGAGGCAAATTCTG	CCTCGTCACTGTCACCTTACACA	CCATTGCAGCGTCTG
PknSERA2	AAATGGAACTGGCGGATTGA	TCTGCACTCGGACCGCTATT	TCCCTGGAAATACG
PknSERA3	TCACGAACCGAAGCATCATC	TCGACAATTTGCACTCGGAAT	CTGGACGAGTGCATAAA
PknTSERA2	TGGACACGACTGCTCGAGATC	TCTTCACGCACTCTTCGTACTTG	CTCGGTGAATCCAG
PknSERA4	GAGGAAAACTGGAACAACTGCAA	CGCTGTACAACTTGGACAGACAA	ACCACTACTCTCCTGGCT
PknSERA5	GTGGGAGGTGCAGTTACACTCA	TCGCTCAAACTGTCGTAATTCAC	CCGTGGACAATTCA
PknSERA6	TCCCAAGCAGACGTATCCTGTA	GAGACACCTGCCAATGGATGTA	CAGCATATTGACCTGTGC
P. berghei			
PbeTBB	GCTCCTTAAGATTTCCAGGTCAAC	TCCTCGACTTGTTAAAGGAGCAA	TTCCATTCCCACGTTTAC
PbeSERA1	TGAACCTGCCTCAATTTCTACACA	GCCTGTTGATGGTATTTCTGATCTAC	CACATCAACCAACTGG
PbeSERA2	AAGCCAAACATGCCCAAGTT	GATCCGGAAGATGAACCTGAAG	AGCTGTCACACCAAGTC
PbeSERA3	TTCAAATCCTAATGCCCAAAGC	AATTGATGCGGCATTTGCA	CCGTTACAGACACAGTCA
PbeSERA4	TTCGAGGGTACAAAGTATTGGTTTAA	TTCACCAGAATATAAGGTTGCCAAA	ACACTTCTCCTGGATATT
PbeSERA5	CCCTCCAGAGATCCTGAATACG	CACTAGGATCAAATCGTCGATTGT	CCTAGTTACCCAAGTATG
